# Unified Approach to Imidodiphosphate-Type Brønsted Acids with Tunable Confinement and Acidity

**DOI:** 10.1021/jacs.1c07067

**Published:** 2021-09-03

**Authors:** Sebastian A. Schwengers, Chandra Kanta De, Oleg Grossmann, Joyce A. A. Grimm, Natascha R. Sadlowski, Gabriela G. Gerosa, Benjamin List

**Affiliations:** Max-Planck-Institut für Kohlenforschung, Kaiser-Wilhelm-Platz 1, 45470 Mülheim an der Ruhr, Germany

## Abstract

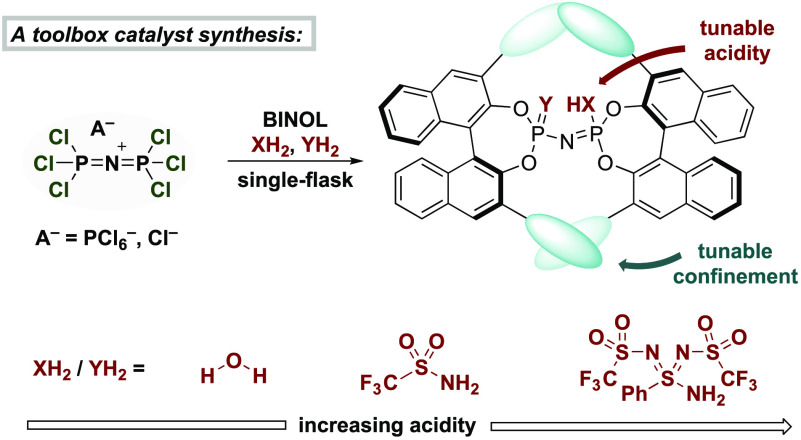

We have designed and realized an efficient and operationally simple single-flask synthesis of imidodiphosphate-based Brønsted acids. The methodology proceeds *via* consecutive chloride substitutions of hexachlorobisphosphazonium salts, providing rapid access to imidodiphosphates (IDP), iminoimidodiphosphates (*i*IDP), and imidodiphosphorimidates (IDPi). These privileged acid catalysts feature a broad acidity range (p*K*_a_ from ∼11 to <2 in MeCN) and a readily tunable confined active site. Our approach enables access to previously elusive catalyst scaffolds with particularly high structural confinement, one of which catalyzes the first highly enantioselective (>95:5 er) sulfoxidation of methyl *n*-propyl sulfide. Furthermore, the methodology delivers a novel, rationally designed super acidic catalyst motif, imidodiphosphorbis(iminosulfonylimino)imidate (IDPii), the extreme reactivity of which exceeds commonly employed super-Brønsted acids, such as trifluoromethanesulfonic acid. The unique reactivity of one such IDPii catalyst has been demonstrated in the first α-methylation of a silyl ketene acetal with methanol as the electrophilic alkylating reagent.

## Introduction

**Figure 1:**
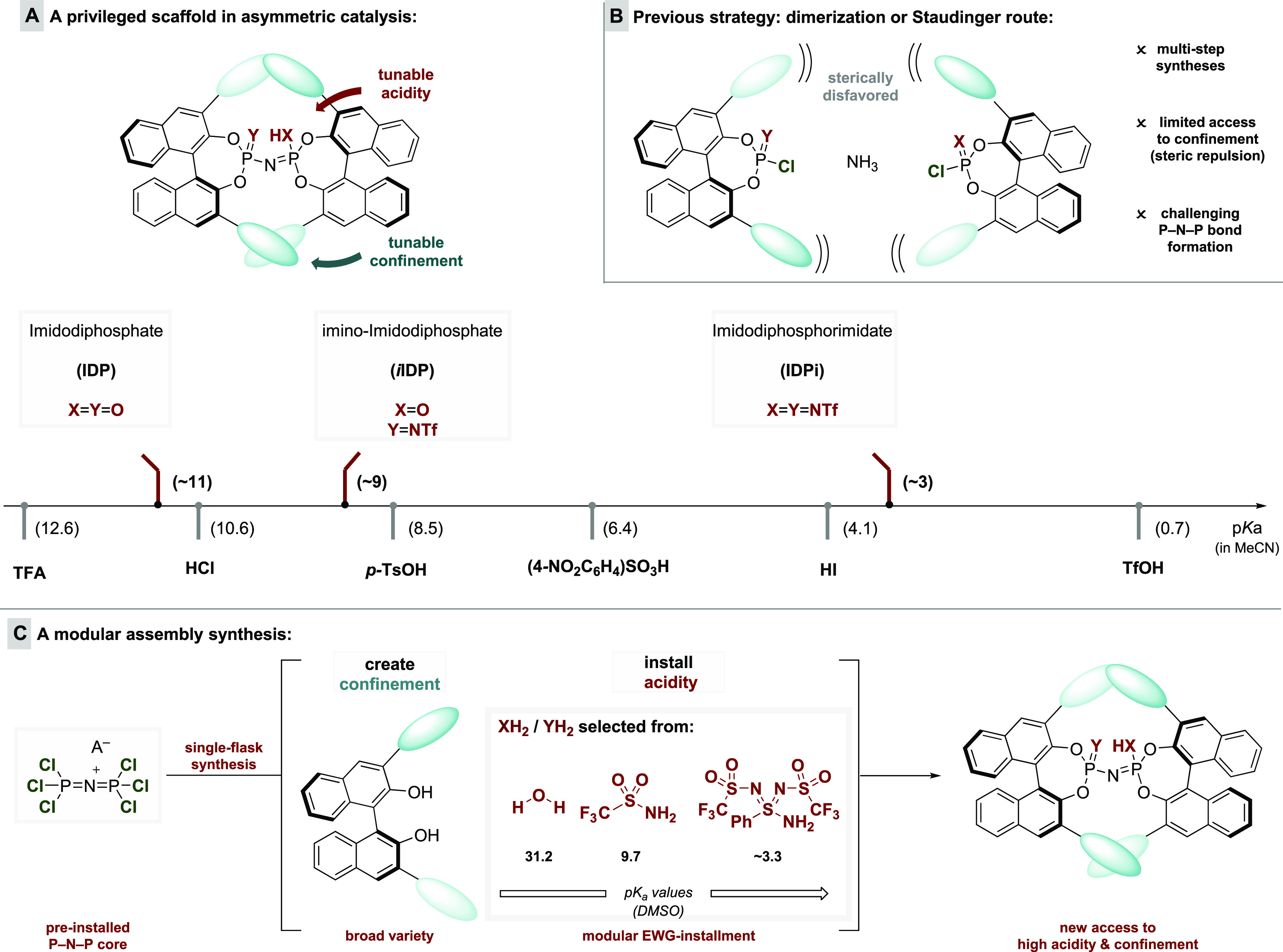
Synthesis of imidodiphosphoryl-derived Brønsted acids.

Acid catalysis is arguably the single most general approach to catalysis there is. It enables the activation of diverse and inherently distinct substrate classes, which, at least in principle, as a necessary and sufficient condition, only require electron density and as such, the potential to catalytically activate the vast majority of all chemical materials exists. It is therefore perhaps not surprising that acidic catalysts have become indispensable tools for chemical synthesis as well as an enabling technology for multimillion-ton-scale productions.^1^ During the last two decades, organic Brønsted acids have enriched the arsenal of asymmetric catalysis, initially in bifunctional catalysts such as proline or BINOL-derived phosphoric acids (CPA),^2,3^ and lately also in more purely acidic motifs.^4^ In this context, we have generalized the underlying principle of asymmetric Brønsted acid catalysis, in which protons act as the activating principle while chiral, enantiopure anions enable enantiodifferentiation, toward asymmetric counteranion directed catalysis (ACDC), including all types of cationic activation principles.^5^ The high versatility of Brønsted acids inspired the development of ever more acidic catalysts to overcome intrinsic reactivity barriers of weakly basic substrates.^6^ However, the highly selective conversion of small and constitutionally unbiased substrates has long remained challenging due to the rather open active site of most Brønsted acid catalysts and the resulting conformational freedom of protonated reactive intermediates and transition states. To overcome these limitations, our group has conceptualized, designed, and established confined acids, the corresponding bases of which possess highly compact anionic active sites. Such counteranions are suggested to formally bind and stabilize cationic transition states of reactions involving small, unfunctionalized substrates. In 2012, we introduced the first generation of such catalysts, dimeric *C*_2_-symmetric imidodiphosphates (IDP).^7^ With their four 3,3′-substituents on the binaphthyl backbone, these catalysts provide a well-defined and very tight microenvironment. IDP catalysts have consequently emerged as powerful and versatile catalyst scaffolds, somewhat resembling enzymatic substrate recognition. Due to the diversity of the substituted and modified BINOL backbone, a broad range of distinct cavities, displaying designable substrate–class recognition, are readily accessible and enable highly stereoselective transformations of previously elusive substrates.^8^ However, whereas IDPs (p*K*_a_ ≈ 11 in MeCN) are significantly stronger acids than chiral phosphoric acids (CPAs, p*K*_a_ ≈ 13 in MeCN), their acidity is still moderate, limiting their applicability to relatively basic substrates such as imines, enol ethers, and certain carbonyl compounds. The replacement of oxygen atoms with one or two NTf groups led to the development of *i*IDPs and IDPis, respectively, comprising high and tunable acidities (p*K*_a_ ≤ 2–9 in MeCN, Figure 1A), in combination with excellent stereoinduction from the enantiopure counteranion.^9−11^ IDPis have also found utility as precatalysts for powerful and user-friendly silylium-based Lewis acid catalysis and have enabled extremely challenging transformations.^12^ The combination of modular acidity and tunable confinement has led to unprecedented and unusual transformations in organocatalysis, such as an organocatalytic olefin activation,^13^ the selective monoaldolization of acetaldehyde enolates,^14^ a widely applicable Prins cyclization,^9^ or a challenging Mukaiyama aldol reaction with sub-ppm catalyst loadings^15^ and the handling of nonclassical carbocations.^16^

Our previous approach to imidodiphosphoryl-type scaffolds relied either on the dimerization of two phosphoryl halide moieties (Figure 1B) in the presence of ammonia or a surrogate for the synthesis of IDPs and IDPis or on a Staudinger approach of a phosphoryl azide with an *N*-sulfonyl phosphoramidite to furnish *i*IDPs.^7,9,10^ The synthesis of IDP and *i*IDP catalysts required the preformation and isolation of the corresponding monomeric phosphoryl units prior to the dimerization/Staudinger approach for each catalyst synthesis. Additionally, *N*-sulfonyl substituent modifications for the *i*IDP and IDPi motif rely on the preparation and isolation of *N*-sulfonylphosphorimidoyl trichloride or *N*-sulfonyl phosphoramidites, respectively, for every core modification, resulting in a time-consuming catalyst library establishment.^17^ Importantly, the dimerization process is strongly influenced by steric properties of the 3,3′-substituents on the BINOL moiety and occasionally provides unsatisfactory yields, proceeds under harsh reaction conditions, or requires prolonged reaction times. BINOLs with highly sterically demanding substituents often do not furnish the desired imidodiphosphoryl motif due to steric repulsion in the dimerization process (Figure 1B). We became highly motivated to address this problem since we are particularly interested in catalysts possessing extreme confinement in combination with extreme acidities, which we deem a requirement toward handling very small *and* nonactivated substrates.^11^ We now report a new, unified, general, and user-friendly synthetic strategy toward imidodiphosphate-type motifs. A particular focus is given to previously elusive catalyst scaffolds and toward the development of even more acidic imidodiphosphoryl-based Brønsted acids, which overcomes remaining reactivity barriers and facilitates the development of novel transformations within the ACDC framework.^5,18^

## Results and Discussion

**Scheme 1:**
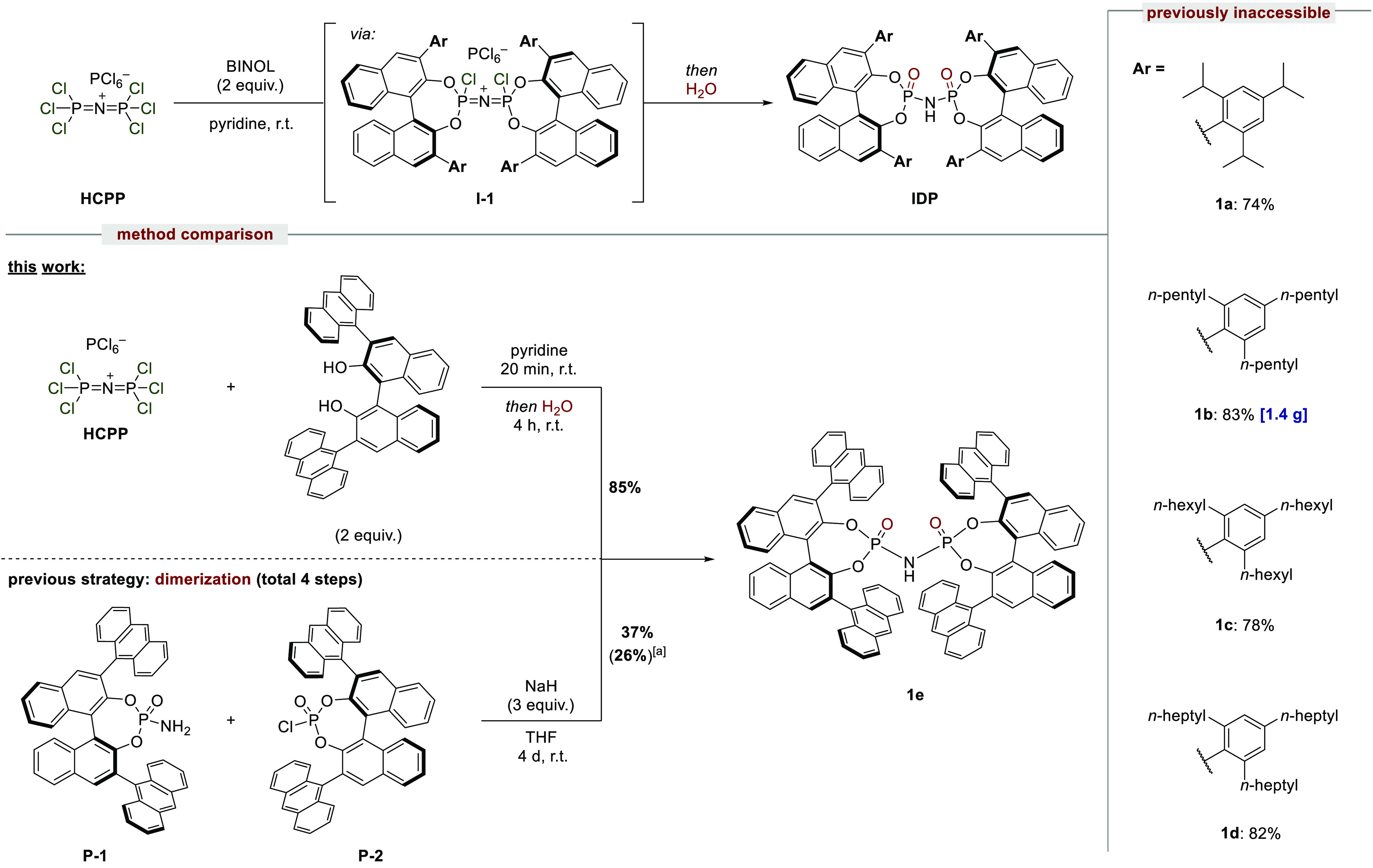
IDP Synthesis and Reaction Scope Yield over four steps from BINOL.

To circumvent the limitations of our earlier developed methods, and to establish a more efficient, straightforward, and operationally simple catalyst synthesis, we envisioned utilizing hexachlorobisphosphazonium hexachlorophosphate (HCPP), initially reported by Becke-Goehring,^19,20^ as a platform molecule for the synthesis of dimeric imidodiphosphoryl-derived Brønsted acids (Figure 1C). Using HCPP as the starting material would bear the following advantages: (a) the P–N–P core is already preinstalled, avoiding inefficient dimerizations by mitigating steric repulsion during the dimerizing event; (b) intermediate **I-1**, which we expected to form upon treating HCPP with two BINOLs (Scheme 1) would be functionalizable by simple chloride substitution with suitable electron-withdrawing groups (EWGs), e.g., sulfonamides; (c) all previously mentioned imidodiphosphoryl-type Brønsted acids would be accessible from the same common intermediate **I-1**; (d) HCPP is readily available in a single step on decagram scales, stable, and would allow simplified large-scale catalyst syntheses and ideally furnishes the desired products in high yields with single product isolation and simplified purification procedures.

We started exploring the reactivity of HCPP by focusing on the synthesis of imidodiphosphates (Scheme 1). We found that, in pyridine, a rapid reaction of HCPP with different BINOLs occurs, resulting in the formation of intermediates **I-1**, which upon addition of water readily provides the desired IDP products. Remarkably, IDPs **1a**–**d**, which were inaccessible with our previously established method, likely due to high steric repulsion within the dimerization, are now readily available. Furthermore, we compared the efficiency of our new methodology to the previously established dimerization approach. Phosphoryl amide **P-1** and phosphoryl chloride **P-2** were independently synthesized and reacted with sodium hydride to furnish the desired IDP **1e** after 4 days in 37% yield.^7^ In contrast, our new methodology provides IDP **1e** from the corresponding BINOL in less than 5 h and in 85% yield, which only requires a single and simplified purification step.

**Scheme 2:**
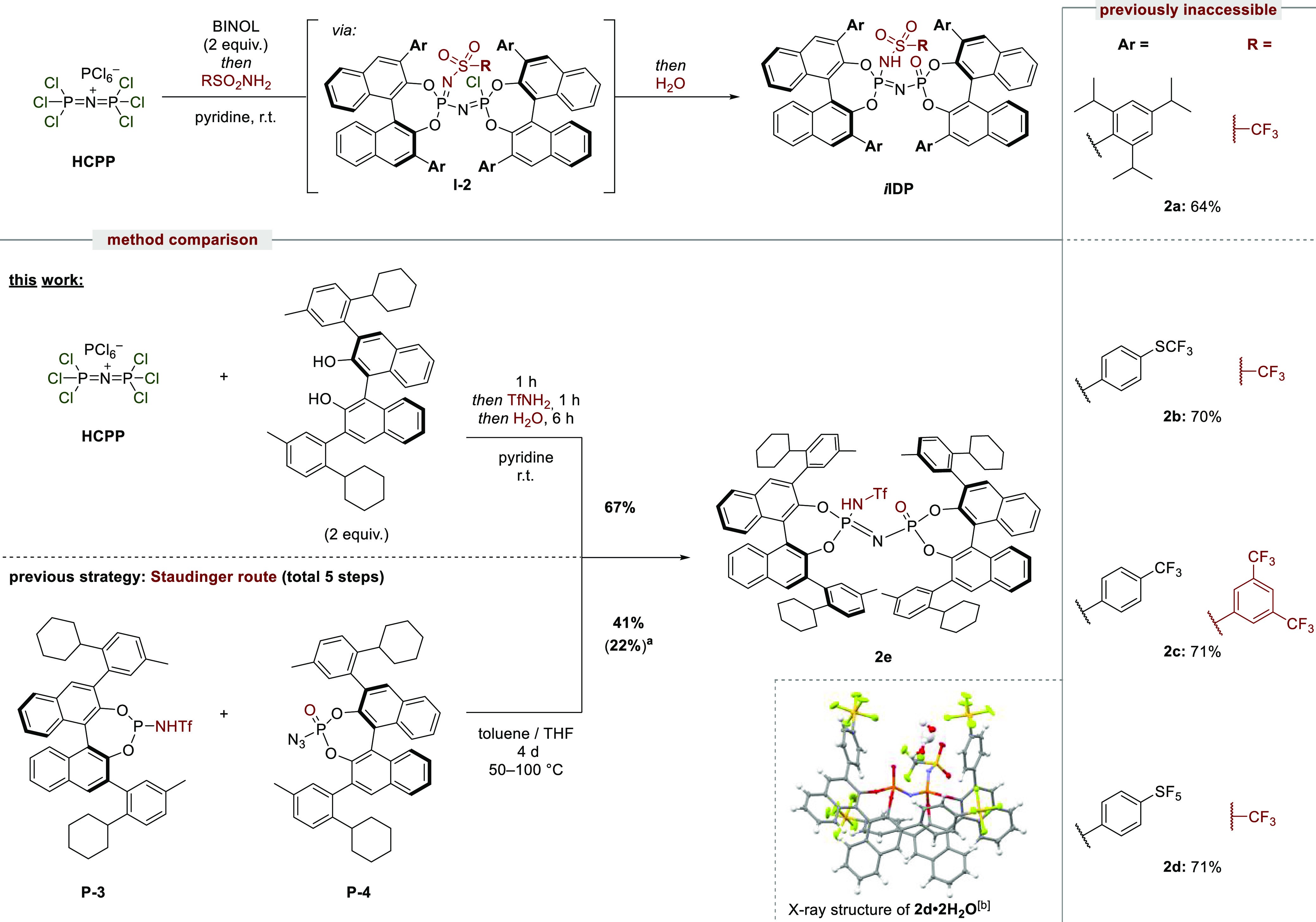
*i*IDP Synthesis, Reaction Scope, and Single-Crystal Structure of **2d**·2H_2_O Yield over five steps from BINOL. Two disordered CH_2_Cl_2_ molecules are omitted for clarity.

With the newly established procedure toward IDPs, in which salt **I-1** was found to be the key intermediate, we envisioned that substituting a chloride of **I-1** with trifluoromethanesulfonamide (TfNH_2_), followed by hydrolysis, should furnish the corresponding *i*IDP motif. Owing to its enhanced acidity but relatively complicated previous synthesis, an expeditious route to this catalyst class is particularly attractive. Indeed, due to the highly electrophilic character of intermediate **I-1**, a rapid substitution of chloride with TfNH_2_ occurs within minutes, resulting in the formation of neutral intermediates **I-2**, which upon hydrolysis with water afforded the desired *i*IDP products (Scheme 2). Our modular approach enables previously unexplored BINOL and sulfonamide combinations, smoothly providing *i*IDPs **2a**–**d** in good yields, following a single-flask procedure and a simplified purification. Once again, the TRIP-BINOL-derived product *i*IDP **2a** was previously inaccessible and is now readily available using the new procedure. Furthermore, various *N*-sulfonyl groups can now be easily introduced by simple chloride substitution of intermediate **I-1** with the sulfonamide of choice, as shown with *i*IDPs **2b** and **2c**. The structure of *i*IDP **2d** has been investigated by X-ray crystallography, illustrating the bifunctional active center coordinated to two H_2_O molecules in a structurally confined cavity.

**Scheme 3:**
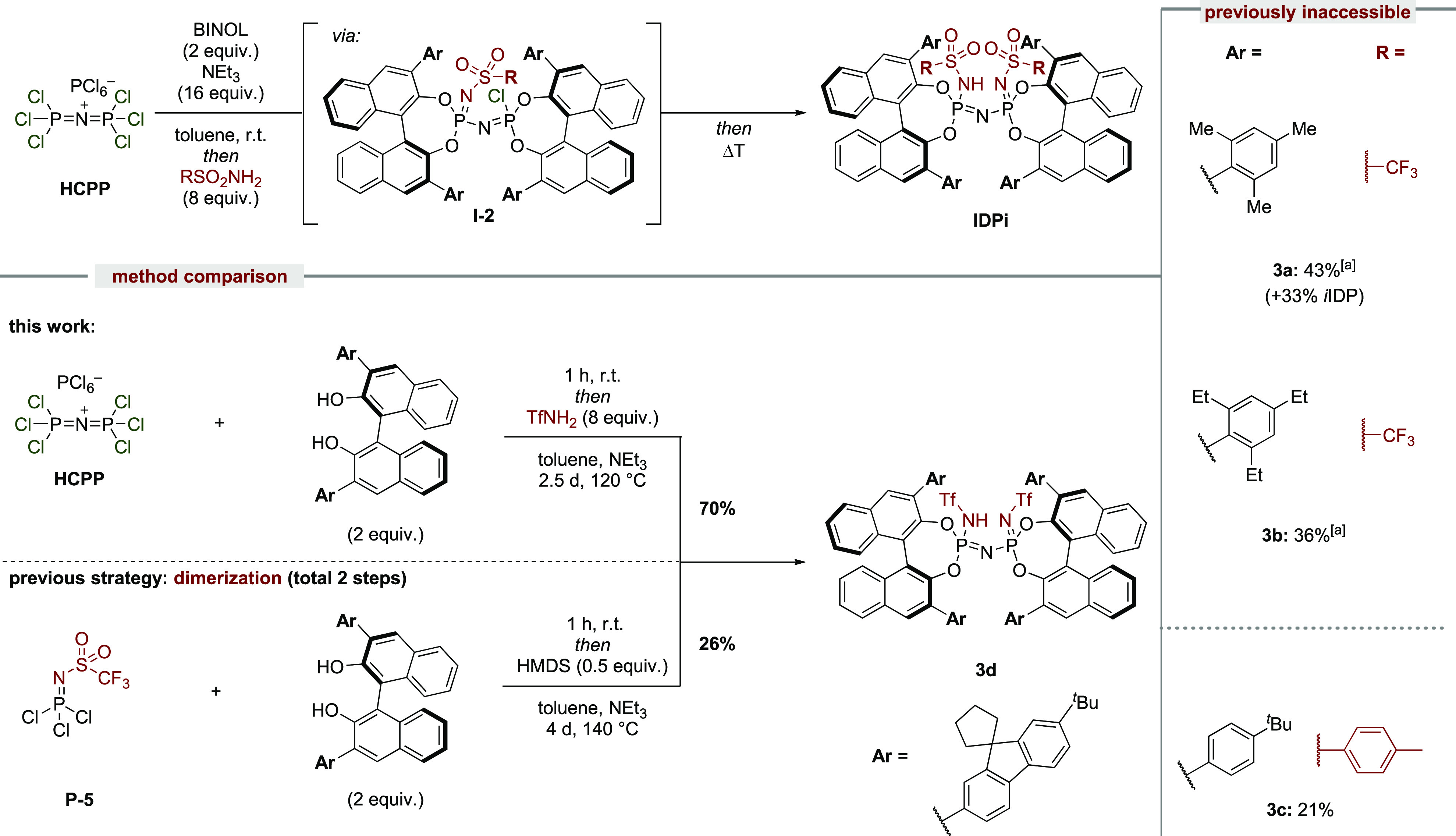
IDPi Synthesis and Reaction Scope 4-DMAP (9 mol %) was added to accelerate the final chloride substitution.

We also investigated the utility of our new approach toward the more acidic IDPi catalyst class (Scheme 3). As hoped, the final chloride substitution of intermediate **I-2** indeed occurs under elevated temperatures and slightly modified reaction conditions (replacing pyridine with NEt_3_ and using toluene as solvent). Highly confined IDPis **3a**,**b**, which were previously elusive following our *in situ* dimerization strategy are now readily accessible, thus expanding the repertoire of novel, structurally confined motifs of this catalyst class. Furthermore, a simple chloride substitution with different sulfonamides, as illustrated with product **3c**, allows a rapid sulfonyl group modification. Following our previous route, access to such IDPi motifs would require a prior synthesis of the corresponding *N*-sulfonylphosphorimidoyl trichloride.^17^ Although the yields were only moderate, unreacted intermediates, such as **I-2**, for the synthesis of IDPi **3a** are isolable by simple flash column chromatography or directly furnish the corresponding *i*IDP upon hydrolysis. We compared the previous dimerization strategy with our new method for the synthesis of IDPi **3d**. Again, the new methodology affords IDPi **3d** in a shorter reaction time and improved yield.

**Scheme 4:**
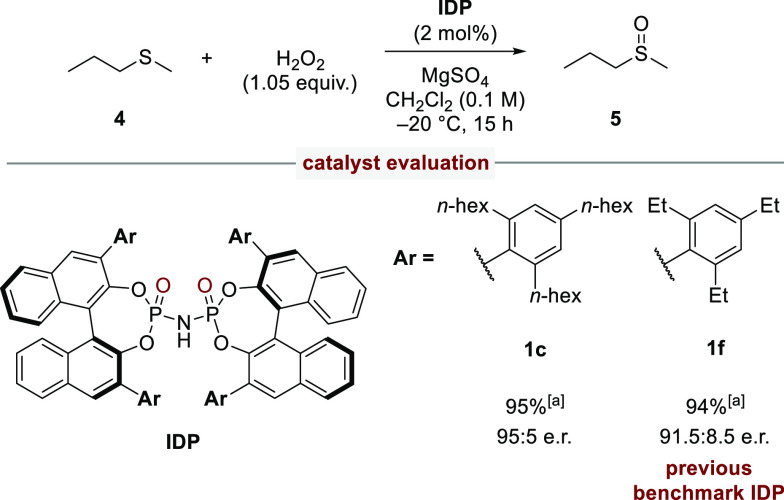
Catalytic Asymmetric Sulfoxidation of Methyl *n*-Propyl Sulfide Yields were determined by ^1^H NMR spectroscopy with dimethyl sulfone as internal standard.

With the established new access to novel and unexplored imidodiphosphates comprising unprecedented structural confinement, we turned our attention to the activation of previously elusive small substrates in asymmetric catalysis. We chose methyl *n*-propyl sulfide **4** as a model substrate for the IDP-catalyzed asymmetric sulfoxidation,^21^ in which our previous benchmark IDP catalyst **1f** furnished an unsatisfactory enantiomeric ratio of 91.5:8.5 of the sulfoxide **5**. Remarkably, IDP **1c** was found to be a superior catalyst for this particularly challenging substrate and delivered the product in 95:5 er (Scheme 4). It should be noted that this is by far the highest enantioselectivity ever obtained with this particular substrate *via* any type of catalytic sulfoxidation.^22^ Such results confirm the importance of having an efficient methodology available to access novel and highly confined catalysts, which are crucial to control structurally unbiased substrates in asymmetric catalysis.

### Toward Superacidity in Asymmetric Counteranion-Directed Catalysis

The formation, stabilization, and utilization of carbocationic intermediates has been extensively studied in academic research and is frequently applied in a technical context, e.g., in Koch–Haaf and Friedel–Crafts reactions.^23^ These transformations usually require strong mineral acids, such as H_2_SO_4_ or TfOH, or strong Lewis acids to dictate the desired carbocation formation upon protonation of olefins or alcohols, whereas the stabilization of carbocationic intermediates by weakly basic counteranions is crucial to prevent undesired side reactions.^24^

A general approach to increase the acidity of Brønsted acids relies on the installment of electron-withdrawing groups into the existing catalyst scaffold.^6b^ Trifluoromethylsulfonyl groups represent one of the strongest and presumably most well-investigated electron-withdrawing group, due to its non-oxidizing properties and inherent stability.^25^ Yagupolskii et al. successfully increased the electron-withdrawing nature of trifluoromethylsulfonyl groups by replacing the corresponding Lewis basic oxygen atoms with additional trifluoromethylsulfonylimino units (Yagupolskii principle).^26^ This acidification effect tremendously increases the acidity of CF_3_SO_3_H (TfOH, p*K*_a_ = −11.4 in DCE) to CF_3_S(NTf)_2_OH (p*K*_a_ = −18 (estimated p*K*_a_ in DCE)).^27^ Analogously, the replacement of Lewis basic =O moieties of aryl sulfonamides with =NTf groups increases the acidity of (4-MeC_6_H_4_)SO_2_NH_2_ (p*K*_a_ = 16.3 in DMSO) toward (4-MeC_6_H_4_)S(NTf)_2_NH_2_ (p*K*_a_ = 3.3 in DMSO), thus enhancing the acidity by 13 p*K*_a_ units and exceeding the electron-withdrawing property of the commonly employed TfNH_2_ group (p*K*_a_ = 9.7 in DMSO) by approximately 6 p*K*_a_ units.^28,29^

**Scheme 5:**
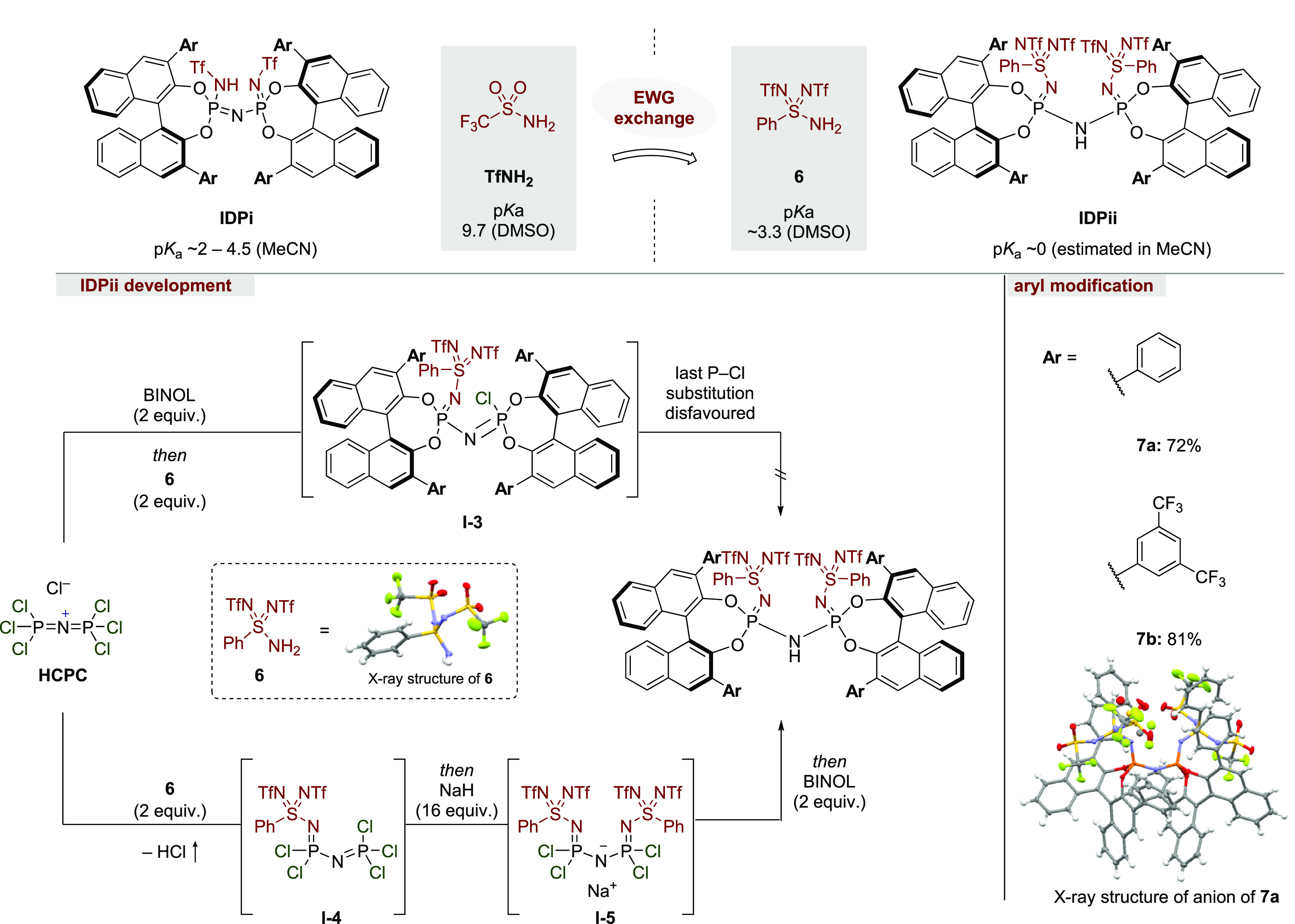
Rational Design and Development of a More Acidic Imidodiphosphazene Catalyst by EWG Replacement

Notably, the utilization of PhS(NTf)_2_NH_2_ (**6**), as the EWG substituent not only enhances the acidity but also simultaneously installs another structural element, in addition to the 3,3′-BINOL substituents, allowing a more flexible and modular implementation of confinement. PhS(NTf)_2_NH_2_ (**6**) was synthesized based on a modified approach reported by Yagupolskii et al. (see Supporting Information for further information) and has been further investigated in this work (Scheme 5).^30^

With the new design and reagent in hand, we evaluated the synthesis of new imidodiphosphorbis(iminosulfonylimino)imidate, IDPii, following the previously described stepwise chloride substitution as shown for the synthesis of IDPis (Scheme 3). Unfortunately, the reaction of sulfonamide **6** with intermediate **I-1** (Scheme 1) proceeded slugglishly and only yielded intermediate **I-3**, which upon hydrolysis afforded the corresponding non-*C*_2_-symmetric iminoimidodiphosphate in poor yields. The desired *C*_2_-symmetric product, bearing two iminosulfonyl units derived from **6**, analogously to IDPis was, unfortunately, not accessible *via* this route. This observation can be explained with the weak nucleophilicity of sulfonamide **6**, hampering the desired chloride substitution of **I-1** and **I-3**.

To overcome the intrinsic barrier of reacting weakly nucleophilic sulfonamide **6** with intermediate **I-3**, showing diminished electrophilic properties, we changed our synthetic strategy. We assumed that the direct reaction of sulfonamide **6** with HCPP, exploiting the immense electrophilic character of this reagent, followed by the BINOL installation event would be more effective. To our delight, we observed the desired transformation of HCPP with sulfonamide **6**, liberating HCl gas to form **I-4**, without the requirement of a base. Unexpectedly, the corresponding PCl_6_^–^ counteranion also reacted with sulfonamide **6** to afford phenylbis(trifluoromethylsulfonylimino)phosphorimidoyl trichloride as undesired side product, which would likely interfere in the BINOL installation step (see Supporting Information for further information). We therefore replaced the PCl_6_^–^ counteranion of HCPP with a chloride counteranion, following Manners’ one-step procedure, to afford hexachlorobisphosphazonium chloride (HCPC).^20a^

As expected, HCPC reacted smoothly with sulfonamide **6** to quantitatively form **I-4** at room temperature within 30 min, presenting an ideal intermediate for our desired catalyst motifs. The reaction of **I-4** with another equivalent of sulfonamide **6** in the presence of sodium hydride or an organic base such as triethylamine afforded sodium bis(trifluoromethylsulfonylimino)tetrachloridophosphazenate **I-5**, in which both sulfonamides are installed into the imidodiphosphate scaffold. We found suitable reaction conditions, in which intermediates **I-4** and **I-5** are formed *in situ* and reacted upon addition of BINOL toward the desired catalyst motif IDPii, in a single-flask procedure, providing **7a** and **7b** in good yields. These catalysts were rapidly acidified, either by dissolving the corresponding salts in dichloromethane and emulsifying with aqueous HCl or by passing a catalyst solution through Dowex 50W-X8. It should be noted that the dimerization strategy for phenylbis(trifluoromethylsulfonylimino)phosphorimidoyl trichloride with BINOL and hexamethyldisilazane (HMDS) or ammonia yielded the desired dimer **7a** in traces (6% on a large scale), whereas the formation of **7b** was not observable. This result underlines the applicability of hexachlorobisphosphazonium salt as a building block to rationally design and successfully enhance the repertoire of imidodiphosphoryl scaffolds, which might turn out to be superior catalysts or interesting ligands for transition-metal catalysis. IDPii **7a** was further characterized by X-ray crystallography.

**Scheme 6:**
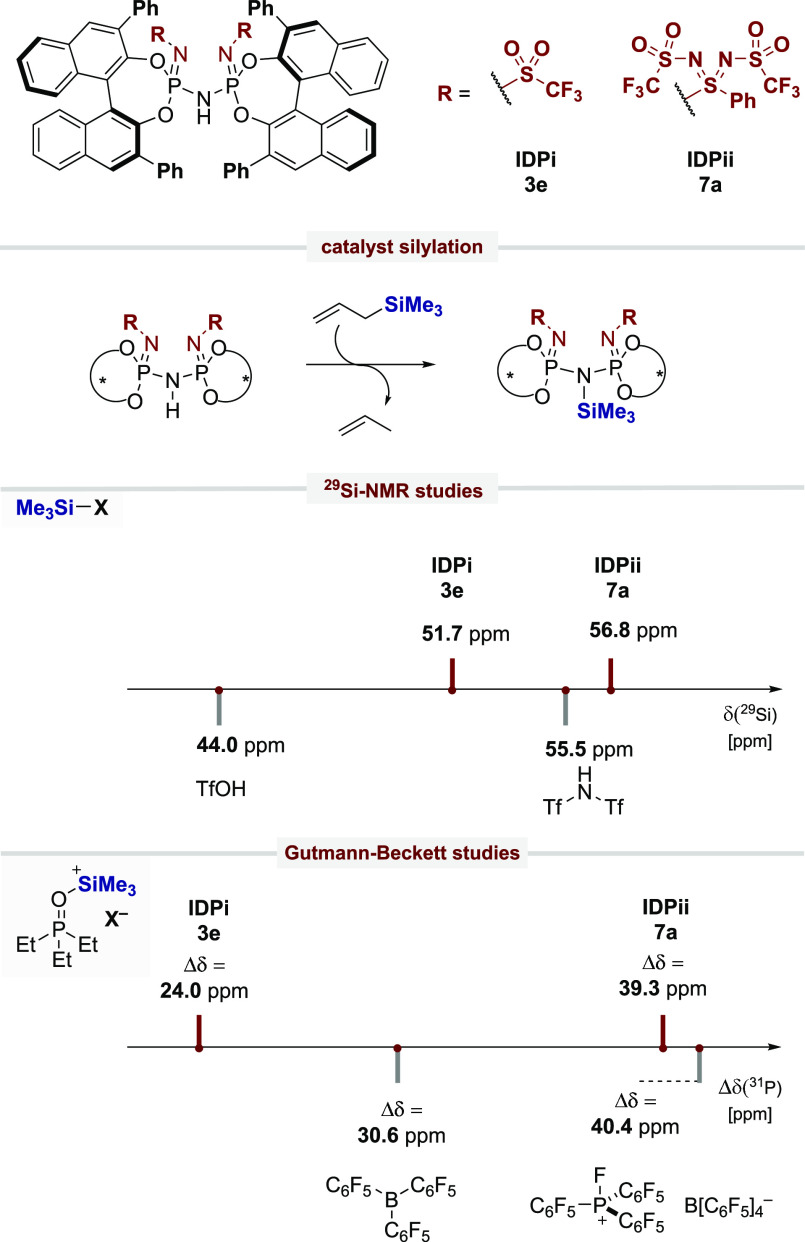
^29^Si NMR and Gutmann–Beckett studies to quantify Lewis acidities

With these novel catalysts in hand, we aimed toward a reactivity comparison of IDPi and IDPii, applying the same phenyl-derived BINOL substituents to evaluate the acidifying effect of our new core modification (Scheme 6). In light of recent ^29^Si NMR studies from Oestreich and our group in combination with Gutmann–Beckett studies,^31^ we focused on the quantification of Lewis acidities of IDPi **3e** and IDPii **7a**, which rapidly react with allyltrimethylsilane to furnish the corresponding Lewis acidic silylated imidodiphosphazene catalysts.^32^ It should be noted, that IDP and *i*IDP were not included in our studies due to inefficient catalytic activity as Lewis acids. As expected, our new catalyst motif IDPii **7a** shows a much higher ^29^Si chemical shift, in direct comparison to that of IDPi **3e**, suggesting a significantly enhanced Lewis acidity.^24,33^ Interestingly, IDPii **7a** exceeds the chemical shift of trimethylsilyl triflate (TMSOTf) and bis(trifluoromethylsulfonyl)imide (TMSNTf_2_), which are commonly employed superacids in organic synthesis. In agreement with our experience of IDPi catalysis, trimethylsilylated IDPi **3e** represents a stronger Lewis acid in comparison to TMSOTf but remains a significantly weaker Lewis acid than TMSNTf_2_. The same reactivity trend has been observed in our Gutmann–Beckett study, in which IDPii **7a** resulted in a triethylphosphine oxide shift of Δδ = 39.3 ppm, whereas the utilization of IDPi **3e** leads to a shift of Δδ = 24.0 ppm, supporting our hypothesis of an increased Lewis acidity of IDPii to the analogous IDPi (see Supporting Information).

**Scheme 7:**
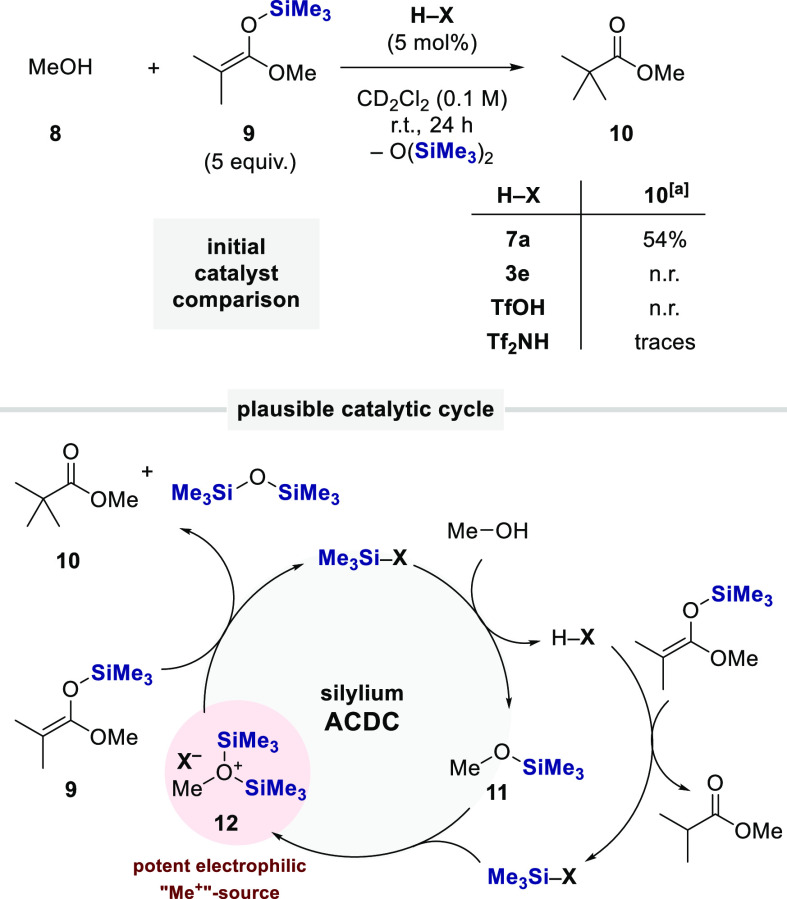
Initial Catalyst Screening for the α-Methylation of a Silyl Ketene Acetal with Methanol Yields were determined by ^1^H NMR spectroscopy with mesitylene as internal standard.

Interestingly, our Gutmann–Beckett study indicates a Lewis acidity of IDPii **7a** that is similar to the extremely Lewis acidic fluorophosphonium tetrakis(pentafluorophenyl)borate salt, initially reported by Stephan et al.,^34^ which has been utilized for various challenging transformations proceeding *via* the formation of carbocationic intermediates.^35^ However, these extremely Lewis acidic catalysts often require strictly inert reaction conditions to prevent catalyst degradation, especially due to hydrolysis pathways in the presence of nucleophilic and protic impurities, such as water or alcohols. In contrast, our catalyst motifs possess the advantage of extreme Lewis acidity, without the requirement of inert reaction conditions, due to the catalytic deprotosilylation cycle, in the presence of sacrificial silylating reagent. This property led to the hypothesis that we might be able to convert methanol—a normally incompatible *nucleophile* for many strong Lewis acids and transition-metal catalysts—into a potent *electrophile*. We reasoned that methanol (**8**) should first undergo a deprotosilylation cycle in the presence of a silylating agent, such as trimethylsilyl ketene acetal **9**, to afford trimethylsilyl methyl ether **11***in situ*, which in return should still be Lewis basic enough to react with another equivalent of trimethylsilylated IDPii to form the corresponding bis(trimethylsilyl)methoxonium salt **12**. Analogously to Meerwein salts, ion pair **12** was envisioned to represent a powerful methylating agent, which should readily react with the nucleophilic silyl ketene acetal **9** to furnish methyl pivalate **10** as the final product (Scheme 7).

Remarkably, a comparison between TfOH, Tf_2_NH, and our IDPi and IDPii catalysts revealed that the desired transformation only proceeded with our new IDPii catalyst class, whereas the other three catalysts did not engage in the desired transformation. Their insufficient reactivity most likely results from the weaker Lewis acidity of these catalysts, consistent with our Lewis acidity measurements. Our newly designed transformation from the *in situ* generation of the highly potent and electrophilic methylating reagent **12** avoids the utilization of commonly employed toxic alkylating reagents such as dimethyl sulfate or methyl iodide. The asymmetric α-alkylation of silyl ketene acetals and the expansion toward various silyl-derived nucleophiles to develop a general asymmetric dehydroxyfunctionalization strategy is currently in investigation in our laboratory and will be communicated independently.

## Conclusion

The imidodiphosphoryl scaffold represents a highly versatile platform to design Brønsted acids, merging enzyme-like substrate recognition with modular acidities that enable several, perhaps surprising and unique, reactions in asymmetric catalysis over the past years. We have revealed a new user-friendly synthesis of imidodiphosphoryl-based catalysts, in which a hexachlorobisphosphazonium salt serves as a building block and selectively reacts with chosen nucleophiles based on a toolbox principle. This methodology, which proceeds *via* common key intermediates, provides a fast and highly efficient access to privileged Brønsted acids, such as IDP, *i*IDP, and IDPi, comprising unique and, most notably, previously inaccessible confinement. In fact, these catalysts were conceptually designed to provide superior enantiocontrol of small and structurally unbiased substrates, as illustrated in the first highly enantioselective sulfoxidation of methyl propyl sulfide.

Furthermore, this novel modular assembly synthesis allows the implementation of new strongly acidifying sulfonamides into the imidodiphosphoryl scaffold, empowering the conceptualization and development of an extremely reactive Brønsted acid, IDPii. Analytical and experimental studies show, under silylium Lewis acid conditions, a significantly enhanced catalytic performance for the IDPii, which overcomes the reactivity of commonly employed super-Brønsted acids, such as TfOH and Tf_2_NH. The superior reactivity enables the realization of a new α-alkylation of a silyl ketene acetal—utilizing methanol as electrophilic methyl surrogate—and expands the repertoire of useful α-alkylation strategies of carbonyl compounds. We anticipate that our methodology provides a new foundation toward future developments of novel imidodiphosphoryl-type catalysts, leading to efficient asymmetric transformation in the field of asymmetric organocatalysis or as ligands in transition-metal catalysis.

## Supporting Information Available

Experimental procedures, additional information, and analytical data for all new compounds (PDF)

## Accession Codes

CCDC 2043273 and 2091821–2091822 contain the supplementary crystallographic data for this paper. These data can be obtained free of charge via www.ccdc.cam.ac.uk/data_request/cif, or by emailing data_request@ccdc.cam.ac.uk, or by contacting The Cambridge Crystallographic Data Centre, 12 Union Road, Cambridge CB2 1EZ, UK; fax: +44 1223 336033.
