# Supplementary material for: Unified Approach to Imidodiphosphate-Type Brønsted Acids with Tunable Confinement and Acidity
Source: J Am Chem Soc. 2021 Sep 3;143(36):14835–44. doi: 10.1021/jacs.1c07067 (PMC8447263; doi:10.1021/jacs.1c07067)
Supplement: Supplementary file 1 — ja1c07067_si_001.pdf [file ja1c07067_si_001.pdf]

Supplementary Information

A Unified Approach to Imidodiphosphate-Type Brønsted Acids with Tunable Confinement and Acidity

Sebastian A. Schwengers, Chandra Kanta De, Oleg Grossmann, Joyce A. A. Grimm, Natascha R. Sadlowski, Gabriela G. Gerosa, and Benjamin List*

Max-Planck-Institut für Kohlenforschung, Kaiser-Wilhelm-Platz 1, 45470 Mülheim an der Ruhr, Germany

Table of contents

1. General Information S3

1.1 General reaction setup S3

2 Catalytic experiments S4

2.1 Asymmetric oxidation of methyl *n*-propyl sulfide S4

2.2 α-Alkylation of methyl trimethylsilyl dimethylketene acetal S5

2.3 Preliminary studies toward the asymmetric α-methylation S17

3. Synthesis of HCPP S18

3.1 One-step synthesis of hexachlorobisphosphazonium hexachlorophosphate (HCPP) S18

3.2 Synthesis of hexachlorobisphosphazonium chloride (HCPC) S18

3.3 Alternative two-step synthesis of HCPP S19

3.3.1 Synthesis of (trimethylsilyl)phosphorimidoyl trichloride S19

3.3.2 Synthesis of hexachlorobisphosphazonium hexachlorophosphate (HCPP) S19

4. Synthesis of BINOL S20

4.1 *(S)*-3,3´-bis(2,4,6-tri-n-hexylphenyl)-BINOL S20

4.2 *(S)*-3,3´-bis(2,4,6-tri-n-pentylphenyl)-BINOL S21

4.3 *(S)*-3,3´-bis(2,4,6-tri-n-heptylphenyl)-BINOL S21

4.4 *(S)*-3,3´-bis(4-((trifluoromethyl)thio)phenyl)-BINOL S22

5. Synthesis of imidodiphosphates (IDP) S23

5.0 Initial NMR experiments S23

5.1 IDP-1a S24

5.2 IDP-1b S25

5.3 IDP-1c S26

5.4 IDP-1d S27

5.5 IDP-1e S28

6. Synthesis of iminoimidodiphosphate (iIDP) S29

6.0 Initial NMR experiments S29

6.1 iIDP-2a S30

6.2 iIDP-2b S31

6.3 iIDP-2c S32

6.4 iIDP-2d S33

6.5 iIDP-2e S34

7. Synthesis of Imidodiphosphorimidates (IDPi) S35

7.0 Initial NMR experiments S35

7.1. IDPi-3a S36

7.1.1 Intermediate I-2 .... S37

7.2 IDPi-3b S38

7.3 IDPi-3c S39

7.4 IDPi-3d S40

8. Synthesis of phenylbis(trifluoromethylsulfonylimino)sulfonamide 6 .... S41

8.1 Sodium phenylbis(trifluoromethylsulfonylimino)sulfinate 5 .... S41

8.2 Phenylbis(trifluoromethylsulfonylimino)sulfonamide 6 .... S42

9 Synthesis of Imidodiphosphorsulfonyliminoimidates (IDPii) S43

9.0 General remarks.......... S43
9.1 Synthesis of IDPii 9a S45
9.2 Synthesis of IDPii 9b S46
10 NMR-spectra S47
10.1 ^{29}Si-NMR studies.......... S85
10.2 Gutmann-Beckett Studies.......... S88
11 GC-traces.......... S89
12.1 Single crystal structure analysis of [2d^{-} H_3O^{+}], 1·H_2O 2· CH_2Cl_2.......... S94
12.2 Single crystal structure analysis of 6.......... S105
12.3 Single crystal structure analysis of IDPii 7a.......... S110

1. General Information

Unless otherwise stated, all reagents were purchased from commercial suppliers and used without further purification. Reactions were monitored by thin layer chromatography (TLC) on silica gel pre-coated plastic sheets (0.2 mm, Macherey-Nagel) or glass plates (SIL G-25 UV_{254}, 0.25 mm, Macherey-Nagel). Visualization was accomplished by irradiation with UV light at 254 nm and/or phosphomolybdic acid (PMA) stain. PMA stain: PMA (10 g) in EtOH (100 ml). Flash column chromatography (FCC) was performed on Merck silica gel (60, particle size 0.040–0.063 mm) or on an automated flash purification system: BIOTAGE Isolera^{TM} Four with pre-packed Sfär Silica HC D columns (10 and 25 g). The solvent mixtures used as eluent refer to percentage by volume (vol%). NMR spectra were recorded on a Bruker AV-500 or Bruker AV-600 spectrometer in deuterated solvents. Proton chemical shifts are reported in ppm (δ) relative to tetramethylsilane (TMS) with the solvent resonance employed as the internal standard ($CDCl_3$ δ=7.26 ppm; CD_2Cl_2 δ=5.32 ppm). NMR solvents were dried and stored over pre-activated 4 Å molecular sieves. Data are reported as follows: chemical shift, multiplicity (s = singlet, d = doublet, t = triplet, q = quartet, p = pentet, sext = sextet, h = heptet, m = multiplet, br = broad), coupling constants (Hz) and integration. ^{13}C chemical shifts are reported in ppm from tetramethylsilane (TMS) with the solvent resonance as the internal standard ($CDCl_3$ δ=77.16 ppm; CD_2Cl_2 δ=53.84 ppm). ^{19}F, ^{31}P NMR spectra were referenced in ppm from CCl_3F and H_3PO_4, respectively. High resolution mass spectra were determined on a Bruker APEX III FTMS (7 T magnet). The title compounds are named based on a suggestion of ChemDraw Professional (15.0; Cambridgesoft).

1.1 General reaction setup

Unless otherwise stated, all reactions were performed under argon using common Schlenk techniques. Reaction vessels were flame-dried under high vacuum (h.v.), purged with argon and cooled to room temperature (r.t.). **HCPP** was stored in a Schlenk flask under argon at r.t.. HCPP was transferred to a tared flame-dried and argonated (1 atm) Schlenk flask and weighted again to determine the amount of **HCPP** transferred to the reaction vessel. All solvents were dried using common drying procedures and stored over pre-activated 4 Å molecular sieves. Acidifications of catalysts in salt form were carried out by dissolving the catalyst (salt form) in CH_2Cl_2 with an equimolar amount of HCl (6 M) and stirring the corresponding emulsion for 30 minutes at r.t.. The lower organic phase (CH_2Cl_2) needs to be separated and concentrated to dryness to furnish the desired catalyst in acidic form. An alternative acidification procedure relies on a chromatographic acidification, in which the catalyst in salt form was dissolved in a small quantity of CH_2Cl_2 and passed through a plug of pre-acidified DOWEX® 50WX8 (50-100 mesh). DOWEX® 50WX8 was purchased from Sigma-Aldrich and was acidified by washing the resin with aqueous H_2SO_4 (0.125 N) until the elute remains colorless, followed by subsequent washing with methanol, Et_2O and CH_2Cl_2.

2 Catalytic experiments

2.1 Asymmetric oxidation of methyl *n*-propyl sulfide

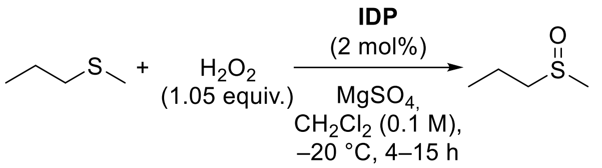

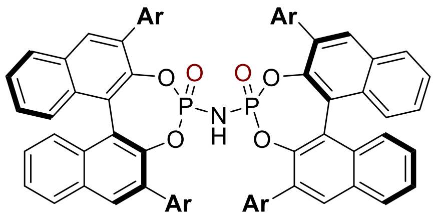

IDP

temp. [°C]	solvent	catalyst	e.r.	yield[a]
r.t.	CyH	**1a**	51:49	29%
		**1b**	91.5:8.5	86%
		**1c**	93.5:6.5	89%
		**1d**	92.5:7.5	88%
		**1e**	90:10	87%
r.t.	CCl_4	**1a**	50:50	36
		**1b**	93:7	99
		**1c**	93:7	99
		**1d**	92.5:7.5	99
		**1e**	92:8	99
0	CCl_4	**1b**	92.5:7.5	83
	CH_2Cl_2	**1b**	94:6	81
–20	CH_2Cl_2	**1a**	50:50	25
		**1b**	95:5	95
		**1c**	95:5	95
		**1d**	95:5	98
		**1e**	91.5:8.5	94

[a] = NMR yields. Yields were determined by ^{1}H NMR using dimethylsulfone as internal standard.

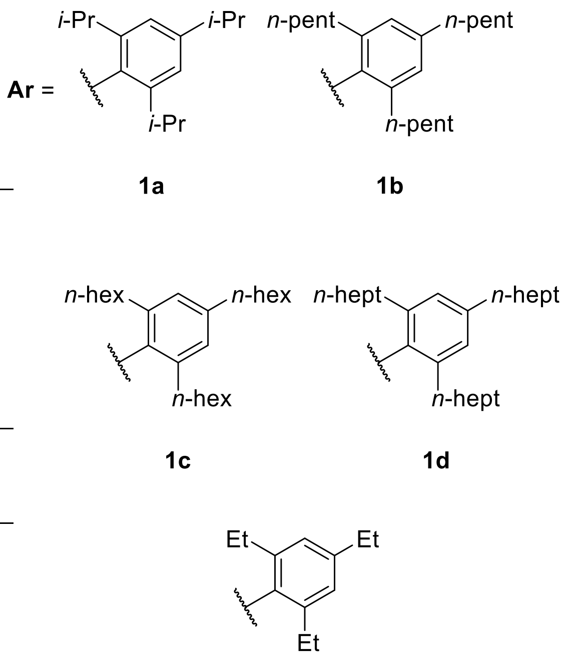

1a **1b** **1c** **1d**

1e
previous
benchmark IDP

Standard procedure: A GC Vial was charged, under aerobic conditions, with the corresponding catalyst (1 µmol, 2 mol%) and anhydrous magnesium sulfate (22.0 mg; 185 µmol). A freshly prepared stock solution of 0.1 M methyl *n*-propyl sulfide in CD_2Cl_2 (500 µl, 50.0 µmol, 1.00 equiv) was added to each GC-vial with the appropriate catalysts, cooled in dry ice (or left at r.t. if the reaction was conducted at r.t.) followed by the addition of hydrogen peroxide (35%, 5 µl, 52.5 µmol, 1.05 equiv.) and stirred at the mentioned temperature in a pre-cooled cryostat for 4 (for CyH and CCl_4 at r.t.) or for 15 h (CCl_4 and CD_2Cl_2 at 0/–20 °C) respectively. A freshly prepared stock solution of the internal standard in CD_2Cl_2 was added to every reaction mixture and filtered through a plug of sodium sulfate. Yields and enantiomeric ratios were determined by NMR spectroscopy and gaschromatography, respectively, without additional purification procedures. The product of the reaction with IDP-1c was isolated to determine the optical rotation: $[\alpha]_D^{25}$ = –72 (c = 1 mg/ml in CH_2Cl_2)

2.2 α-Alkylation of methyl trimethylsilyl dimethylketene acetal

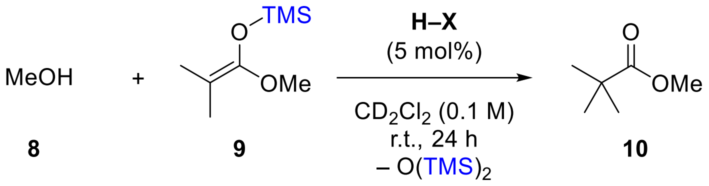

8 9 10

H–X	12
**7a**	54%
**3e**	n.r.
**TfOH**	n.r.
**Tf_2NH**	traces

Procedure: Four NMR tubes were charged with the corresponding catalyst **7a**, **3e**, **TfOH** or **Tf_2NH** (5 mol%). A CD_2Cl_2 stock solution was prepared, containing mesitylene as internal standard (34.8 mg, 290 µmol), methanol (10 µL, 250 µmol) trimethylsilyl dimethylketene acetal (238 mg, 1.37 mmol) in 2.5 ml DCM-d2. 500 µl of this stock solution was transferred to each NMR tube, the tubes sealed and the reaction progress monitored over time by NMR.

^{1}H-NMR-spectra after 24 h reaction time of each catalyst:

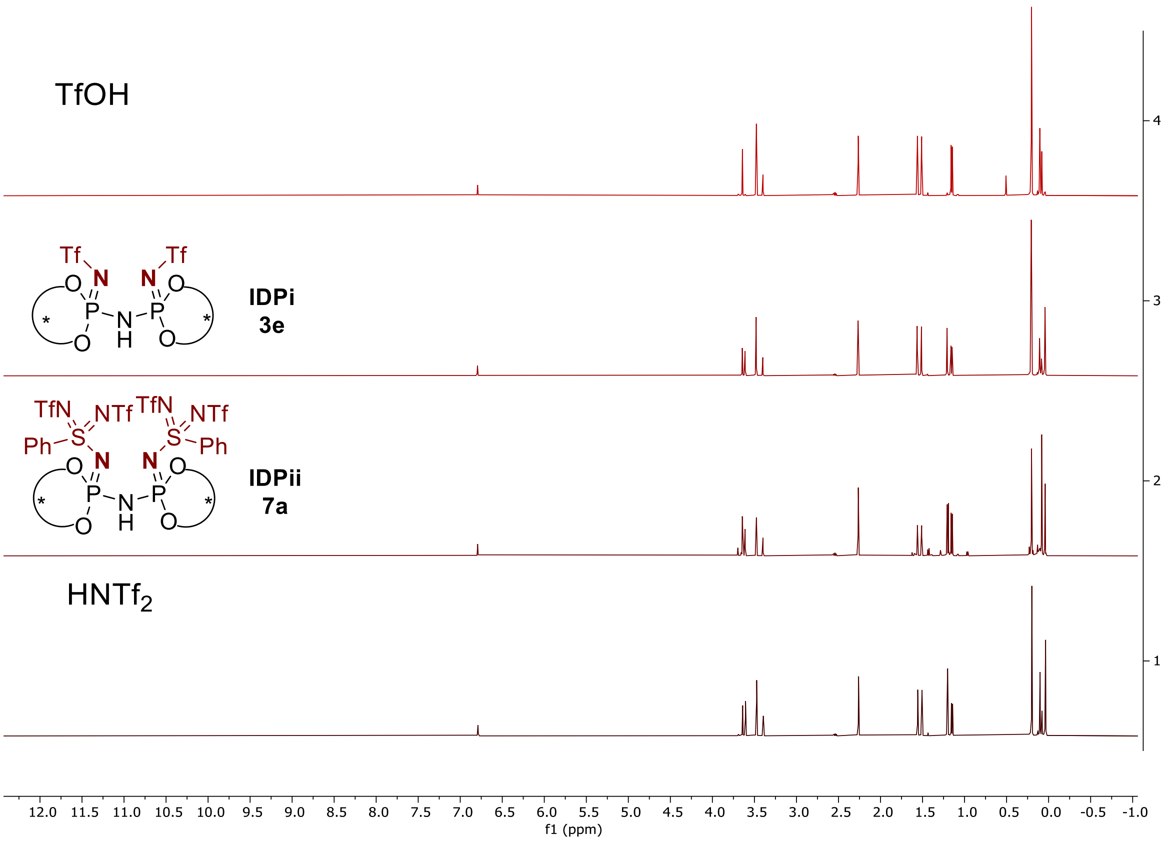

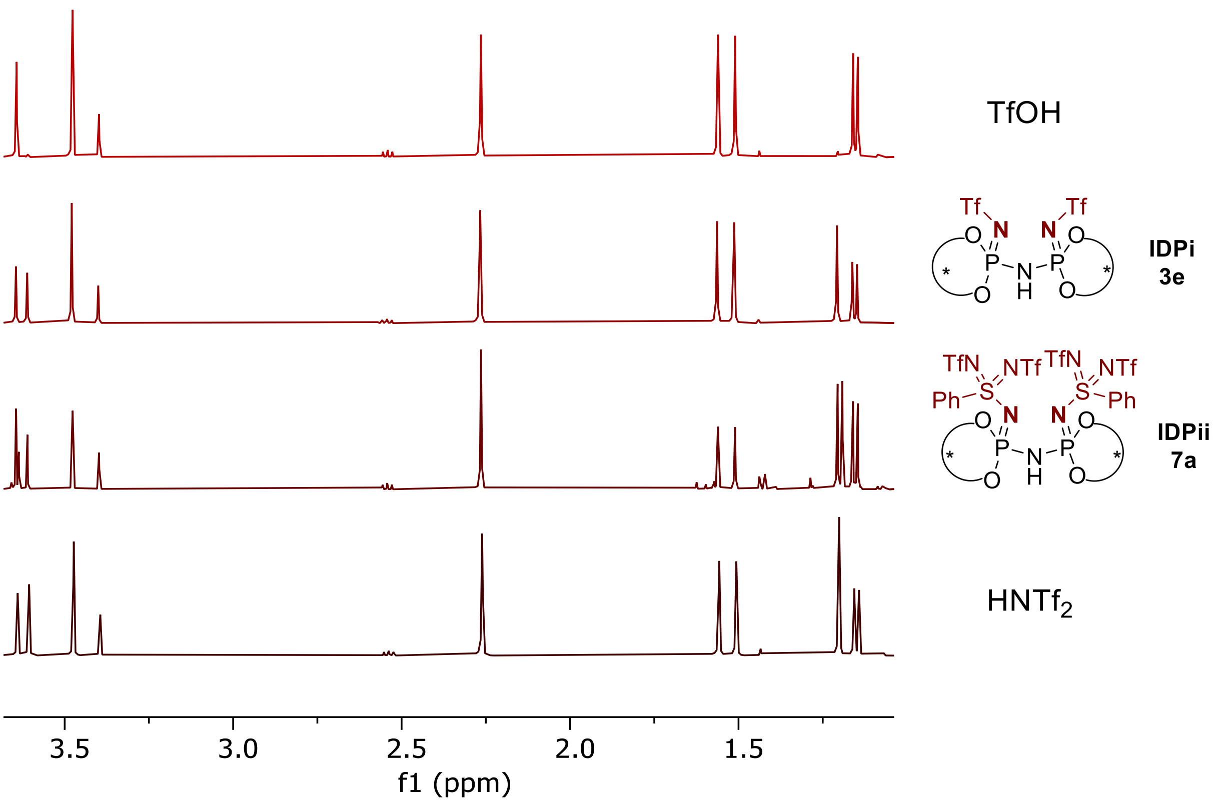

NMR spectra with catalyst **IDPii 7a** followed over time. Product signals are indicated with a black arrow.

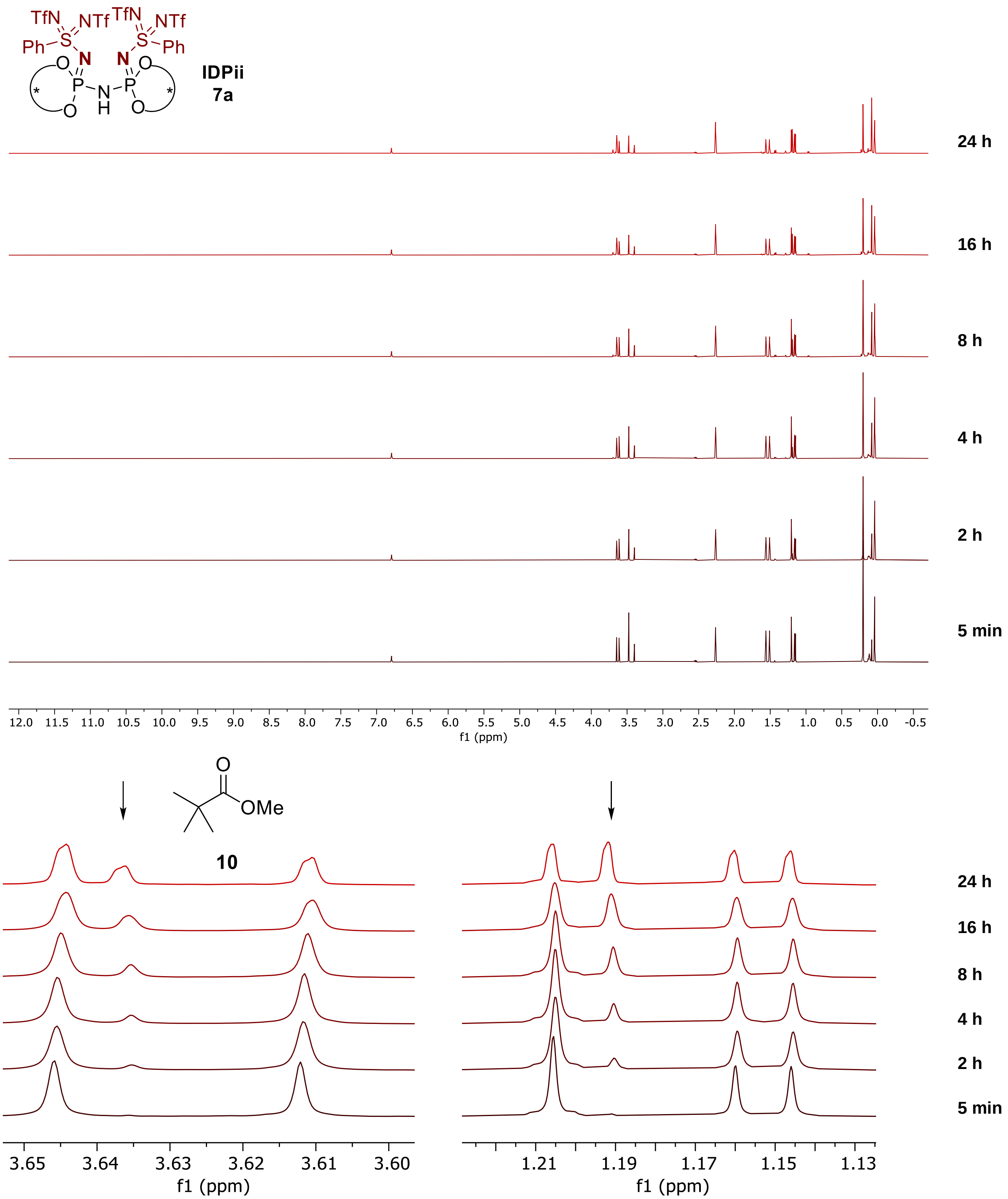

The formation of the desired product was confirmed by HRMS (ESI): m/z calcd for $C_6H_{13}O_2$: 117.091005 $[M+H]^+$, found 117.091010

In collaboration with Dr. Markus Leutzsch from our NMR-department, additional NMR experiments were performed in a thick-wall 3mm J-Young NMR tube and analyzed over a certain time period. The sample was prepared under standard conditions as described above and cooled to –78°C. Prior to the NMR measurements the sample was shaken and quickly transferred to the NMR magnet, where the first ^{1}H NMR spectrum was acquired with a single scan after approx. 3 min. The raw NMR data was imported into MNOVA 14.1.2 with the reaction monitoring plugin and processed therein. The first ^{1}H NMR was used as concentration reference for all the others ^{1}H NMRs taken.

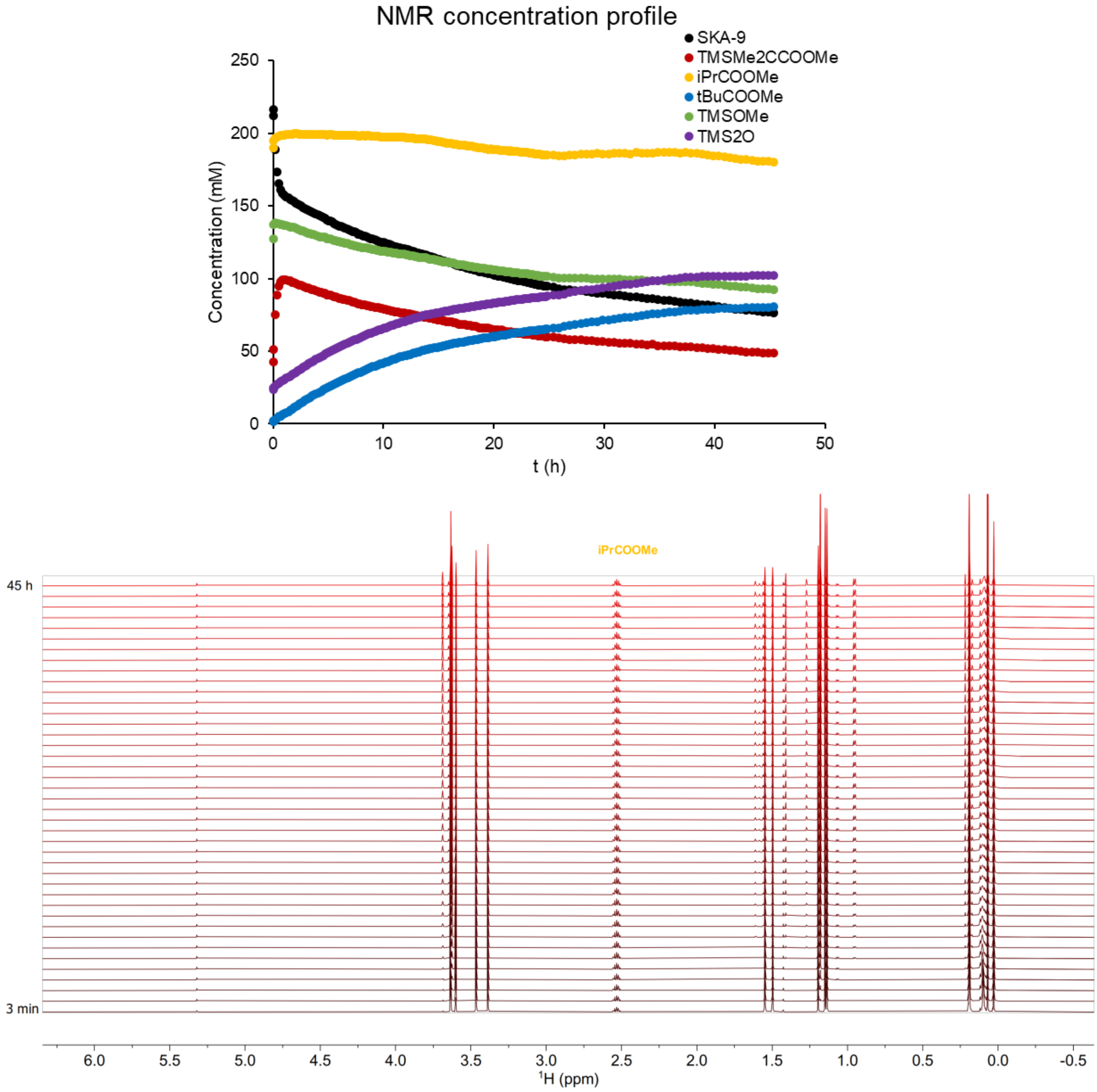

Figure 1: ^{1}H NMR spectra taken at different time points during the reaction.

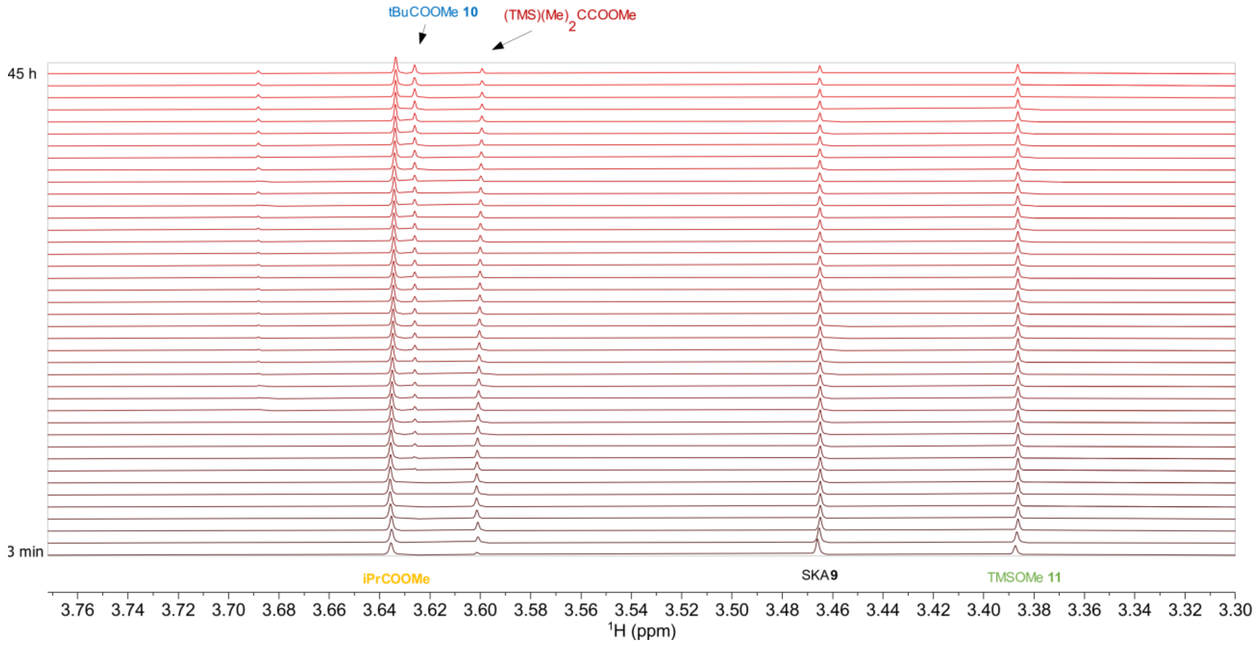

Figure 2: OMe-region of the ^{1}H NMR spectrum at different time points during the reaction.

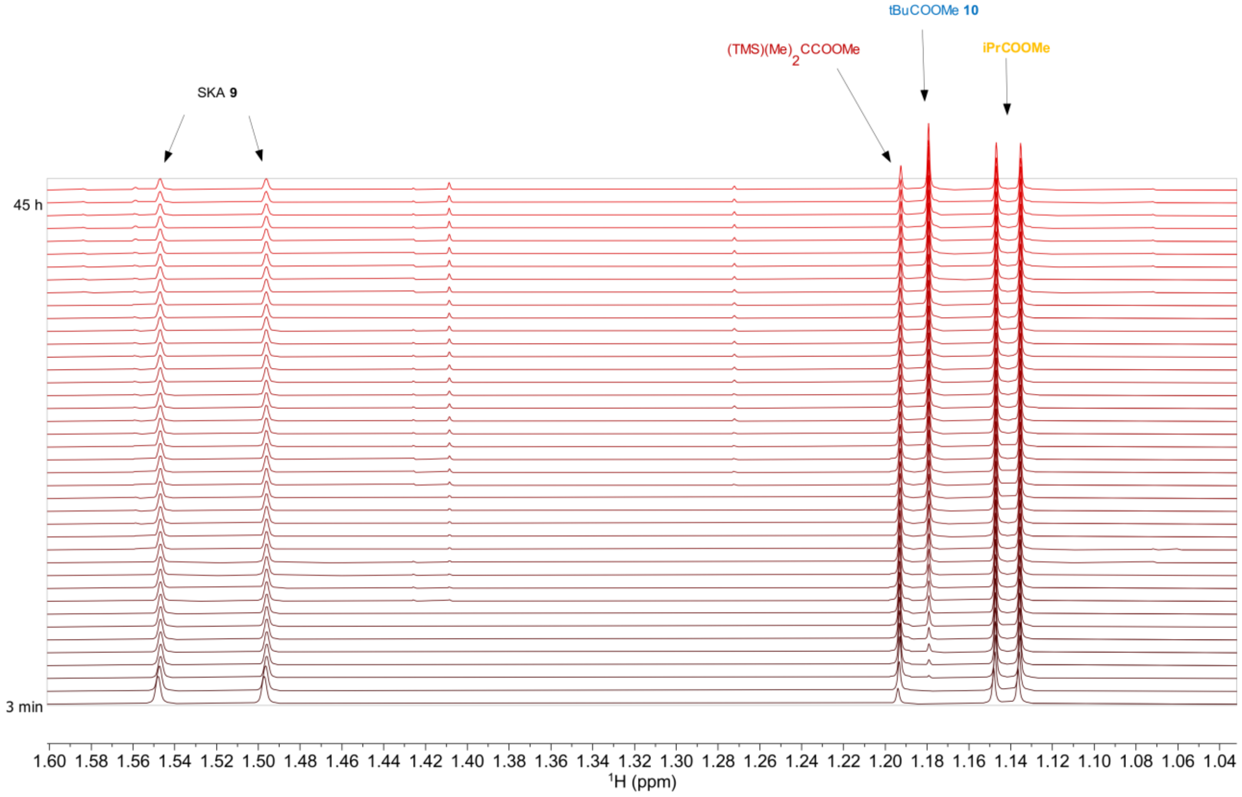

Figure 3: aliphatic Me-group region of the ^{1}H NMR spectrum at different time points during the reaction.

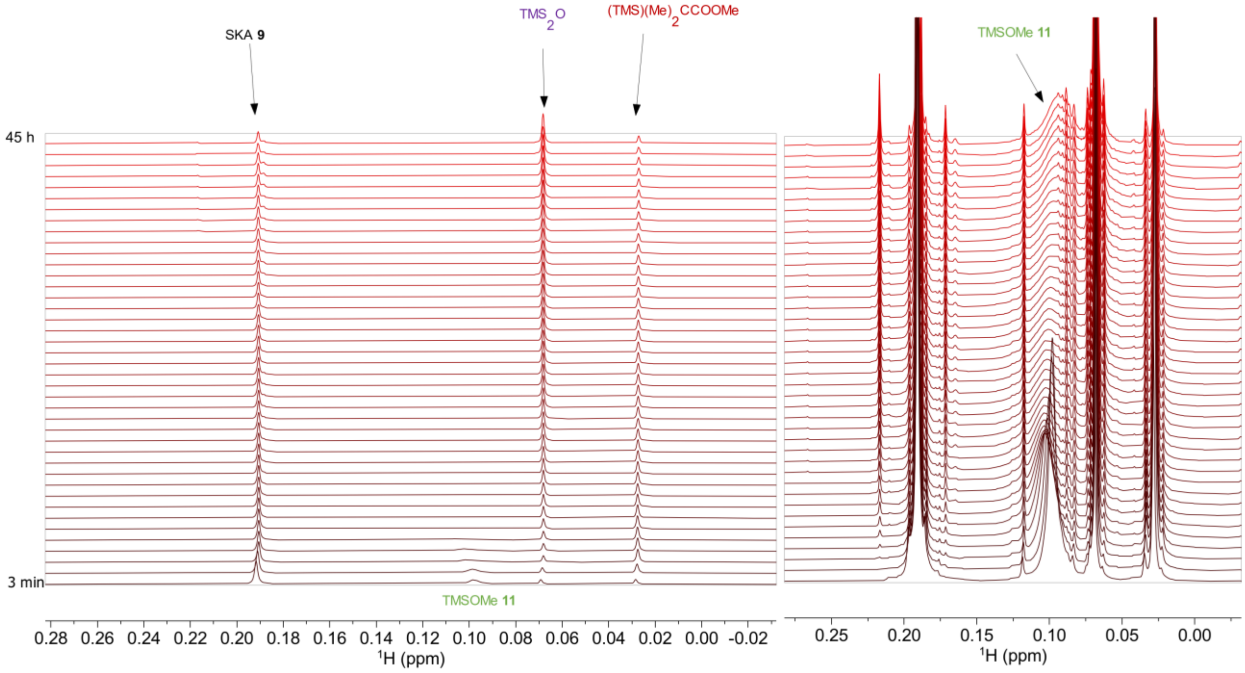

Figure 4: TMS region of the ^{1}H NMR spectrum at different time points during the reaction. The right stacked plot shows an increased intensity level to show the broadening of the TMSOMe signal at later time points.

Mechanistic aspects

To investigate the origin of the methyl group in the product **10** the reaction was studied with CD_3OD as electrophilic methyl surrogate.

The following figure shows ^1H and ^2H NMR spectra at the beginning of the reaction with a comparison to the non-deuterated reaction. The methyl signal of TMSOMe is missing in the ^1H NMR. In the ^2H NMR spectrum, the O-Me signal is observed as well as the signal of $Me_2CDCOOOMe$ originating from the TMSOMe formation process.

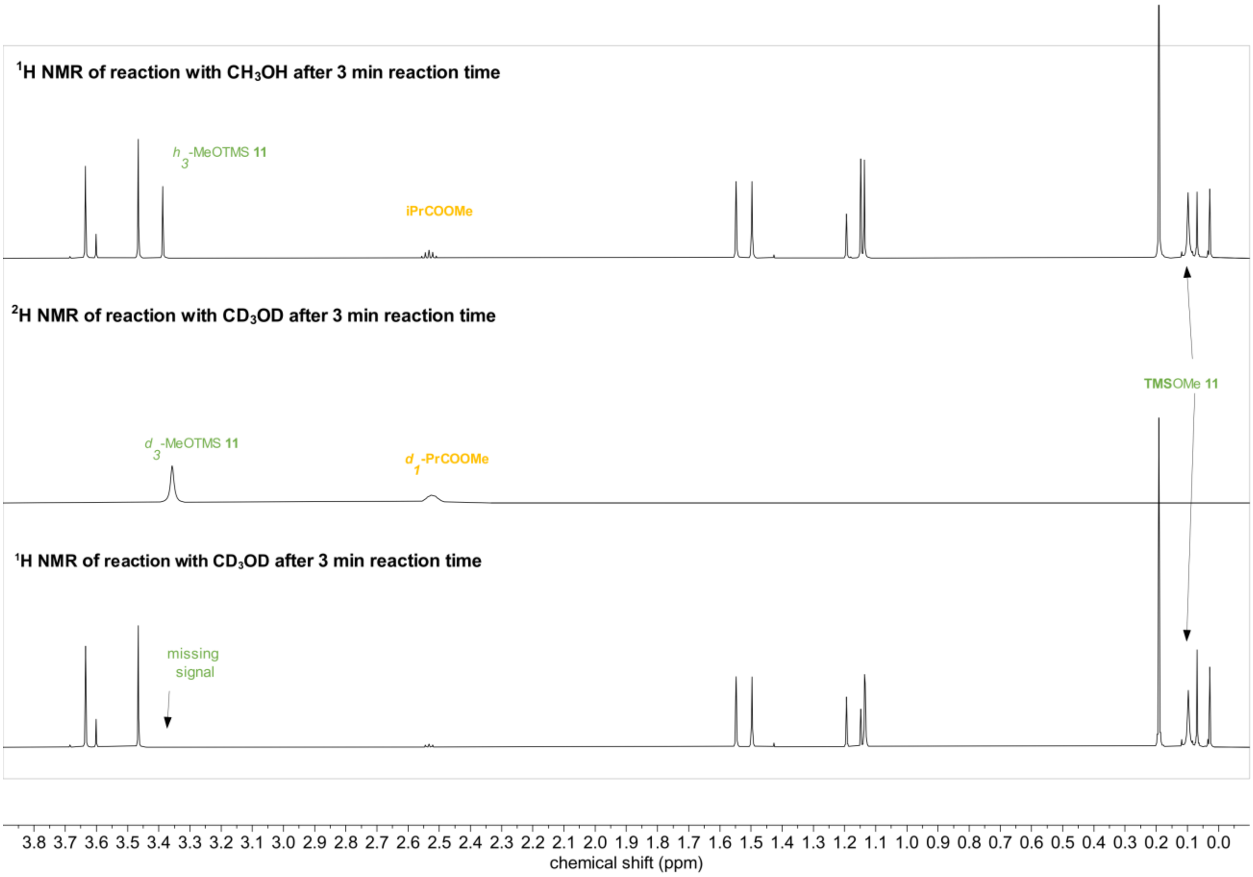

Figure 5: ^1H and ^2H NMR spectra of the reaction in presence of CD_3OD in comparison to the reaction with CH_3OH at the beginning of the reaction.

After 22h of reaction time the ^{2}H NMR spectrum has visibly changed. The formation of a signal of an aliphatic signal at 1.15 ppm is observed which can clearly be attributed to the product d_3-**10**. In the ^{1}H NMR the signal of the deuterated product is observed at 1.174 ppm. This is slightly shifted due the isotope effect on the chemical shielding of the d_3-Me group. Additionally, the fully protonated product is also observed at 1.179 ppm. This indicates that bis(trimethylsilyl)methoxonium ion is able to catalyze a transesterification reaction. The presence of small amount of h_3-MeOTMS is also visible at this point of the reaction further supporting this. However, the outcome of this experiment clearly shows, the methanol added to the reaction is the major methyl source for the α-alkylation.

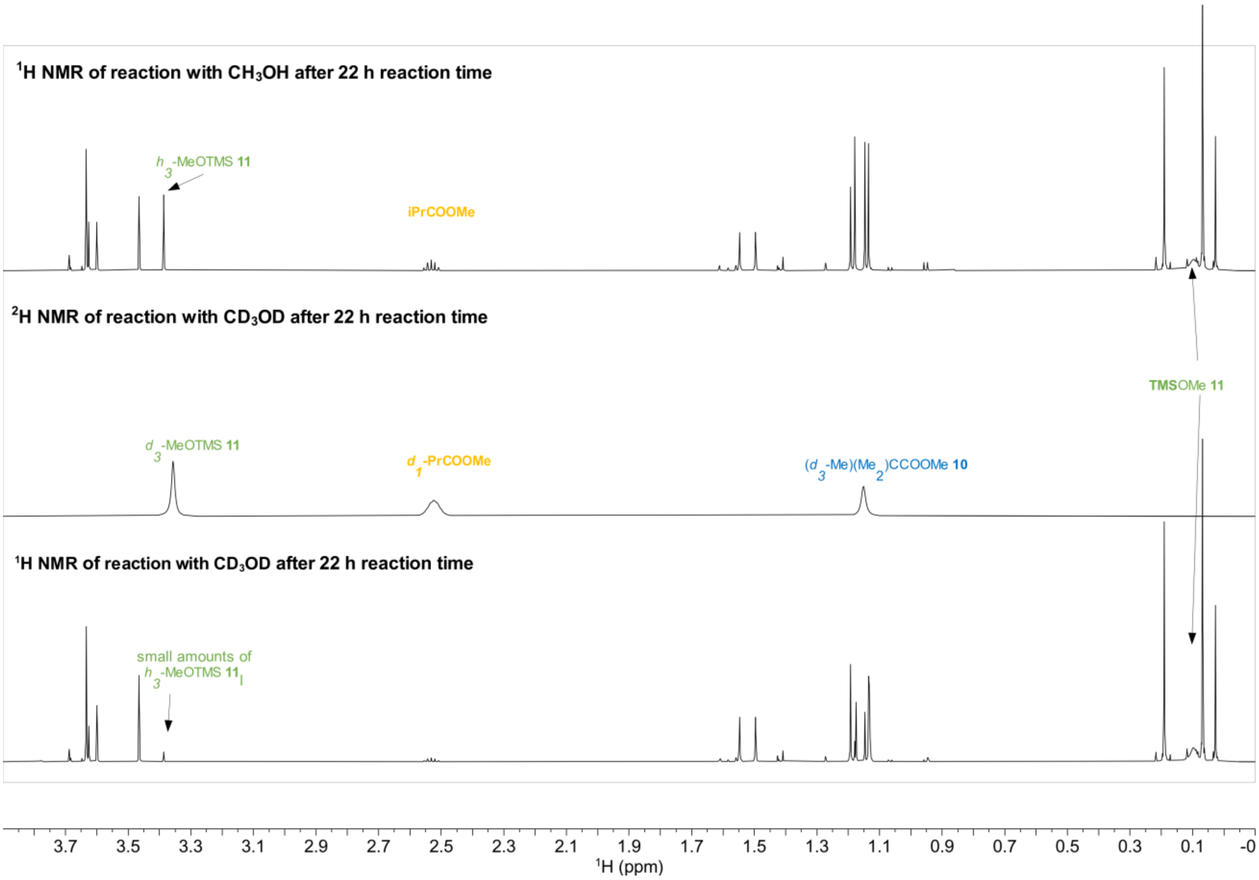

Figure 6: ^{1}H and ^{2}H NMR spectra of the reaction in presence of CD_3OD in comparison to the reaction with CH_3OH after 22 h of reaction time.

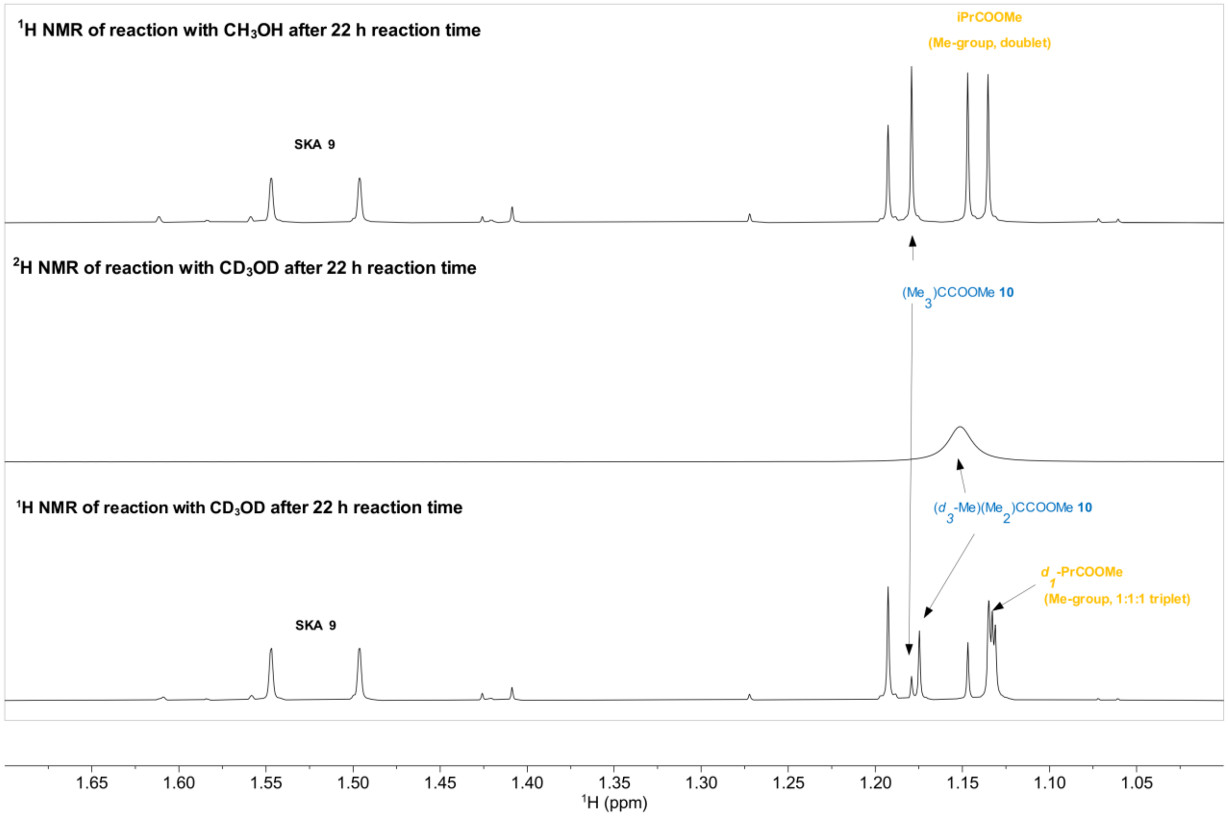

Figure 7: Aliphatic region of the ^{1}H and ^{2}H NMR spectra of the reaction in presence of CD_3OD in comparison to the reaction with CH3OH after 22 h of reaction time.

Structural assignment of all main compounds by 1H HMBC measurement:

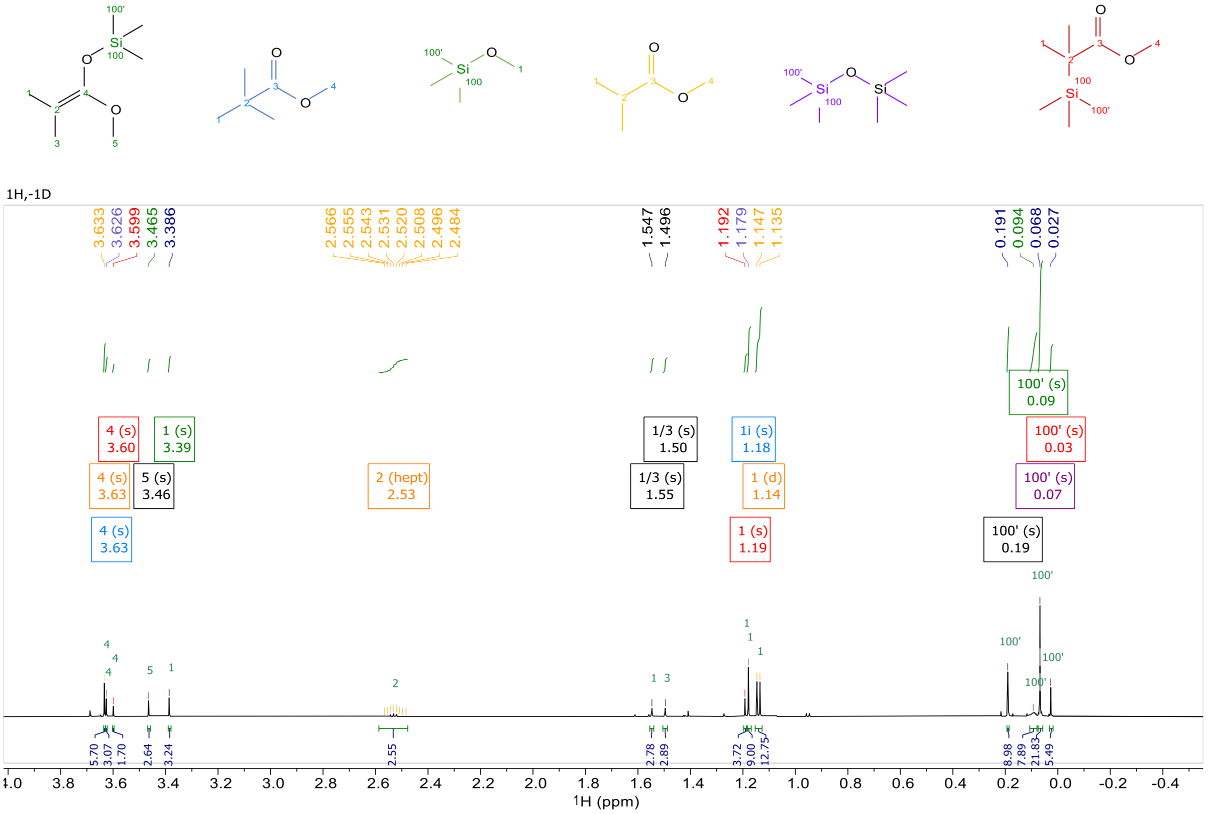

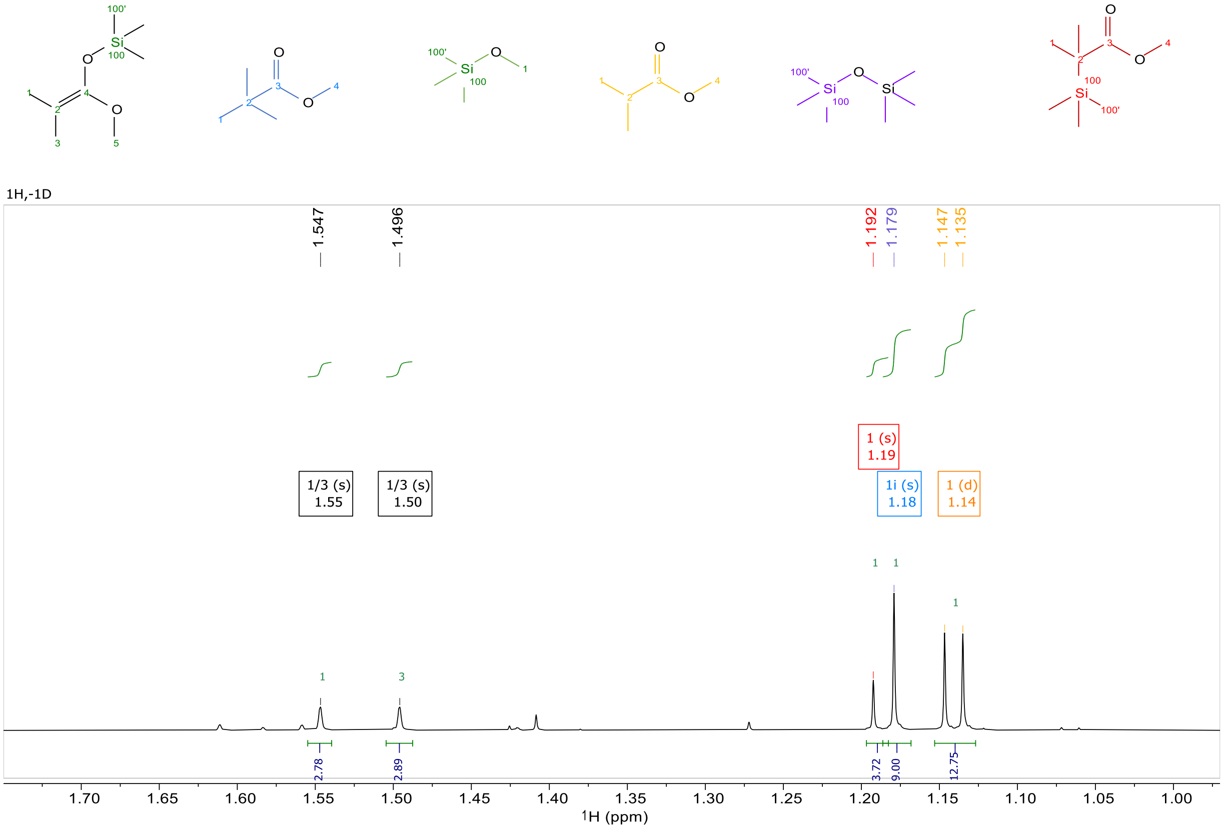

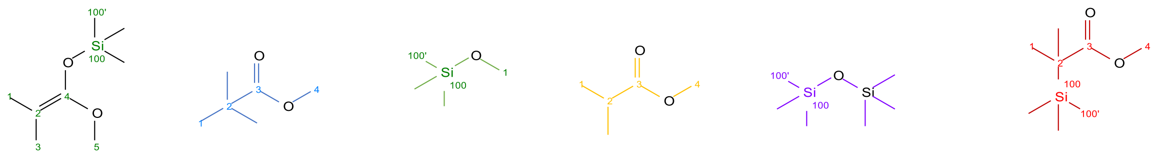

1H,-1D

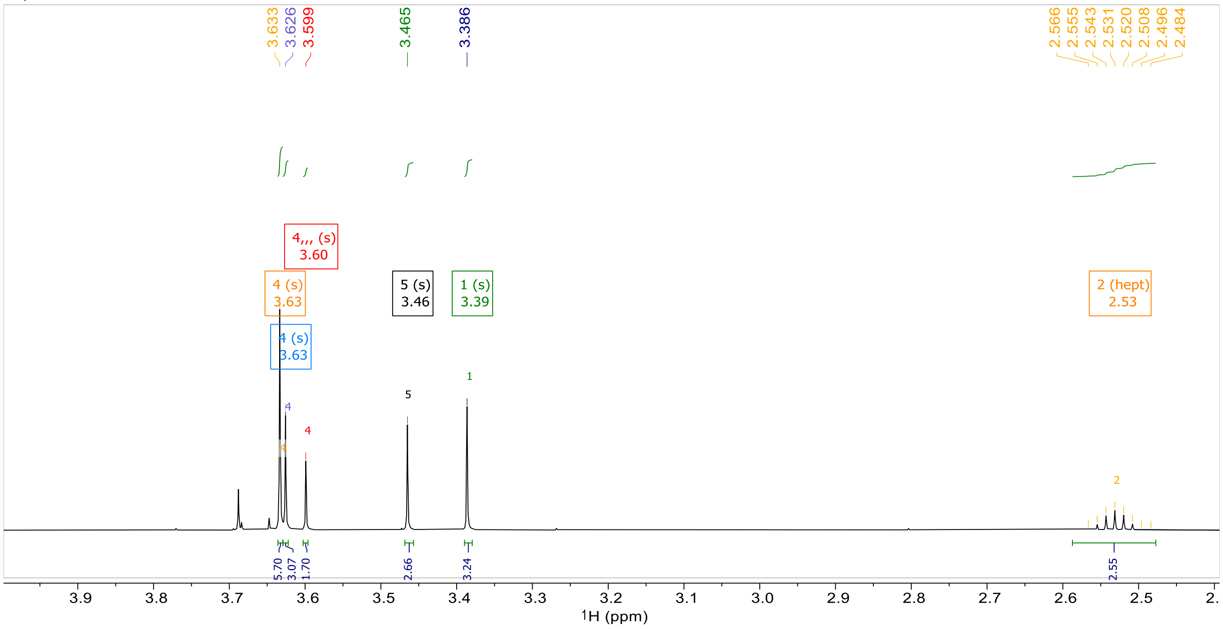

1H,13C-HMBC

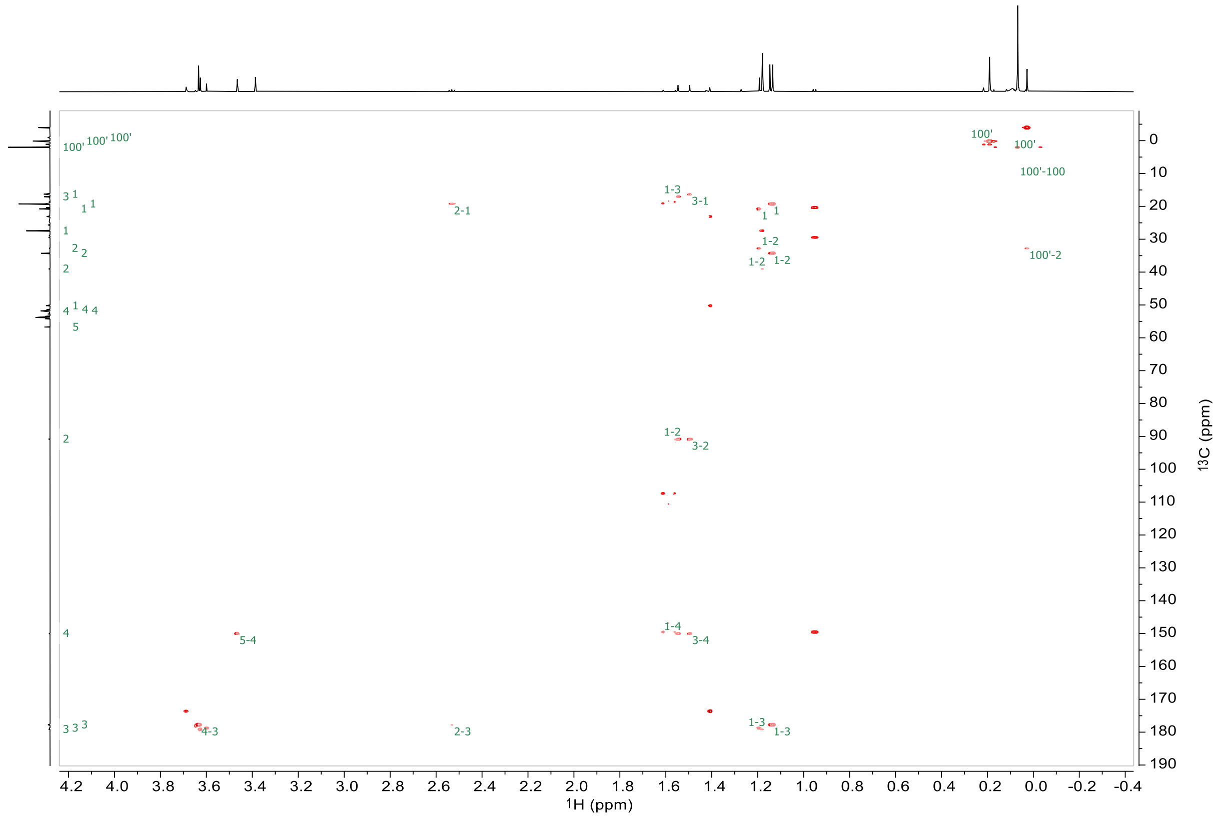

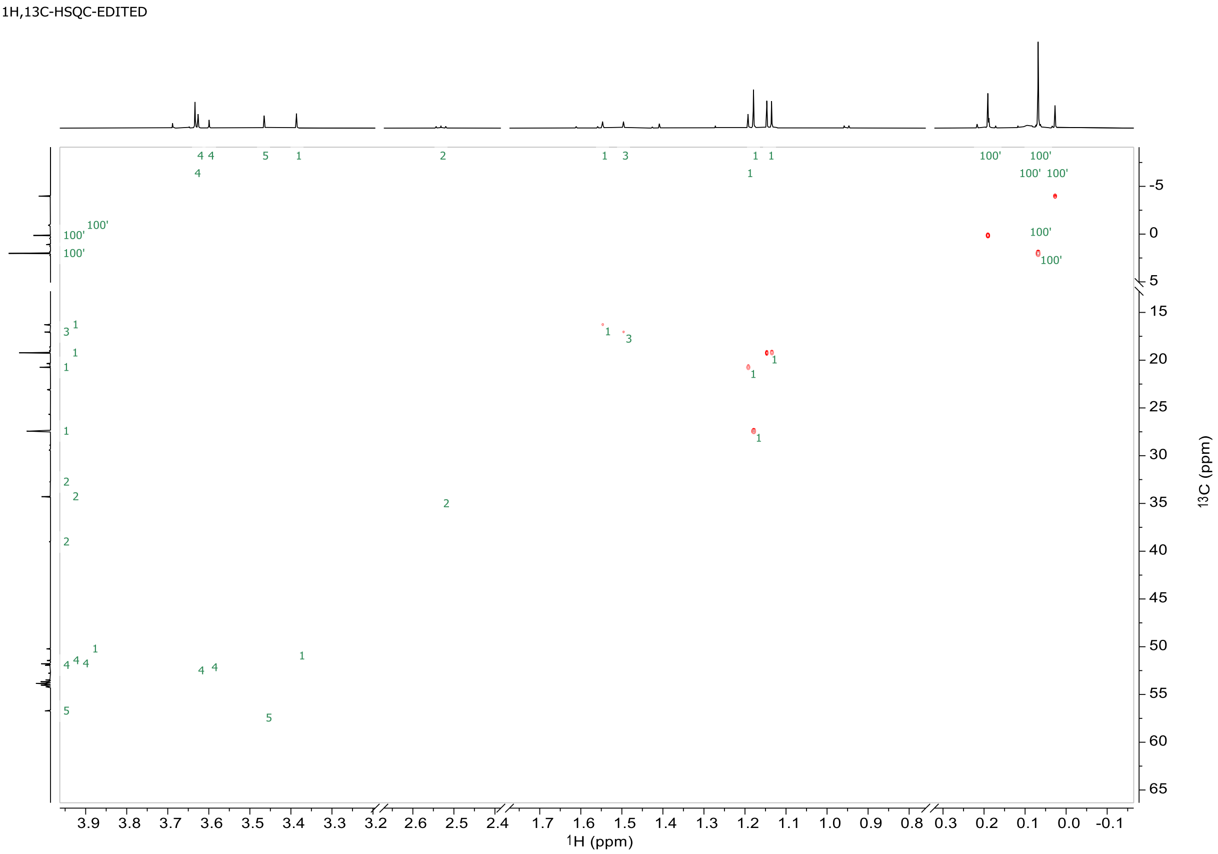
1H,13C-HSQC-EDITED
1H (ppm)
13C (ppm)

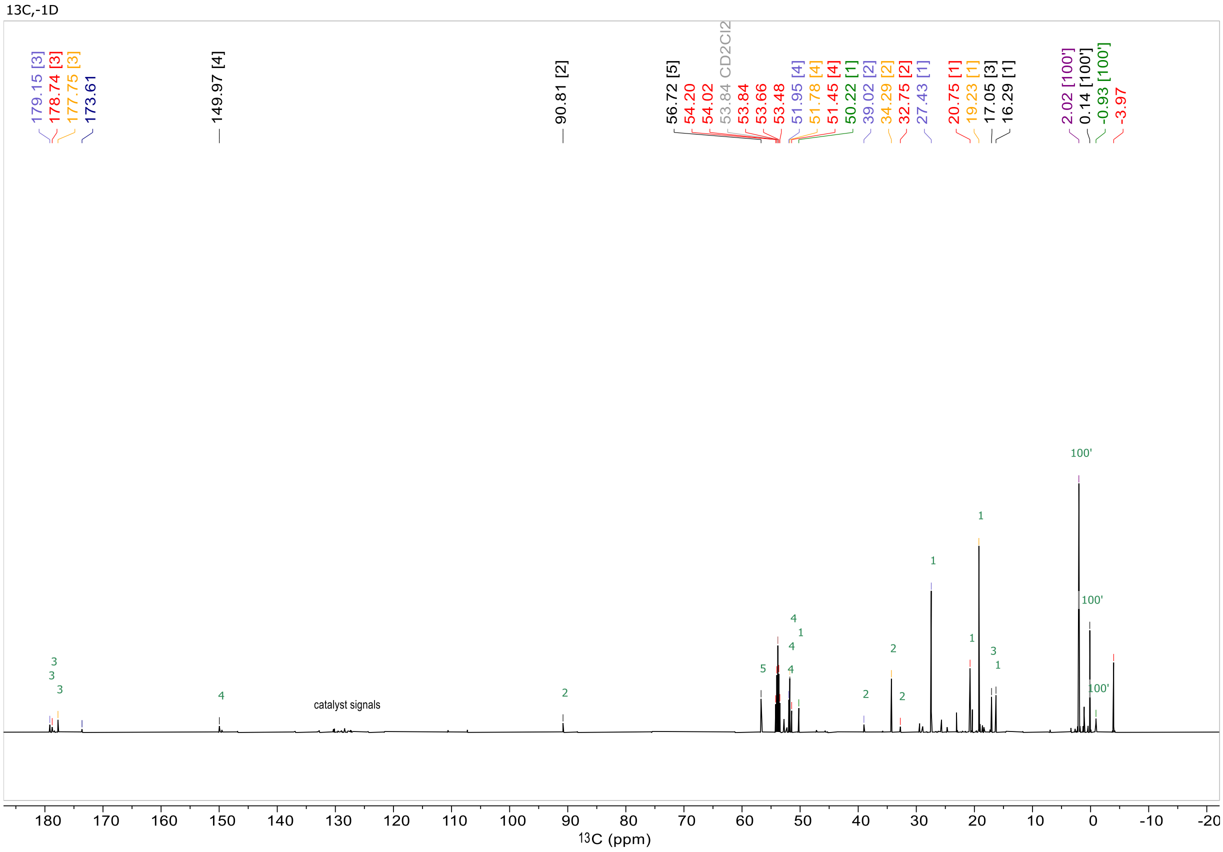
13C,-1D
179.15 [3]
178.74 [3]
177.75 [3]
173.61
149.97 [4]
90.81 [2]
56.72 [5]
54.20
54.02
53.84 CD2Cl2
53.84
53.66
53.48
51.95 [4]
51.78 [4]
51.45 [4]
50.22 [1]
39.02 [2]
34.29 [2]
32.75 [2]
27.43 [1]
20.75 [1]
19.23 [1]
17.05 [3]
16.29 [1]
2.02 [100']
0.14 [100']
-0.93 [100']
-3.97
catalyst signals
13C (ppm)

2.3 Preliminary studies toward the asymmetric α-methylation

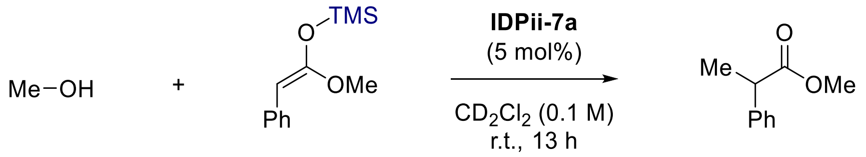

8 | **13** (5 equiv). *E*/*Z* = 9:1 | **14** **51% NMR-yield[a]** **50:50 e.r.**

[a]Yields were determined by 1H-NMR spectroscopy with mesitylene as internal standard.

An initial experiment toward the enantioselective α-methylation of silyl ketene acetal **13** shows promising NMR-yields of the desired product **14**, but unfortunately without any enantioenrichment. The application of various silyl ketene acetals and further optimization studies are currently in progress.

3. Synthesis of HCPP

3.1 One-step synthesis of hexachlorobisphosphazonium hexachlorophosphate (HCPP)

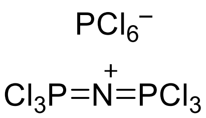

A 250 ml two necked round bottom flask, equipped with a reflux condenser and sulfuric acid filled gas bubbler, was flame-dried under argon. The flask was charged with PCl_5 (53.0 g, 254 mmol, 1 equiv.), NH_4Cl (4.31 g, 80.6 mmol, 0.95 equiv.) and suspended in nitrobenzene (80 ml). The suspension was heated 5.5 h to 130 °C. Within the first 3 h a constant gas development was observed, whereas the gas development slowly ceased after that time and most of the solid dissolved during the reaction progress (Note: sublimed PCl_5 was re-dissolved into the reaction mixture by carefully shaking the glas aparatus). The hot reaction mixture was filtered under inert conditions (argon overpressure through filter paper fitted PE-tube) into a 100 ml Schlenk-flask. Upon cooling to r.t., a colorless precipitate formed from the filtrate. The suspension was left o.n. at r.t. and filtered under inert reaction conditions (argon overpressure through filter paper fitted PE-tube). The beige solid was extensively washed with dry hexanes until the filtrates remain colorless and additionally washed with DCM (100 ml) to afford the desired product as a colorless solid (62%, 28.3 g, 53.2 mmol).

^{31}P NMR (203 MHz, CD_2Cl_2) δ=21.63 (s, 2P), –297.93 ppm (s, 1P).

Reference: *Z. Anorg. Allg. Chem.* **1977**, *433*, 229

Note: This synthesis is more practicable in comparison to the two-step synthesis (see 3.3 Alternative two-step synthesis of **HCPP**) due to a simple purification procedure and scalability.

3.2 Synthesis of hexachlorobisphosphazonium chloride (HCPC)

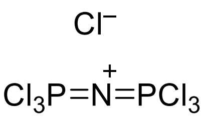

A 100 ml Schlenk-flask was charged with hexachlorobisphosphazonium hexachlorophosphate (28.3 g, 53.2 mmol, 1 equiv.), which was then suspended in DCM (60 ml), followed by portion-wise addition of 4-Dimethylaminopyridine (6.50 g, 53.2 mmol, 1 equiv.) to form a dark yellow solution, of which a colorless solid precipitated upon few minutes. The reaction mixture was stirred 1 h at r.t., followed by inert filtration (argon overpressure through filter paper fitted PE-tube). The remaining yellowish solid was washed with DCM (100 ml each run), where each time the solid was suspended and thoroughly stirred in DCM at r.t. and cooled in an ice-bath before the filtration process. The washing process was repeated three times until the filtrate remained colorless to afford the desired product as a colorless solid (87%, 14.9 g, 46.0 mmol).

^{31}P NMR (203 MHz, CD_2Cl_2) δ = –10.1 (s, 2P).
Reference: *Inorg. Chem.* **2004**, *43*, 2765

3.3 Alternative two-step synthesis of HCPP

3.3.1 Synthesis of (trimethylsilyl)phosphorimidoyl trichloride

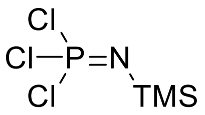

A 500 ml flame-dried two-neck round-bottom flask equipped with a dropping funnel was charged with diethylether (200 ml), hexamethyldisilazane (12.5 ml, 60 mmol), cooled to 0 °C (ice bath) followed by dropwise addition of *n*-butyllithium (24 ml, 2.5 M in hexanes, 60 mmol) within 5 minutes at 0 °C. The solution was stirred additional 30 minutes at 0 °C and slowly warmed to r.t. forming a colorless suspension. The suspension was cooled again to 0 °C (ice bath) followed by the dropwise addition of phosphorous trichloride (5.2 ml, 60 mmol) within 2 minutes. The cooling bath was removed after full addition of phosphorous trichloride and the colorless reaction suspension was allowed to warm to r.t. and stirred additional 30 minutes at r.t.. The colorless suspension was cooled to 0 °C (ice bath) followed by the dropwise addition of sulfuryl chloride (6 ml, 60 mmol). The colorless suspension was warmed to r.t. again and stirred additional 60 minutes at r.t. followed by inert filtration over a pad of previously dried celite (Schlenk frit, height 3 cm of celite) to obtain a colorless filtrate which was carefully concentrated (130 mbar to 30 mbar) at 0 °C under inert conditions. The resulting highly viscous oil was then purified by bulb-to-bulb distillation under static vacuum (5 mbar, 25 to 40 °C) condensing the desired product at –78 °C. (Trimethylsilyl)phosphorimidoyl trichloride was isolated as a colorless viscous oil (89%, 12 g, 53 mmol).

^{1}H NMR (501 MHz, CD_2Cl_2) δ= 0.17 ppm (d, $J_{H\text{-}P}$=1.1, 9H). $^{31}P\{^{1}H\}$ NMR (203 MHz, CD_2Cl_2) δ –55.35 ppm (s, 1P).

Reference: *Inorg. Chem.* **2002**, *41*, 1690

3.3.2 Synthesis of hexachlorobisphosphazonium hexachlorophosphate (HCPP)

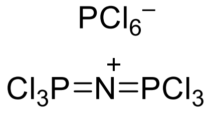

A flame-dried 100 ml Schlenk tube was charged with phosphorus pentachloride (16.6 g, 79.8 mmol) followed by the addition of a solution of (trimethylsilyl)phosphorimidoyl trichloride (12.0 g, 53.0 mmol) in dichloromethane (25 ml) at 0 °C to form a colorless suspension which was stirred 2.5 h at r.t.. Additional dichloromethane (20 ml) was added followed by inert filtration through a filter paper fitted PE-tube with argon pressure (1.3 bar). The colorless precipitate was washed followed by inert filtration two times with dichloromethane (40 ml each) and dried in high vacuum ($1x10^{-3}$ mbar) for 5 hours to obtain the desired product, as a colorless powder (15.4 g, 72%).

^{31}P NMR (203 MHz, CD_2Cl_2) δ=21.63 (s, 2P), –297.93 ppm (s, 1P).

Reference: *Inorg. Chem.* **2004**, *43*, 2765

4. Synthesis of BINOL

4.1 *(S)*-3,3´-bis(2,4,6-tri-n-hexylphenyl)-BINOL

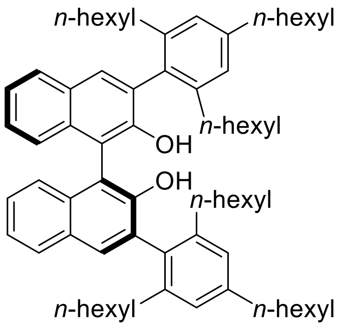

An under high vacuum flame-dried 50 ml Schlenk tube was argonated and charged with a freshly prepared solution of (2,4,6-tri-*n*-hexylphenyl)zinc(II)bromide (8.80 ml (c = 0.5 ml), 4.4 mmol, 4 equiv.) and (S)-3,3'-dibromo-2,2'-bis(methoxymethoxy)-1,1'-binaphthalene (585 mg, 1.1 mmol, 1 equiv.) followed by the addition of THF (10 ml). Pd(P(*t*-Bu)$_3$)$_2$ (16.9 mg, 33 µmol, 3 mol%) was added, the solution cooled in liquid nitrogen, degassed by vacuum suction and purged with argon. The reaction was heated to 60 °C for 24 h. HCl in dioxane (4 M, 12 ml, 48 mmol, 43 equiv.) was added and the resulting yellowish solution stirred for additional 24 h at r.t.. All volatiles were removed *in vacuo*, the residue dissolved in CH_2Cl_2, transferred to a separation funnel followed by the addition of water. The aqueous phase was extracted with CH_2Cl_2 (3x30 ml), the combined organic phase dried over sodium sulfate, concentrated to dryness and further purified by FCC (gradient: hexanes / CH_2Cl_2 95:5 to 90:10) to elute the desired product as yellowish oil (845 mg, 82%).

^{1}H NMR (501 MHz, CD_2Cl_2) δ=7.89 (dd, *J*=8.2, 1.3 Hz, 2H), 7.76 (s, 2H), 7.38 (ddd, *J*=8.0, 6.7, 1.2 Hz, 2H), 7.30 (ddd, *J*=8.2, 6.8, 1.3 Hz, 2H), 7.20 (dd, *J*=8.4, 1.1 Hz, 2H), 7.03 (d, *J*=3.6 Hz, 4H), 4.97 (s, 2H), 2.66–2.59 (m, 4H), 2.55–2.46 (m, 2H), 2.46–2.38 (m, 4H), 2.38–2.26 (m, 2H), 1.67 (ddd, *J*=13.0, 8.5, 6.4 Hz, 4H), 1.50–1.29 (m, 20H), 1.26–1.13 (m, 12H), 1.13–1.02 (m, 12H), 0.94–0.86 (m, 6H), 0.78 (t, *J*=7.1 Hz, 6H), 0.68 ppm (t, *J*=6.9 Hz, 6H). ^{13}C NMR (126 MHz, CD_2Cl_2) δ=150.9, 143.4, 142.6, 142.5, 134.0, 132.4, 131.6, 129.6, 129.5, 128.7, 127.4, 127.4, 127.0, 124.6, 124.1, 113.6, 36.3, 34.5, 34.3, 32.2, 32.0, 31.9, 31.5, 31.4, 29.9, 29.6, 29.6, 23.1, 23.0, 22.9, 14.3, 14.2, 14.1 ppm. (other signals not observed or detected). HRMS (ESI): m/z calcd for $C_{68}H_{93}O_2^-$: 941.719160 [M-H]$^-$, found 941.718105. $[\alpha]_D^{25}$ = –46 (c = 1 mg/ml in CH_2Cl_2)

4.2 *(S)*-3,3´-bis(2,4,6-tri-n-pentylphenyl)-BINOL

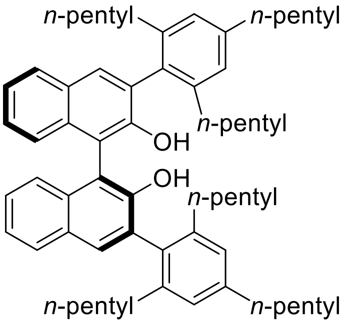

The BINOL has been synthesized based on example **4.1.** FCC-conditions: (gradient: hexanes / CH_2Cl_2 95:5 to 80:20); 71% yield (yellowish oil).

^{1}H NMR (501 MHz, CD_2Cl_2) δ=7.89 (dd, *J*=8.3, 1.3 Hz, 2H), 7.76 (s, 2H), 7.38 (ddd, *J*=8.1, 6.7, 1.2 Hz, 2H), 7.30 (ddd, *J*=8.2, 6.7, 1.3 Hz, 2H), 7.20 (dd, *J*=8.4, 1.1 Hz, 2H), 7.03 (d, *J*=4.4 Hz, 4H), 4.98 (s, 2H), 2.66–2.60 (m, 4H), 2.55–2.46 (m, 2H), 2.45–2.36 (m, 4H), 2.32 (dt, *J*=13.7, 7.6 Hz, 2H), 1.72–1.62 (m, 4H), 1.50–1.45 (m, 4H), 1.42 (dd, *J*=8.5, 6.7 Hz, 4H), 1.40–1.36 (m, 8H), 1.21 (qd, *J*=3.7, 1.6 Hz, 8H), 1.09 (dddt, *J*=10.3, 5.5, 3.5, 2.1 Hz, 8H), 0.95–0.90 (m, 6H), 0.82–0.77 (m, 6H), 0.69 ppm (t, *J*=6.9 Hz, 6H). ^{13}C NMR (126 MHz, CD_2Cl_2) δ=151.0, 143.3, 142.5, 142.5, 133.9, 132.4, 131.6, 129.6, 129.5, 128.7, 127.4, 127.3, 127.0, 124.6, 124.1, 113.5, 36.2, 34.4, 34.2, 32.4, 32.1, 32.0, 31.6, 31.2, 31.0, 23.0, 22.8, 22.8, 14.3, 14.2, 14.0 ppm (other signals not observed or detected). HRMS (ESI): m/z calcd for $C_{62}H_{82}O_2^-$: 858.631366 $[M-H]^-$, found 858.631480. $[\alpha]_D^{25}$ = –50 (c = 1 mg/ml in CH_2Cl_2)

4.3 *(S)*-3,3´-bis(2,4,6-tri-n-heptylphenyl)-BINOL

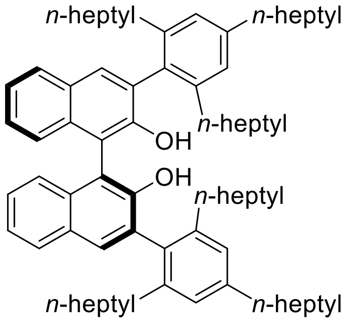

The BINOL has been synthesized based on example **4.1.** FCC-conditions: (gradient: hexanes / CH_2Cl_2 95:5 to 80:20); 53% yield (yellowish oil).

53% yield; ^{1}H NMR (501 MHz, CD_2Cl_2) δ=7.89 (d, *J*=7.7 Hz, 2H), 7.75 (s, 2H), 7.38 (ddd, *J*=8.1, 6.8, 1.3 Hz, 2H), 7.29 (ddd, *J*=8.2, 6.8, 1.3 Hz, 2H), 7.19 (dd, *J*=8.5, 1.1 Hz, 2H), 7.03 (d, *J*=3.9 Hz, 4H), 4.96 (s, 2H), 2.66–2.59 (m, 4H), 2.50 (dt, *J*=13.5, 7.8 Hz, 2H), 2.46–2.36 (m, 4H), 2.36–2.28 (m, 2H), 1.70–1.62 (m, 4H), 1.50–1.42 (m, 4H), 1.42–1.35 (m, 10H), 1.35–1.28 (m, 10H), 1.23–1.14 (m, 16H), 1.13–1.00 (m, 16H), 0.93–0.88 (m, 6H), 0.82–0.77 (m, 6H), 0.76–0.70 ppm (m, 6H). ^{13}C NMR (126 MHz, CD_2Cl_2) δ=146.1, 138.5, 137.7, 137.7, 129.1, 127.5, 126.7, 124.8, 124.6, 123.9, 122.6, 122.2, 119.8, 119.2, 108.8, 31.4, 29.7, 29.4, 27.5, 27.4, 27.3, 27.1, 26.7, 26.6, 25.4, 25.1, 24.8, 24.6, 18.3, 18.2, 18.1, 9.5, 9.4, 9.4 ppm. (other signals not observed or detected). HRMS (ESI): m/z calcd for $C_{74}H_{105}O_2^-$: 1025.812005$[M-H]^-$, found 1025.812920. $[\alpha]_D^{25}$ = –42 (c = 1 mg/ml in CH_2Cl_2)

4.4 *(S)*-3,3´-bis(4-((trifluoromethyl)thio)phenyl)-BINOL

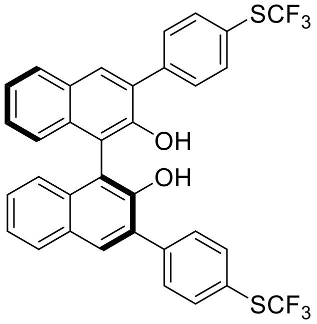

An under high vacuum flame-dried and argonated Schlenk flask equipped with a stirring bar was charged with *(S)*-3,3'-di(pinacolboryl)-2,2'-bis(methoxymethoxy)-1,1'-binaphthalene (1.03 g, 1.64 mmol, 1.0 eq.), $Ba(OH)_2$ octahydrate (2.1 g, 6.64 mmol, 4.0 eq.), 4-(trifluoromethylthio)-bromobenzene (0.7 ml, 4.63 mmol, 2.8 eq.), dioxane (10 ml) and water (2.5 ml). The reaction mixture was degassed followed by the addition of $[Pd(PPh_3)_4]$ (5 mol%), the reaction flask was sealed and heated to reflux overnight. After cooling to r.t., water was added and the reaction mixture extracted with CH_2Cl_2. The combined organic layers were washed with brine, dried over Na_2SO_4, filtered and concentrated to dryness to obtain a yellowish oil, which was suspended in HCl / dioxane (4 M), stirred overnight and then concentrated to dryness. After column chromatography (hexanes / EtOAc 10:1) the desired product was obtained as yellowish solid (81%, 850 mg, 1.33 mmol).

^{1}H NMR (501 MHz, CD_2Cl_2) δ = 8.10 (s, 2H), 8.02 – 7.96 (m, 2H), 7.88 – 7.82 (m, 4H), 7.82 – 7.76 (m, 4H), 7.44 (ddd, *J*=8.2, 6.9, 1.4, 2H), 7.36 (ddd, *J*=8.2, 6.9, 1.4, 2H), 7.23 – 7.17 (m, 2H), 5.44 ppm (s, 2H). ^{13}C NMR (126 MHz, CD_2Cl_2) δ = 150.2, 140.6, 136.2, 133.2, 132.0, 131.0, 130.8, 129.5, 129.3, 128.7, 128.6, 127.8, 124.6, 124.0, 123.4, 123.4, 112.1 ppm. ^{19}F NMR (471 MHz, $CDCl_3$) δ –43.0 ppm (s). HRMS (ESI): *m*/*z* calcd for $C_{34}H_{19}F_6O_2S_2^-$: 637.073621 [*M*–H]$^-$; found: 637.074070; $[\alpha]_D^{25}$ = –18 (c = 1 mg/ml in CH_2Cl_2)

5. Synthesis of imidodiphosphates (IDP)

5.0 Initial NMR experiments

A 10 ml schlenk tube was charged with HCPP (160 mg, 300 µmol, 1 equiv.), (*S*)-3,3´-(phenyl)BINOL (268 mg, 611 µmol, 2 equiv.) followed by the addition of pyridine (3 ml). The reaction was stirred 15 minutes at r.t.. An aliquote (300 µl) was transferred to an NMR tube under inert conditions, diluted with anhydrous CD_2Cl_2 (300 µl) followed by NMR analysis. After NMR analysis, water (ca. 100 µl) was added to the NMR tube and the NMR sample re-measured within ca. five minutes.

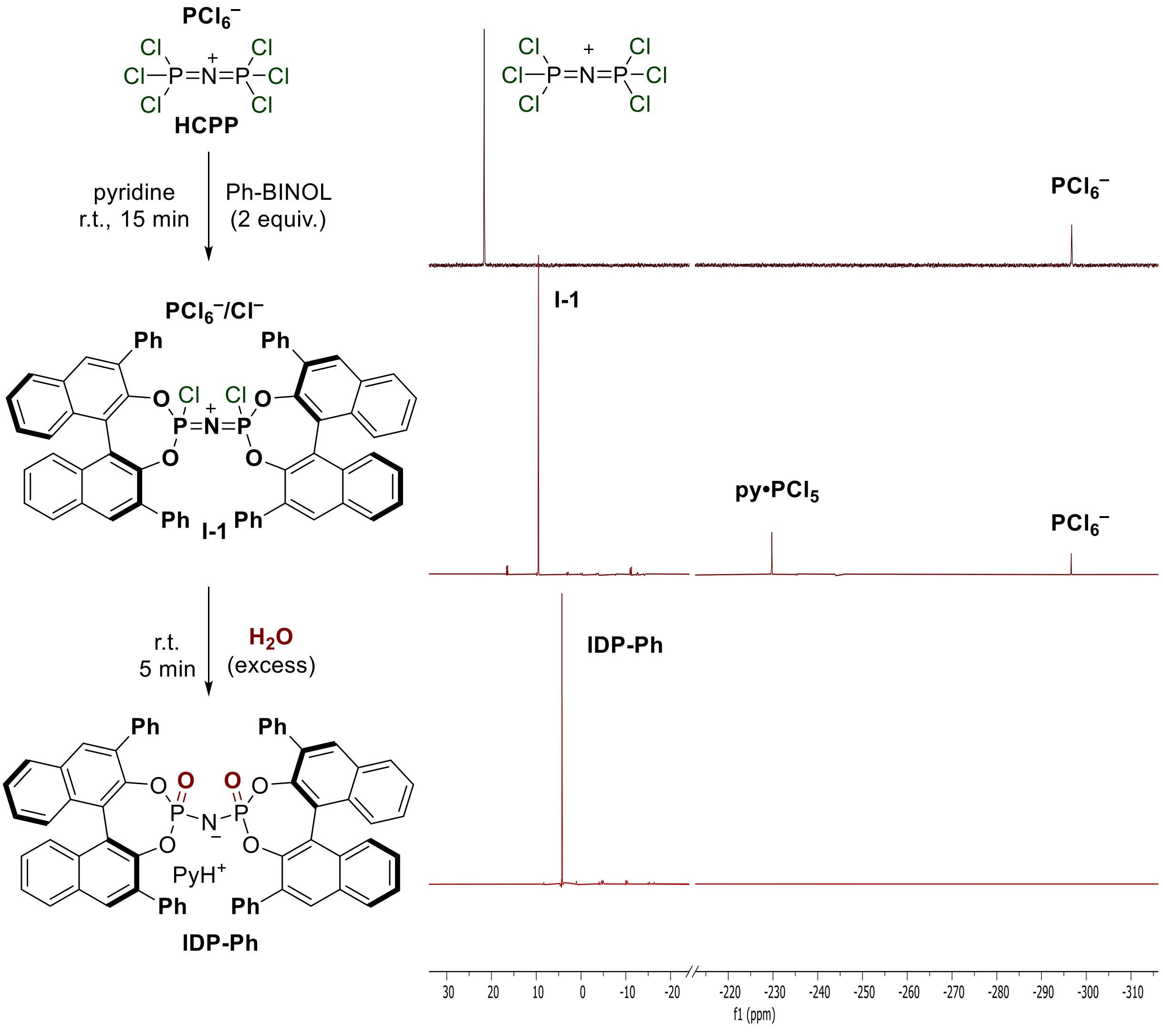

5.1 IDP-1a

(S,S)-4,4'-azanediylbis(2,6-bis(2,4,6-triisopropylphenyl)dinaphtho[2,1-d:1',2'-f][1,3,2]dioxaphosphepine 4-oxide)

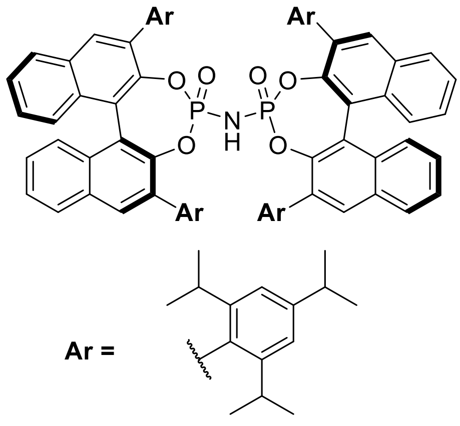

A flame-dried Schlenk was charged with HCPP (58.0 mg, 109 µmol 1 equiv.), (*S*)-TRIP-BINOL (155 mg, 224 µmol, 2.06 equiv.) followed by the addition of pyridine (2.2 ml). The resulting suspension was stirred 22 h at r.t. followed by the addition of water (400 µl, 22.2 mmol, 204 equiv.) and stirred additional 48 h at 50 °C. After cooling to r.t., the reaction mixture was poured into aqueous HCl (6 M, 20 ml) and extracted with CH_2Cl_2 (3x20 ml). The combined organic phase was dried over sodium sulfate, concentrated to dryness and the resulting crude product was purified by FCC (gradient: hexanes / CH_2Cl_2 4:1 up to 2:3) to elute the desired product as a salt. The salt was dissolved in CH_2Cl_2 (15 ml), treated with aqueous HCl (6 M, 15 ml) under vigorous for 30 minutes, the organic phase was separated, concentrated to dryness and dried o.n. in h.v. to furnish the desired product in acidic form as a colorless solid (120 mg, 74%)

^{1}H NMR (501 MHz, CD_2Cl_2) δ=7.89 (d, *J*=8.2 Hz, 2H), 7.87–7.80 (m, 4H), 7.54 (s, 2H), 7.48 (ddd, *J*=8.1, 6.7, 1.1 Hz, 2H), 7.44 (ddd, *J*=8.2, 6.8, 1.1 Hz, 2H), 7.27 (ddd, *J*=8.3, 6.8, 1.3 Hz, 2H), 7.19 (ddd, *J*=8.4, 6.8, 1.3 Hz, 4H), 6.99 (d, *J*=8.9 Hz, 2H), 6.97 (t, *J*=2.5 Hz, 4H), 6.84 (d, *J*=1.8 Hz, 2H), 6.75 (d, *J*=1.8 Hz, 2H), 5.58 (s, 3H), 2.84 (p, *J*=6.9 Hz, 2H), 2.76 (p, *J*=6.9 Hz, 2H), 2.64–2.48 (m, 4H), 2.43 (p, *J*=6.8 Hz, 2H), 1.28 (d, *J*=6.9 Hz, 6H), 1.23–1.20 (m, 12H), 1.16 (d, *J*=6.9 Hz, 6H), 1.12 (d, *J*=6.9 Hz, 6H), 1.04 (d, *J*=6.8 Hz, 6H), 1.01 (d, *J*=6.8 Hz, 7H), 0.93 (d, *J*=6.7 Hz, 6H), 0.86 (d, *J*=6.8 Hz, 6H), 0.75 (d, *J*=6.8 Hz, 6H), 0.43 (d, *J*=6.8 Hz, 6H), -0.20 ppm (d, *J*=6.8 Hz, 6H). ^{13}C NMR (126 MHz, CD_2Cl_2) δ=149.0, 148.5, 148.3, 147.9, 147.6, 146.9, 146.8, 146.8, 146.1, 133.6, 133.4, 132.9, 132.8, 132.6, 131.7, 131.5, 130.9, 128.8, 128.1, 127.7, 127.2, 126.6, 126.5, 126.0, 126.0, 122.6, 122.4, 121.8, 120.7, 54.4, 54.2, 54.0, 53.8, 53.6, 34.7, 34.6, 31.8, 31.5, 31.2, 31.1, 27.3, 25.9, 25.4, 24.8, 24.6, 24.5, 24.0, 24.0, 23.8, 23.7, 23.3, 22.6 ppm. (other signals not observed or detected). ^{31}P NMR (203 MHz CD_2Cl_2) δ=13.96 (s). HRMS (ESI): m/z calcd for $C_{148}H_{210}N_1O_6P_2$+H^+: 1484.797043 $[M+H]^+$, found 1484.798910 $[\alpha]_D^{25}$ = +22 (c = 1 mg/ml in CH_2Cl_2)

5.2 IDP-1b

(S,S)-4,4'-azanediylbis(2,6-bis(2,4,6-tripentylphenyl)dinaphtho[2,1-d:1',2'-f][1,3,2]dioxaphosphepine 4-oxide)

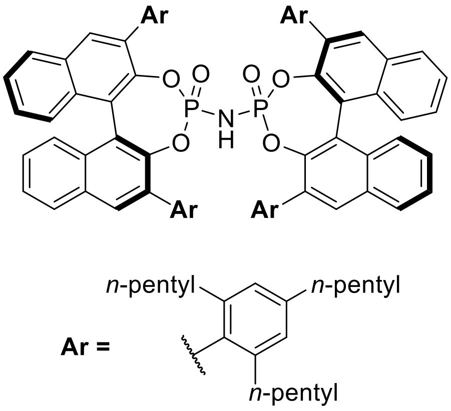

A 50 ml flame-dried and argonated Schlenk tube was charged with HCPP (509 mg, 0.95 mmol), dissolved in pyridine (10 ml) followed by immediate addition of a solution of (S)-3,3´-bis(2,4,6-tri-n-pentylphenyl)-BINOL (1.69 g, 1.96 mmol, 2.05 equiv.) in pyridine (10 ml). The slightly yellowish solution was stirred at r.t. for 18 h followed by the addition of water (5 ml, 290 equiv.) and stirred additional 4 h at 60 °C until full conversion was observed by ^{31}P-NMR. The reaction mixture was diluted with additional water (15 ml) resulting in the formation of a colorless suspension which upon addition of CH_2Cl_2 (20 ml) formed an emulsion, which was transferred to a separation funnel. The aqueous phase was extracted with CH_2Cl_2 (twice 100 ml each) and EtOAc (50 ml). The combined organic phase was washed with sat. $NaHCO_3$, dried over sodium sulfate, concentrated to dryness and further purified by FCC (gradient: hexanes / CH_2Cl_2 (4:1) up to hexanes / CH_2Cl_2 (1:1) to elute the desired product as a colorless foam. The product in salt form was dissolved in CH_2Cl_2 (10 ml) followed by the addition of HCl 6 N (10 ml) and the resulting emulsion stirred vigorously for 30 minutes. The CH_2Cl_2layer was isolated, concentrated to dryness and dried o.n. in h.v. at r.t. to furnish the desired product in acidic form as a highly viscous oil (83% yield, 1.45 g).

^{1}H NMR (501 MHz, CD_2Cl_2) δ=7.90–7.82 (m, 4H), 7.77 (s, 2H), 7.56 (s, 2H), 7.49 (ddd, *J*=8.1, 6.5, 1.4 Hz, 2H), 7.45–7.38 (m, 4H), 7.36 (ddd, *J*=8.3, 6.6, 1.3 Hz, 2H), 7.18 (ddd, *J*=8.3, 6.8, 1.3 Hz, 2H), 7.01 (d, *J*=8.6 Hz, 2H), 6.92 (d, *J*=1.8 Hz, 2H), 6.80 (t, *J*=2.0 Hz, 4H), 6.38 (d, *J*=1.8 Hz, 2H), 5.19 (s, 1H), 2.51 (ddd, *J*=16.1, 8.6, 6.1 Hz, 8H), 2.36 (ddd, *J*=14.2, 8.1, 6.5 Hz, 2H), 2.21 (ddd, *J*=14.2, 8.1, 6.7 Hz, 2H), 2.15 (t, *J*=8.2 Hz, 4H), 2.09–2.02 (m, 2H), 1.99 (ddd, *J*=9.4, 6.4, 2.6 Hz, 4H), 1.67–1.47 (m, 12H), 1.47–1.39 (m, 2H), 1.39–1.25 (m, 20H), 1.24–1.03 (m, 20H), 0.90–0.83 (m, 18H), 0.79 (dt, *J*=11.0, 7.0 Hz, 18H), 0.64 (t, *J*=7.3 Hz, 6H), 0.61–0.50 (m, 6H), 0.45 (t, *J*=6.8 Hz, 6H), 0.33–0.23 ppm (m, 2H). ^{13}C NMR (126 MHz, CD_2Cl_2) δ=146.7, 146.0, 142.9, 142.6, 141.6, 141.5, 133.4, 133.2, 133.0, 133.0, 132.6, 132.5, 132.4, 131.6, 131.1, 128.5, 128.5, 127.7, 127.2, 127.1, 126.8, 126.4, 126.2, 126.2, 125.9, 125.7, 122.7, 122.1, 36.3, 36.0, 34.4, 34.2, 34.1, 33.6, 32.6, 32.4, 32.3, 32.1, 32.1, 31.8, 31.6, 31.5, 31.2, 31.0, 23.3, 23.0, 23.0, 22.9, 22.5, 14.3, 14.3, 14.2, 14.1, 13.8 ppm. (other signals not observed or detected). ^{31}P NMR (203 MHz, CD_2Cl_2) δ=4.68 ppm. HRMS (ESI): *m/z* calcd for $C_{124}H_{160}N_1O_6P_2^-$: 1821.172643 [*M*–H]$^-$; found: 1821.174180; $[\alpha]_D^{25}$ = –14 (c = 1 mg/ml in CH_2Cl_2)

5.3 IDP-1c

(S,S)-4,4'-azanediylbis(2,6-bis(2,4,6-trihexylphenyl)dinaphtho[2,1-d:1',2'-f][1,3,2]dioxaphosphepine 4-oxide)

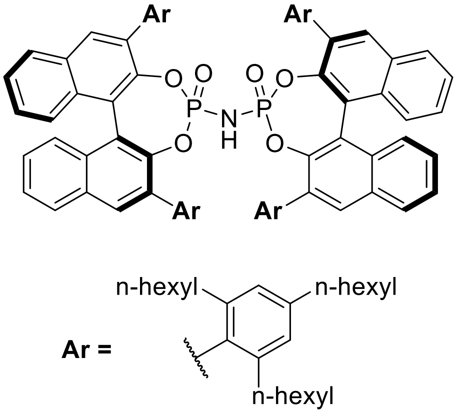

A 10 ml flame-dried and argonated Schlenk tube was charged with HCPP (46 mg, 86,4 µmol) and dissolved in a solution of (*S*)-(o,o,p-tri(n-hexyl)phenyl)BINOL (163 mg, 0,17 mmol 2 equiv. in 1.5 ml pyridine). The reaction was stirred for 19 h at r.t. followed by the addition of water (300 µl, 16.6 mmol, 192 equiv.) and stirred additional 5 h at 80 °C until ^{31}P-NMR analysis shows clean hydrolysis to the desired product. After cooling to r.t. the reaction mixture was poured into 30 ml $HCl_{(aq)}$ and extracted with CH_2Cl_2 (3x30 ml). The combined organic phases were dried over sodium sulfate, concentrated to dryness and purified by FCC (Biotage, gradient: hexanes up to hexanes / CH_2Cl_2 (1:1)) to elute the desired product as a salt. Acidification was carried out by dissolving the salt in CH_2Cl_2 (3 ml) followed by the addition of HCl 6 N (3 ml) and the resulting emulsion stirred vigorously for 30 minutes. The organic phase was isolated, concentrated to dryness followed by drying o.n. in h.v. to furnish the desired product in acidic form as a colorless viscous oil (78%, 172 mg)

^{1}H NMR (501 MHz, CD_2Cl_2) δ=7.86 (dd, *J*=13.8, 8.2 Hz, 4H), 7.77 (s, 2H), 7.56 (s, 2H), 7.49 (dd, *J*=8.3, 6.6 Hz, 2H), 7.42 (dd, *J*=16.8, 8.6 Hz, 4H), 7.37–7.33 (m, 2H), 7.17 (ddd, *J*=8.3, 6.7, 1.3 Hz, 2H), 7.01 (d, *J*=8.6 Hz, 2H), 6.92 (s, 2H), 6.80 (dd, *J*=4.8, 1.7 Hz, 4H), 6.37 (d, *J*=1.6 Hz, 2H), 6.05 (sbr, 1H), 2.59–2.43 (m, 8H), 2.33 (dt, *J*=14.2, 7.2 Hz, 2H), 2.23 (dt, *J*=14.1, 7.3 Hz, 2H), 2.16 (t, *J*=8.2 Hz, 4H), 2.08–1.92 (m, 6H), 1.65–1.48 (m, 12H), 1.46–1.39 (m, 3H), 1.38–1.26 (m, 28H), 1.25–1.09 (m, 28H), 1.08–0.98 (m, 6H), 0.95–0.82 (m, 20H), 0.82–0.74 (m, 23H), 0.57 (t, *J*=7.1 Hz, 12H), 0.32 ppm (dd, *J*=12.3, 6.7 Hz, 2H). ^{13}C NMR (126 MHz, CD_2Cl_2) δ=146.3, 145.6, 142.4, 142.1, 141.1, 141.0, 133.0, 132.8, 132.6, 132.5, 132.2, 132.1, 132.0, 131.1, 130.7, 128.1, 128.1, 127.3, 126.7, 126.5, 126.3, 126.0, 125.8, 125.7, 125.6, 125.5, 125.2, 122.3, 121.7, 36.0, 35.6, 34.1, 33.7, 33.7, 33.2, 32.2, 32.0, 31.8, 31.8, 31.7, 31.7, 31.5, 31.4, 31.2, 31.0, 31.0, 31.0, 29.6, 29.4, 29.3, 29.2, 28.8, 28.2, 22.7, 22.6, 22.5, 22.5, 22.3, 14.0, 13.9, 13.8, 13.8, 13.5 ppm. (other signals not observed or detected). ^{31}P NMR (203 MHz, CD_2Cl_2) δ=4.73 ppm (s, 2P). HRMS (ESI): m/z calcd for $C_{136}H_{186}N_1O_6P_2+H^+$: 1991.374993 $[M+H]^+$, found 1991.375490; $[\alpha]_D^{25}$ = –8 (c = 0.05 mg/ml in CH_2Cl_2)

5.4 IDP-1d

(S,S)-4,4'-azanediylbis(2,6-bis(2,4,6-triheptylphenyl)dinaphtho[2,1-d:1',2'-f][1,3,2]dioxaphosphepine 4-oxide)

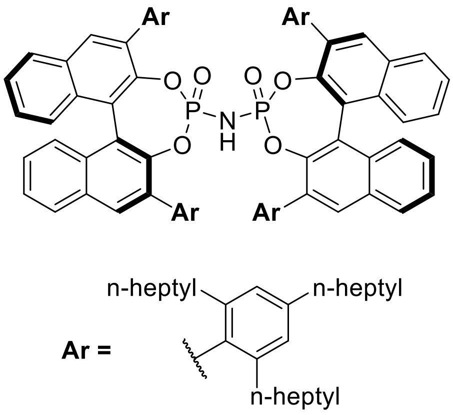

A 10 ml flame-dried and argonated Schlenk tube was charged with HCPP (117 mg, 220 µmol) and dissolved in a solution of *(S)*-(o,o,p-tri(n-heptyl)phenyl)BINOL (490 mg, 0.48 mmol 2.1 equiv. in 4 ml pyridine). The reaction was stirred for 19 h at r.t. followed by the addition of water (800 µL, 44.4 mmol, 202 equiv.) and stirred additional 5 h at 80 °C until ^{31}P-NMR analysis shows clean hydrolysis to the desired product. After cooling to r.t. the reaction mixture was poured into 30 ml $HCl_{(aq)}$ and extracted with CH_2Cl_2 (3x30 ml). The combined organic phases were dried over sodium sulfate, concentrated to dryness and purified by FCC (Biotage, gradient: hexanes up to hexanes / CH_2Cl_2 (3:2)) to elute the desired product as a salt. Acidification was carried out by dissolving the salt in CH_2Cl_2 (5 ml) and $HCl_{(aq)}$ (6 M, 5 ml). The organic phase was isolated, concentrated to dryness followed by drying o.n. in h.v. to furnish the desired product as a colorless viscous oil (82%, 389 mg)

^{1}H NMR (501 MHz, CD_2Cl_2) δ=7.85 (dd, *J*=14.4, 7.9 Hz, 4H), 7.77 (s, 2H), 7.56 (s, 2H), 7.49 (ddd, *J*=8.1, 6.5, 1.3 Hz, 2H), 7.45–7.41 (m, 2H), 7.41–7.36 (m, 2H), 7.36–7.32 (m, 2H), 7.17 (ddd, *J*=8.3, 6.8, 1.4 Hz, 2H), 7.01 (d, *J*=8.6 Hz, 2H), 6.91 (d, *J*=1.7 Hz, 2H), 6.80 (dd, *J*=7.3, 1.7 Hz, 4H), 6.37 (d, *J*=1.7 Hz, 2H), 4.10 (sbr, 1H), 2.58–2.44 (m, 8H), 2.34 (dt, *J*=14.2, 7.2 Hz, 2H), 2.22 (dt, *J*=14.2, 7.3 Hz, 2H), 2.15 (t, *J*=8.2 Hz, 4H), 2.09–2.00 (m, 4H), 1.96 (ddd, *J*=13.5, 10.5, 5.8 Hz, 2H), 1.63–1.49 (m, 12H), 1.36–1.24 (m, 38H), 1.24–1.12 (m, 36H), 1.04–0.95 (m, 8H), 0.93–0.75 (m, 50H), 0.70 (t, *J*=7.1 Hz, 6H), 0.62–0.48 (m, 6H), 0.30 ppm (dt, *J*=11.8, 7.6 Hz, 2H). ^{13}C NMR (126 MHz, CD_2Cl_2) δ=146.8, 146.7, 146.7, 146.0, 146.0, 146.0, 142.9, 142.6, 142.5, 141.6, 141.4, 133.5, 133.2, 133.1, 133.0, 133.0, 132.6, 132.5, 132.4, 131.5, 131.1, 128.6, 128.5, 127.7, 127.2, 127.1, 126.8, 126.4, 126.2, 126.2, 126.1, 125.9, 125.7, 122.7, 122.1, 36.4, 36.1, 34.5, 34.2, 34.1, 33.7, 32.5, 32.4, 32.3, 32.3, 32.2, 32.0, 31.9, 31.5 (d, *J*=2.5), 30.3, 30.1, 30.1, 29.9, 29.7, 29.7, 29.6, 29.6, 29.5, 29.2, 28.9, 23.3, 23.1, 23.1, 23.1, 22.8, 14.4, 14.3, 14.3, 14.3, 14.3, 14.2 ppm. (other signals not observed or detected). ^{31}P NMR (203 MHz, CD_2Cl_2) δ=4.02 ppm (s, 2P). HRMS (ESI): m/z calcd for $C_{148}H_{210}N_1O_6P_2+H^+$: 2159.562792 $[M+H]^+$, found 2159.561040; $[\alpha]_D^{25}$ = –8 (c = 1 mg/ml in CH_2Cl_2)

5.5 IDP-1e

(S,S)-2,6-di(anthracen-9-yl)-4-((-2,6-di(anthracen-9-yl)-4-oxidodinaphtho[2,1-d:1',2'-f][1,3,2]dioxaphosphepin-4-yl)amino)dinaphtho[2,1-d:1',2'-f][1,3,2]dioxaphosphepine 4-oxide

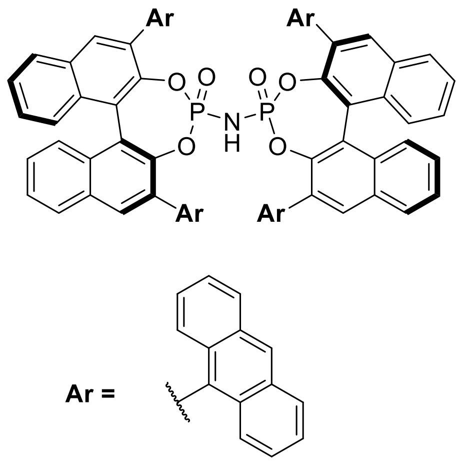

An under high vacuum 5 ml flame-dried Schlenk-tube was charged with hexachlorobisphosphazonium hexachlorophosphate (41 mg, 77 µmol), (*S*)-3,3'-di(anthracen-9-yl)-[1,1'-binaphthalene]-2,2'-diol (98.4 mg, 154 µmol) followed by the addition of pyridine (1 ml). The suspension was stirred for 15 minutes at r.t. followed by the addition of distilled water (250 µl, 13.8 mmol) and was stirred additional 4 h at r.t.. The reaction mixture was poured into aqueous HCl (6 M) solution and extracted with dichloromethane. The combined organic phase was dried over sodium sulfate, concentrated in vacuo and purified by flash column chromatography (dichloromethane) to afford a colorless solid, which was acidified with aqueous 6 M HCl. The solid was dissolved in a small quantitiy of dichloromethane and precipitated with hexanes. After decantation of the organic phase the desired product was obtained as a colorless powder (90 mg, 85%).

The NMR-data match with previous reported literature.

^{1}H NMR (501 MHz, CD_2Cl_2) δ = 8.19 (s, 2H), 8.07 – 8.01 (m, 4H), 7.90 (s, 2H), 7.88 – 7.85 (m, 2H), 7.85 – 7.81 (m, 2H), 7.79 (s, 2H), 7.74 – 7.69 (m, 7H), 7.69 – 7.63 (m, 7H), 7.52 (ddd, *J*=8.2, 6.7, 1.3, 2H), 7.49 (s, 2H), 7.48 – 7.43 (m, 4H), 7.41 – 7.37 (m, 2H), 7.36 – 7.32 (m, 2H), 7.31 – 7.24 (m, 6H), 7.14 – 7.07 (m, 4H), 6.88 (ddd, *J*=9.0, 6.5, 1.3, 2H), 5.88 (ddd, *J*=8.7, 6.5, 1.2, 2H), 5.59 – 5.52 (m, 2H), 2.13 ppm (sbr, 1H). ^{13}C NMR (126 MHz, CD_2Cl_2) δ = 146.57, 134.28, 133.03, 131.88, 131.59, 131.32, 131.18, 130.94, 130.89, 130.62, 129.11, 128.91, 128.83, 128.14, 127.75, 127.67, 127.64, 127.34, 126.55, 126.31, 126.14, 125.31, 125.28, 125.16, 122.61 ppm. ^{31}P NMR (203 MHz, CD_2Cl_2) δ = 12.83 ppm (s). HRMS (ESI): m/z calcd for $C_{96}H_{55}N_1O_6P_2^-$: 1380.358843[M–H]$^+$, found 1380.358390

6. Synthesis of iminoimidodiphosphate (iIDP)

6.0 Initial NMR experiments

A 10 ml schlenk tube was charged with HCPP (160 mg, 300 µmol, 1 equiv.), (*S*)-3,3´-(phenyl)BINOL (268 mg, 611 µmol, 2 equiv.) followed by the addition of pyridine (3 ml). The reaction was stirred 15 minutes at r.t.. An aliquote (300 µl) was transferred to an NMR tube under inert conditions, diluted with anhydrous CD_2Cl_2 (300 µl) followed by NMR analysis. Trifluoromethanesulfonamide (358 mg, 2.40 mmol, 8 equiv.) was added and the reaction stirred for additional 15 min at r.t.. An aliquote (300 µl) was transferred to an NMR tube under inert conditions, diluted with anhydrous CD_2Cl_2 (300 µl) followed by NMR analysis. After NMR analysis, water (ca. 100 µl) was added to the NMR tube and the NMR sample re-measured within ca. five minutes.

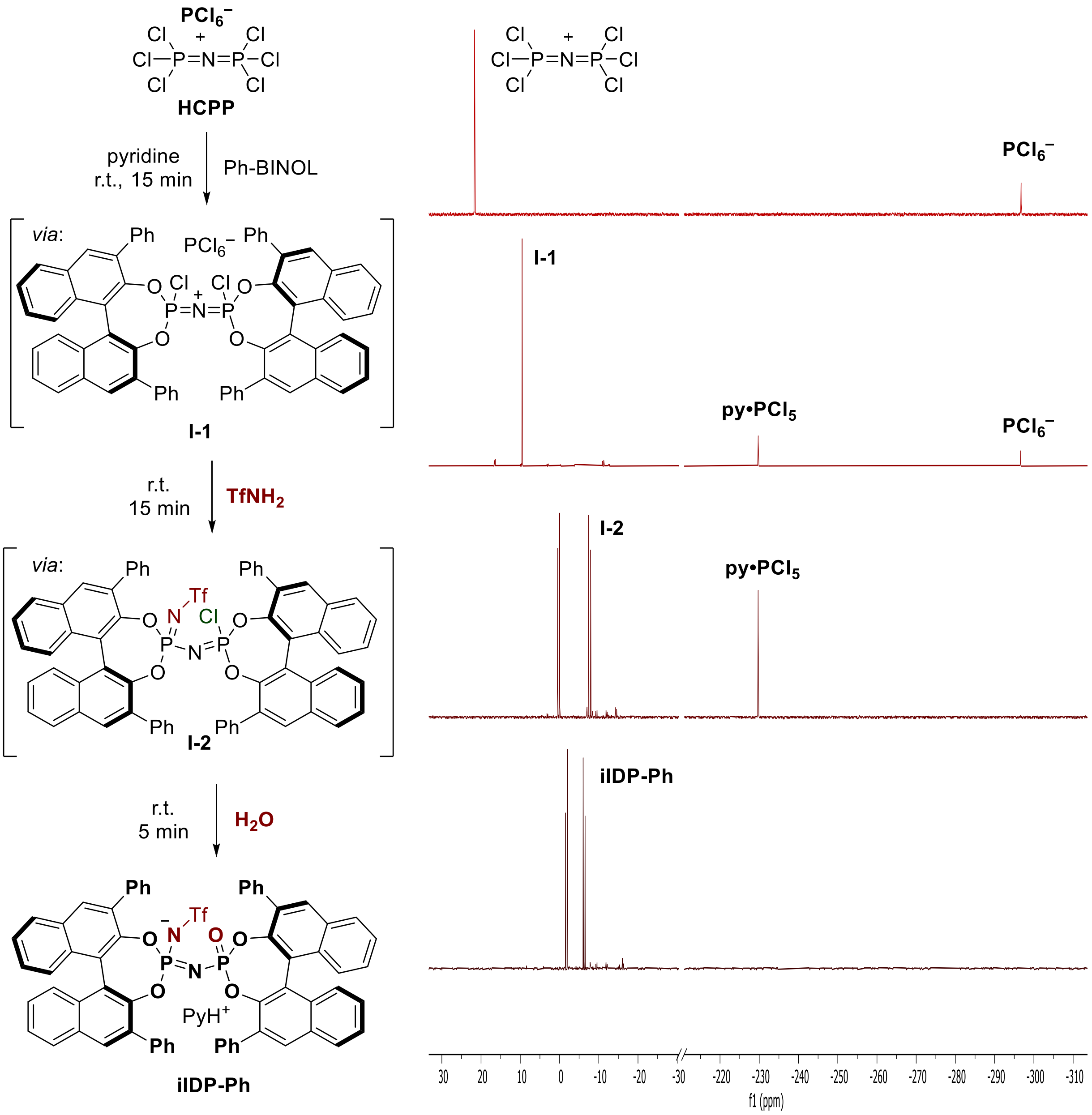

6.1 iIDP-2a

(S,S)-1,1,1-trifluoro-N-(4-((4-oxido-2,6-bis(2,4,6-triisopropylphenyl)dinaphtho[2,1-d:1',2'-f][1,3,2]dioxaphosphepin-4-yl)amino)-2,6-bis(2,4,6-triisopropylphenyl)-4l5-dinaphtho[2,1-d:1',2'-f][1,3,2]dioxaphosphepin-4-ylidene)methanesulfonamide

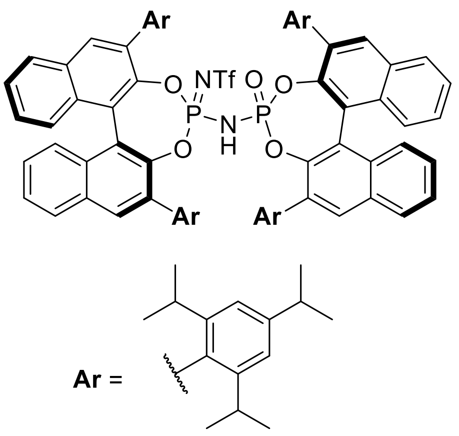

A flame-dried 10 ml Young-Schlenk tube was charged with HCPP (99.0 mg, 186 µmol), *(S)*- 3,3'-di(2,4,6-triisopropylphenyl)-[1,1'-binaphthalene]-2,2'-diol (268 mg, 388 µmol 2.1 equiv.) followed by the addition of toluene (2 ml) and subsequent dropwise addition of NEt_3 (109 µL, 781 µmol, 4.2 equiv.) to form a colorless suspension, which was stirred additional 1.5 h at r.t.. $TfNH_2$ (243 mg, 1.63 mmol, 8.76 equiv.) was added in one portion followed by addition of additional NEt_3 (415 µL, 2.98 mmol, 16 equiv.) and the suspension stirred additional 25 h at 80 °C. 4-DMAP (20.5 mg, 0.167 µmol, 0.9 equiv.) was added and the reaction mixture stirred 17 h at 110 °C. Water (2 ml) and CH_2Cl_2 (2 ml) were added and the emulsion stirred additional 16 h at r.t., followed by isolation of the organic phase, which was dried over sodium sulfate, concentrated to dryness and the resulting crude product was purified by FCC (hexanes / EtOAc 9:1) to elute the desired product as a salt. The salt was dissolved in CH_2Cl_2 (10 ml) and acidified with HCl (6 M, 10 ml) for 30 min at r.t. The organic phase was isolated, concentrated to dryness and dried in high vacuum o.n. to afford the desired product in acidic form (64%, 194 mg, 186 µmol).

^{1}H NMR (501 MHz, CD_2Cl_2) δ=7.92–7.82 (m, 5H), 7.78 (s, 1H), 7.66 (s, 1H), 7.54 (s, 1H), 7.52–7.40 (m, 4H), 7.26 (dddd, *J*=16.7, 8.3, 6.8, 1.3 Hz, 2H), 7.18 (dddd, *J*=20.9, 8.4, 6.8, 1.3 Hz, 2H), 7.12 (d, *J*=1.8 Hz, 1H), 7.08 (d, *J*=1.7 Hz, 1H), 7.02 (d, *J*=8.5 Hz, 1H), 6.98 (d, *J*=1.8 Hz, 1H), 6.95 (d, *J*=1.8 Hz, 1H), 6.93 (d, *J*=1.7 Hz, 1H), 6.90 (d, *J*=8.6 Hz, 1H), 6.88–6.84 (m, 2H), 6.78 (d, *J*=8.7 Hz, 1H), 6.76 (d, *J*=1.8 Hz, 1H), 6.73 (d, *J*=1.7 Hz, 1H), 5.15 (s, 1H), 3.03 (p, *J*=6.6 Hz, 1H), 2.91–2.80 (m, 4H), 2.80–2.73 (m, 2H), 2.63–2.55 (m, 2H), 2.55–2.49 (m, 1H), 2.42 (p, *J*=6.8 Hz, 1H), 2.25 (p, *J*=6.8 Hz, 1H), 1.30 (dd, *J*=6.2 Hz, 6H), 1.27–1.22 (m, 9H), 1.22–1.20 (m, 9H), 1.20–1.16 (m, 9H), 1.08 (d, *J*=6.8 Hz, 3H), 1.04 (d, *J*=6.7 Hz, 3H), 0.94 (d, *J*=6.8 Hz, 3H), 0.92–0.87 (m, 9H), 0.79 (d, *J*=6.8 Hz, 6H), 0.73 (d, *J*=6.8 Hz, 3H), 0.67 (d, *J*=6.8 Hz, 3H), 0.38 (d, *J*=6.8 Hz, 3H), –0.08 (d, *J*=6.8 Hz, 3H), –0.28 ppm (d, *J*=6.8 Hz, 3H). ^{13}C NMR (126 MHz, CD_2Cl_2) δ = 149.4, 149.2, 149.1, 148.7, 148.5, 148.1, 147.8, 147.5, 147.4, 147.2, 147.1, 146.6, 146.1, 146.0, 145.9, 145.8, 145.8, 145.3, 145.2, 134.2, 134.0, 133.7, 133.6, 133.5, 133.2, 133.1, 132.8, 132.8, 132.2, 132.2, 132.0, 132.0, 131.8, 131.8, 131.8, 131.7, 131.6, 131.6, 131.5, 131.3, 131.0, 130.9, 130.7, 128.6, 128.4, 128.1, 127.9, 127.1, 127.1, 127.0, 126.9, 126.8, 126.8, 126.4, 126.3, 126.3, 126.2, 122.8, 122.7, 122.7, 122.6, 122.6, 122.5, 122.5, 122.0, 121.8, 121.5, 121.3, 121.3, 121.2, 120.9, 120.7, 120.7, 120.5, 34.7, 34.6, 34.6, 34.4, 31.5, 31.5, 31.3, 30.9, 30.8, 30.7, 27.9, 26.8, 25.7, 25.3, 25.1, 25.0, 24.9, 24.8, 24.8, 24.7, 24.4, 24.3, 24.2, 24.1, 24.0, 24.0, 23.9, 23.8, 23.6, 23.5, 23.4, 23.0, 22.5, 21.7 ppm (other signals not observed or detected).^{19}F NMR (471 MHz, CD_2Cl_2) δ=–77.67 (s, 3F). ^{31}P NMR (203 MHz, CD_2Cl_2) δ=3.08 (d, *J*=80.5 Hz, 1P), –1.58 (d, *J* = 80.5 Hz, 1P). HRMS

(ESI): *m/z* calcd for $C_{101}H_{112}F_3N_2O_7P_2S_1^-$: 1615.762314 [*M*–H]⁻; found: 1615.762680; $[\alpha]_D^{25}$ = –16 (c = 1 mg/ml in CH_2Cl_2)

6.2 iIDP-2b

(S,S)-1,1,1-trifluoro-N-(4-((4-oxido-2,6-bis(4-((trifluoromethyl)thio)phenyl)dinaphtho[2,1-d:1',2'-f][1,3,2]dioxaphosphepin-4-yl)amino)-2,6-bis(4-((trifluoromethyl)thio)phenyl)-4l5-dinaphtho[2,1-d:1',2'-f][1,3,2]dioxaphosphepin-4-ylidene)methanesulfonamide

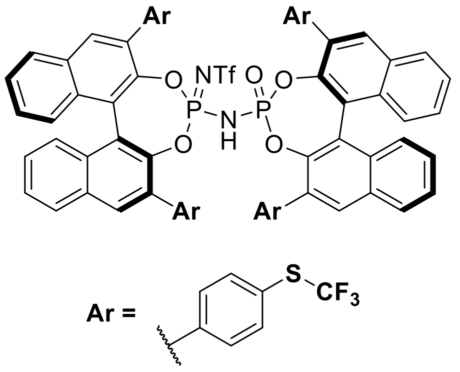

An under high vacuum flame-dried and argonated Schlenk flask was charged with HCPP (50 mg, 94 µmol, 1.0 equiv.), *(S)*-3,3'-bis(4-((trifluoromethyl)thio)phenyl)-BINOL (121 mg, 0.189 mmol, 2.0 equiv.) followed by the addition of pyridine (1 ml) to form a clear yellow solution, which was stirred until full consumption of BINOL (1 h) resulting in the formation of a suspension. Trifluoromethanesulfonamide (68.6 mg, 0.460 mmol, 4.9 equiv.) was added and the reaction mixture stirred additional 16 h at r.t.. Water (0.1 ml) was added and the reaction stirred additional 4 h, at r.t., followed by the addition of HCl (4 N, 10 ml) to quench the reaction. The aqueous phase was extracted with CH_2Cl_2 and the combined organic layers were washed with brine, dried over $NaSO_4$ and concentrated to dryness. The obtained solid was purified by column chromatography (pentane:Et_2O with a gradient of 4:1 to 2:1) to furnish the desired product as a salt, which was acidified by dissolving in a small quantity of CH_2Cl_2 and passing through a pad of DOWEX 50WX-8 to obtain the desired product as a colorless solid in acidic form (70%, 100 mg, 0.094 mmol)

^{1}H NMR (501 MHz, CD_2Cl_2) δ=8.19–8.16 (m, 2H), 8.15 (s, 1H), 8.12 (s, 1H), 8.08 (d, *J*=4.8 Hz, 1H), 8.06 (d, *J*=4.8 Hz, 1H), 7.87–7.79 (m, 2H), 7.71–7.57 (m, 11H), 7.54 (s, 1H), 7.47 (d, *J*=8.5 Hz, 1H), 7.44–7.34 (m, 7H), 7.07 (d, *J*=8.4 Hz, 2H), 7.05 (d, *J*=8.3 Hz, 2H), 6.68 (d, *J*=8.3 Hz, 2H), 6.64 (d, *J*=8.3 Hz, 2H), 4.39 ppm (s). ^{13}C NMR (126 MHz, CD_2Cl_2) δ=144.0, 143.9, 143.6, 143.6, 143.5, 142.7, 142.6, 139.4, 138.9, 138.9, 138.6, 136.6, 136.2, 135.5, 135.3, 132.6, 132.5, 132.3, 132.3, 132.2, 132.2, 132.1, 132.1, 132.0, 131.8, 131.8, 131.5, 131.2, 131.1, 131.1, 130.9, 130.8, 130.8, 130.6, 130.5, 129.2, 129.2, 129.1, 128.8, 128.7, 128.5, 128.4, 128.1, 127.8, 127.7, 127.6, 127.3, 127.2, 127.2, 127.1, 127.1, 126.9, 126.9, 124.5, 124.2, 123.7, 123.7, 123.7, 123.7, 123.6, 123.2, 122.6, 122.6, 122.3, 122.3 ppm. (other signals not detected or observed). ^{19}F NMR (471 MHz, CD_2Cl_2) δ=–42.8 (s, CF_3), –42.9 (s, CF_3), –42.9 (s, CF_3), –43.13 (s, CF_3), –79.96 ppm (s, $2CF_3$). ^{31}P NMR (203 MHz, CD_2Cl_2) δ–2.3 (d, J=113.3 Hz, 1P), –8.3 ppm (d, J=113.4 Hz, 1P). HRMS (ESI) m/z calcd for $C_{69}H_{36}F_{15}N_2O_7P_2S_5^-$ (M–H)⁻ : 1511.0367, found: 1511.0376.; $[\alpha]_D^{25}$ = +308 (c = 1 mg/ml in CH_2Cl_2)

6.3 iIDP-2c

(S,S)-N-(4-((4-oxido-2,6-bis(4-(trifluoromethyl)phenyl)dinaphtho[2,1-d:1',2'-f][1,3,2]dioxaphosphepin-4-yl)amino)-2,6-bis(4-(trifluoromethyl)phenyl)-4l5-dinaphtho[2,1-d:1',2'-f][1,3,2]dioxaphosphepin-4-ylidene)-3,5-bis(trifluoromethyl)benzenesulfonamide

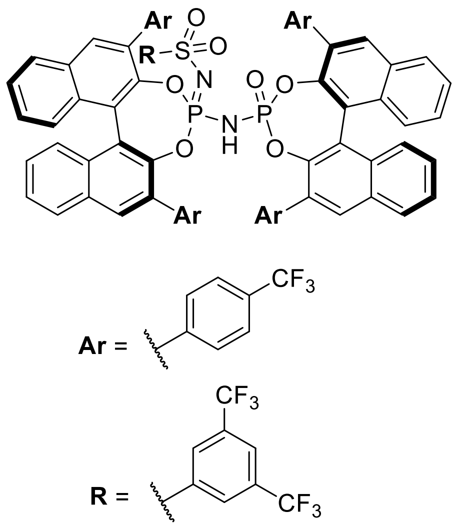

An under high vacuum flame-dried and argonated Schlenk flask was charged with HCPP (67.3 mg, 0.126 mmol,1.0 equiv.), *(S)*-3,3'-bis(4 trifluoromethylphenyl)-BINOL (161 mg, 0.253 mmol, 2.0 equiv.) followed by the addition of pyridine (1 ml) to form a clear yellow solution, which was stirred until full consumption of BINOL (1 h) resulting in the formation of a suspension. 3,5-bis(trifluoromethyl)benzenesulfonamide (94.2 mg, 0.632 mmol, 4.9 equiv.) was added and the reaction mixture stirred additional 16 h at r.t.. Water (0.15 ml) was added and the reaction stirred additional 4 h, at r.t., followed by the addition of an excess of HCl (4 N, 10 ml) to quench the reaction. The aqueous phase was extracted with CH_2Cl_2 and the combined organic layers were washed with brine, dried over $NaSO_4$ and concentrated to dryness. The obtained solid was purified by column chromatography (pentane:Et_2O with a gradient of 4:1 to 2:1) to furnish the desired product as a salt, which was further acidified by dissolving in a small quantitiy of CH_2Cl_2 and passing through a pad of DOWEX 50WX-8 to obtain the desired product as a colorless solid in acidic form (65%, 51.3 mg, 0,094 mmol).

^{1}H NMR (501 MHz, CD_2Cl_2) δ=8.25–8.21 (m, 2H), 8.16 (s, 1H), 8.08 (d, *J*=8.3 Hz, 1H), 8.03 (s, 1H), 8.00 (d, *J*=8.3 Hz, 1H), 7.87 (s, 1H), 7.85–7.77 (m, 5H), 7.75 (s, 1H), 7.69–7.50 (m, 12H), 7.47 (d, *J*=8.5 Hz, 1H), 7.45–7.40 (m, 3H), 7.38 (d, *J*=8.1 Hz, 2H), 7.09 (s), 6.91 (s, 4H), 6.88 (d, *J*=8.2 Hz, 2H), 6.81 ppm (d, *J*=8.1 Hz, 2H). ^{13}C NMR (126 MHz, CD_2Cl_2) δ=145.4, 145.4, 144.3, 144.3, 143.8, 143.8, 143.5, 143.5, 140.3, 140.1, 140.0, 133.1, 133.1, 133.0, 133.0, 132.9, 132.8, 132.5, 132.5, 132.4, 132.3, 132.3, 132.2, 132.1, 131.9, 131.8, 131.7, 131.5, 131.3, 130.3, 130.2, 130.0, 129.9, 129.7, 129.6, 129.5, 129.4, 129.3, 129.2, 129.2, 128.3, 128.1, 127.8, 127.8, 127.6, 127.5, 127.4, 127.4, 127.1, 127.1, 127.0, 126.9, 126.7, 126.7, 126.0, 126.0, 125.8, 125.3, 125.3, 125.2, 125.2, 125.2, 125.1, 125.1, 124.9, 124.9, 124.9, 124.8, 124.8, 124.1, 124.1, 124.1, 124.0, 123.6, 122.9, 122.9, 122.7, 121.9 ppm. (other signals not detected or observed). ^{19}F NMR (471 MHz, CD_2Cl_2) δ=–62.63 (s, CF_3), –62.72 (s, CF_3), –62.88 (s, CF_3), –63.04 (s, CF_3), –63.12 ppm (s, 2CF_3). ^{31}P NMR (203 MHz, CD_2Cl_2) δ=0.3 (d, *J*=103.5 Hz), –4.75 ppm (d, *J*=103.5 Hz). HRMS (ESI) m/z calculated for $C_{76}H_{39}F_{18}N_2O_7P_2S_1^-$ (M–H)$^-$: 1527.1671, found: 1527.1676; $[\alpha]_D^{25}$ = +278 (c = 1 mg/ml in CH_2Cl_2)

6.4 iIDP-2d

(S,S)-1,1,1-trifluoro-N-(4-((4-oxido-2,6-bis(4-(pentafluoro-l6-sulfanyl)phenyl)dinaphtho[2,1-d:1',2'-f][1,3,2]dioxaphosphepin-4-yl)amino)-2,6-bis(4-(pentafluoro-l6-sulfanyl)phenyl)-4l5-dinaphtho[2,1-d:1',2'-f][1,3,2]dioxaphosphepin-4-ylidene)methanesulfonamide

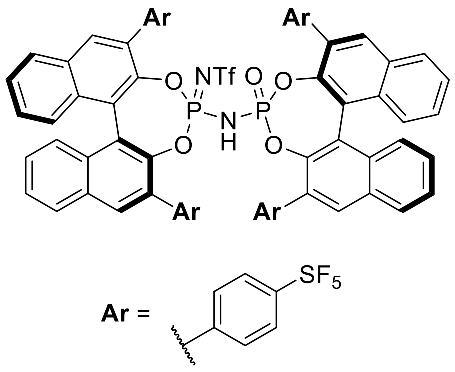

An under high vacuum flame-dried and argonated Schlenk flask was charged with HCPP (57 mg, 107 µmol, 1.0 equiv.), *(S)*- 3,3'-bis(4-(pentafluorsulfanyll)phenyl))-BINOL (147 mg, 213 µmol, 2.0 equiv.) followed by the addition of pyridine (1 ml) to form a clear yellow solution, which was stirred until full consumption of BINOL (1 h) resulting in the formation of a suspension. Trifluormethanesulfonamide (79.5 mg, 534 µmol, 5 equiv.) was added and the reaction mixture stirred additional 16 h at r.t.. Water (0.1 ml) was added and the reaction stirred additional 4 h, at r.t., followed by the addition of an excess of HCl (4 N, 10 ml) to quench the reaction. The aqueous phase was extracted with CH_2Cl_2 and the combined organic layers were washed with brine, dried over Na_2SO_4 and concentrated to dryness. The obtained solid was purified by column chromatography (Hexane:EtOAc with a gradient of 10:1 to 5:1) to furnish the desired product as a salt which, was acidified by dissolving in a small quantitiy of $CH_2Cl_2$and passing through a pad of DOWEX 50WX-8 to obtain the desired product as a colorless solid in acidic form (71%, 123 mg, 0,076 mmol).

^{1}H NMR (501 MHz, CD_2Cl_2) δ=8.26–8.22 (m, 2H), 8.17 (s, 1H), 8.14–8.03 (m, 3H), 7.90–7.80 (m, 2H), 7.80–7.75 (m, 3H), 7.74–7.58 (m, 9H), 7.55–7.48 (m, 3H), 7.47–7.37 (m, 5H), 7.12 (d, *J*=8.6 Hz, 2H), 7.03 (d, *J*=8.6 Hz, 2H), 6.73 (d, *J*=8.5 Hz, 2H), 6.67 (d, *J*=8.4 Hz, 2H), 6.21 ppm (s, 1H), 2.58 (s, 1H). ^{13}C NMR (126 MHz, CD_2Cl_2) δ=153.7, 153.4, 153.2, 153.1, 144.4, 144.3, 144.0, 143.9, 143.0, 133.2, 133.0, 133.0, 132.7, 132.7, 132.5, 132.5, 132.4, 132.4, 132.3, 132.1, 132.1, 131.9, 131.6, 131.6, 131.4, 130.9, 130.3, 130.2, 130.1, 129.6, 129.6, 129.5, 129.4, 128.8, 128.5, 128.2, 128.2, 128.0, 127.8, 127.6, 127.4, 127.3, 127.3, 127.2, 126.7, 126.7, 126.0, 125.8, 125.5, 124.3, 124.2, 122.9, 122.9, 122.6 ppm. (other signals not detected or observed) ^{19}F NMR (471 MHz, CD_2Cl_2) δ=85.42–83.27 (m, 4F), 63.41–62.27 (m, 16F), –79.99 (s, 3F). ^{31}P NMR (203 MHz, CD_2Cl_2) δ=–1.2 (d, *J*=105.2 Hz, 1P), –6.2 (d, *J*=105.4 Hz, 1P). HRMS (ESI) m/z calculated for $C_{65}H_{36}F_{23}N_2O_7P_2S_5^-$ $(M–H)^-$: 1615.0240, found: 1615.0249; $[\alpha]_D^{25}$ = +304 (c = 1 mg/ml in CH_2Cl_2)

6.5 iIDP-2e

(*S*,*S*)-N-(4-((2,6-bis(2-cyclohexyl-5-methylphenyl)-4-oxidodinaphtho[2,1-d:1',2'-f][1,3,2]dioxaphosphepin-4-yl)amino)-2,6-bis(2-cyclohexyl-5-methylphenyl)-4l5-dinaphtho[2,1-d:1',2'-f][1,3,2]dioxaphosphepin-4-ylidene)-1,1,1-trifluoromethanesulfonamide

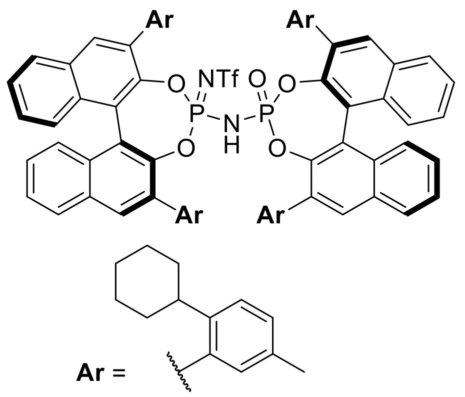

A 25 ml flame dried Schlenk-tube was charged with HCPP (265 mg, 498 µmol, 1 equiv.), 3,3'-bis(2-cyclohexyl-5-methylphenyl)-[1,1'-binaphthalene]-2,2'-diol (628 mg, 996 µmol, 2 equiv.) followed by addition of pyridine (10 ml) to form a yellowish solution. The reaction was stirred 50 min at r.t., followed by addition of $TfNH_2$ (371 mg, 2.49 mmol (5 equiv.) and stirred 1 h at r.t.. Water (1 ml, 55.5 mmol, 112 equiv.) was added and the resulting suspension stirred 6 h at r.t.. The reaction mixture was poured into ice-cooled $HCl_{(aq)}$ (6 M, 100 ml), transferred to a separation funnel and the aqueous phase extracted with DCM (3 x 40 ml). The combined organic phase was dried over sodium sulfate, concentrated to dryness and the crude product purified by FCC (DCM / EtOAc 4:1) to elute the desired product as salt. The salt was dissolved in a small quantitiy of DCM (ca. 4 ml), overlayered with pentane and stand for 3 d in a freezer (–20 °C) to form colorless crystals. The organic phase was decanted and the colorless crystals dissolved in 20 ml DCM followed by addition of HCl (6 M, 20 ml) and stirred for 30 min at r.t.. The DCM phase was isolated, concentrated to dryness and further dried in h.v o.n. to afford the desired product in acidic form as a colorless solid (67 %, 503 mg, 498 µmol). [NMR analysis shows two sets of signals, which indicate the presence of rotamers (ratio 9:1). The main signals are listed below]

^{1}H NMR (501 MHz, CD_2Cl_2) δ = 7.87 – 7.80 (m, 4H), 7.80 – 7.74 (m, 2H), 7.57 (ddd, *J*=19.1, 11.3, 7.6, 4H), 7.37 (dt, *J*=8.7, 7.3, 4H), 7.27 – 7.16 (m, 2H), 7.13 (ddd, *J*=12.3, 7.3, 2.7, 2H), 7.03 (dd, *J*=13.2, 8.4, 2H), 6.94 – 6.83 (m, 7H), 6.78 (s, 1H), 6.51 (s, 1H), 6.29 (d, *J*=2.0, 1H), 5.98 (d, *J*=1.9, 1H), 5.97 – 5.89 (sbr, 1H), 5.66 (d, *J*=2.0, 1H), 2.73 – 2.66 (m, 1H), 2.05 (s, 3H), 1.87 (s, 3H), 1.83 (s, 2H), 1.76 – 1.67 (m, 5H),1.52 (t, *J*=10.3, 4H), 1.43 (d, *J*=18.4, 6H), 1.37 (td, *J*=12.1, 11.2, 2.8, 2H), 1.31 – 1.06 (m, 14H), 1.04 – 0.94 (m, 3H), 0.92 – 0.50 (m, 11H), 0.43 – 0.29 (m, 1H), 0.07 – -0.06 ppm (m, 1H).

^{13}C NMR (126 MHz, CD_2Cl_2) δ 145.64, 145.53, 145.35, 145.27, 144.88, 144.80, 144.06, 144.00, 143.94, 143.76, 143.58, 143.47, 135.37, 135.26, 134.78, 134.72, 134.30, 134.22, 134.15, 133.37, 133.10, 132.93, 132.78, 132.74, 132.72, 132.57, 132.17, 132.14, 131.98, 131.93, 131.78, 131.63, 131.58, 131.41, 131.09, 130.23, 129.83, 129.58, 129.51, 129.31, 129.07, 128.98, 128.22, 127.54, 127.46, 127.17, 127.04, 126.99, 126.96, 126.63, 126.55, 126.43, 126.22, 125.12, 122.74, 122.44, 122.44, 121.80, 121.73, 54.27, 54.06, 53.84, 53.62, 53.41, 41.38, 41.29, 41.07, 40.03, 37.56, 37.07, 35.99, 35.67, 33.55, 33.50, 33.18, 31.98, 31.01, 27.65, 27.56, 27.48, 27.42, 27.13, 27.10, 26.92, 26.77, 26.56, 26.51, 26.32, 21.66, 21.46, 20.79, 20.57 ppm. (other signals not detected or observed. ^{19}F NMR (471 MHz, CD_2Cl_2) δ = –80.32 ppm (s, 3F). ^{31}P NMR (203 MHz, CD_2Cl_2) δ = –3.79 (d, *J*=117.4), –8.44 ppm (d, *J*=117.4). HRMS (ESI): *m*/*z* calcd for $C_{93}H_{88}F_3N_2O_7P_2S_1^-$: 1495.574514 [*M*–H]$^-$; found: 1495.574410; $[\alpha]_D^{25}$ = +130 (c = 1 mg/ml in CH_2Cl_2)

7. Synthesis of Imidodiphosphorimidates (IDPi)

7.0 Initial NMR experiments

A 10 ml schlenk tube was charged with HCPP (160 mg, 300 µmol, 1 equiv.), (*S*)-3,3´-(phenyl)BINOL (268 mg, 611 µmol, 2 equiv.) followed by the addition of pyridine (3 ml). The reaction was stirred 15 minutes at r.t.. An aliquote (300 µl) was transferred to an NMR tube under inert conditions, diluted with anhydrous CD_2Cl_2 (300 µl) followed by NMR analysis. Trifluoromethanesulfonamide (358 mg, 2.40 mmol, 8 equiv.) was added and the reaction stirred for additional 15 min at r.t.. An aliquote (300 µl) was transferred to an NMR tube under inert conditions, diluted with anhydrous CD_2Cl_2 (300 µl) followed by NMR analysis. The reaction was stirred 3 d at 90°C. An aliquote (300 µl) was transferred to an NMR tube under inert conditions, diluted with anhydrous CD_2Cl_2 (300 µl) followed by NMR analysis.

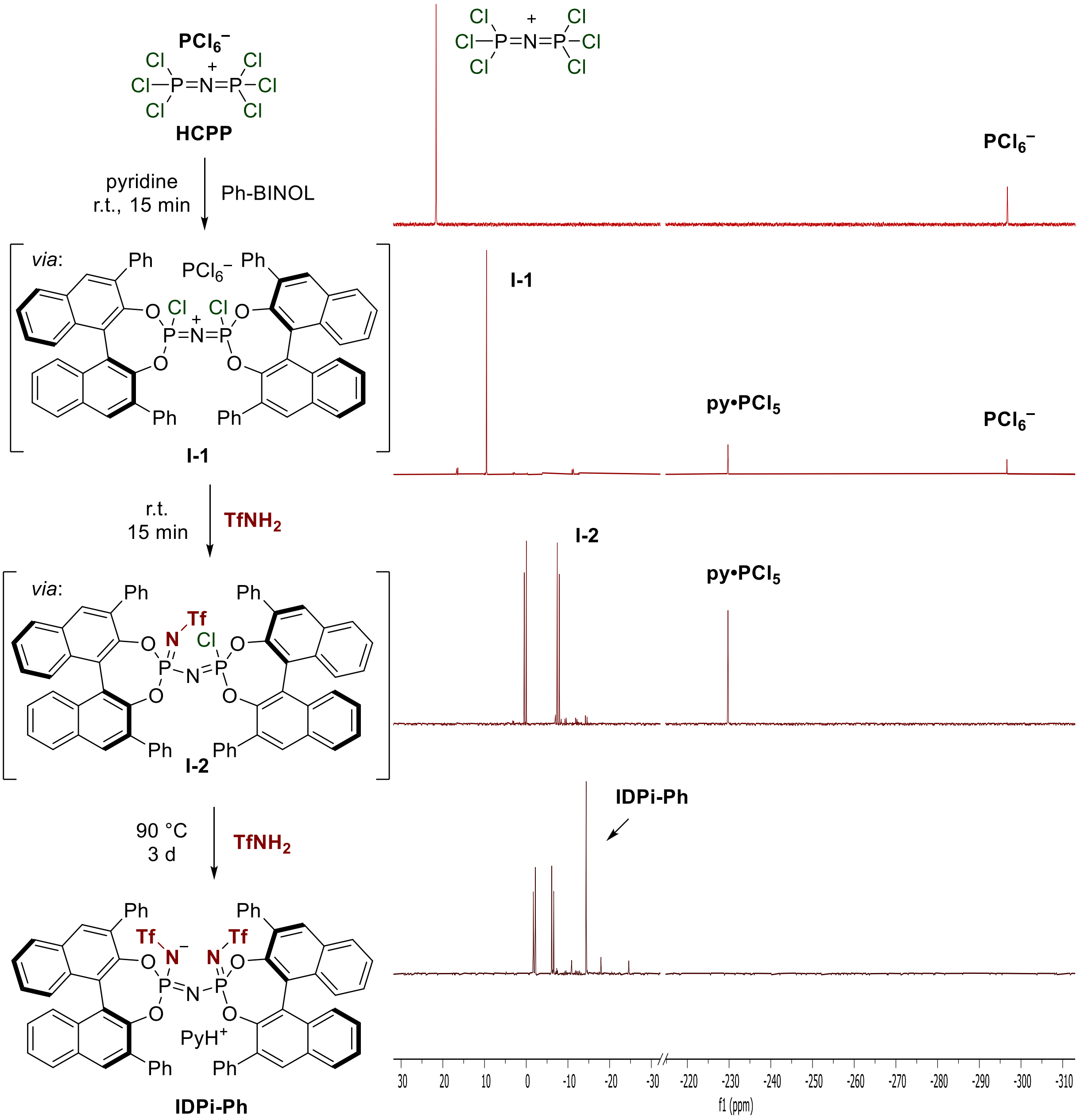

7.1. IDPi-3a

(S,S)-N,N'-(azanediylbis(2,6-dimesityl-4l5-dinaphtho[2,1-d:1',2'-f][1,3,2]dioxaphosphepine-4-yl-4-ylidene))bis(1,1,1-trifluoromethanesulfonamide)

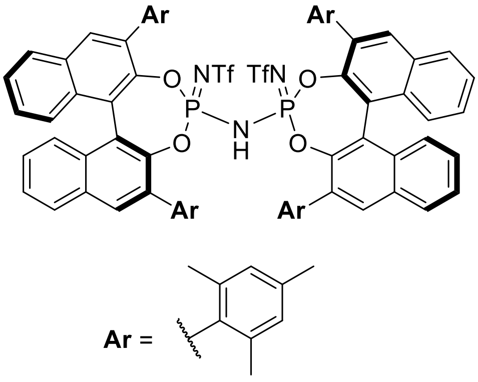

A 10 ml flame-dried Schlenk tube was charged with phosphazene (129 mg, 0.24 mmol, 1 equiv.), *(S)*-3,3´-bis(mesityl)-BINOL (271 mg, 0.52 mmol, 2.1 equiv.) and suspended in toluene (5 ml). Triethylamine (150 µl, 1,09 mmol, 4.5 equiv.) was added dropwise via a Hamilton syringe under vigorous stirring forming a slightly orange suspension which faded away within 5 minutes at r.t.. The resulting colorless suspension was stirred additional 60 minutes at r.t. followed by the addition of $TfNH_2$ (289 mg, 1.94 mmol, 8 equiv.) additional triethylamine (0.54 ml, 3.88 mmol, 16 equiv.) and stirred 26 h at 80 °C. The orange suspension was cooled to r.t. followed by addition of 4-dimethylaminopyridine (26.6 mg, 220 µmol, 0.9 equiv.) and stirred for additional 6 d at 100 °C. The reaction mixture was then analyzed by NMR (a small aliquote was transferred to an NMR tube under inert conditions, all volatiles were removed in high vacuum followed by the addition of CD_2Cl_2) showing a conversion of approximately 60% to the desired product. The reaction mixture was then quenched with ca. 1 ml aqueous HCl (6 M) followed by dilution with dichloromethane (ca. 10 ml). The organic phase was washed with HCl (6 M, 10 ml) followed by washing with sat. $NaHCO_{3(aq)}$ (2x10ml). The organic phase was dried over sodium sulfate, concentrated to dryness followed by FCC (Biotage, gradient: n-hexane/EtOAc (100/0) up to (60/40) to elute the desired product as salt (m = 126 mg, 44 % yield referred to the sodium salt). The corresponding intermediate **I-2** was isolated and stored for further transformations (114 mg, 36 %). 46 mg of the salt was dissolved in a small quantitiy of dichloromethane and passed through a Pasteur pipette filled (ca 3 cm height) with Dowex40W-X8 (acidic form) to elute the desired product in acidic form (m = 42 mg, 90 % yield).

^{1}H NMR (501 MHz, CD_2Cl_2) δ = 8.0 – 7.9 (m, 2H), 7.9 – 7.8 (m, 2H), 7.8 (s, 2H), 7.6 (ddd, *J*=8.2, 6.8, 1.3, 2H), 7.5 – 7.5 (m, 4H), 7.4 (ddd, *J*=8.4, 6.9, 1.4, 2H), 7.4 – 7.3 (m, 2H), 7.2 (ddd, *J*=8.4, 6.8, 1.4, 2H), 7.1 (d, *J*=8.0, 2H), 6.9 (s, 2H), 6.8 (s, 2H), 6.7 (s, 2H), 6.4 (s, 2H), 4.5 (s, 1H), 2.2 (s, 6H), 2.2 (s, 6H), 2.2 (s, 6H), 1.9 (s, 6H), 1.8 (s, 6H), 0.9 (s, 6) ppm. ^{13}C NMR (126 MHz, CD_2Cl_2) δ 145.1, 145.1, 145.1, 144.7, 144.7, 144.6, 138.0, 137.4, 137.32 137.1, 136.9, 135.6, 134.2, 133.2, 132.9, 132.8, 132.6, 132.4, 132.3, 132.3, 132.3, 129.2, 128.8, 128.6, 128.5, 128.2, 128.2, 127.4, 127.4, 127.2, 127.1, 126.7, 126.7, 122.4, 122.2, 21.7, 21.4, 21.0, 20.6, 19.8, 19.5 ppm (other signals not detected or observed). ^{19}F NMR (471 MHz, CD_2Cl_2) δ=–78.71 ppm (s, 2CF_3); ^{31}P NMR (203 MHz, CD_2Cl_2) δ –10.65 ppm (s); HRMS: calcd for $C_{78}H_{64}F_6N_3O_8P_2S_2^-$ [M–H]–: 1410.351986; found: 1410.351340; $[\alpha]_D^{25}$ = +20 (c = 0.25 mg/ml in CH_2Cl_2)

7.1.1 Intermediate I-2

(S,S)-N-(4-((4-chloro-2,6-dimesityl-4l5-dinaphtho[2,1-d:1',2'-f][1,3,2]dioxaphosphepin-4-ylidene)amino)-2,6-dimesityl-4l5-dinaphtho[2,1-d:1',2'-f][1,3,2]dioxaphosphepin-4-ylidene)-1,1,1-trifluoromethanesulfonamide

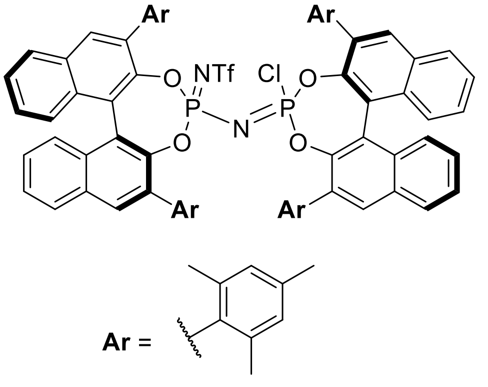

^{1}H NMR (600 MHz, CD_2Cl_2) δ = 8.0 – 7.9 (m, 2H), 7.9 (ddd, *J*=8.2, 2.0, 1.0, 2H), 7.9 (t, *J*=1.1, 1H), 7.8 (d, *J*=1.0, 1H), 7.6 – 7.6 (m, 1H), 7.6 – 7.5 (m, 2H), 7.5 – 7.5 (m, 3H), 7.5 – 7.4 (m, 3H), 7.4 – 7.3 (m, 1H), 7.3 – 7.2 (m, 2H), 7.2 – 7.2 (m, 1H), 7.0 (d, *J*=8.5, 1H), 6.9 – 6.9 (m, 1H), 6.9 – 6.9 (m, 1H), 6.8 – 6.8 (m, 2H), 6.8 – 6.8 (m, 1H), 6.7 – 6.6 (m, 1H), 6.3 (d, *J*=1.3, 1H), 6.2 – 6.2 (m, 1H), 2.2 (s, 3H), 2.2 (s, 3H), 2.2 (s, 3H), 2.1 (s, 3H), 2.1 (s, 5H), 1.9 (s, 3H), 1.8 (s, 3H), 1.8 (s, 3H), 1.6 (s, 3H), 0.8 (s, 3H), 0.7 (s, 3H) ppm. ^{13}C NMR (151 MHz, CD_2Cl_2) δ = 145.9, 145.8, 145.2, 145.2, 145.2, 145.0, 144.9, 138.4, 138.2, 137.9, 137.7, 137.5, 137.3, 137.2, 137.2, 136.9, 136.2, 135.8, 135.6, 134.3, 133.3, 133.3, 133.2, 133.1, 133.0, 132.9, 132.8, 132.7, 132.5, 132.4, 132.3, 132.2, 132.2, 132.2, 132.1, 132.0, 131.8, 129.2, 129.0, 128.8, 128.7, 128.6, 128.6, 128.5, 128.2, 128.1, 128.0, 127.7, 127.6, 127.4, 127.3, 127.2, 127.2, 127.1, 126.9, 126.9, 126.7, 126.3, 126.2, 122.3, 122.2, 122.1, 30.1, 21.9, 21.5, 21.3, 21.2, 21.1, 21.0, 20.3, 20.1, 19.9, 19.8, 19.5, 19.4 (other signals not detected or observed). ^{19}F NMR (565 MHz, CD_2Cl_2) δ = –80.0 ppm (s, CF_3). ^{31}P NMR (243 MHz, CD_2Cl_2) δ = 8.0 (d, *J*=79.3, 1P), –1.9 (d, *J*=78.5, 1P) ppm. HRMS: calculated for $C_{77}H_{64}Cl_1F_3N_2O_6P_2S_1Na_1^+$ ([M + Na]$^+$): 1321.348650; found: 1321.349321

7.2 IDPi-3b

(S,S)-N,N'-(azanediylbis(2,6-bis(2,4,6-triethylphenyl)-4l5-dinaphtho[2,1-d:1',2'-f][1,3,2]dioxaphosphepine-4-yl-4-ylidene))bis(1,1,1-trifluoromethanesulfonamide)

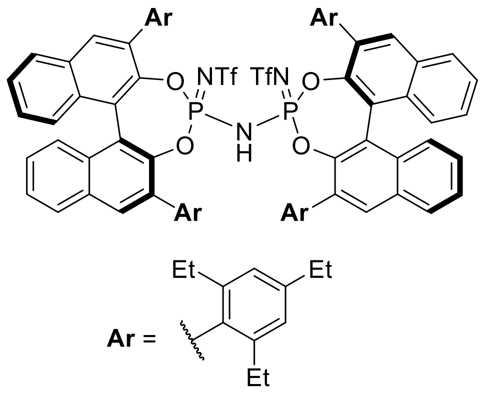

A 10 ml flame-dried and argonated Schlenk tube was charged with HCPP (45 mg, 0,085 mmol, 1.0 equiv.) and *(S)*-3,3´-(*o,o,p*-tri(n-ethyl)phenyl)BINOL (110 mg, 0.18 mmol, 2.2 equiv.) and dissolved in 1 ml toluene. To the solution was added NEt_3 (53 µl, 0.38 mmol, 4.5 equiv.) dropwise under vigorous stirring. The resulting yellow suspension was stirred for 60 min at r.t. followed by the addition of H_2NTf (100 mg, 0.68 mmol, 8 equiv.) and NEt_3 (195 µl, 137 mg, 1.35 mmol, 16,0 equiv.). The mixture was heated to 80 °C under stirring for 15 h and subsequently 4-DMAP (9.3 mg, 0.076 mmol, 0.9 equiv.) was added at r.t.. The reaction was stirred for 120 h at 120 °C. The crude mixture was purified without work-up by FCC (Biotage, gradient: hexanes up to hexanes / EtOAc (5:1)) to elute the desired product as a salt. Acidification was carried out by dissolving the salt in Et_2O (3 ml) and flushing the solution through a 5 cm pad of DOWEX® 50WX2. The organic phase was concentrated to dryness furnish the desired product as a colorless solid (36%, 48 mg)

^{1}H NMR (600 MHz, CD_2Cl_2) δ = 7.9 (dd, *J*=18.3, 8.3, 4H), 7.8 (s, 2H), 7.6 (s, 2H), 7.5 (t, *J*=7.6, 2H), 7.5 (t, *J*=7.5, 2H), 7.4 (t, *J*=7.8, 2H), 7.3 (d, *J*=8.4, 2H), 7.2 (t, *J*=7.7, 2H), 7.0 (s, 2H), 6.9 (s, 1H), 6.9 (s, 3H), 6.8 (s, 2H), 6.5 (s, 2H), 2.7 – 2.6 (m, 6H), 2.6 – 2.5 (m, 6H), 2.4 – 2.2 (m, 6H), 2.2 – 2.1 (m, 2H), 2.1 – 2.0 (m, 2H), 1.7 – 1.6 (m, 2H), 1.2 (t, *J*=7.6, 6H), 1.2 (t, *J*=7.6, 6H), 1.2 (t, *J*=7.4, 6H), 0.9 (t, *J*=7.5, 6H), 0.8 (t, *J*=7.5, 6H), 0.0 (t, *J*=7.6, 6H) ppm. ^{13}C NMR (151 MHz, CD_2Cl_2) δ = 144.9, 144.7, 144.6, 144.5, 144.0, 143.4, 143.0, 142.7, 142.4, 134.7, 133.2, 132.5, 132.0, 131.8, 131.7, 131.7, 128.7, 128.7, 127.5, 127.2, 127.1, 126.8, 126.7, 126.2, 126.1, 125.8, 124.8, 122.5, 122.0, 119.9, 117.8, 30.1, 29.1, 28.7, 28.1, 27.5, 27.3, 26.8, 16.4, 16.2, 15.9, 15.5, 15.0, 14.4 ppm (other signals not observed or detected). ^{19}F NMR (565 MHz, CD_2Cl_2) δ = –78.4 ppm (2 CF_3). ^{31}P NMR (243 MHz, CD_2Cl_2) δ = –10.2 (s, 2P). HRMS (ESI): m/z calcd for $C_{90}H_{88}F_3N_3O_8P_2S_2^-$: 1578.539786 [M-H]$^-$, found 1578.540540; $[\alpha]_D^{25}$ = +18 (c = 1 mg/ml in CH_2Cl_2)

7.3 IDPi-3c

(S,S)-N-(4-((2,6-bis(4-(tert-butyl)phenyl)-4-((4-methylphenyl)sulfonamido)-4l5-dinaphtho[2,1-d:1',2'-f][1,3,2]dioxaphosphepin-4-ylidene)amino)-2,6-bis(4-(tert-butyl)phenyl)-4l5-dinaphtho[2,1-d:1',2'-f][1,3,2]dioxaphosphepin-4-ylidene)-4-methylbenzenesulfonamide

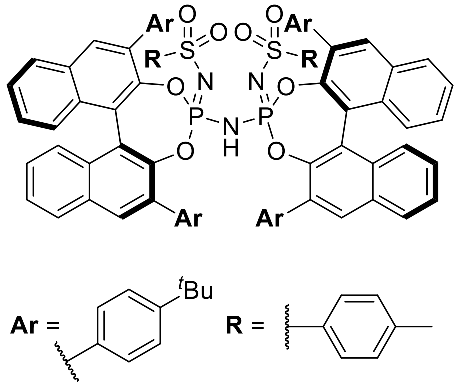

A 10 ml flame-dried and argonated Schlenk tube was charged with HCPP (30 mg, 56 µmol, 1.0 equiv.) and *(S)*-3,3´-di(*p*-^{t}Bu-phenyl)BINOL (64 mg, 116 µmol, 2.05 equiv.) and dissolved in 0.8 ml toluene. To the solution was added NEt_3 (35 µl, 254 µmol, 4.5 equiv.) dropwise under vigorous stirring. The resulting yellow suspension was stirred for 60 min at r.t. followed by the addition of 4-methylbenzenesulfonamide (77.2 mg, 451 µmol, 8.0 equiv.) and NEt_3 (126 µl, 91.2 mg, 902 µmol, 16.0 equiv.). The mixture was heated to 110 °C under stirring for 120 h. The crude mixture was purified without work-up by FCC (Biotage, hexanes / EtOAc (5:1)) to elute the desired product as a salt. Acidification was carried out by dissolving the salt in Et_2O (3 ml) and flushing the solution through a 5 cm pad of DOWEX® 50WX2. The organic phase was concentrated to dryness to furnish the desired product as a colorless solid (21%, 18 mg)

^{1}H NMR (501 MHz, CD_2Cl_2) δ = 8.1 (d, *J*=8.0, 2H), 7.9 (d, *J*=7.9, 2H), 7.9 (s, 2H), 7.7 (t, *J*=7.3, 2H), 7.6 (s, 2H), 7.5 – 7.5 (m, 4H), 7.5 – 7.4 (m, 2H), 7.4 – 7.3 (m, 12H), 7.2 – 7.2 (m, 4H), 6.9 – 6.8 (m, 4H), 6.7 – 6.7 (m, 4H), 6.6 – 6.5 (m, 4H), 2.3 (sbr, 1H), 2.2 (s, 6H), 1.2 (s, 18H), 1.0 (s, 18H) ppm. ^{13}C NMR (126 MHz, CD_2Cl_2) δ = 150.9, 150.4, 144.7, 142.7, 134.5, 133.6, 133.5, 133.2, 132.5, 132.3, 132.3, 132.0, 131.2, 131.2, 129.8, 129.2, 129.1, 128.9, 127.7, 127.3, 126.9, 126.8, 126.7, 126.3, 126.0, 125.8, 124.9, 124.0, 122.6, 34.7, 34.6, 31.3, 31.3 ppm (other signals not observed or detected). ^{31}P NMR (203 MHz, CD_2Cl_2) δ = –8.9 ppm (s, 2P). HRMS (ESI): m/z cald for $C_{94}H_{86}N_3O_8P_2S_2^-$: 1510.533715 [M-H]$^-$, found 1510.533940; $[\alpha]_D^{25}$ = +60 (c = 0.04 mg/ml in CH_2Cl_2)

7.4 IDPi-3d

(*S*,*S*)-N,N'-(azanediylbis(2,6-bis(2'-(tert-butyl)spiro[cyclopentane-1,9'-fluoren]-7'-yl)-4l5-dinaphtho[2,1-d:1',2'-f][1,3,2]dioxaphosphepine-4-yl-4-ylidene))bis(1,1,1-trifluoromethanesulfonamide)

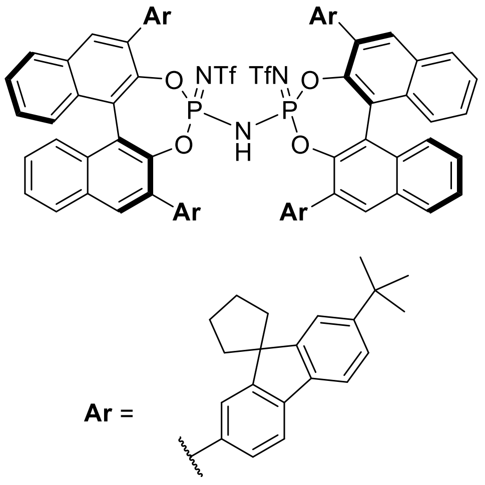

10 ml flame-dried and argonated Schlenk tube was charged with HCPP (30 mg, 56 µmol, 1.0 equiv.) and (*S*)-3,3'-bis(2'-(tert-butyl)spiro[cyclopentane-1,9'-fluoren]-7'-yl)-BINOL (64 mg, 116 µmol, 2.05 equiv.) and dissolved in 0.8 ml toluene. To the solution was added NEt_3 (35 µl, 254 µmol, 4.5 equiv.) dropwise under vigorous stirring. The resulting yellow suspension was stirred for 60 min at r.t. followed by the addition of H_2NTf (327 mg, 2.19 mmol, 8 equiv.). The mixture was heated to 120 °C under stirring for 36h. The crude mixture was purified without work-up by FCC (gradient: hexanes / EtOAc 1:0 to 9:1) to elute the desired product as a salt. Acidification was carried out by dissolving the salt in Et_2O (3 ml) and flushing the solution through a 5 cm pad of DOWEX® 50WX2. The organic phase was concentrated to dryness to furnish the desired product as a colorless solid (70%, 392 mg)

^1H NMR (501 MHz, CD_2Cl_2) δ = 8.94 (sbr, 1H), 8.11 (s, 2H), 8.08 (dd, *J*=8.1, 6.2, 4H), 7.86 (ddd, *J*=8.2, 6.8, 1.3, 2H), 7.79 (d, *J*=7.9, 2H), 7.69 (ddd, *J*=8.4, 6.8, 1.3, 2H), 7.61 (ddd, *J*=8.2, 6.1, 1.9, 2H), 7.52 (d, *J*=1.9, 2H), 7.47 (d, *J*=8.4, 4H), 7.41 (dd, *J*=8.0, 1.9, 4H), 7.35 (dd, *J*=4.8, 3.1, 4H), 7.30 – 7.22 (m, 8H), 7.11 (s, 2H), 6.62 (d, *J*=8.0, 2H), 6.49 (dd, *J*=8.0, 1.7, 2H), 6.26 – 6.21 (m, 2H), 2.22 – 2.14 (m, 2H), 2.12 – 1.92 (m, 22H), 1.88 – 1.81 (m, 2H), 1.81 – 1.71 (m, 4H), 1.71 – 1.63 (m, 2H), 1.32 (s, 18H), 1.31 ppm (s, 18H). ^{13}C NMR (126 MHz, CD_2Cl_2) δ 154.81, 154.67, 154.46, 154.00, 151.32, 150.72, 143.81, 142.79, 139.60, 138.91, 136.19, 135.99, 134.66, 134.43, 133.91, 133.86, 132.15, 131.74, 131.52, 131.30, 129.11, 128.64, 128.32, 127.41, 126.81, 126.77, 126.73, 126.48, 123.91, 123.73, 123.57, 123.26, 121.83, 119.65, 119.52, 119.16, 118.58, 118.43, 118.06, 57.91, 57.81, 40.03, 39.99, 39.11, 38.91, 34.84, 34.77, 31.30, 30.57, 26.97, 26.74, 26.54, 26.37 ppm (other signals not observed or detected). ^{19}F NMR (471 MHz, CD_2Cl_2) δ = –78.75 ppm (s, 6F). ^{31}P NMR (203 MHz, CD_2Cl_2) δ = –17.10 ppm (s, 2P). HRMS (ESI): m/z cald for $C_{94}H_{86}N_3O_8P_2S_2^-$: 2034.707586 [M-H]$^-$, found 2034.727460; $[\alpha]_D^{25}$ = +198 (c = 1.2 mg/ml in CH_2Cl_2)

8. Synthesis of phenylbis(trifluoromethylsulfonylimino)sulfonamide 6

8.1 Sodium phenylbis(trifluoromethylsulfonylimino)sulfinate 5

sodium-(S-phenyl-N-((trifluoromethyl)sulfonyl)sulfinimidoyl)((trifluoromethyl)sulfonyl)amide

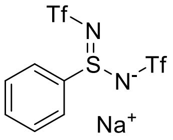

A 50 ml schlenk tube was charged with an aqueous solution of $TfNH_2$ (2.50 g, 16.8 mmol) and NaOH (2.50 g, 62.5 mmol, 20 ml H_2O) and cooled to –20 °C (ethanol, dry ice). Chlorine gas was bubbled through the solution maintaining the temperature between –20 to –10°C for 15 minutes to form a slightly yellowish emulsion, which forms two phases upon standing. The emulsion was carefully transferred to a pre-cooled 50 ml dropping funnel, connected to a 10 ml schlenk-tube, which is charged with a solution of thiophenol (PhSH 550 mg, 5 mmol) in 5 ml anhydrous dichloromethane and cooled to –30 °C. The lower yellowish phase (containing $TfNCl_2$ of the dropping funnel was slowly added to the cooled dichloromethane solution (highly exothermic, few drops in a 10 second intervall). The dichloromethane solution was stirred 1 h at r.t. and quenched with water. The organic phase was isolated, and concentrated to dryness to obtain a highly viscous oil. This oil was dissolved in a small quantity of dichloromethane and loaded on silica followed by FCC (gradient: 1. pure dichloromethane to remove TfNH2 impurites followed by DCM/acetone (3/2) to elute the desired product which was isolated as a colorless solid. The solid was dissolved in diethylether, washed with sat. $NaHCO_{3(aq)}$, the organic phase dried over sodium sulfate and concentrated to dryness to afford the desired product as a beige solid (74%, 1.50 g, 3,71 mmol).

^{1}H NMR (501 MHz, CD_3CN) δ = 7.86 – 7.78 (m, 2H), 7.64 – 7.55 (m, 3H) ppm. ^{13}C NMR (126 MHz, CD_3CN) δ = 142.68, 132.90 (d, *J*=326.1), 130.56, 126.76, 119.97 (q, *J*=322.2) ppm. ^{19}F NMR (471 MHz, CD_3CN) δ = –79.34 ppm (s, 6F). HRMS: calculated for $C_8H_5F_6N_2O_4S_3$ ([M – H]$^-$): 402.932380; found: 402.932121

8.2 Phenylbis(trifluoromethylsulfonylimino)sulfonamide 6

N,N'-(amino(phenyl)-l6-sulfanediylidene)bis(1,1,1-trifluoromethanesulfonamide)

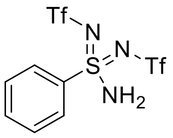

A 100 ml schlenk flask was charged with sodium phenylbis(trifluoromethylsulfinate) (5.33 g, 12.5 mmol), Selectfluor (13.6 g, 38. 5 mmol, 3 equiv.) and suspended in 30 ml acetonitrile. The suspension was stirred 16 h at 80 °C to form a yellowish suspension. An aliquote of the solution was taken, concentrated under inert conditions and analyzed by NMR (CD_3CN). ^{19}F-NMR indicated full conversion of the starting material to the desired sulfonyl fluoride (^{19}F NMR (471 MHz CD_2Cl_2 δ = 69.7 (s), –75.0 (s) ppm). All volatiles were removed in high vacuum under inert reaction conditions and the resulting solid residue re-suspended in anhydrous DCM (40 ml). The suspension was transferred to a schlenk-frit, which is filled with a plug of silica and connected to a 250 ml schlenk-flask, by cannula with argon overpressure and washed with additional DCM (3x20 ml). The combined DCM phase containing the desired sulfonyl fluoride was amidated with ammonia gas for 10 minutes to form a colorless suspension. The suspension was poured into a 250 ml round-bottom flask and all volatiles were removed in vacuum (Rotavap). The residue was dissolved in a mixture of diethylether (100 ml) and $HCl_{(aq)}$ (6M, 100 ml). The organic phase was additionally washed with $HCl_{(aq)}$ (6M, 2 x 100 ml), washed with brine and concentrated to dryness to yield the desired product as a beige solid, which was further purified by recrystallization from toluene to afford the desired product as a crystalline solid (88%, 4.60 g, 12.5 mmol).

^{1}H NMR (501 MHz, CD_3CN) δ = 8.2 – 8.1 (m, 2H), 7.9 – 7.9 (m, 1H), 7.8 – 7.7 (m, 2H), 7.4 (s br, 2H) ppm. ^{13}C NMR (126 MHz, CD_3CN) δ = 137.5, 137.4, 131.2, 129.0, 119.8 ppm (q, *J*=960.8). ^{19}F NMR (471 MHz, CD_3CN) δ = -79.3 (s) ppm. HRMS (ESI): m/z cald for $C_8H_6F_6N_3O_4S_3^-$: 417.943020 [M-H]$^-$, found 417.942700

9 Synthesis of Imidodiphosphorsulfonyliminoimidates (IDPii)

9.0 General remarks

HCPP and **HCPC** show similar reactivity with phenylbis(trifluoromethylsulfonylimino)sulfonamide **6**. However, the counteranion PCl_6^- of **HCPP** also reacts with **6** to afford phenylbis(trifluoromethylsulfonylimino)phosphorimidoyl trichloride, which would interfere with the BINOL installation step. Therefore, the PCl_6^--counteranion was exchanged with a Cl^--counteranion to prevent any undesired reactivity.

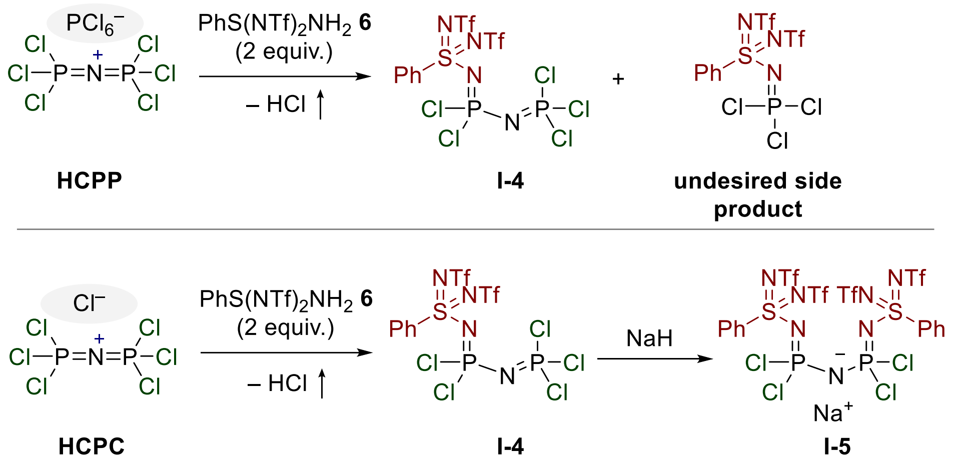

NMR spectra (CD_2Cl_2) of **I-3** and **I-4**, respectively, are shown. NMR spectra correspond to reaction mixtures and not to isolated compounds:

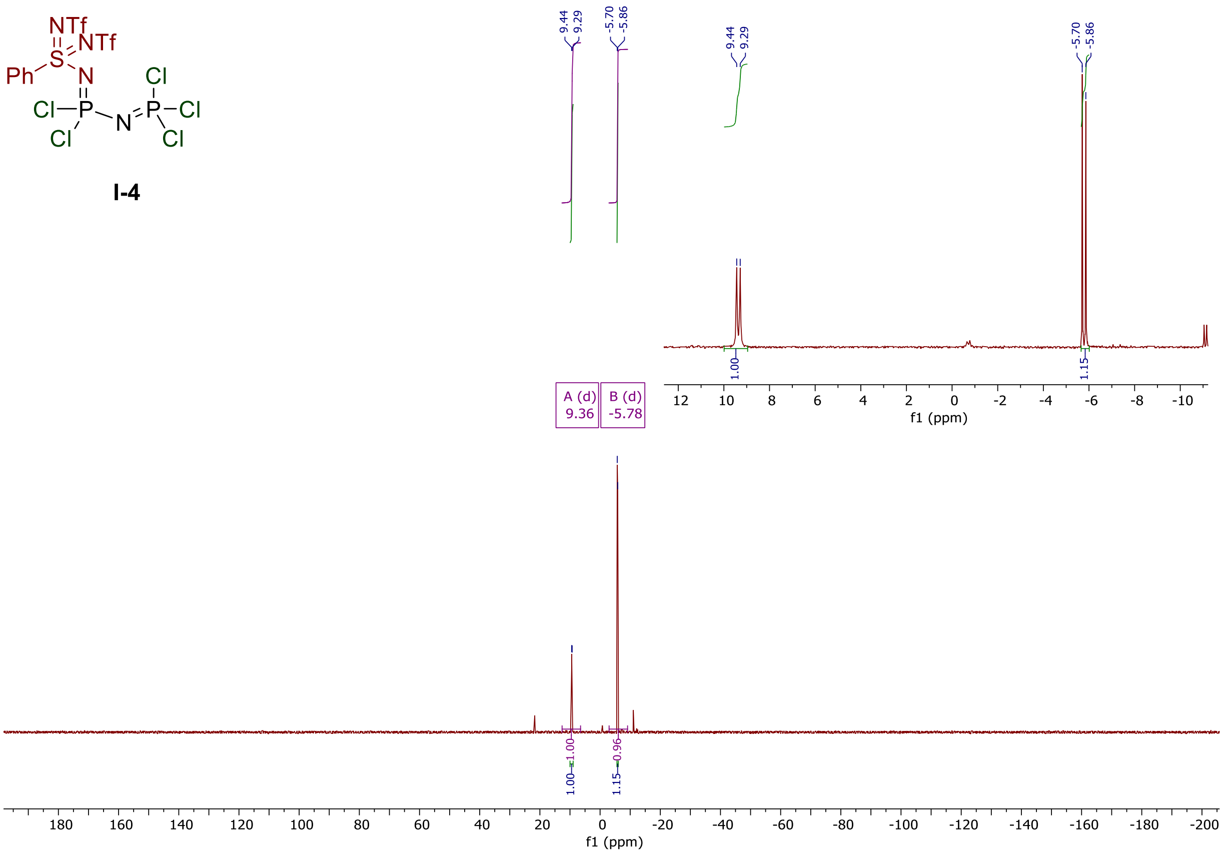

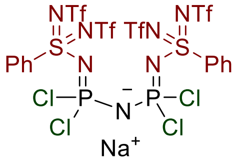

I-5

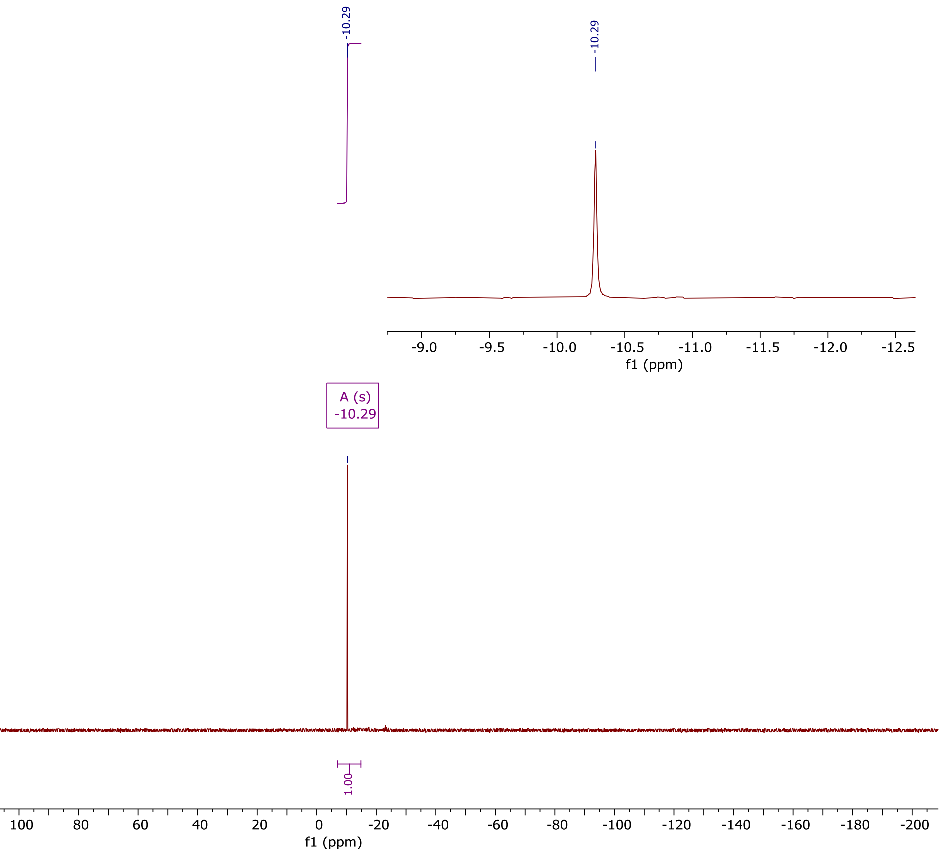

-10.29
-10.29
-9.0
-9.5
-10.0
-10.5
-11.0
-11.5
-12.0
-12.5
f1 (ppm)
A (s)
-10.29
1.00
180
160
140
120
100
80
60
40
20
0
-20
-40
-60
-80
-100
-120
-140
-160
-180
-200
f1 (ppm)

9.1 Synthesis of IDPii 9a

N,N',N'',N'''-(((azanediylbis(2,6-diphenyl-4l5-dinaphtho[2,1-d:1',2'-f][1,3,2]dioxaphosphepine-4-yl-4-ylidene))bis(azanylylidene))bis(phenyl-l6-sulfanyldiylidene))tetrakis(1,1,1-trifluoromethanesulfonamide)

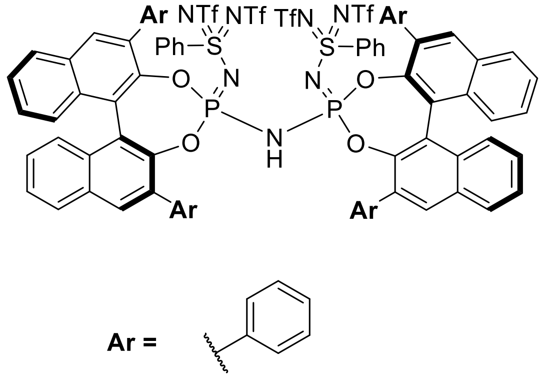

A 25 ml flame dried and argonated schlenk flask was charged with hexachlorobisphosphazonium chloride (132 mg, 407 µmol, 1 equiv.) and of N,N'-(amino(phenyl)-l6-sulfanediylidene)bis(1,1,1-trifluoromethane-sulfonamide) from example 18 (343 mg, 818 µmol, 2 equiv.) and suspended in toluene (4 ml). The suspension was stirred for 15 minutes until gas development ceased. Sodium hydride (dispersion in mineral oil, 60%, 218 mg, 5.45 mmol, 13 equiv.) was added and the reaction mixture stirred 3 h at 60 °C. (S)-3,3´-(Phenyl)BINOL (401 mg, 914 µmol, 2.2 equiv.) was added and the reaction mixture stirred for 23 h at 100 °C. The reaction mixture was then carefully poured into sat. NaHCO3 and the aqueous layer extracted with DCM. The combined organic phases were washed with brine, dried over sodium sulfate and concentrated to dryness followed by purification by FCC (Biotage, gradient DCM up to DCM/MeOH (3:2)) to elute the desired product as a salt. This salt was then acidified by dissolving in a small quantitiy of DCM and flushing through a 5 cm pad of DOWEX® 50WX8. The organic phase was concentrated to dryness to furnish the desired product in acidic form (60%, 437 mg).

^{1}H NMR (501 MHz, CD_2Cl_2) δ = 8.07 – 7.99 (m, 4H), 7.78 – 7.71 (m, 4H), 7.59 – 7.53 (m, 4H), 7.52 – 7.47 (m, 2H), 7.36 – 7.10 (m, 24H), 7.02 – 6.92 (m, 6H), 6.88 – 6.79 (m, 6H), 5.56 (s br, 1H) ppm. ^{13}C NMR (126 MHz, CD_2Cl_2) δ = 143.96, 143.76, 143.72, 139.41, 136.09, 134.94, 133.18, 133.06, 132.85, 132.68, 132.61, 132.32, 132.01, 131.87, 130.77, 129.92, 129.83, 129.71, 129.58, 128.99, 128.90, 128.58, 128.39, 128.07, 127.69, 127.53, 127.45, 127.34, 127.11, 126.89, 126.82, 125.82, 123.69, 123.12, 122.82, 120.56, 120.52, 118.00, 115.43 ppm (other signals not observed or detected). ^{19}F NMR (471 MHz, CD_2Cl_2) δ = –77.22 (s, 3F), –77.96 (s, 3F) ppm. ^{31}P NMR (203 MHz, CD_2Cl_2) δ = –20.11 (s, 2P) ppm. HRMS (ESI): m/z calcd for $C_{80}H_{50}F_{12}N_7O_{12}P_2S_6^-$: 1782.113102 [M-H]⁻, found 1782.110140; $[\alpha]_D^{25}$ = +126 (c = 1 mg/ml in CH_2Cl_2)

9.2 Synthesis of IDPii 9b

N,N',N'',N'''-(((azanediylbis(2,6-bis(3,5-bis(trifluoromethyl)phenyl)-4l5-dinaphtho[2,1-d:1',2'-f][1,3,2]dioxaphosphepine-4-yl-4-ylidene))bis(azanylylidene))bis(phenyl-l6-sulfanyldiylidene))tetrakis(1,1,1-trifluoromethanesulfonamide)

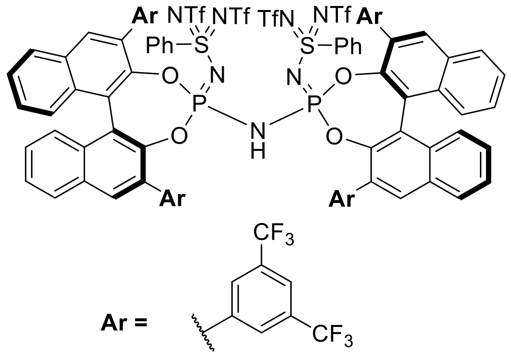

A 10 ml schlenk-tube was charged with HCPC (73.0 mg, 0.23 mmol), phenylbis(trifluoromethylsulfonylimino)sulfonamide (191 mg, 0.46 mmol, 2 equiv.) and suspended in 2 ml toluene. The reaction was stirred 30 min at r.t. (150 rpm) until the gas development ceased. Sodium hydride (dispersion in mineral oil, 60%; 116 mg, 2.90 mmol, 13 equiv.) was added and the resulting suspension stirred for 2 h in a pre-heated metal block to 110 °C. (*S*,*S*)-3,3´-(m,m-bistrifluoromethylphenyl)BINOL was added in one portion and the sealed schlenk-flask and 44 h stirred at 110°C. The reaction mixture was cooled to r.t., diluted with DCM and poured into sat. NaHCO3(aq). The aqueous phase was extracted with DCM (4 x 20 ml), the combined organic phase dried over sodium sulfate and concentrated to dryness. Flash column purification (Biotage gradient; DCM / MeOH up to 4 / 1) yields the desired product as a salt, which was acidified by dissolving the product in a small quantitiy of DCM and passing through pre-activated Dowex40WX8 to afford the desired product in acidic form (81%, 426 mg, 0.18 mmol);

^{1}H NMR (600 MHz, CD_2Cl_2) δ = 8.1 (dt, *J*=8.4, 1.1, 2H), 8.0 (m, 6H), 7.9 (s, 2H), 7.8 – 7.7 (m, 6H), 7.7 (ddd, *J*=8.2, 6.8, 1.2, 2H), 7.6 (ddd, *J*=8.1, 6.6, 1.5, 2H), 7.5 (d, *J*=8.6, 2H), 7.3 (ddd, *J*=8.7, 6.8, 1.3, 2H), 7.1 (d, *J*=7.6, 4H), 7.0 – 6.9 (m, 6H), 6.8 – 6.7 (m, 2H), 6.3 (t, *J*=7.8, 4H), 6.3 (s, 2H), 6.1 (sbr, 1H) ppm. ^{13}C NMR (151 MHz, CD_2Cl_2) δ = 143.7, 143.7, 142.6, 142.5, 142.5, 139.3, 139.1, 138.5, 135.3, 134.0, 133.3, 133.2, 132.6, 132.1, 131.8, 131.7, 131.7, 131.6, 131.5, 131.3, 131.3, 131.2, 131.1, 131.1, 130.7, 130.1, 129.9, 129.5, 129.0, 128.9, 128.5, 128.1, 127.7, 127.3, 127.2, 127.1, 126.7, 126.4, 124.9, 124.6, 123.1, 123.0, 122.9, 122.8, 122.1, 121.3, 121.0, 120.8, 120.4, 118.7, 118.3 ppm (other signals not observed or detected.^{19}F NMR (471 MHz, CD_2Cl_2) δ = –62.1 (s, 3F), –62.7 (s, 3F), –74.6 (s, 3F), –78.6 (s, 3F) ppm. ^{31}P NMR (203 MHz, CD_2Cl_2) δ = –19.7 (s, 2P). HRMS (ESI): m/z cald for $C_{88}H_{42}N_7O_{12}S_6F_{36}P_2^-$: 2326.012186 [M-H]$^-$, found 2326.013370; $[\alpha]_D^{25}$ = +158 (c = 1 mg/ml in CH_2Cl_2)

10 NMR-spectra

^{1}H NMR (501 MHz, CD_2Cl_2)

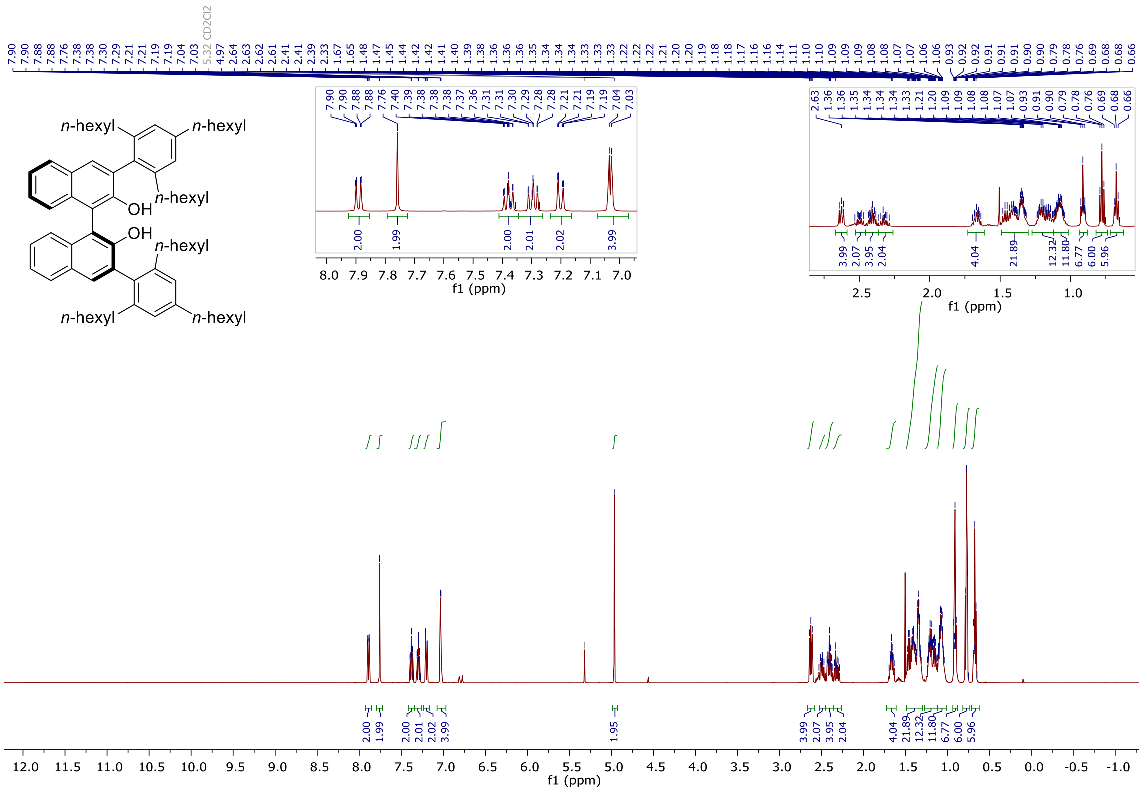

^{13}C NMR (126 MHz, CD_2Cl_2)

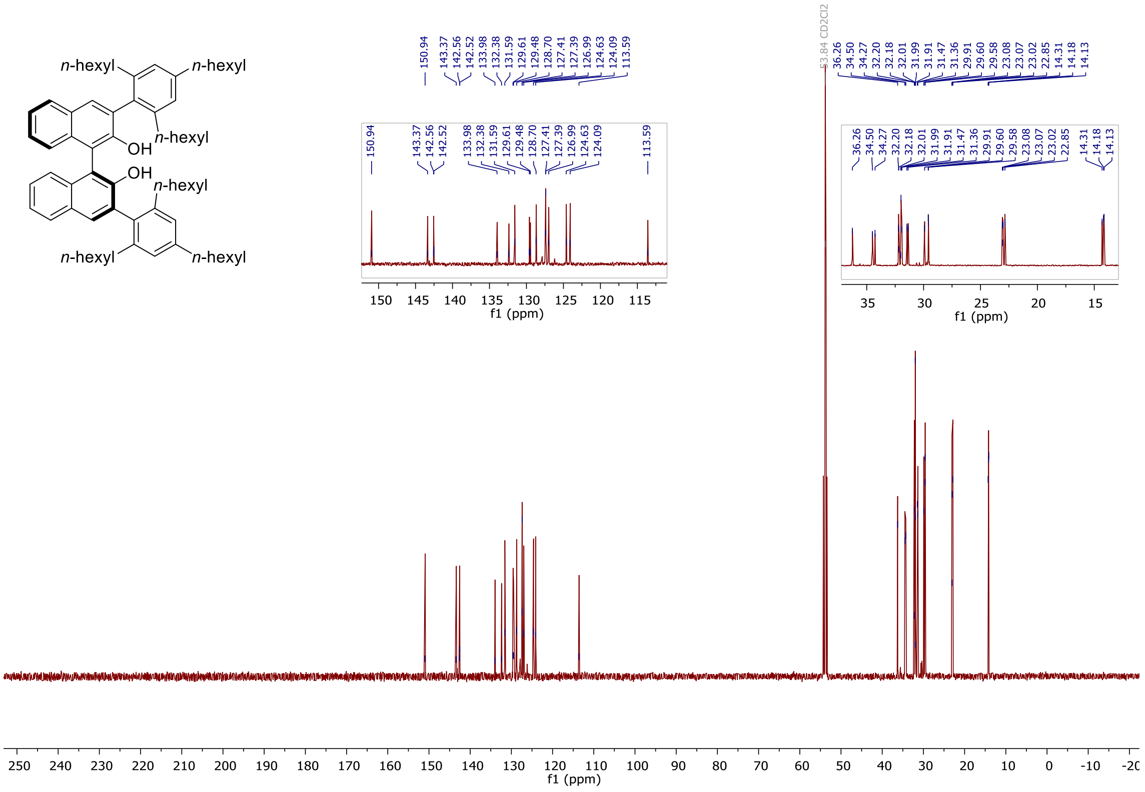

^{1}H NMR (501 MHz, CD_2Cl_2)

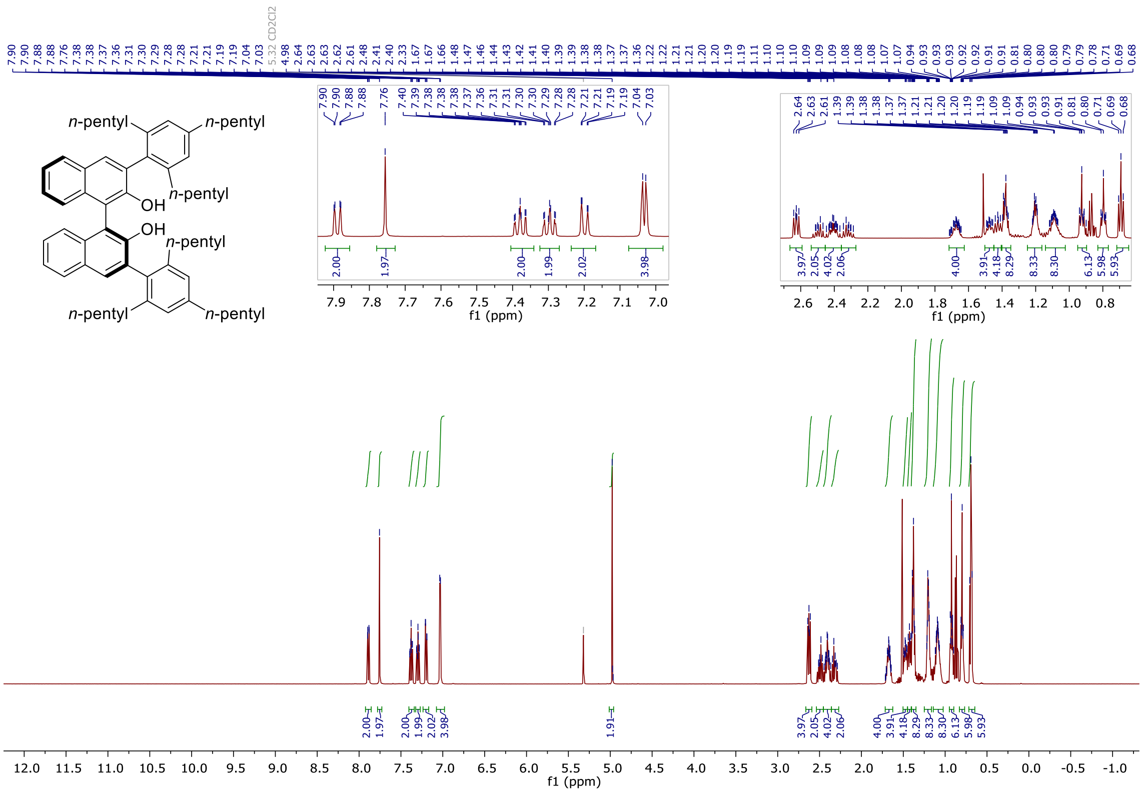

^{13}C NMR (126 MHz, CD_2Cl_2)

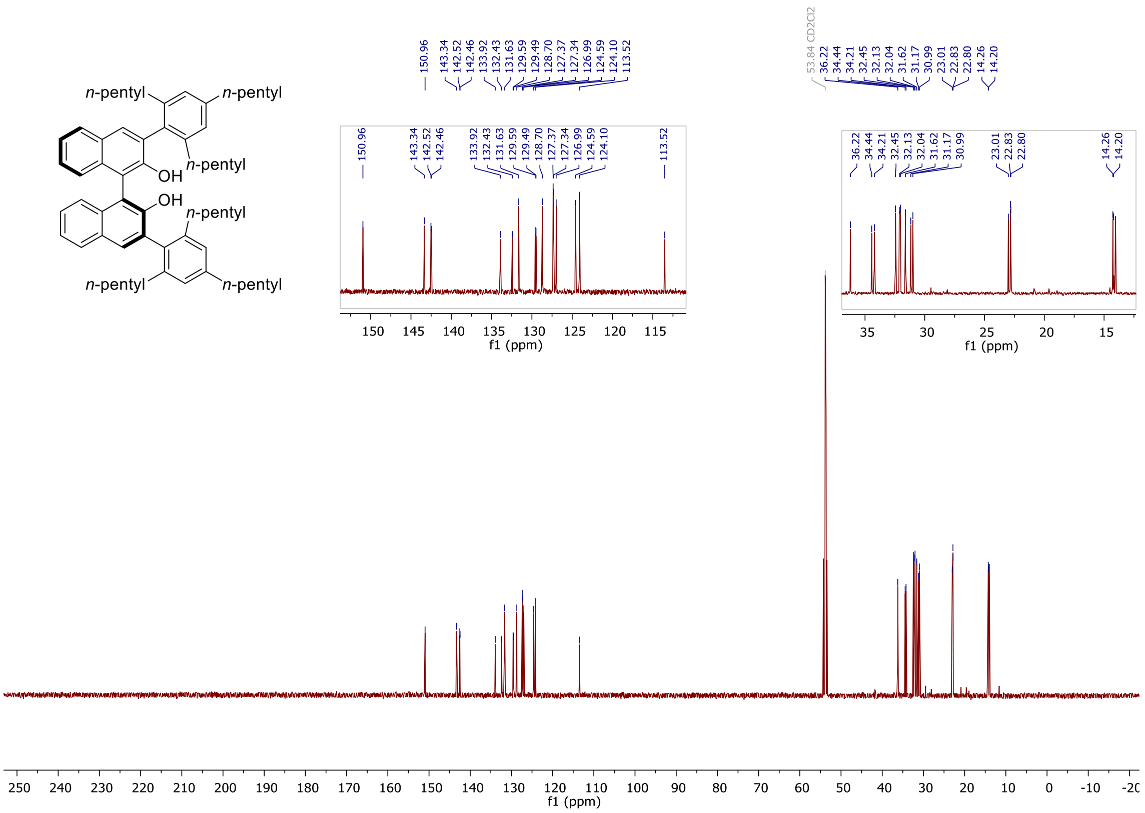

^{1}H NMR (501 MHz, CD_2Cl_2)

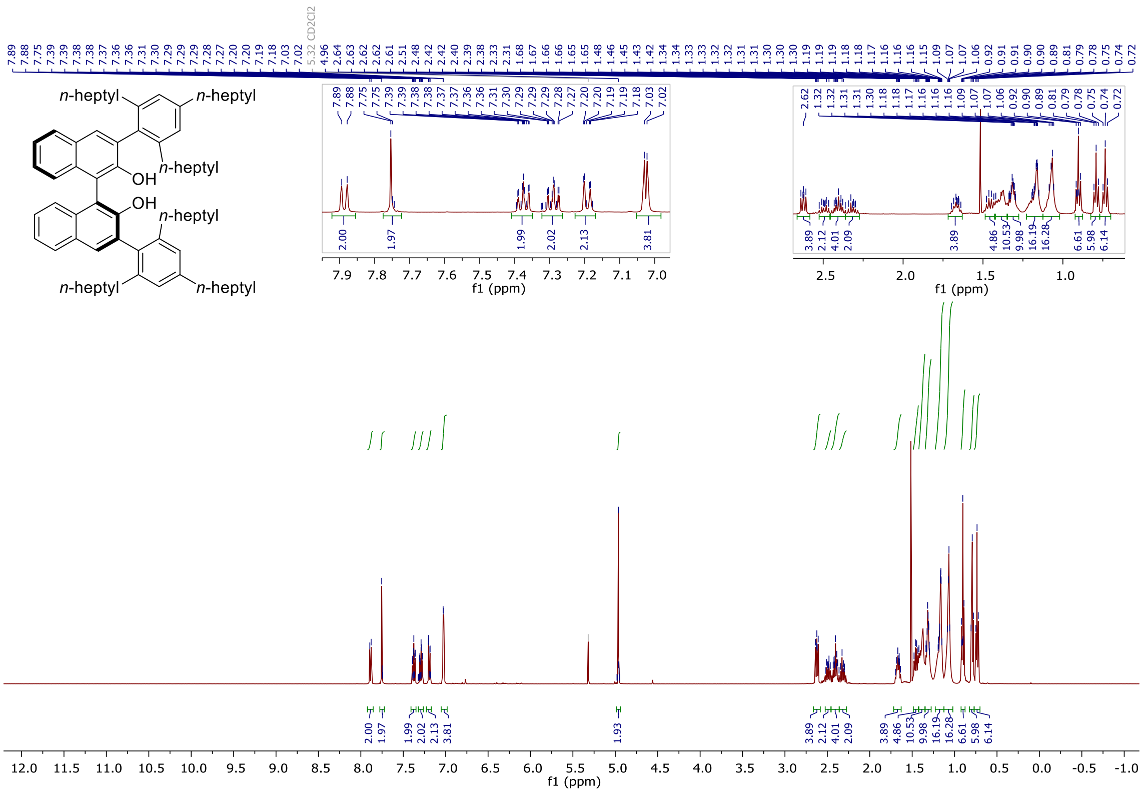

^{13}C NMR (126 MHz, CD_2Cl_2)

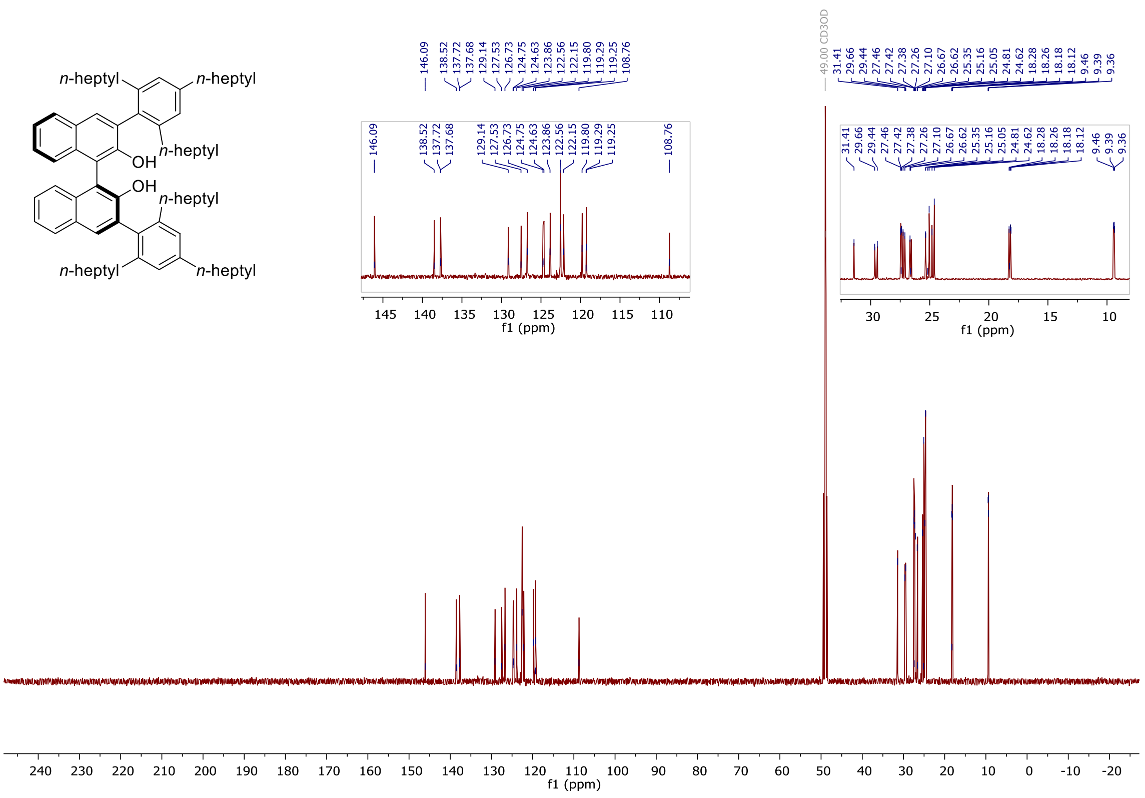

^{1}H NMR (501 MHz, $CDCl_3$)

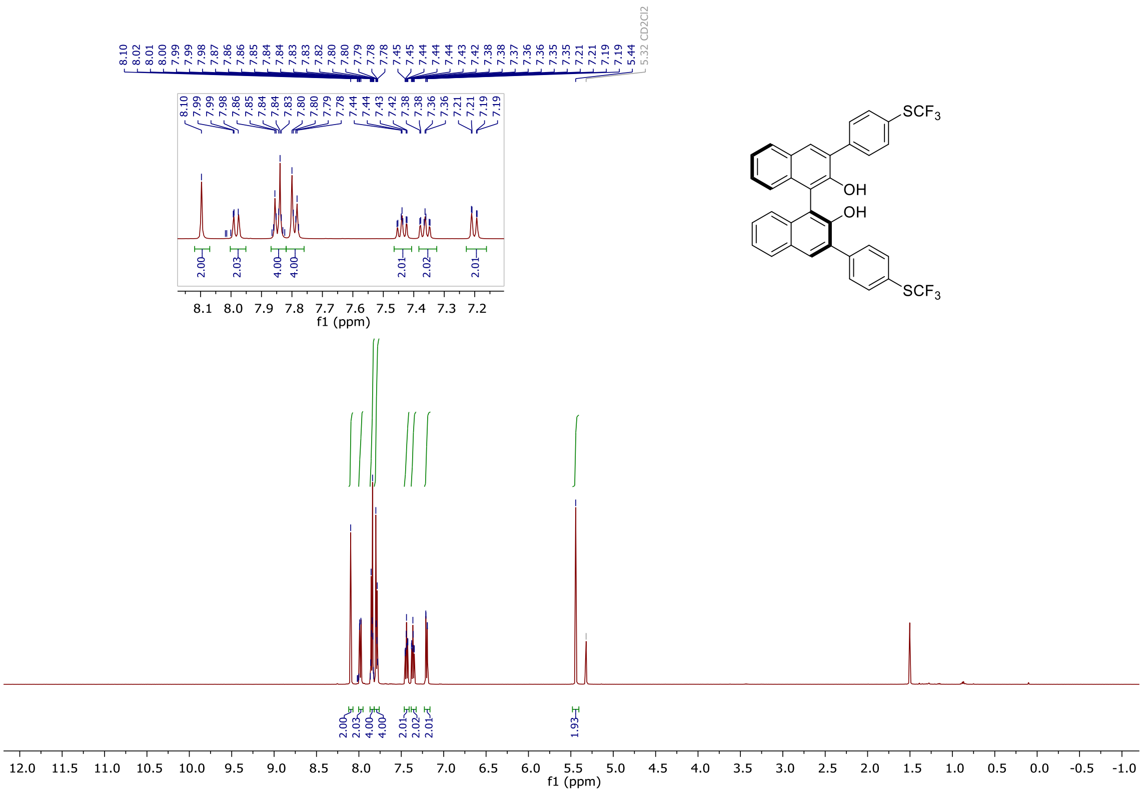

^{13}C NMR (126 MHz, $CDCl_3$)

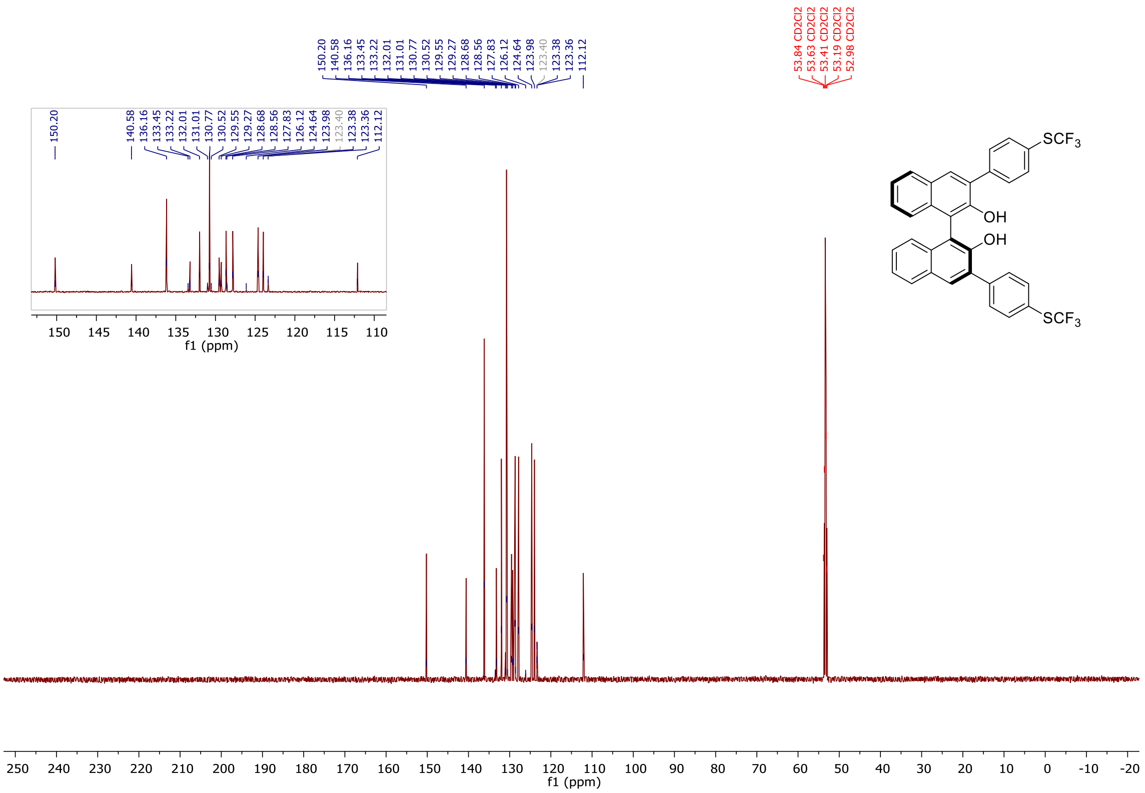

^{19}F NMR (471 MHz, $CDCl_3$)

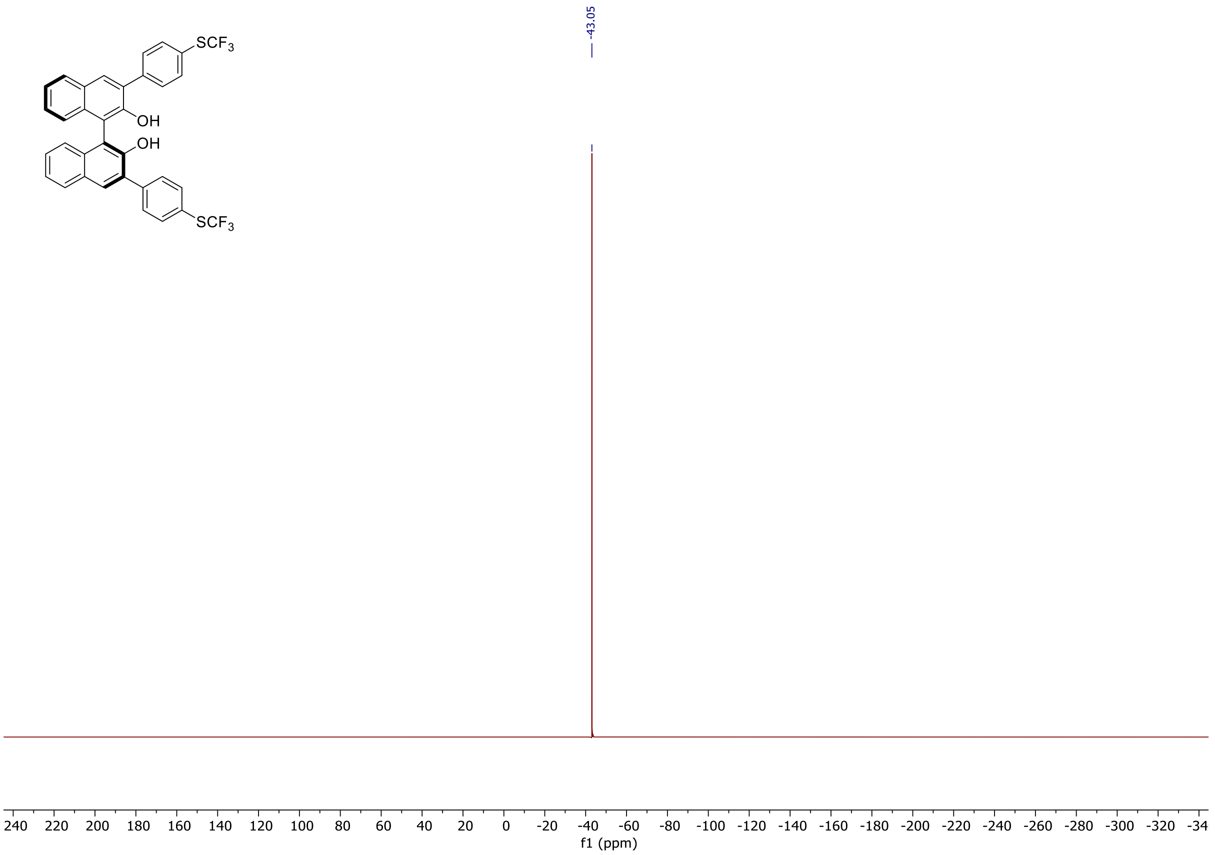

^{1}H NMR (501 MHz, CD_2Cl_2)

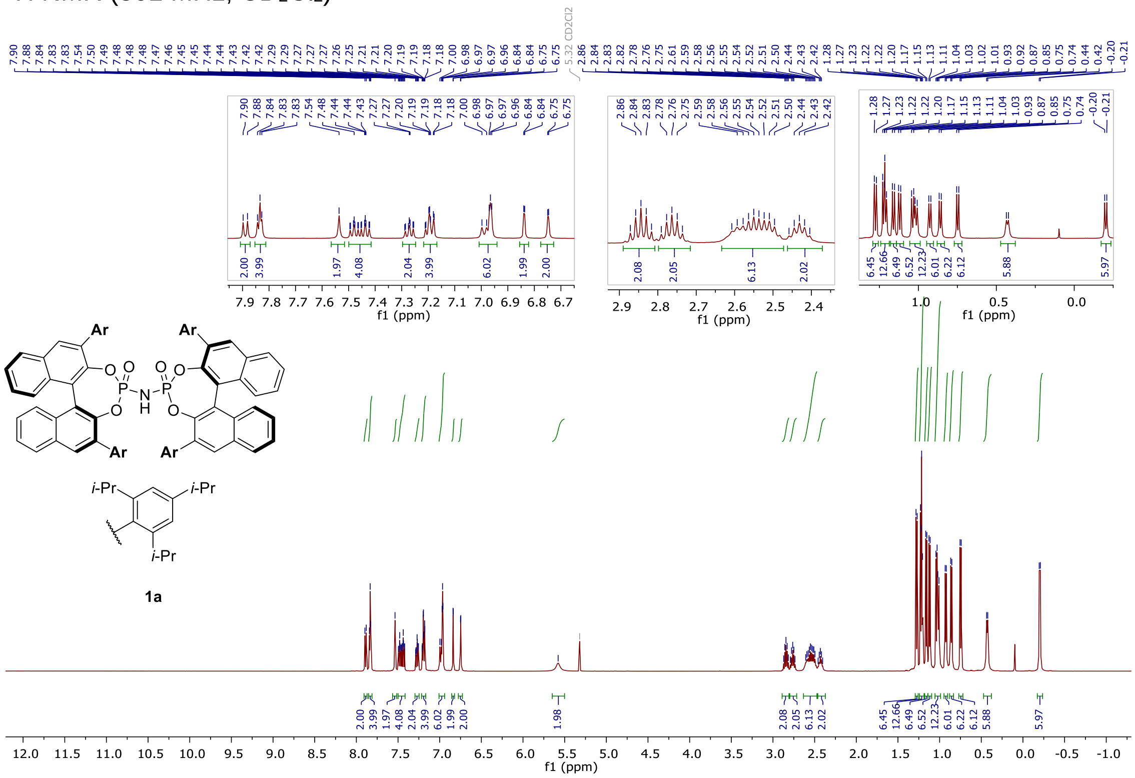

^{13}C NMR (126 MHz, CD_2Cl_2)

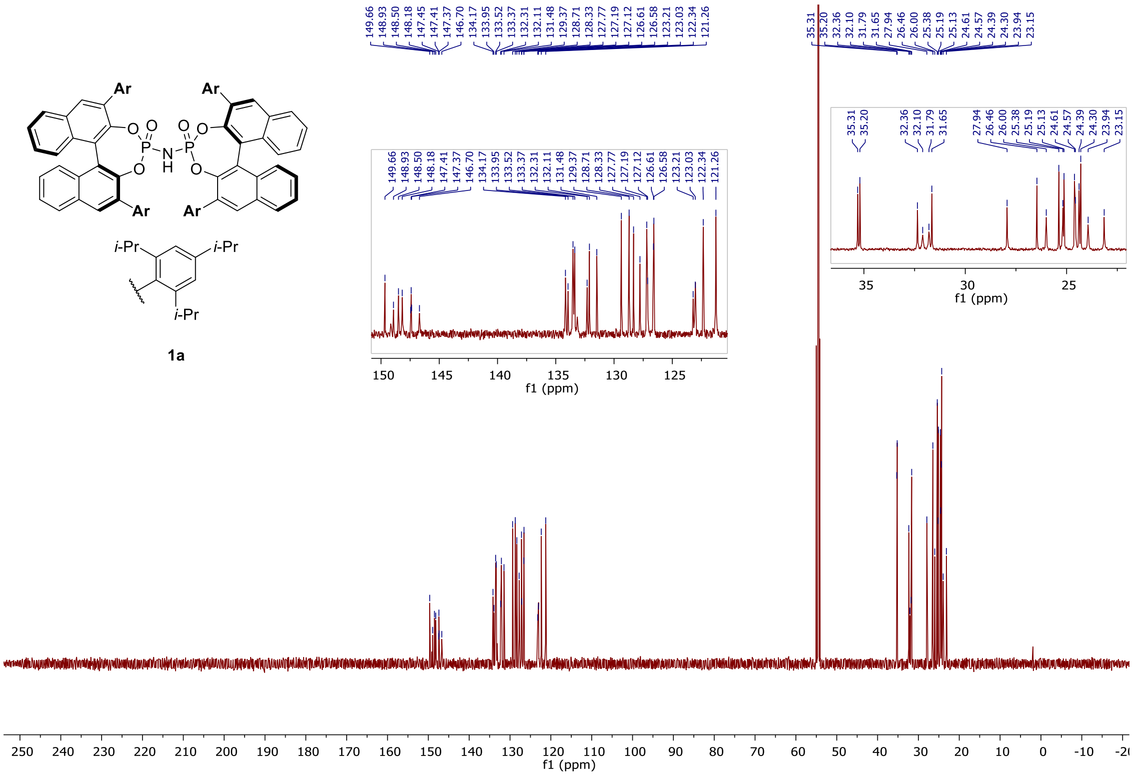

^{31}P NMR (203 MHz CD_2Cl_2)

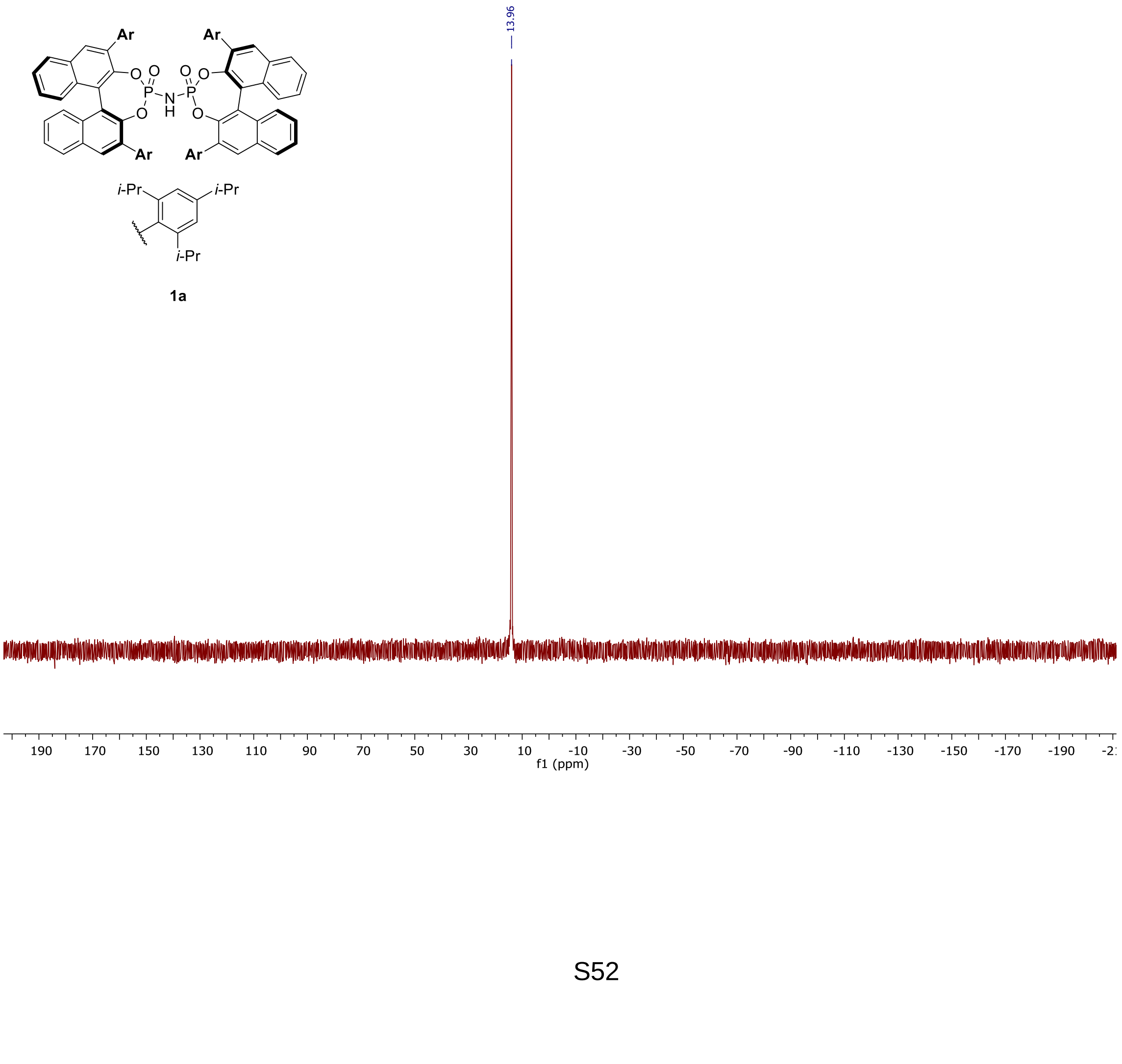

^{1}H NMR (501 MHz, CD_2Cl_2)

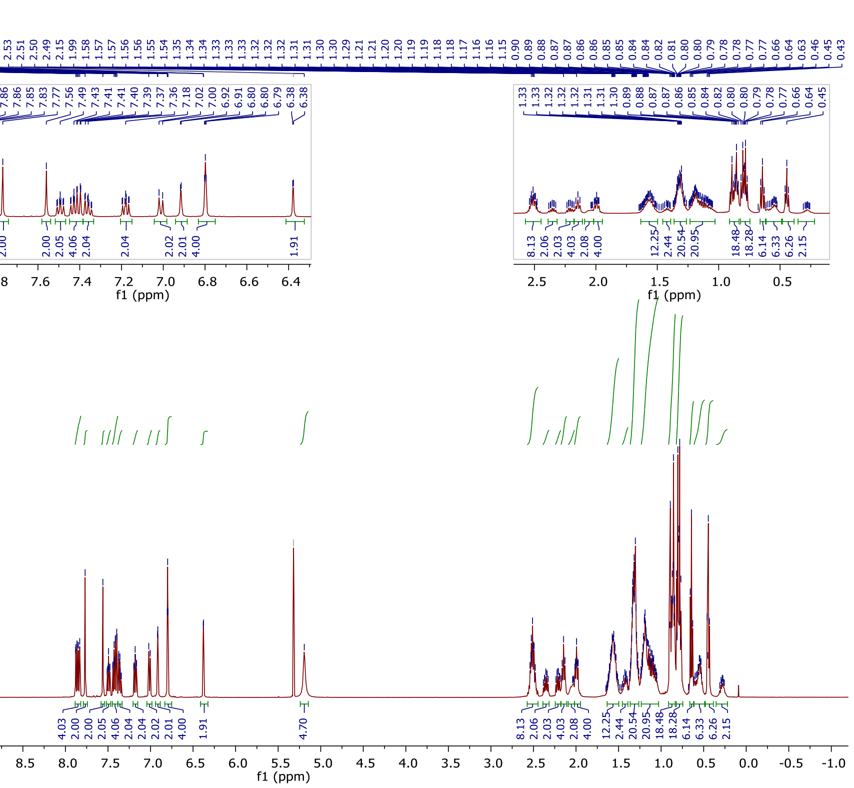

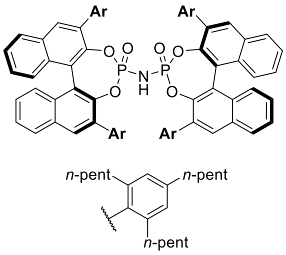

1b

^{13}C NMR (126 MHz, CD_2Cl_2)

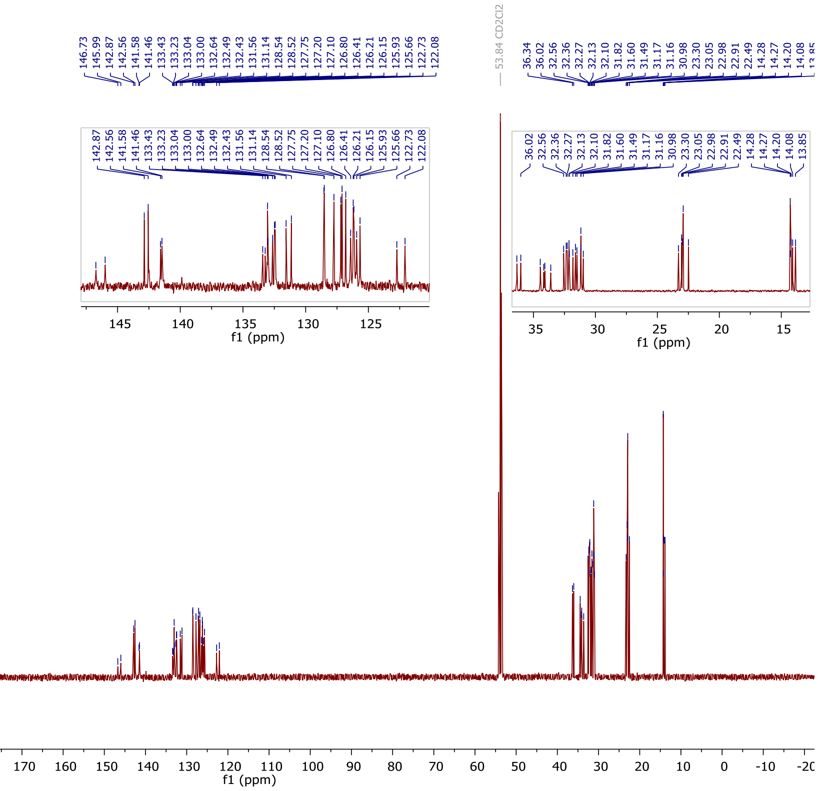

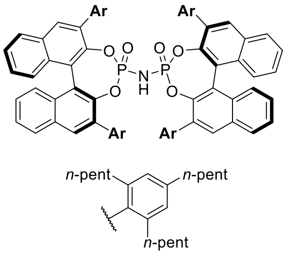

1b

^{31}P NMR (203 MHz, CD_2Cl_2)

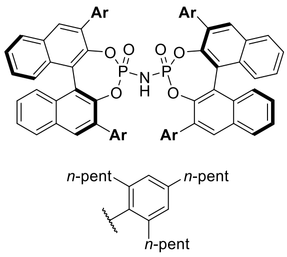

1b

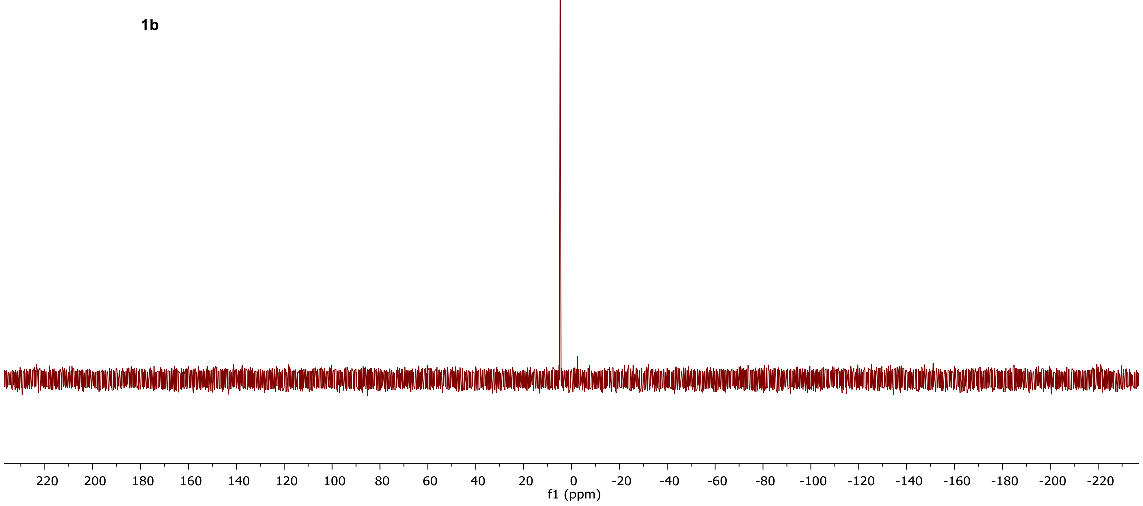

^{1}H NMR (501 MHz, CD_2Cl_2)

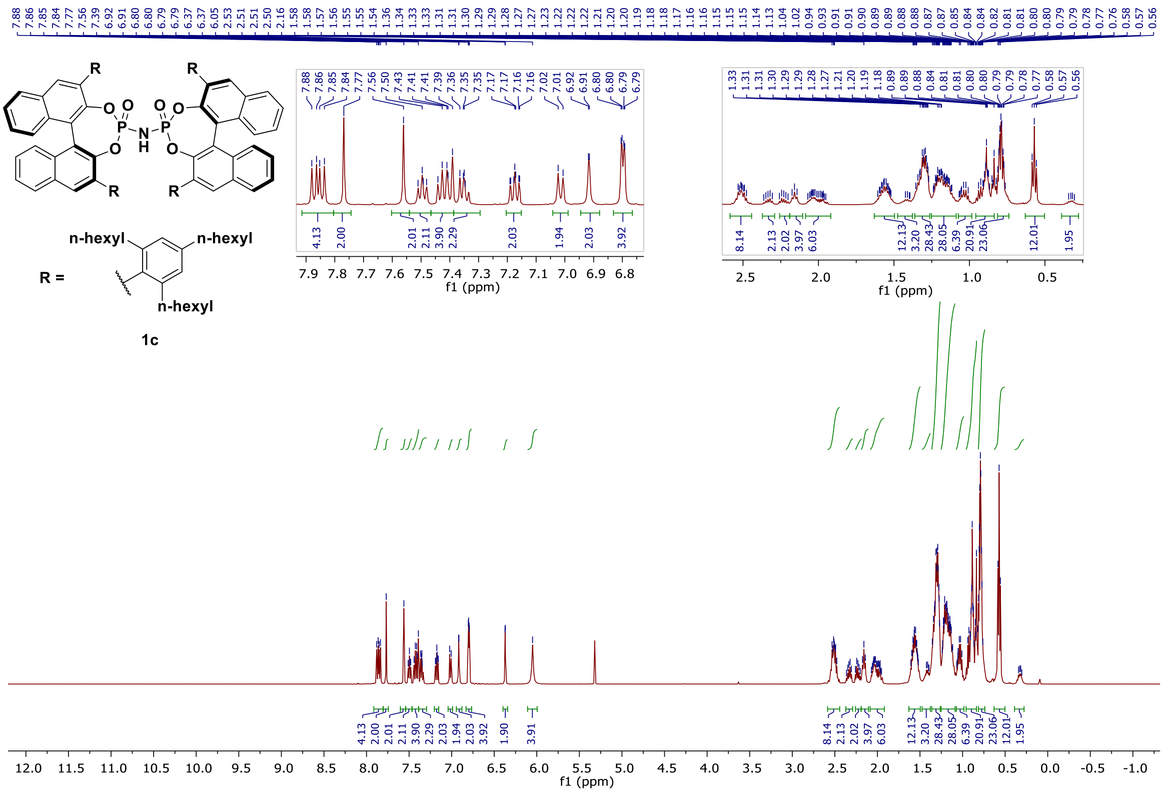

^{13}C NMR (126 MHz, CD_2Cl_2)

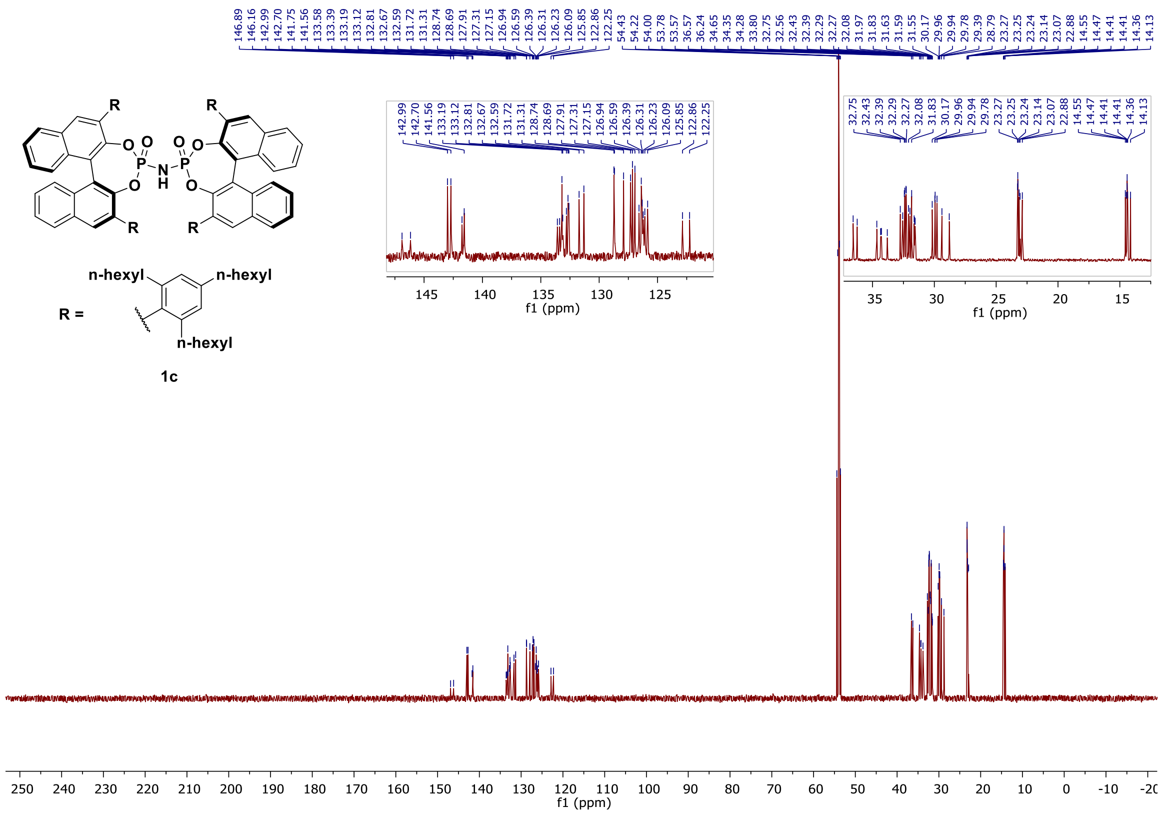

^{31}P NMR (203 MHz, CD_2Cl_2)

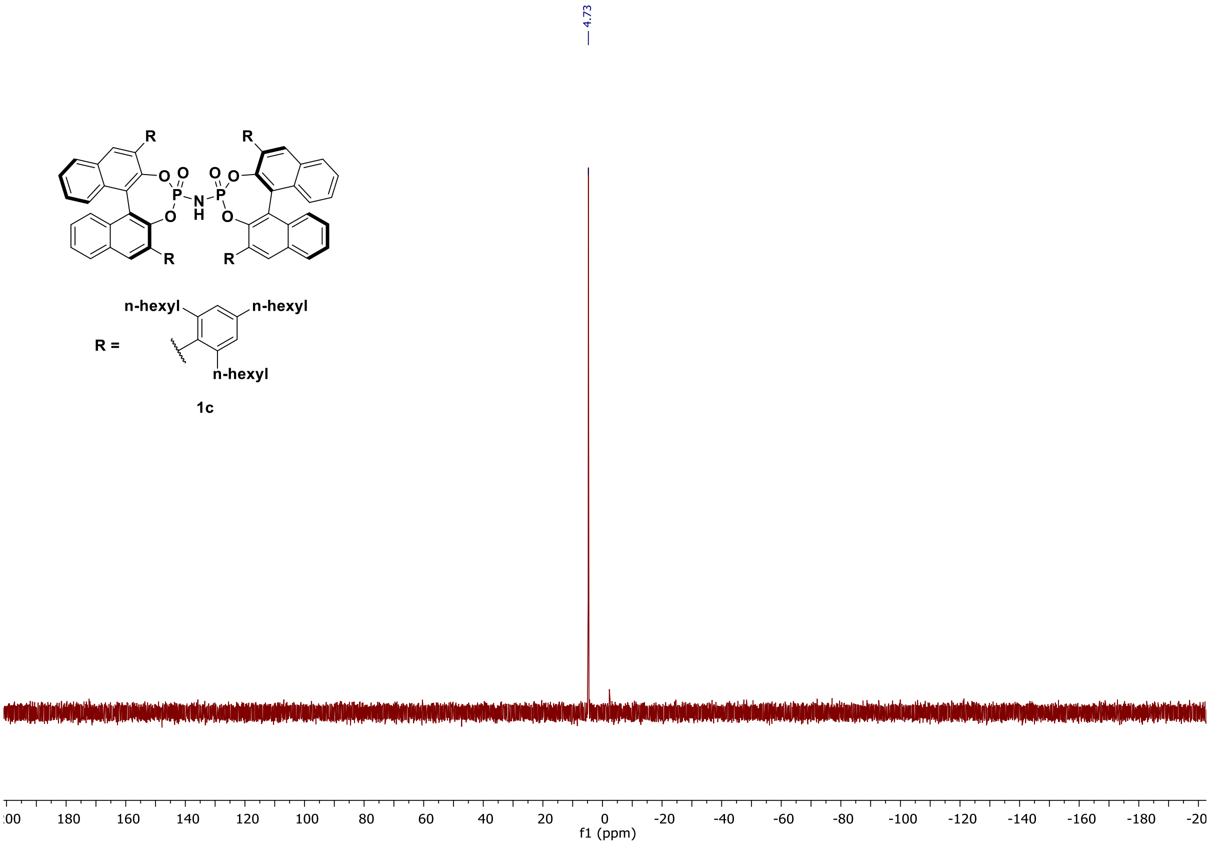

1c

^{1}H NMR (501 MHz, CD_2Cl_2)

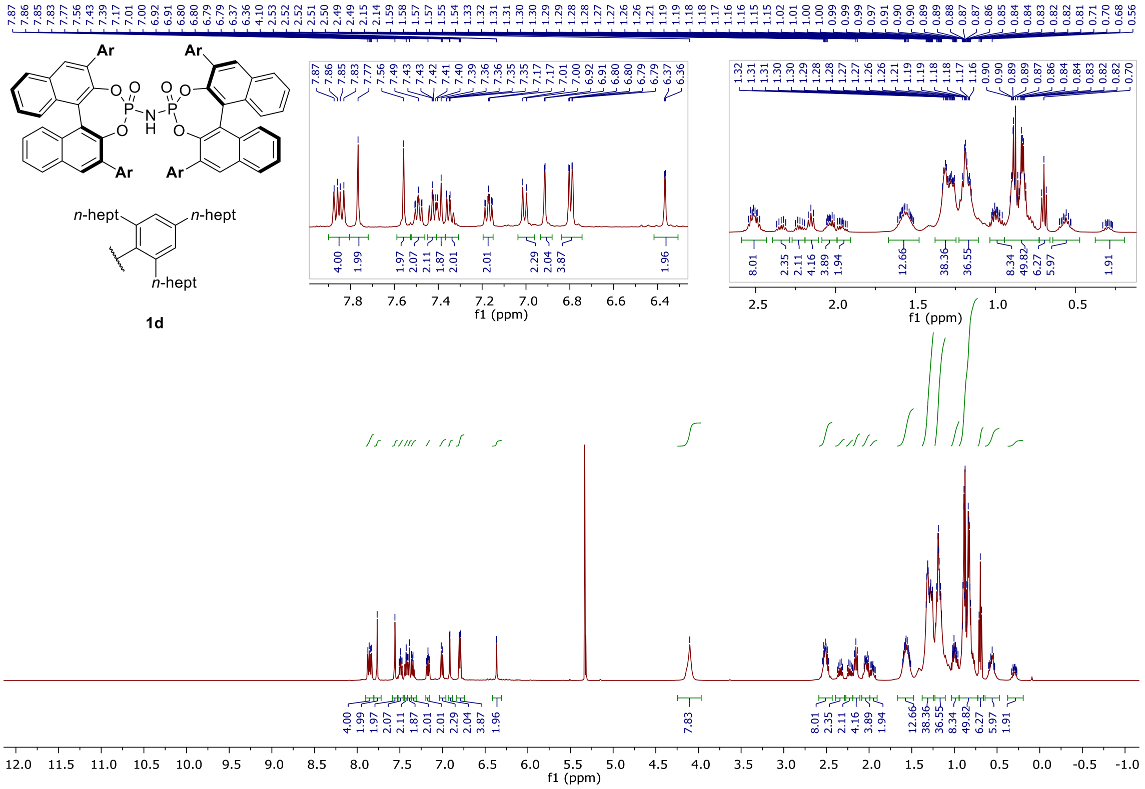

^{13}C NMR (126 MHz, CD_2Cl_2)

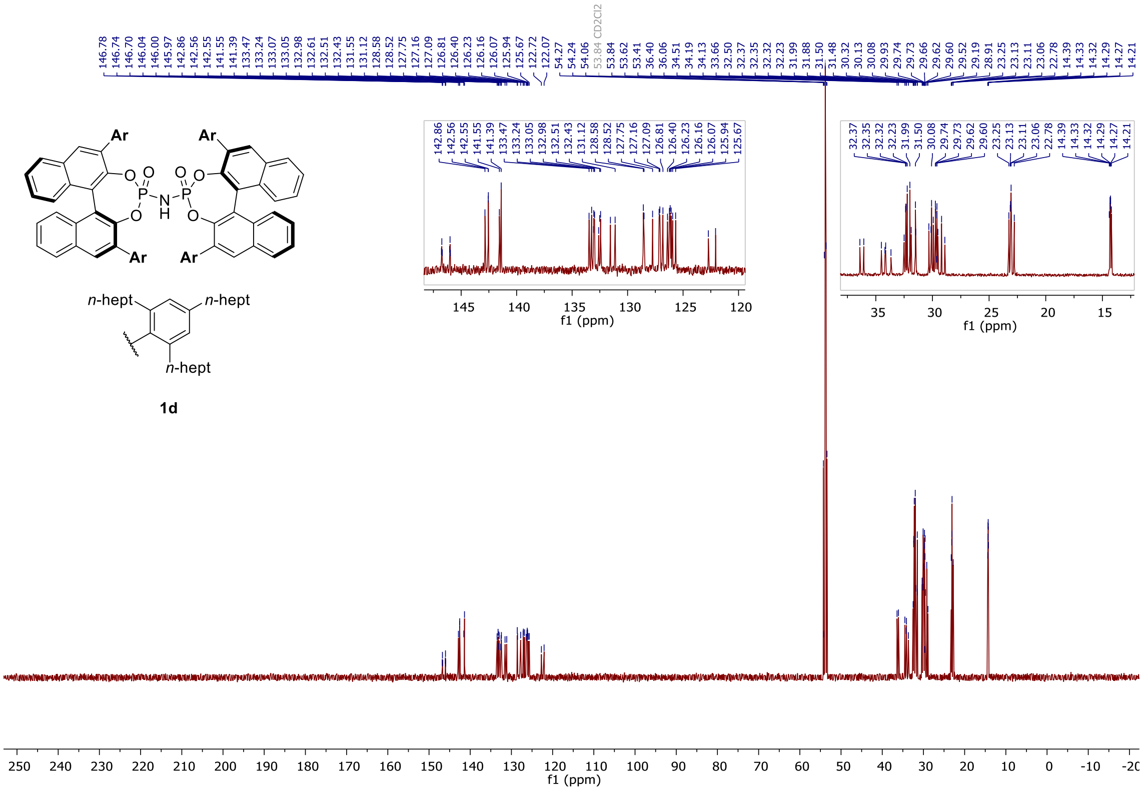

^{31}P NMR (203 MHz, CD_2Cl_2)

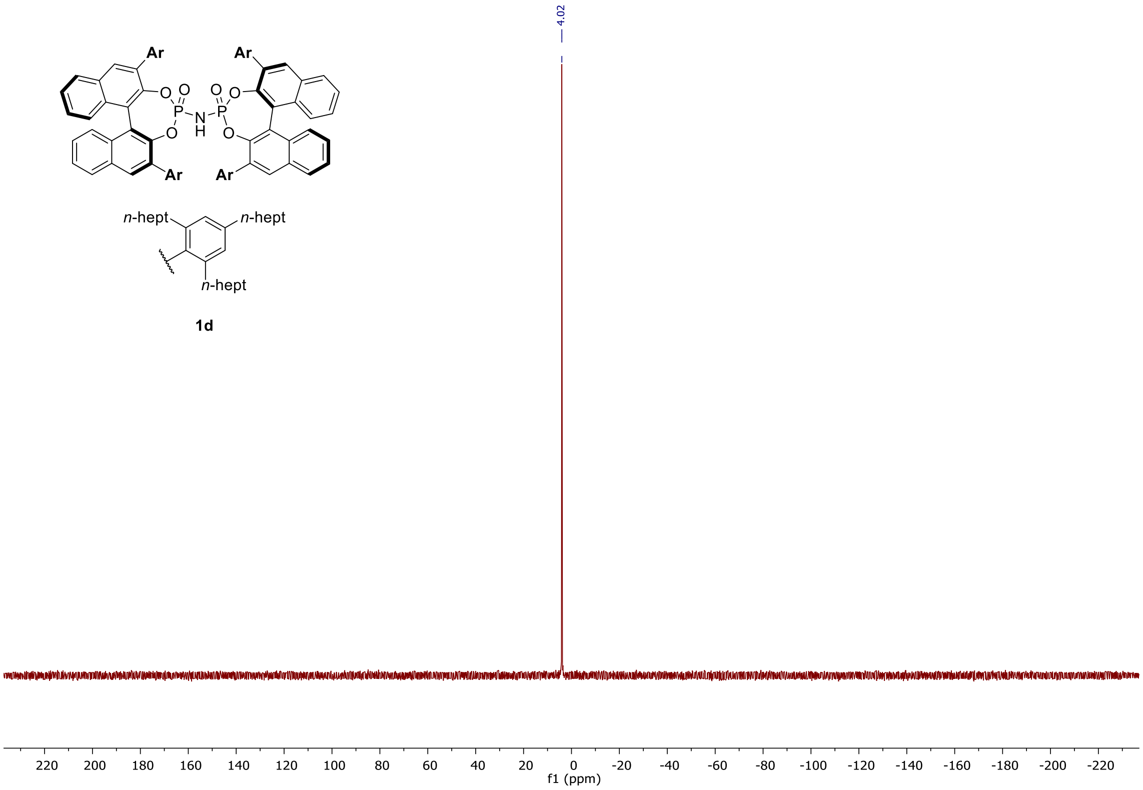

^{1}H NMR (501 MHz, CD_2Cl_2)

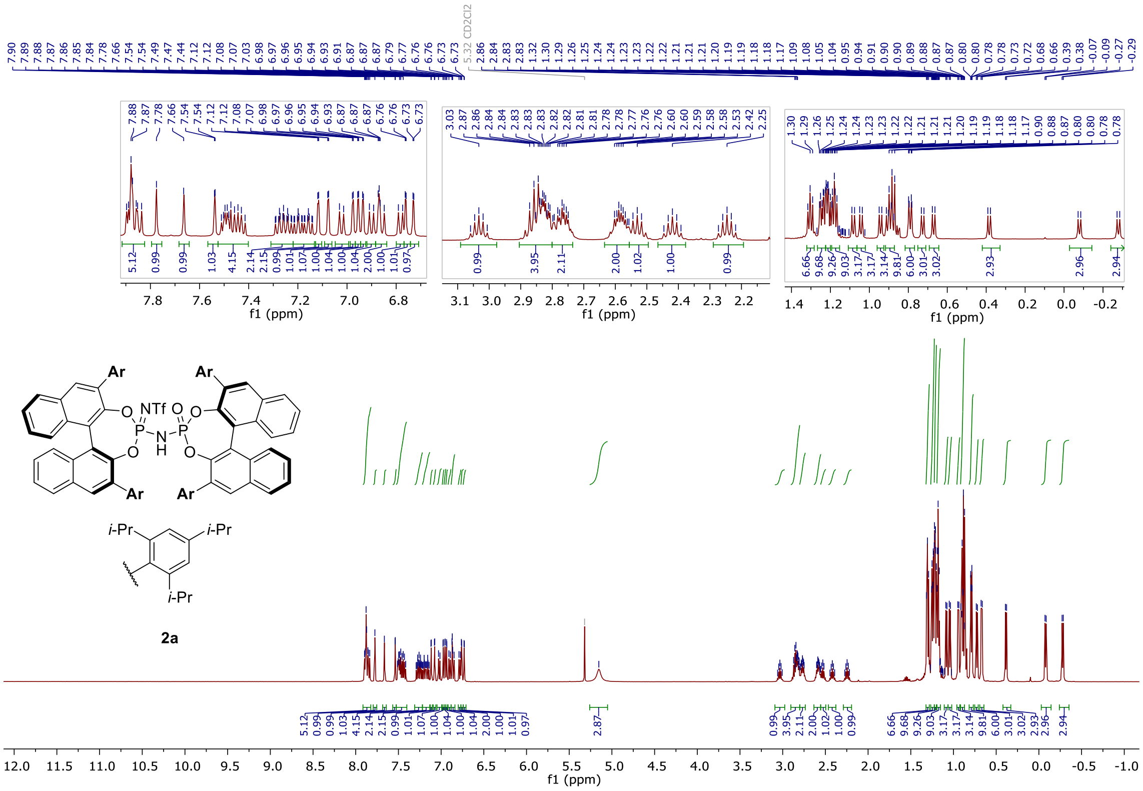

^{13}C NMR (126 MHz, CD_2Cl_2)

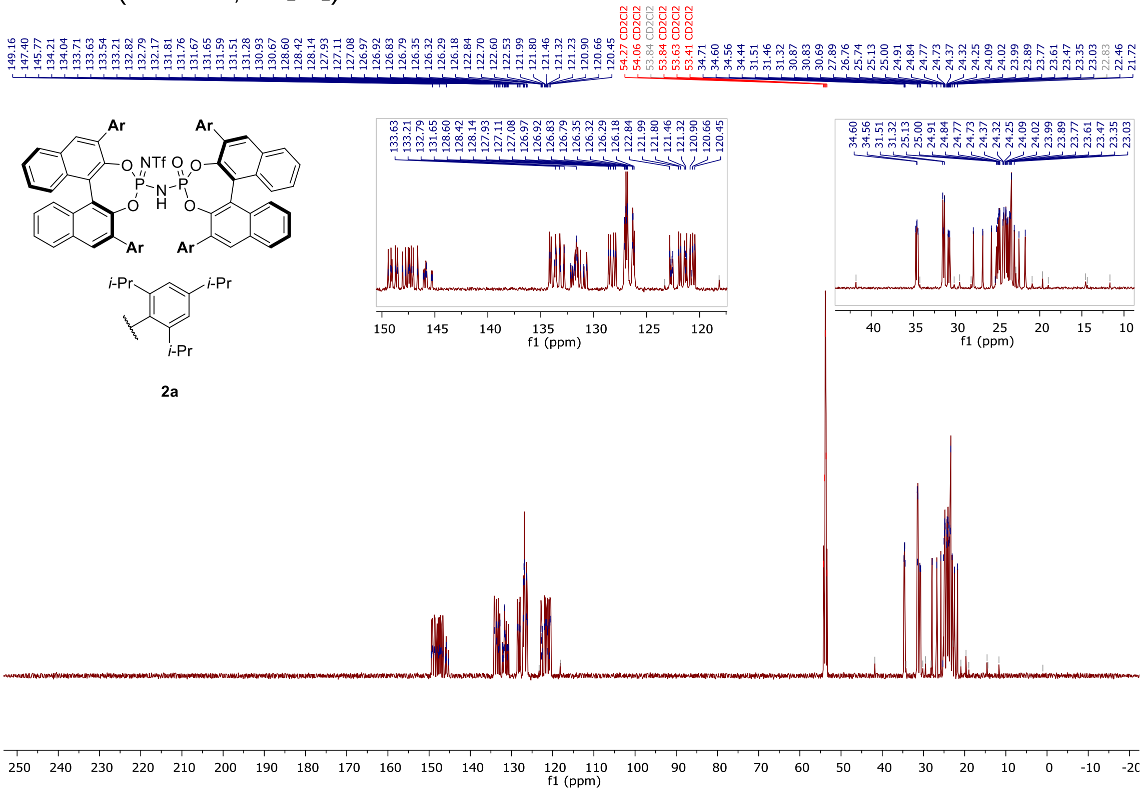

^{19}F NMR (471 MHz, CD_2Cl_2)

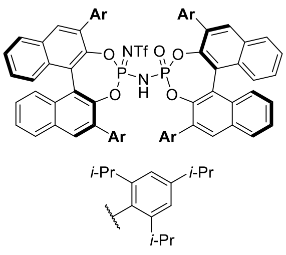

2a

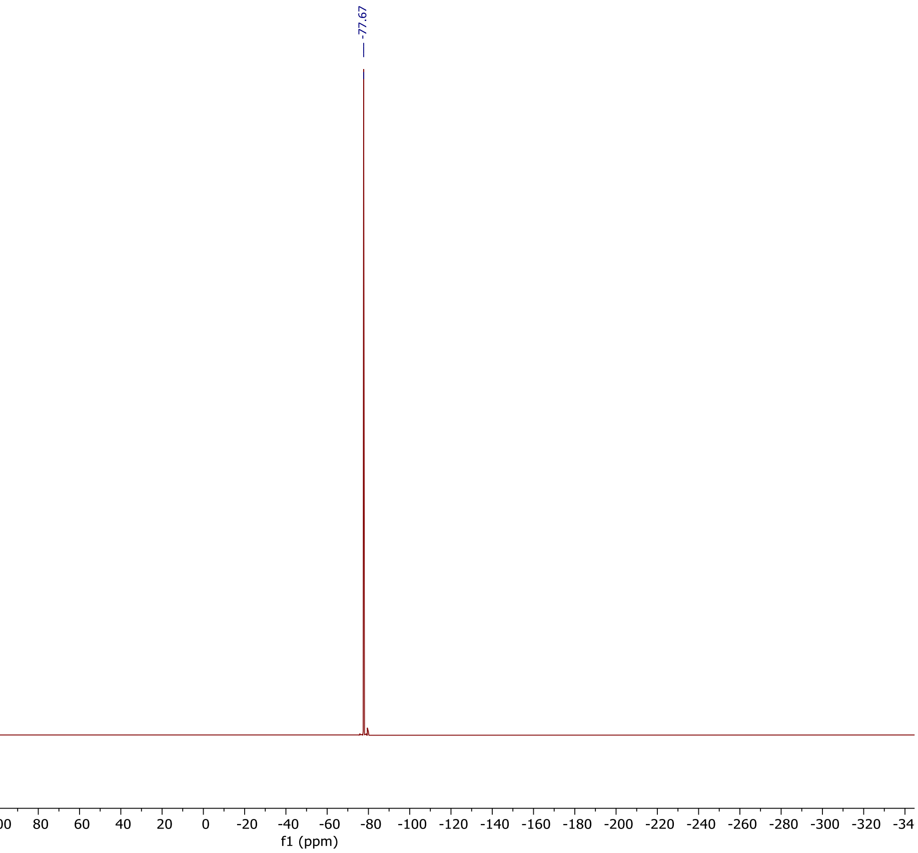

^{31}P NMR (203 MHz, CD_2Cl_2)

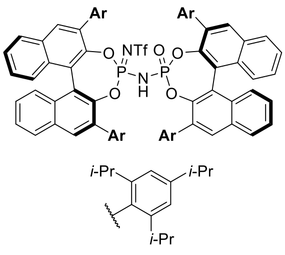

2a

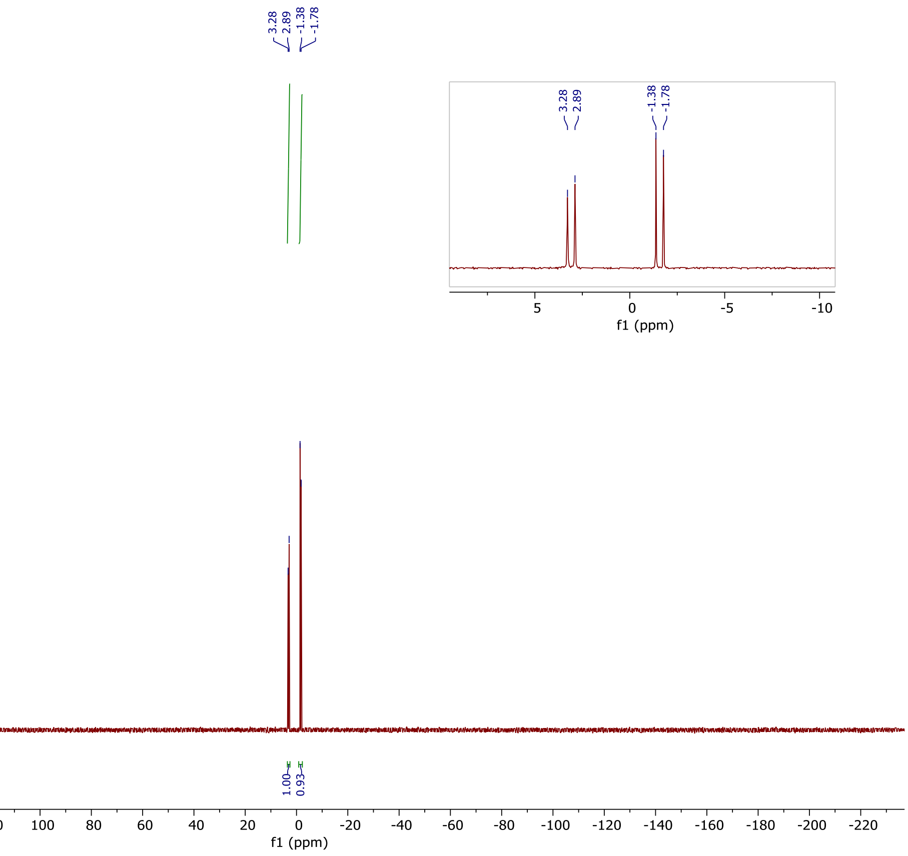

^{1}H NMR (501 MHz, $CDCl_3$)

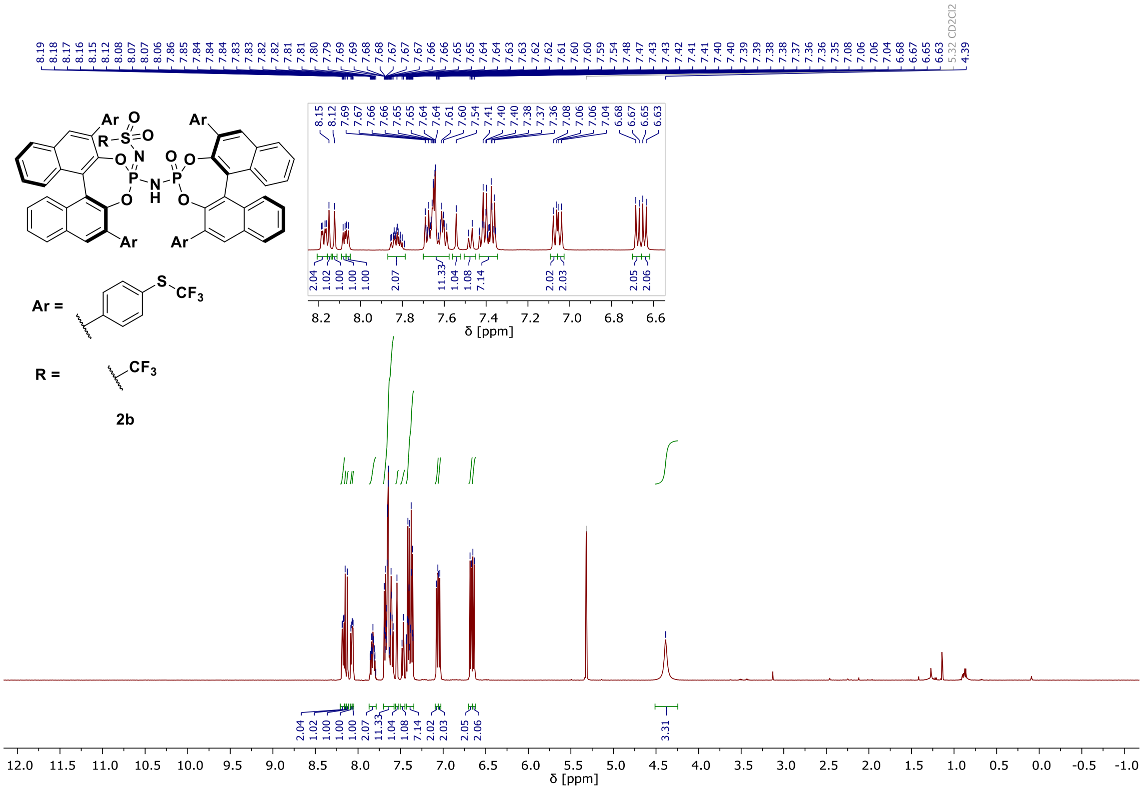

^{13}C NMR (125 MHz, CD_2Cl_2)

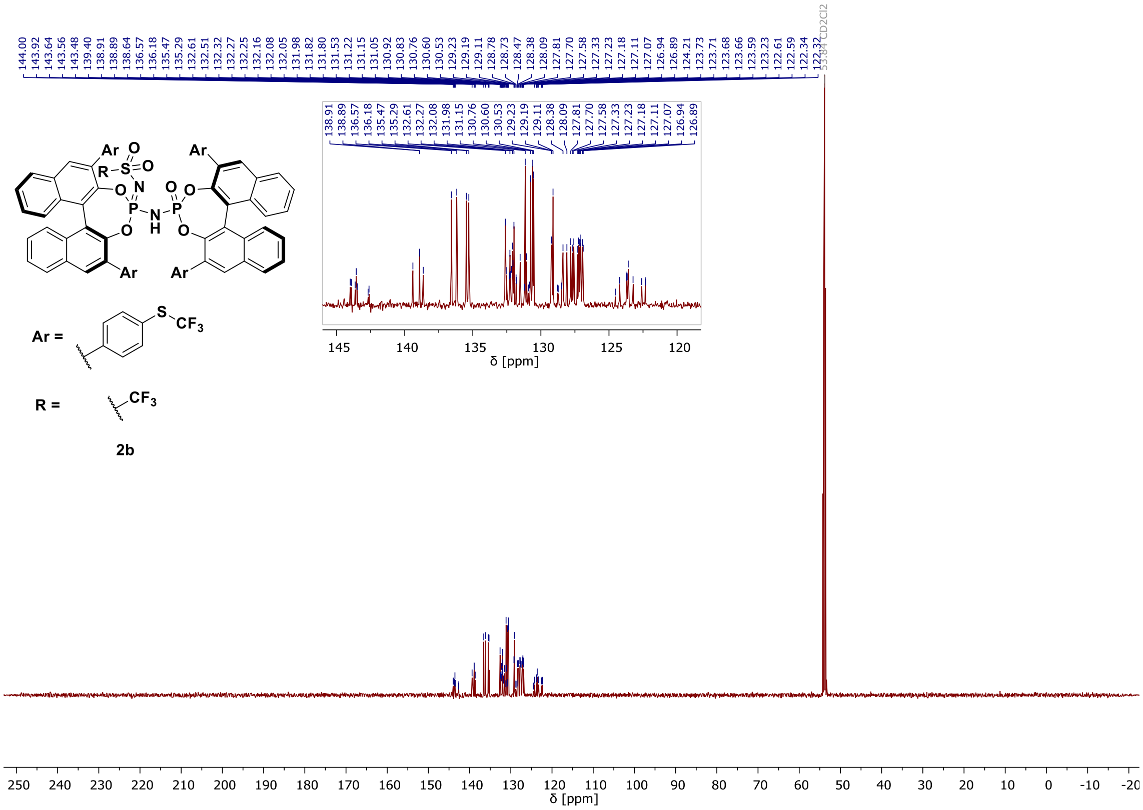

^{19}F-NMR (471 MHz, CD_2Cl_2)

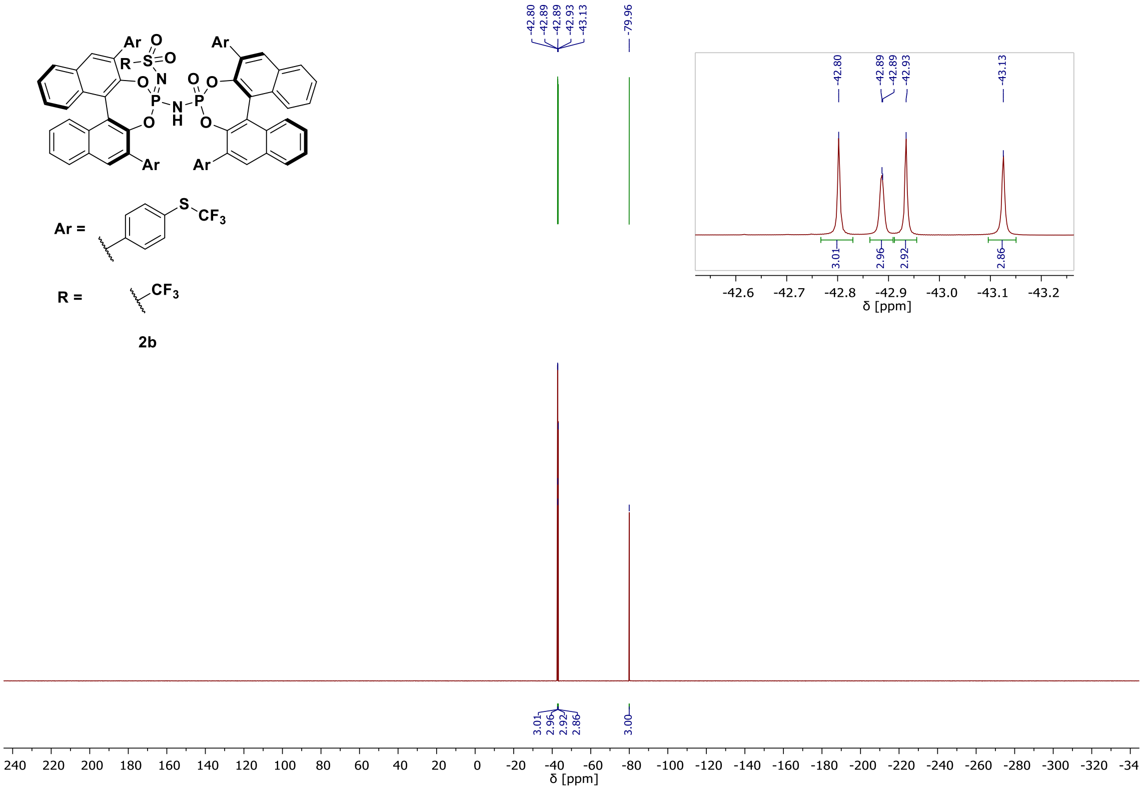

^{31}P-NMR (203 MHz, CD_2Cl_2)

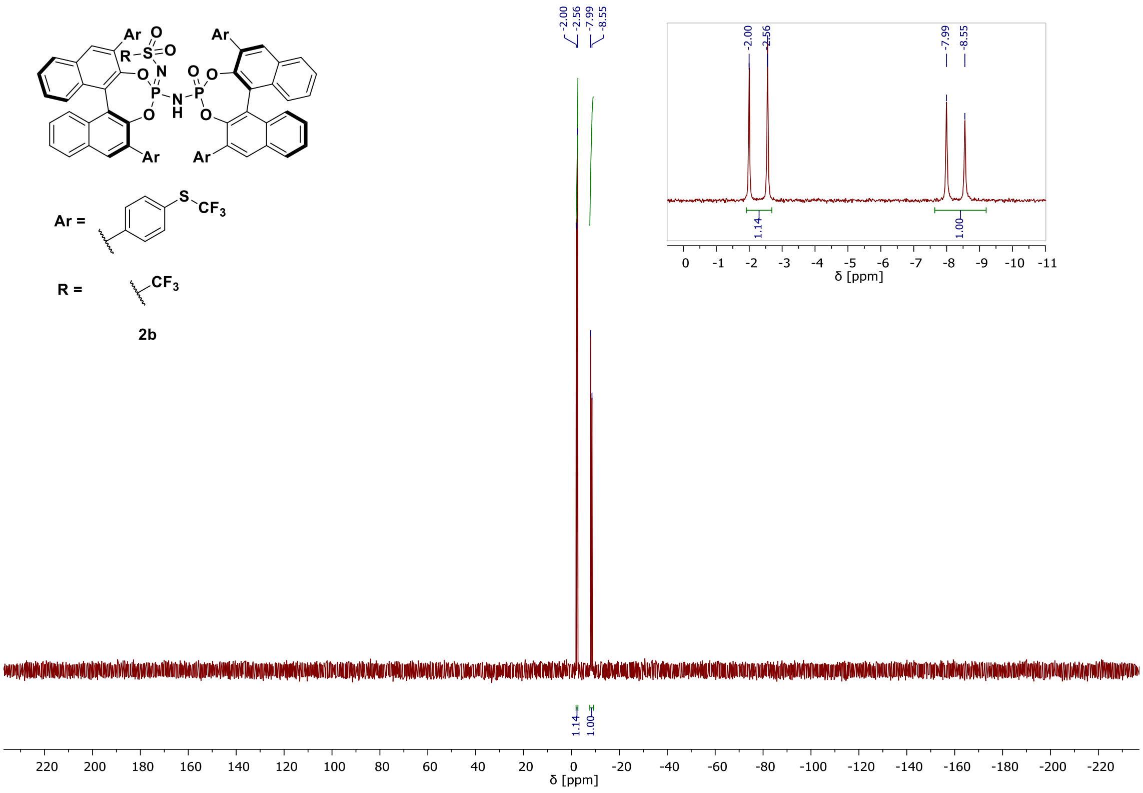

^{1}H-NMR (500 MHz, CD_2Cl_2)

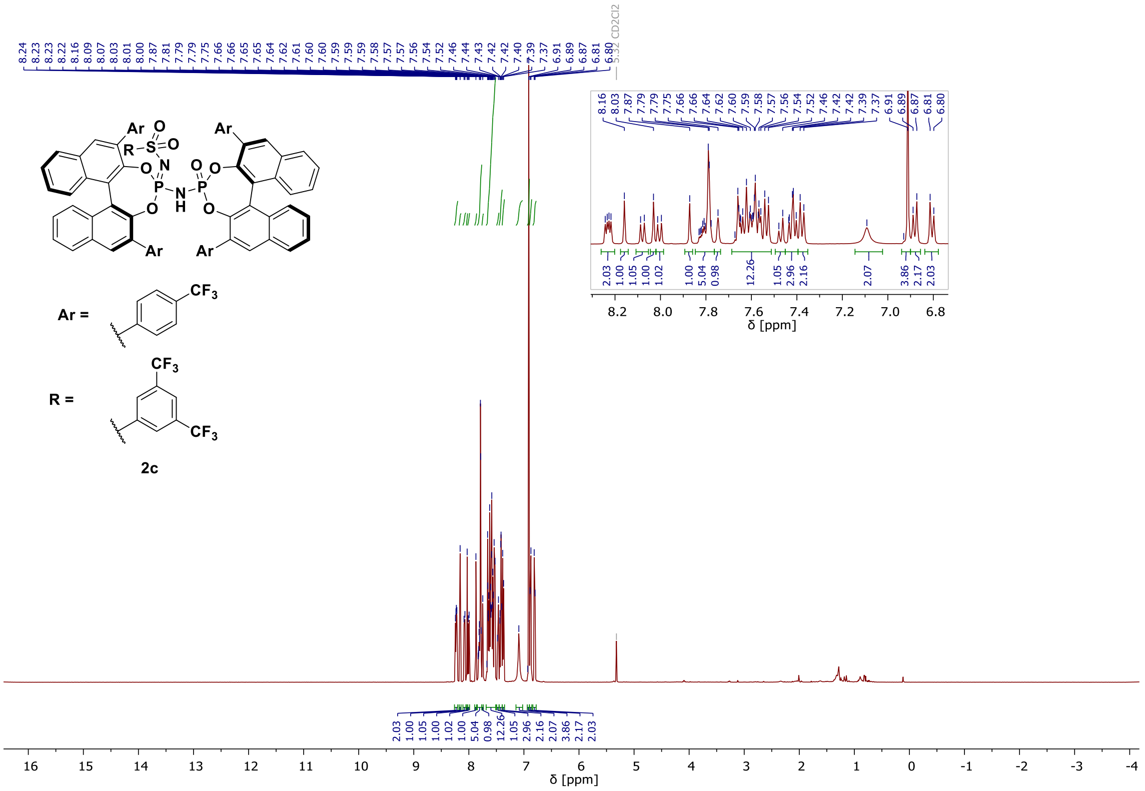

^{13}C NMR (126 MHz, CD_2Cl_2)

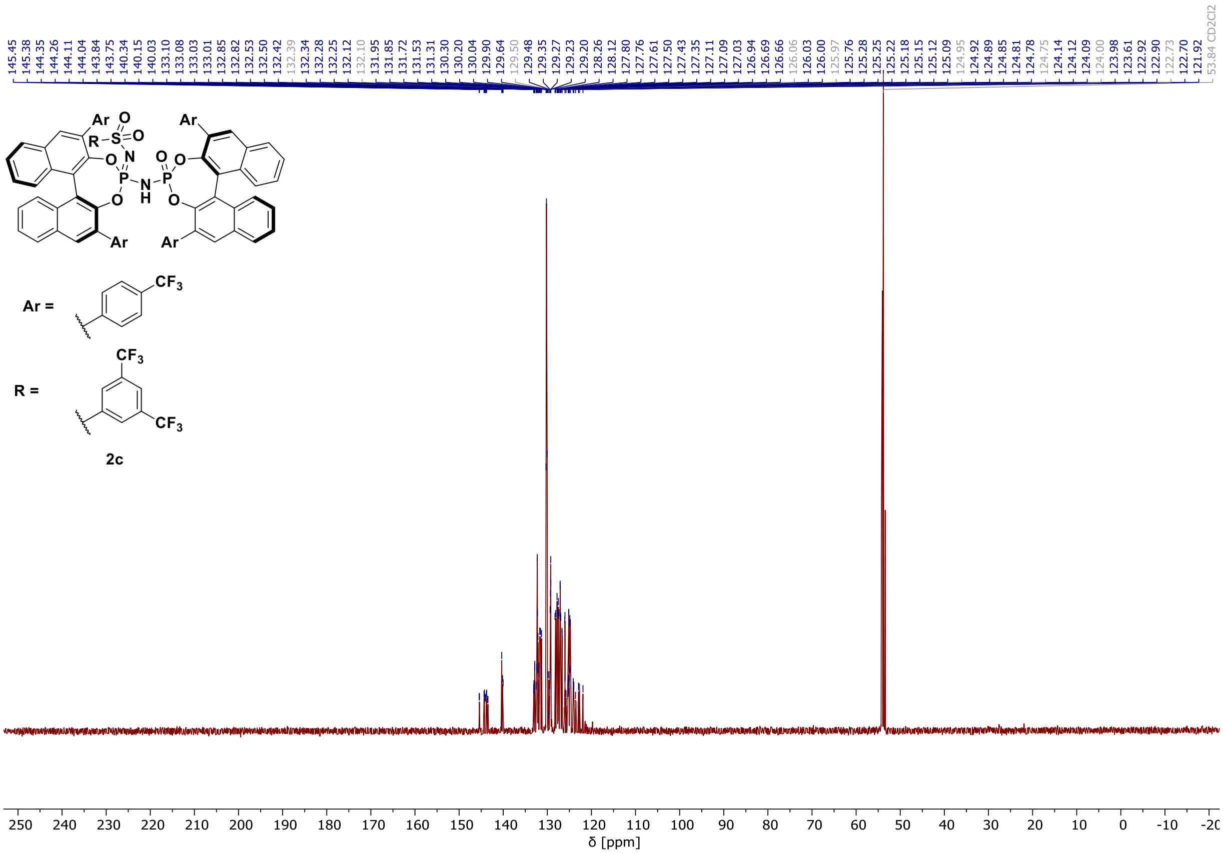

^{19}F-NMR (471 MHz, CD_2Cl_2)

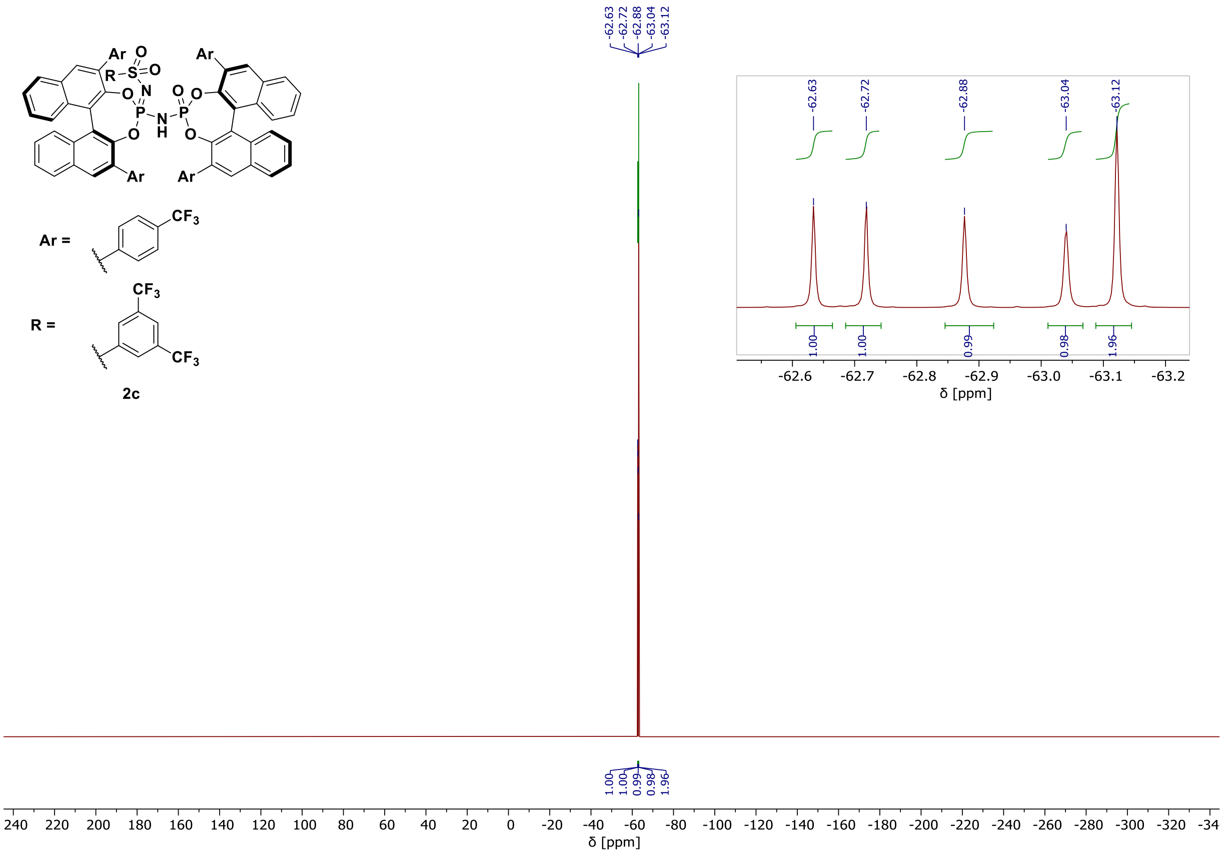

^{31}P-NMR (203 MHz, CD_2Cl_2)

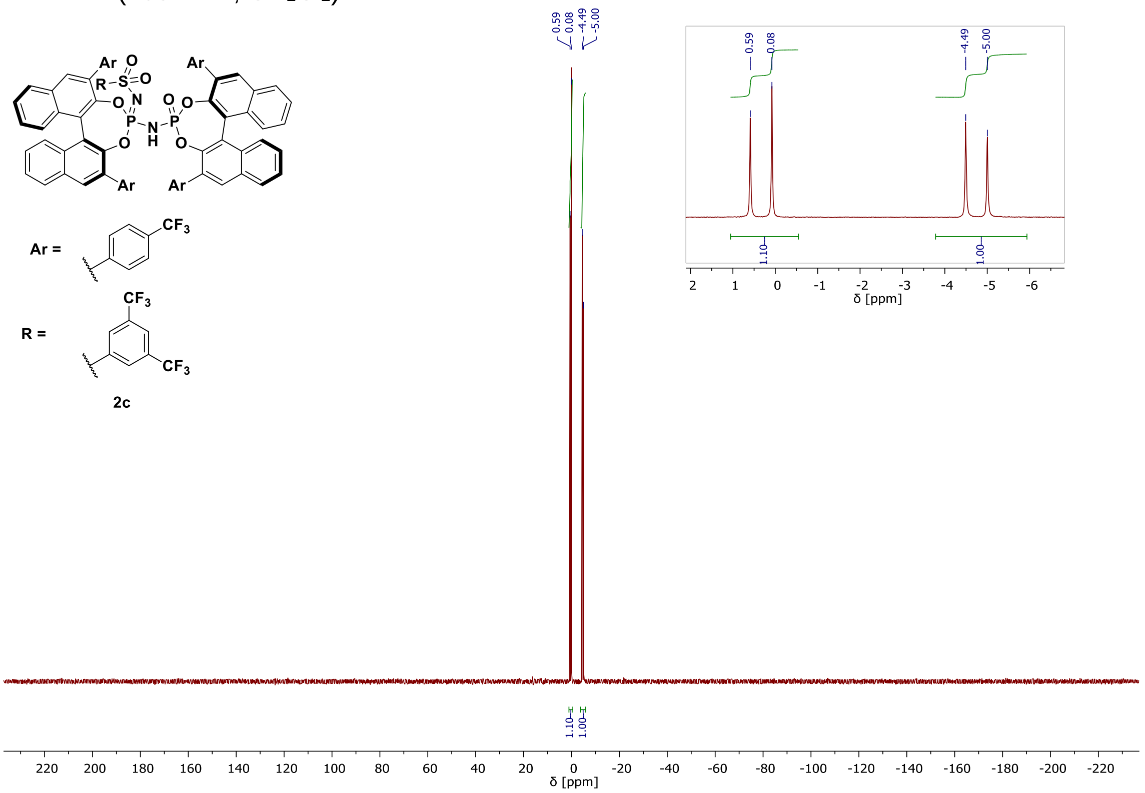

^{1}H-NMR (500 MHz, CD_2Cl_2)

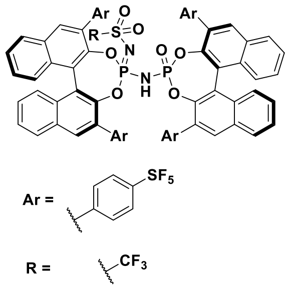

2d

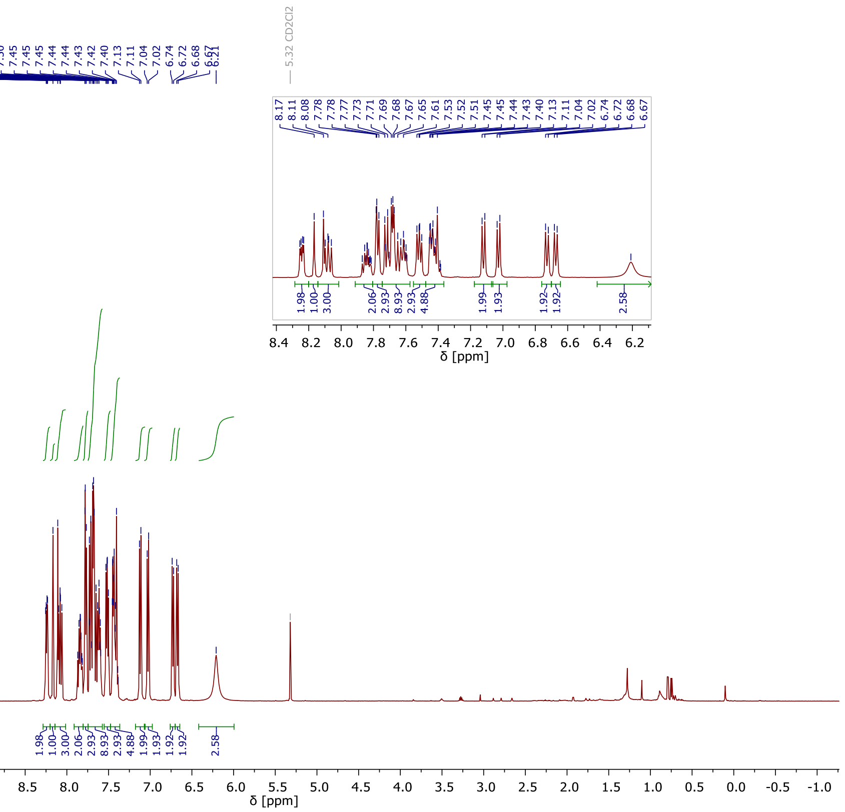

^{13}C-NMR (125 MHz, CD_2Cl_2)

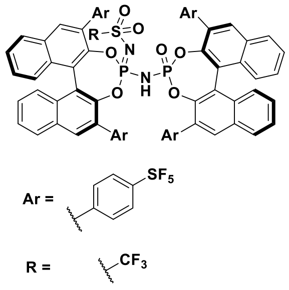

2d

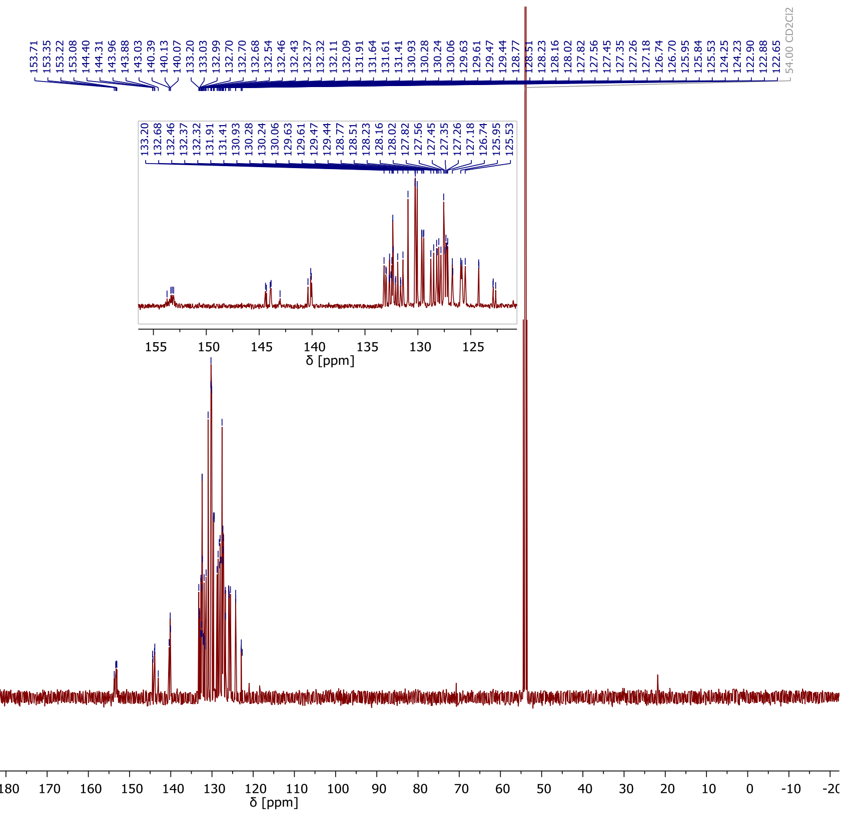

^{19}F NMR (471 MHz, CD_2Cl_2)

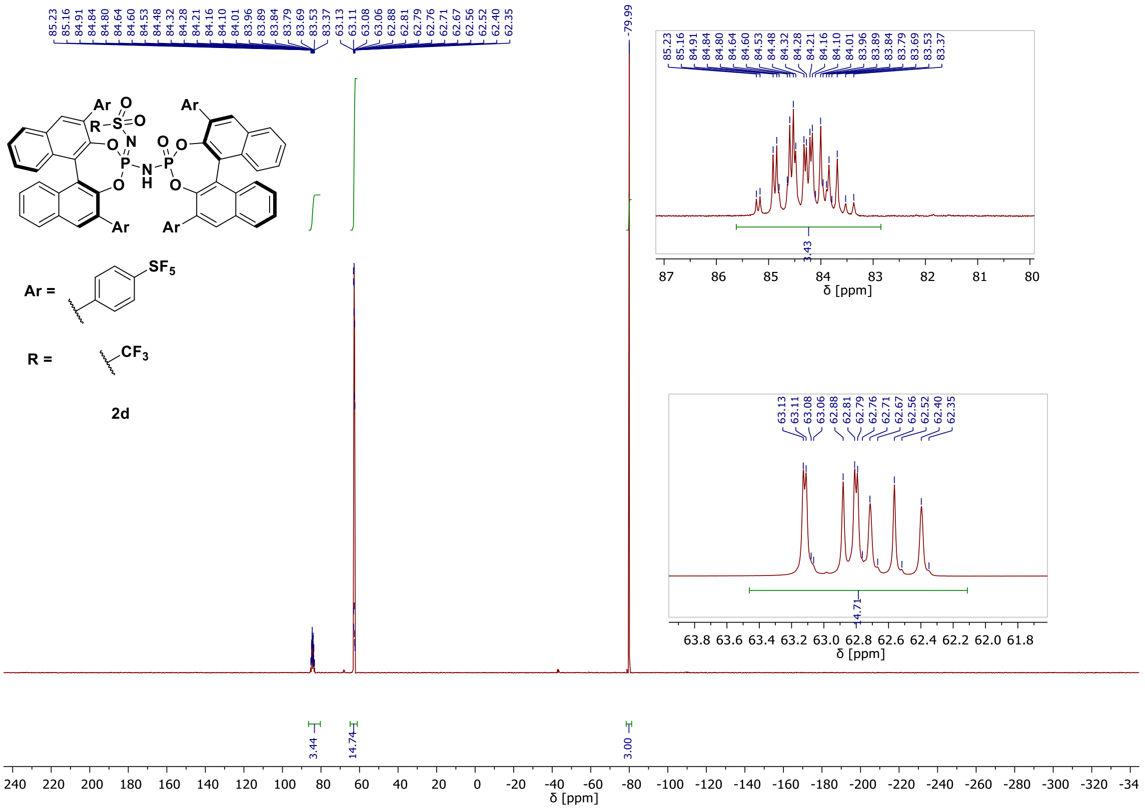

^{31}P-NMR (203 MHz, CD_2Cl_2)

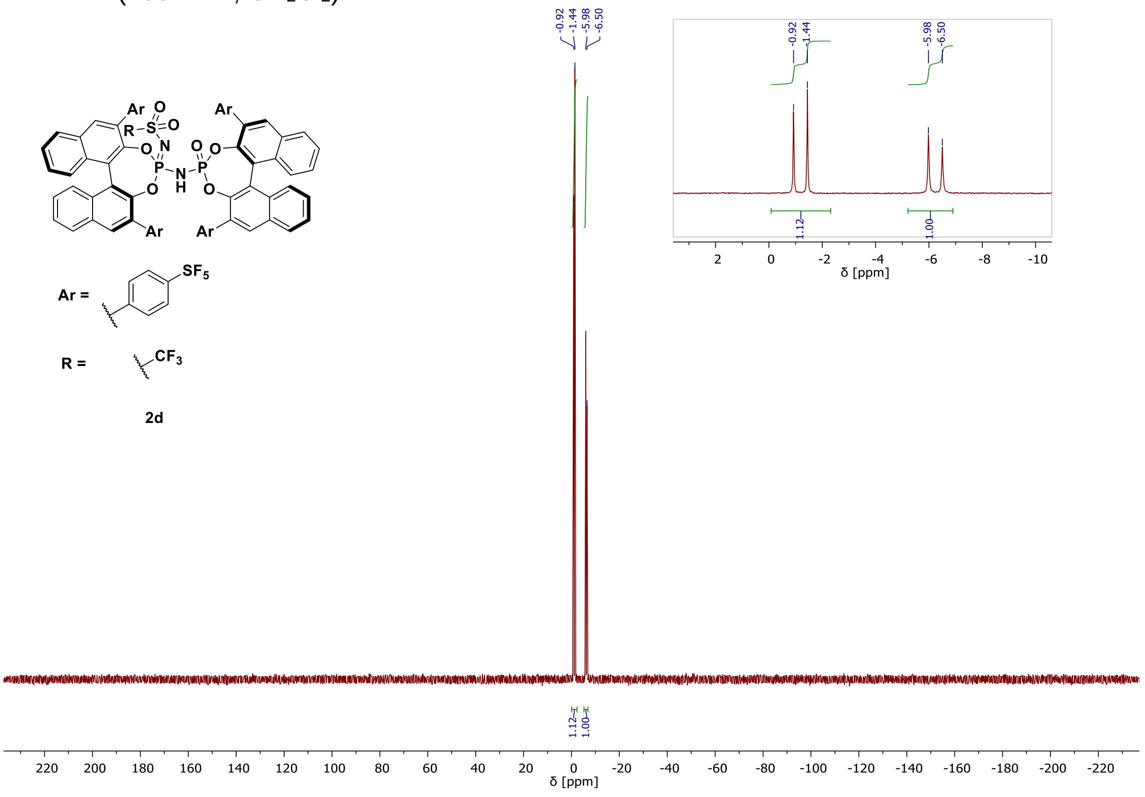

^{1}H NMR (501 MHz, CD_2Cl_2)

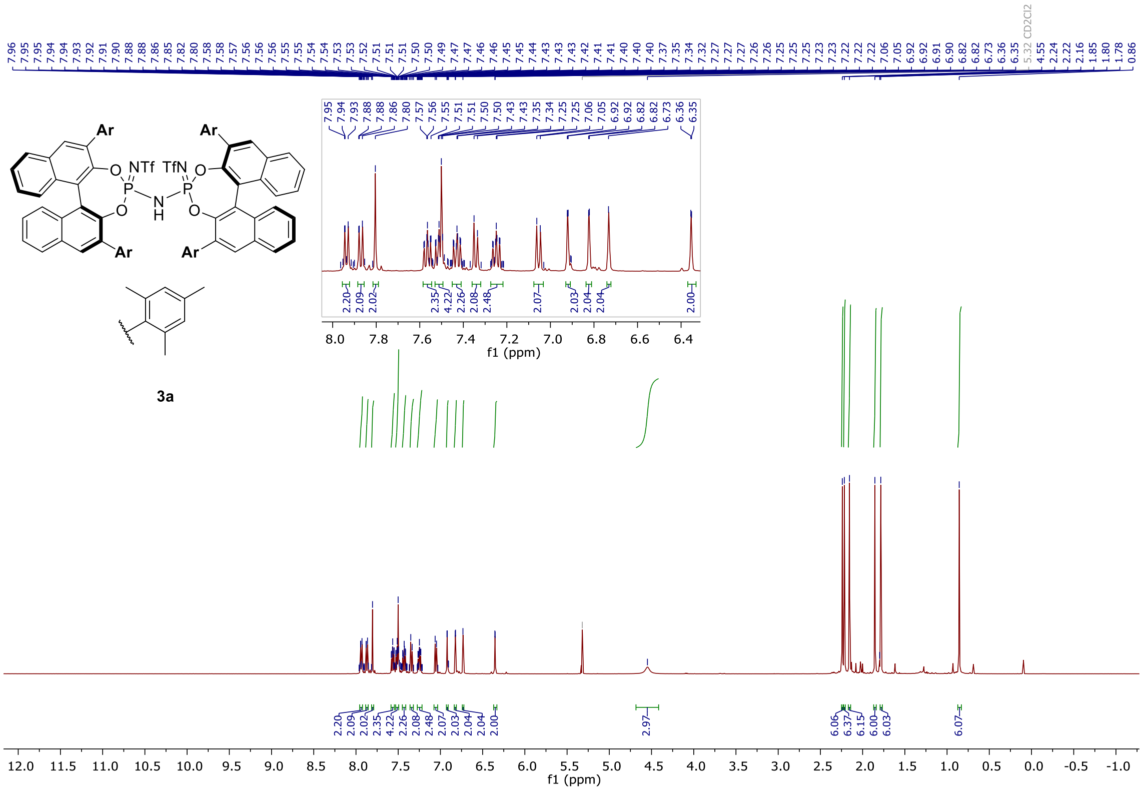

^{13}C NMR (126 MHz, CD_2Cl_2)

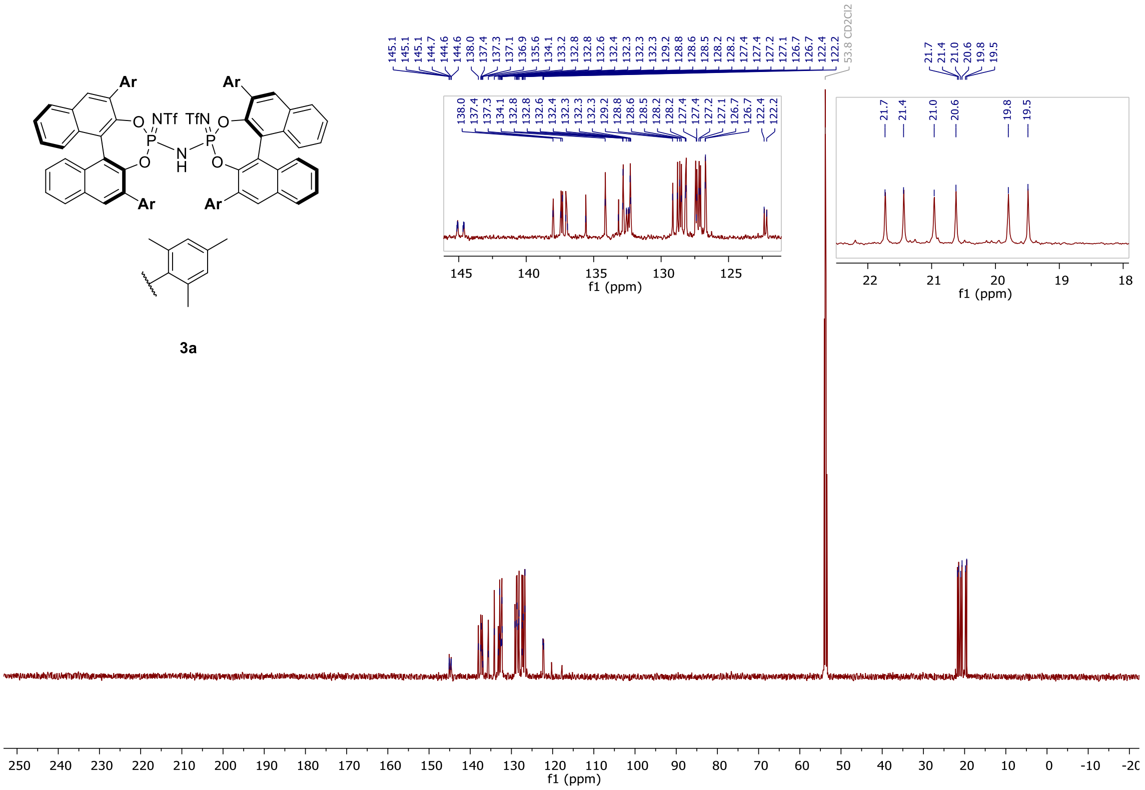

^{19}F NMR (471 MHz, CD_2Cl_2)

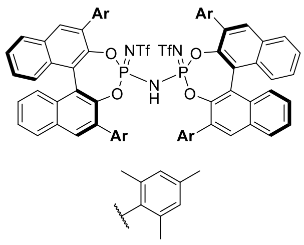

3a

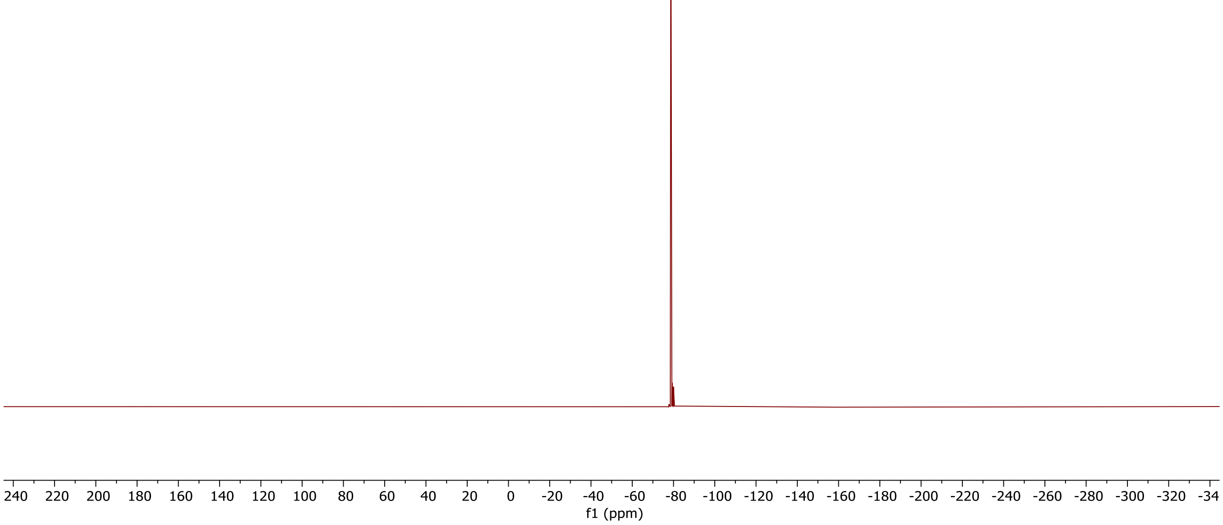

^{31}P NMR (203 MHz, CD_2Cl_2)

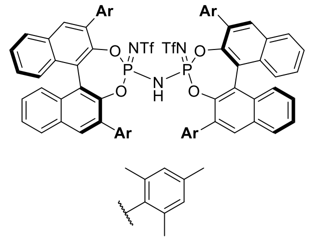

3a

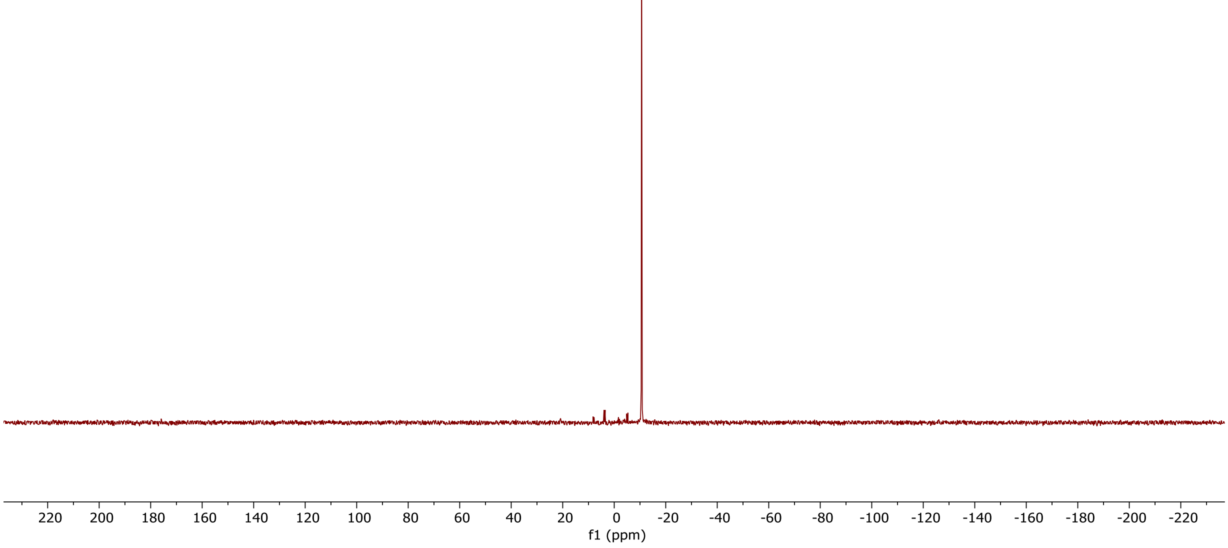

^{1}H NMR (600 MHz, CD_2Cl_2)

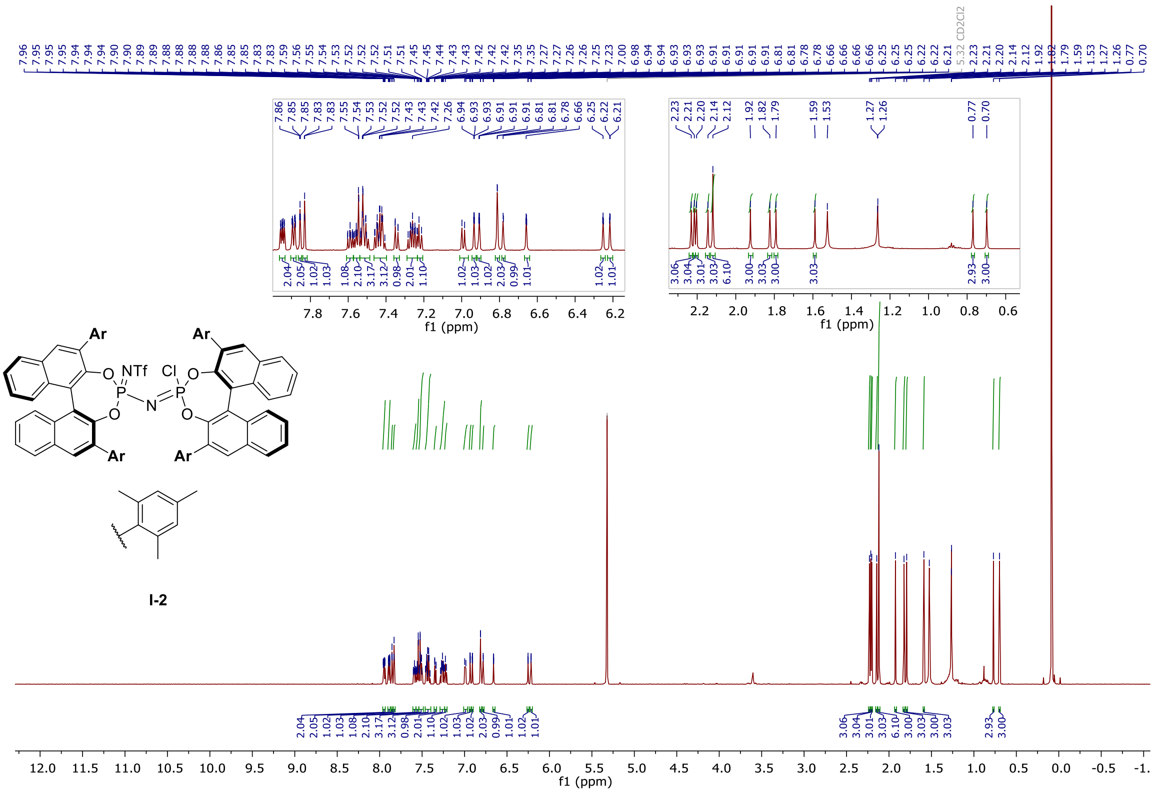

^{13}C NMR (151 MHz, CD_2Cl_2)

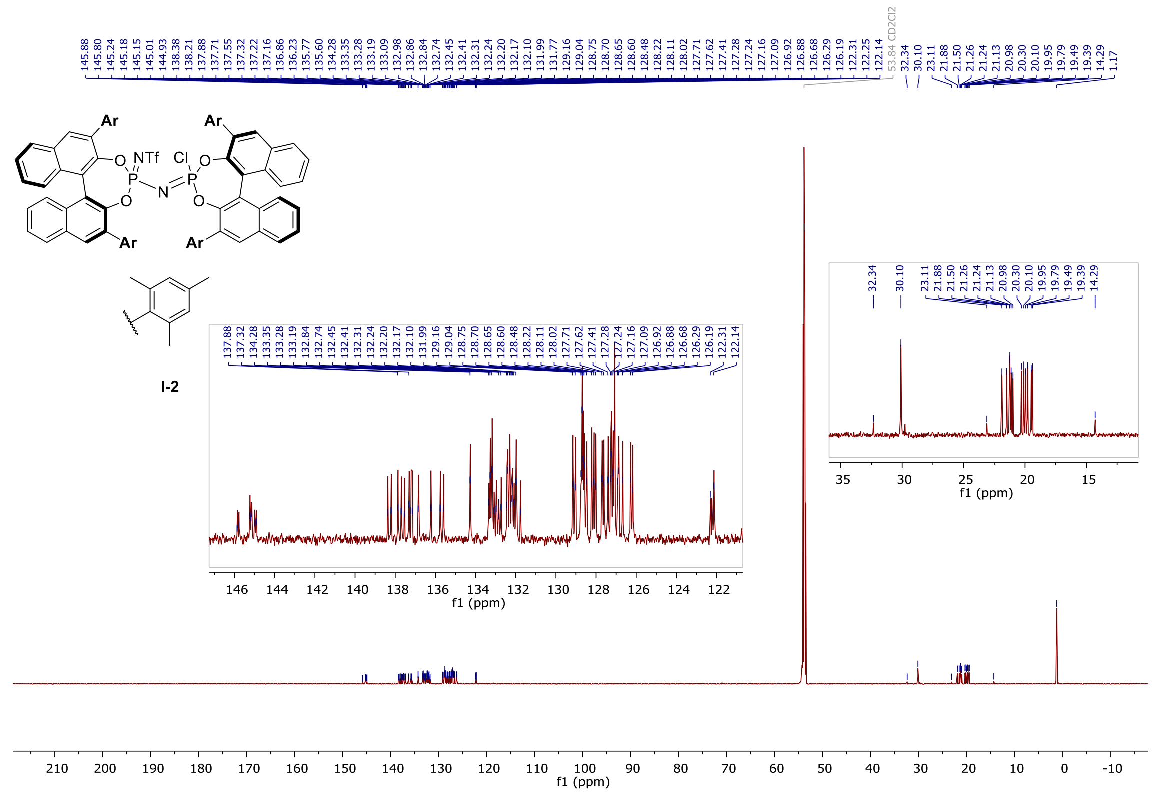

^{19}F NMR (565 MHz, CD_2Cl_2)

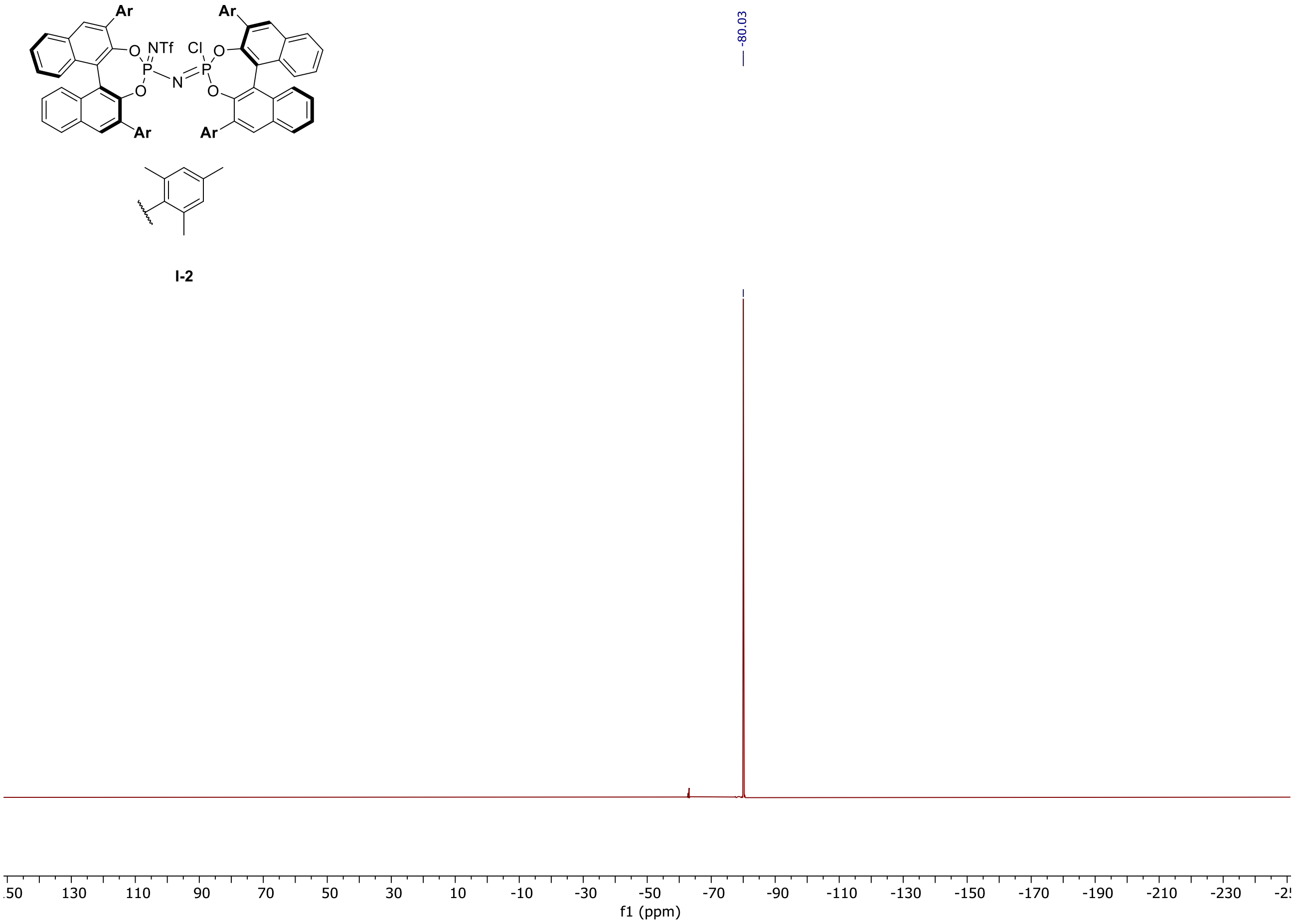

^{31}P NMR (243 MHz, CD_2Cl_2)

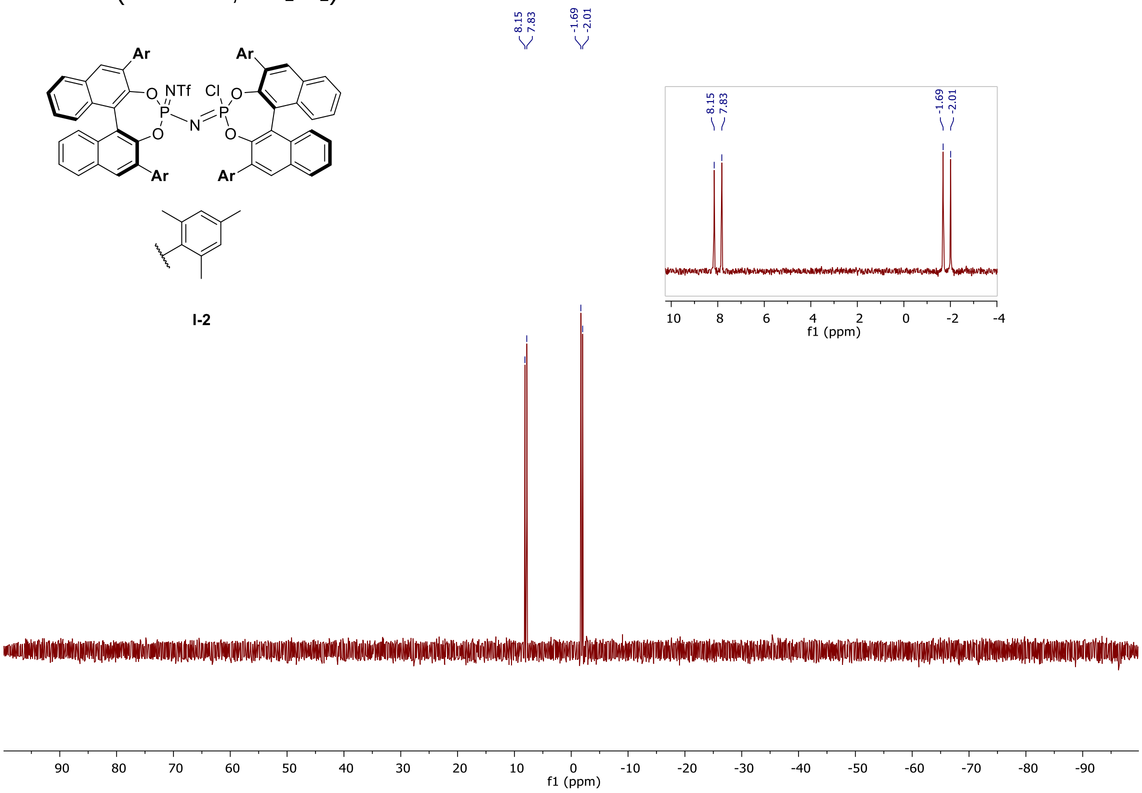

^{1}H NMR (600 MHz, CD_2Cl_2)

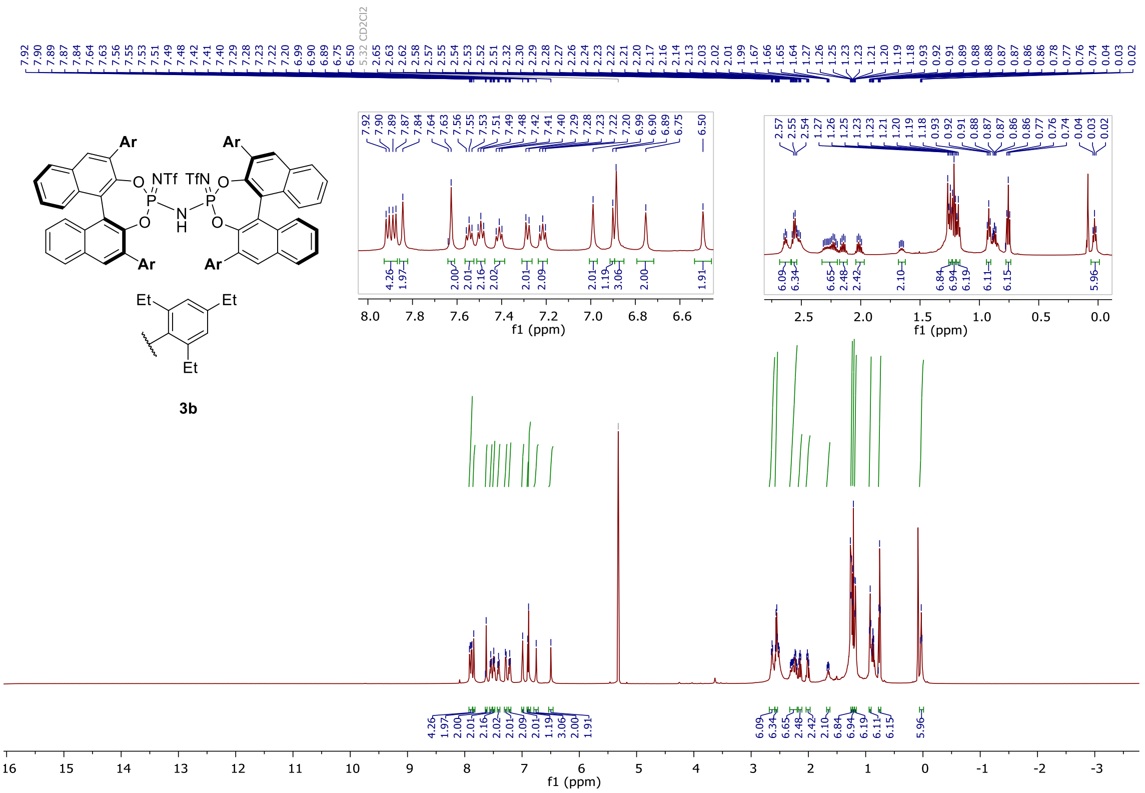

^{13}C NMR (151 MHz, CD_2Cl_2)

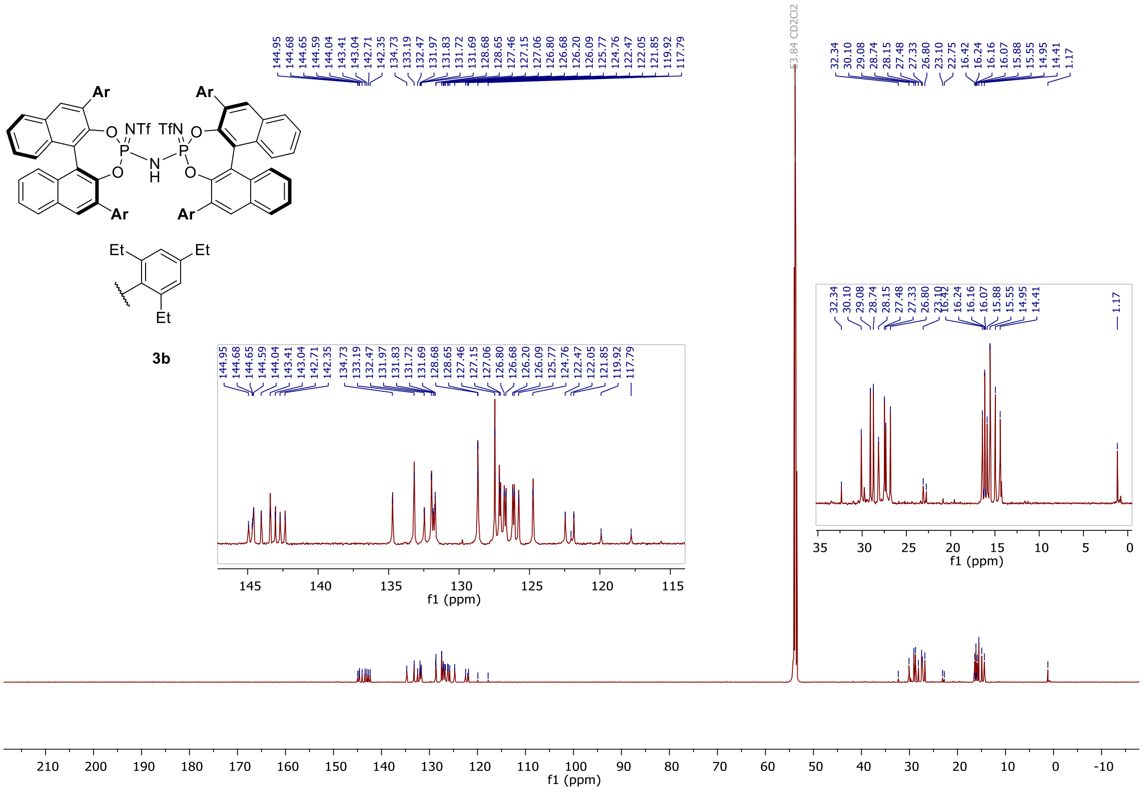

^{19}F NMR (565 MHz, CD_2Cl_2)

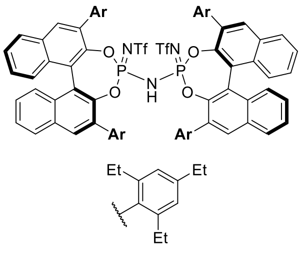

3b

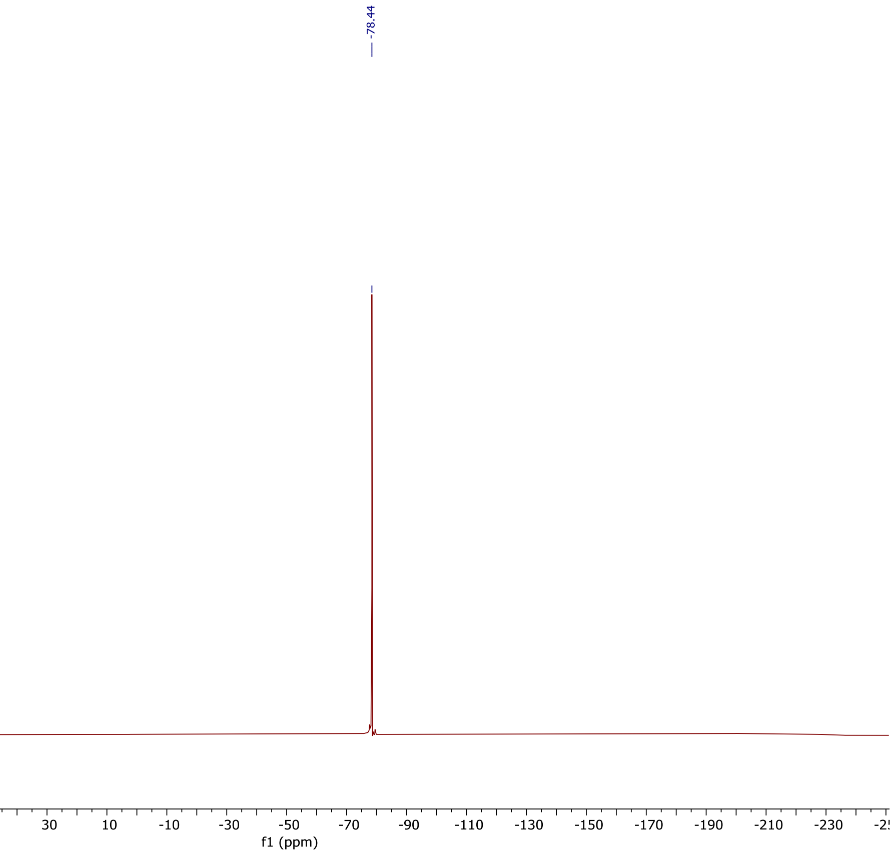

^{31}P NMR (243 MHz, CD_2Cl_2),

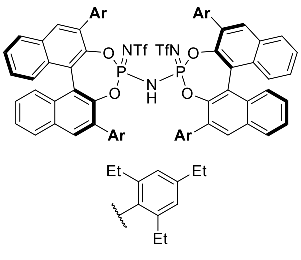

3b

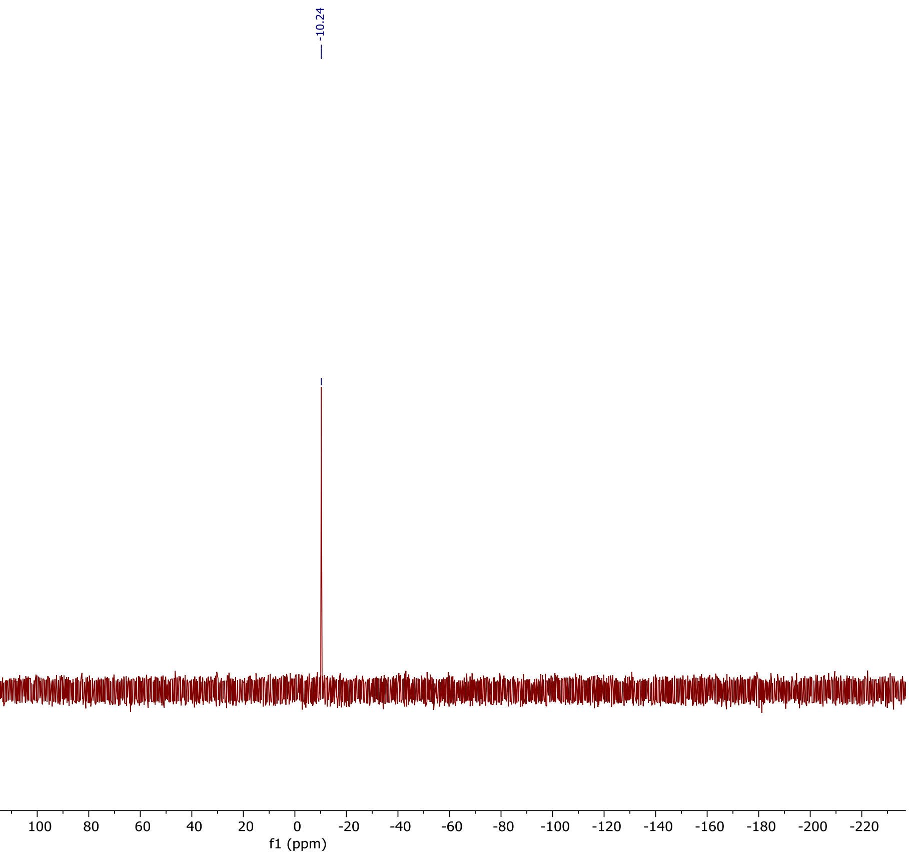

^1H NMR (501 MHz, CD_2Cl_2)

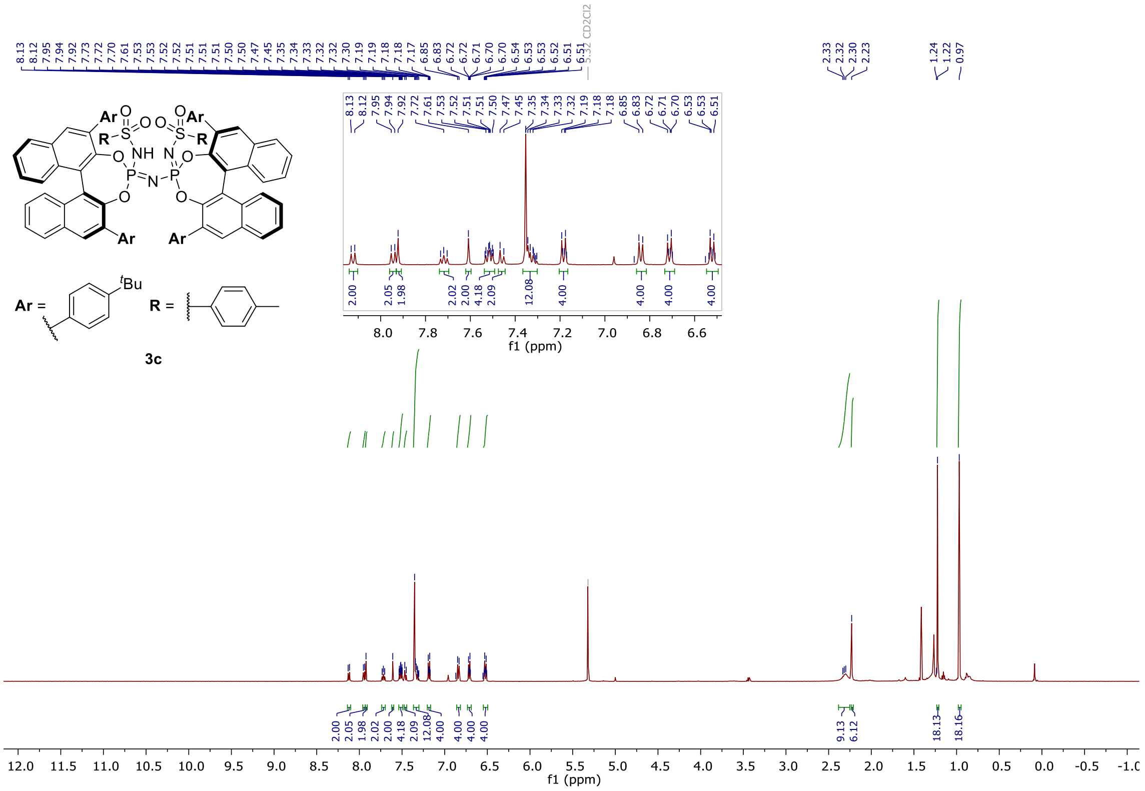

^{13}C NMR (126 MHz, $CDCl_3$)

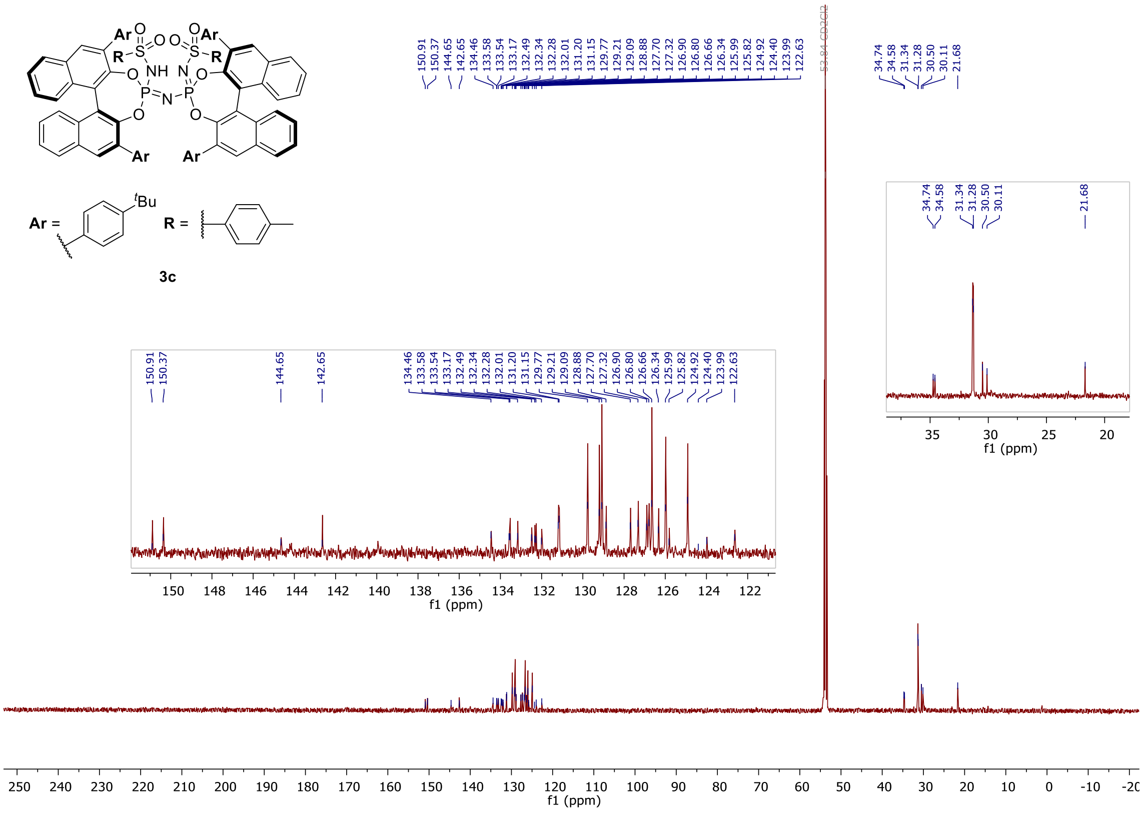

^{31}P NMR (203 MHz, $CDCl_3$)

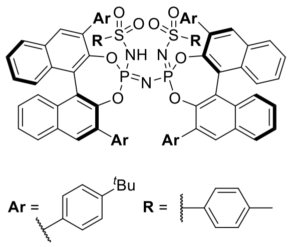

3c

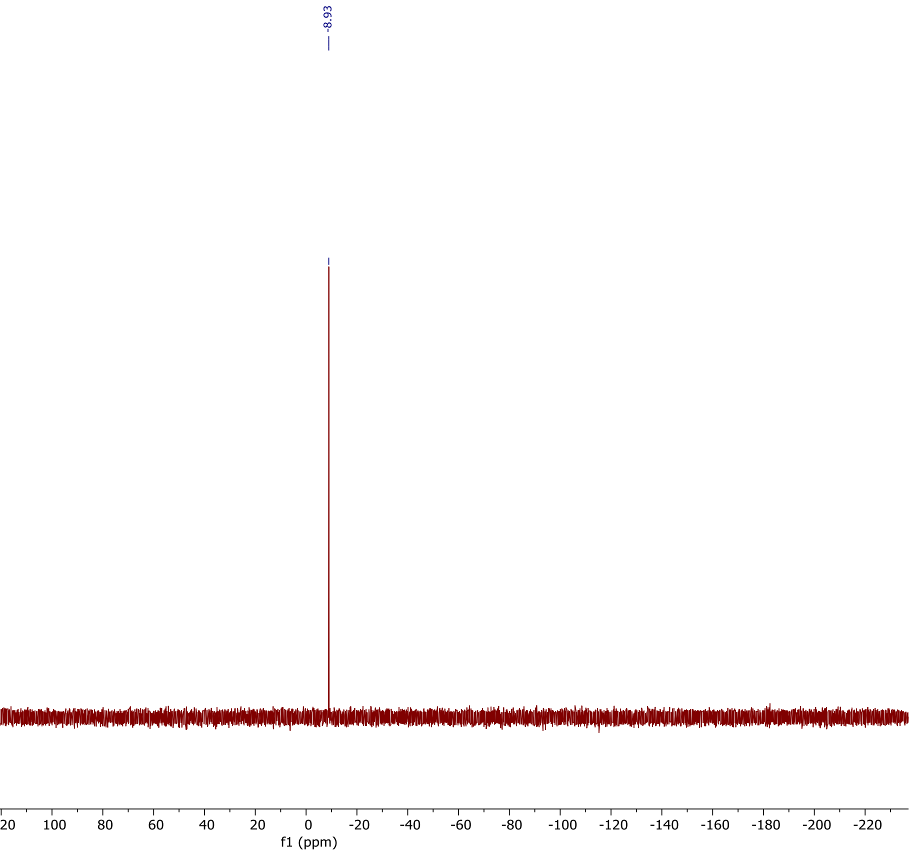

^{1}H NMR (501 MHz, CD_2Cl_2)

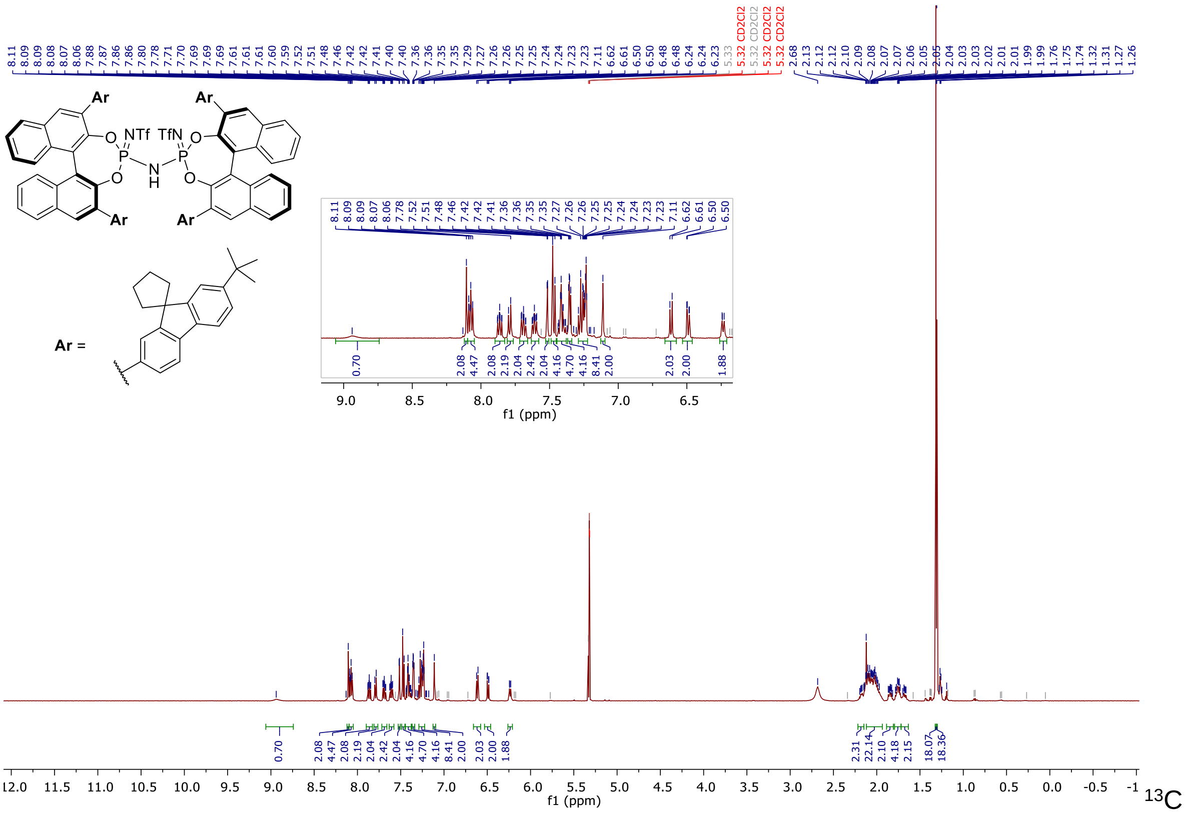

^{13}C-NMR (126 MHz, $CDCl_3$)

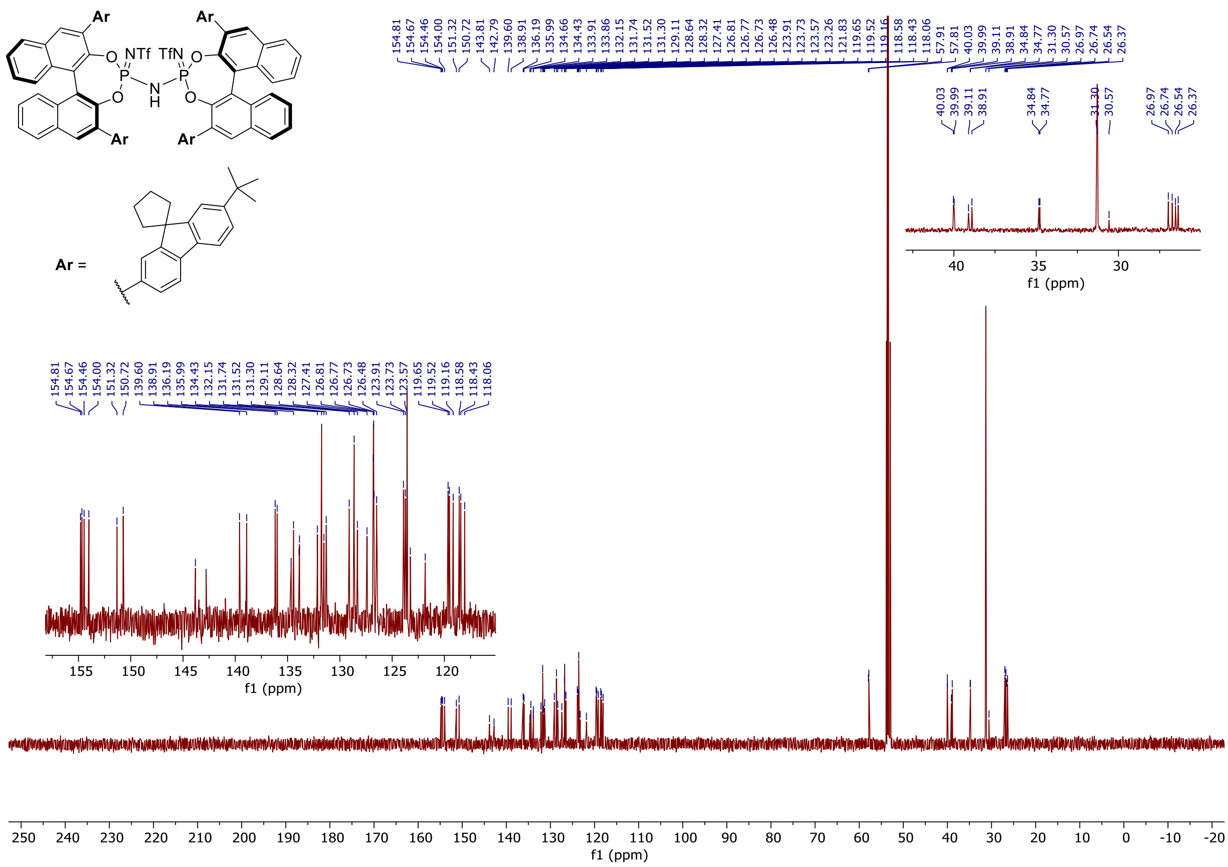

^{19}F NMR (565 MHz, CD_2Cl_2)

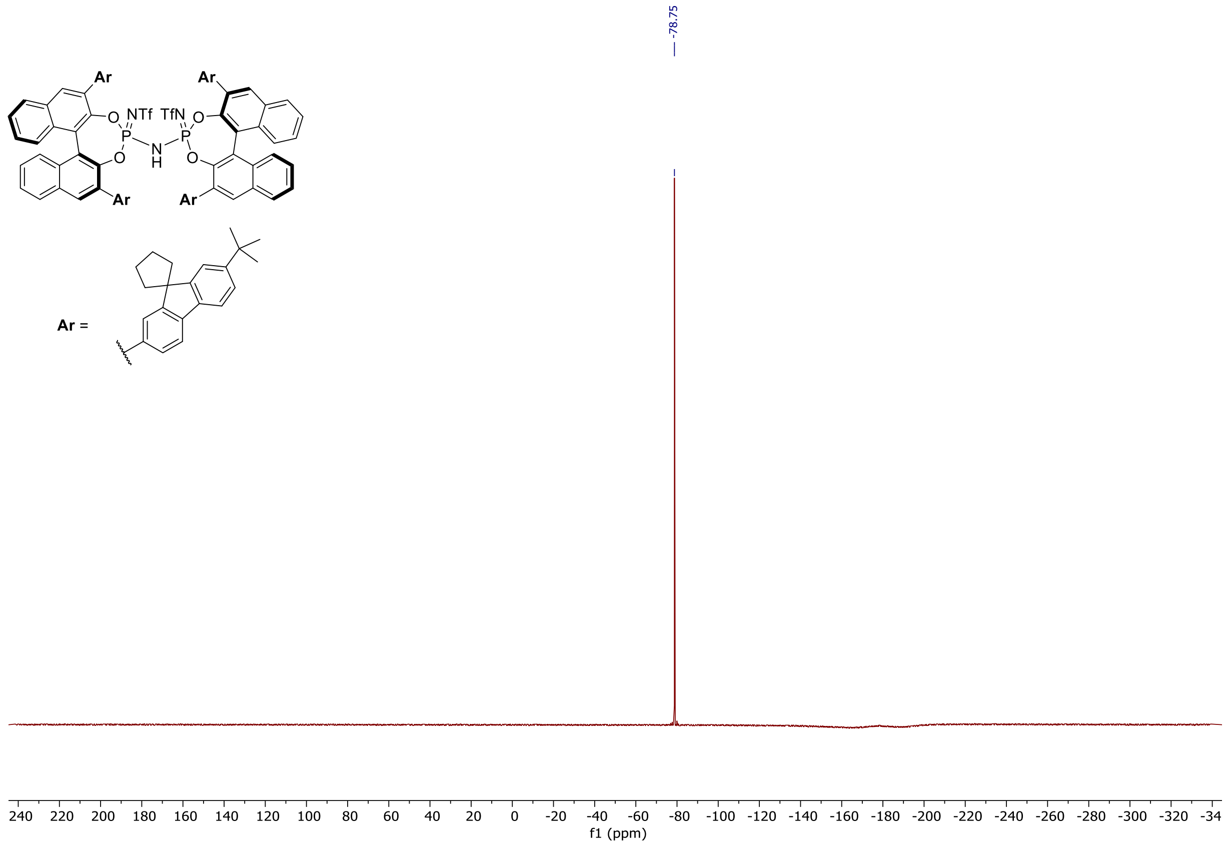

^{31}P NMR (203 MHz, $CDCl_3$)

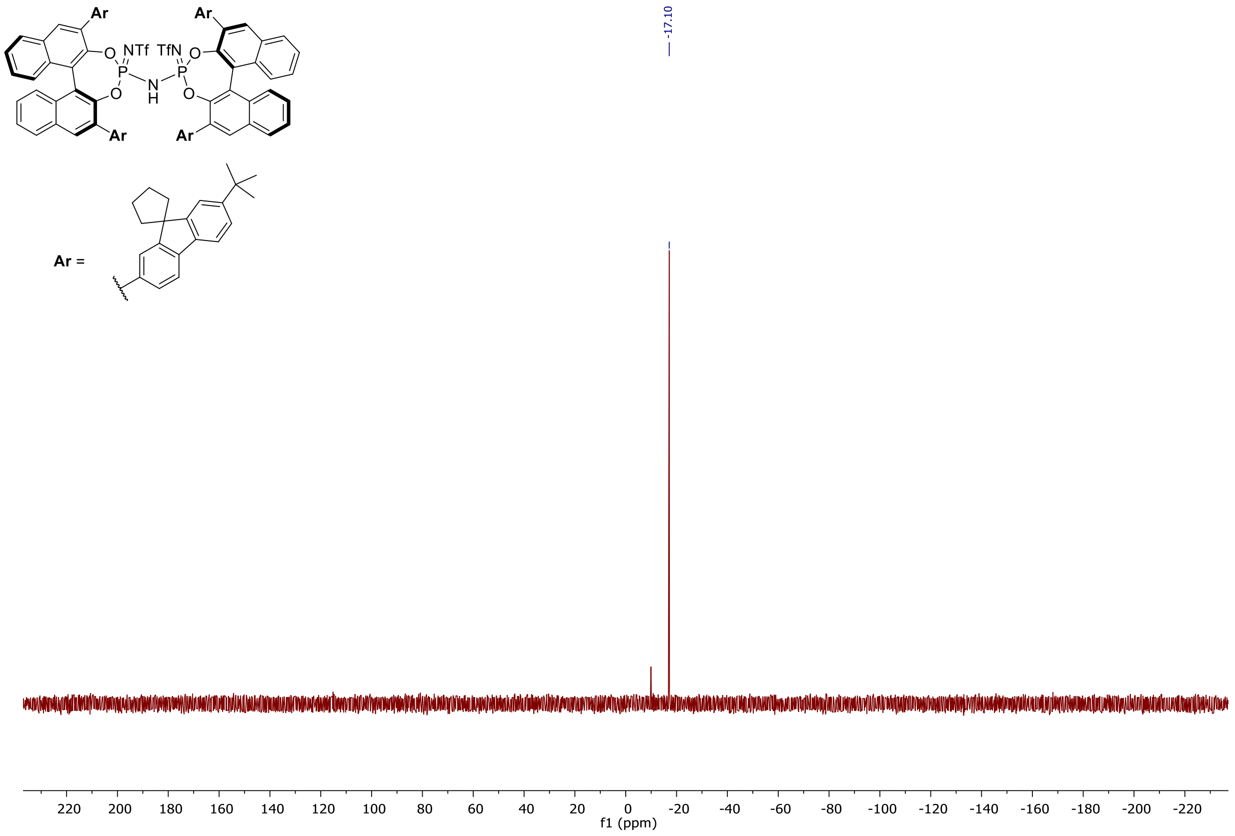

^{1}H NMR (501 MHz, CD_3CN)

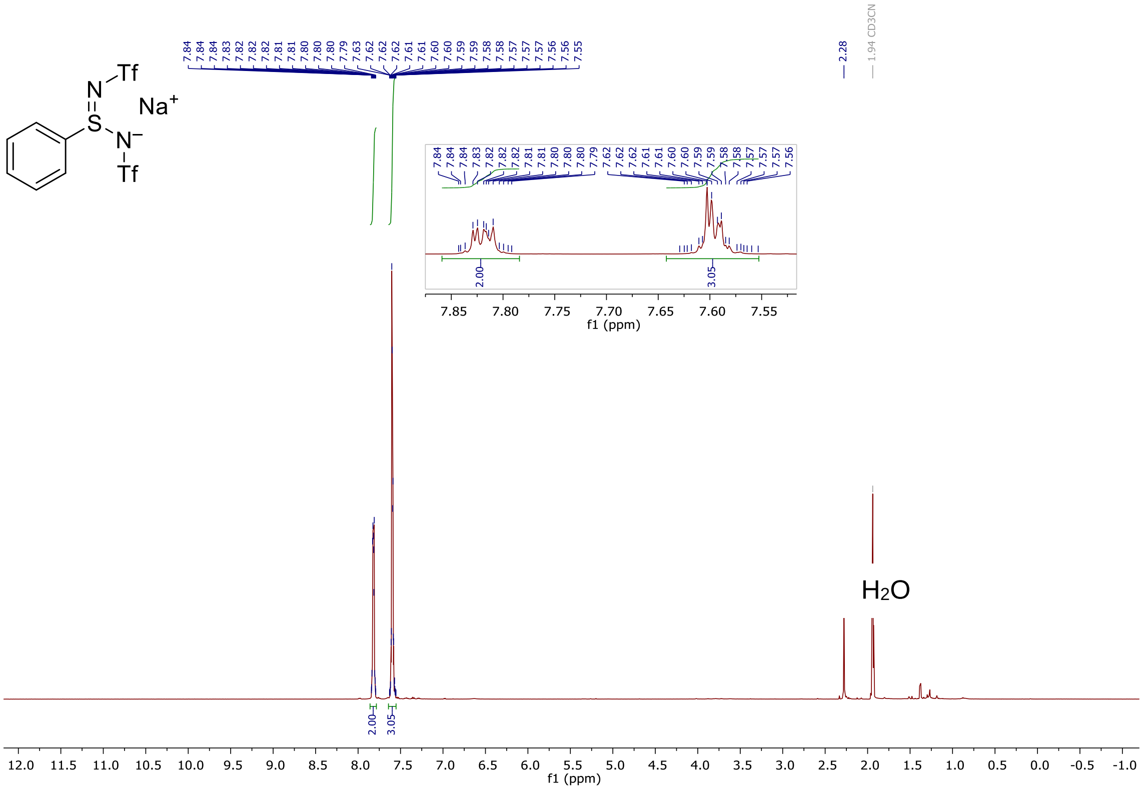

^{13}C NMR (126 MHz, CD_3CN)

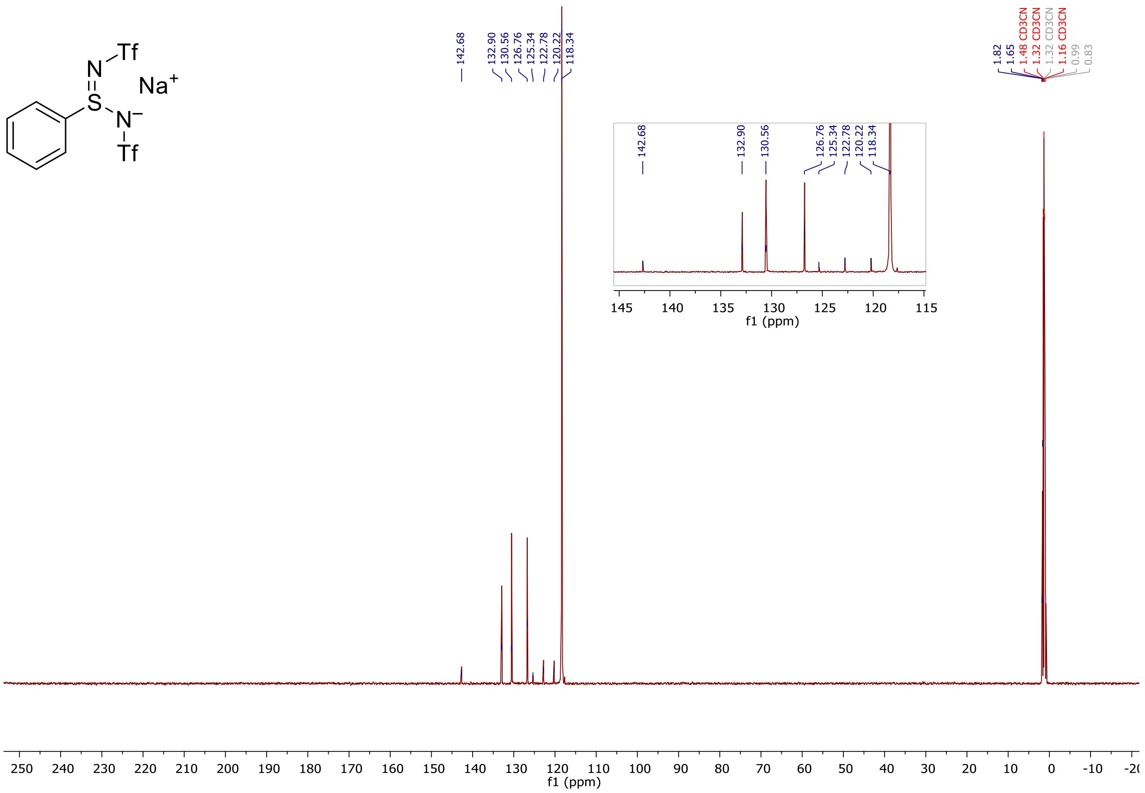

^{19}F NMR (565 MHz, CD_3CN)

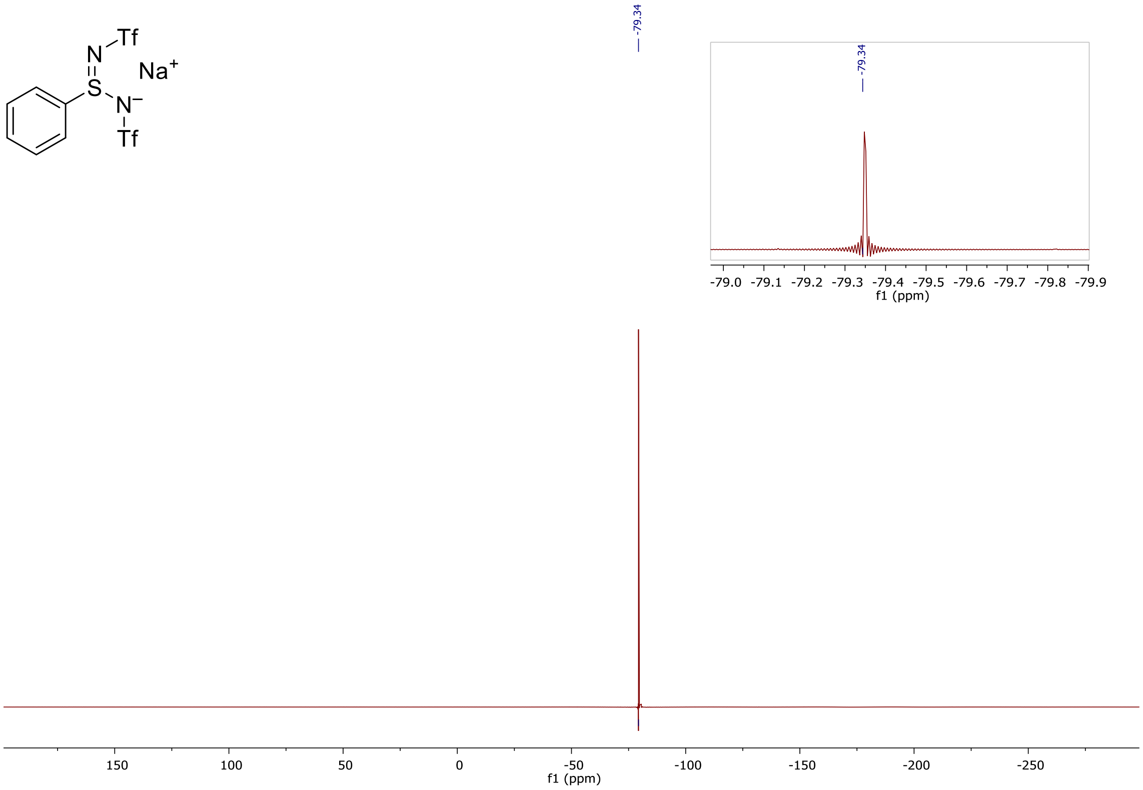

^{1}H NMR (501 MHz, CD_3CN)

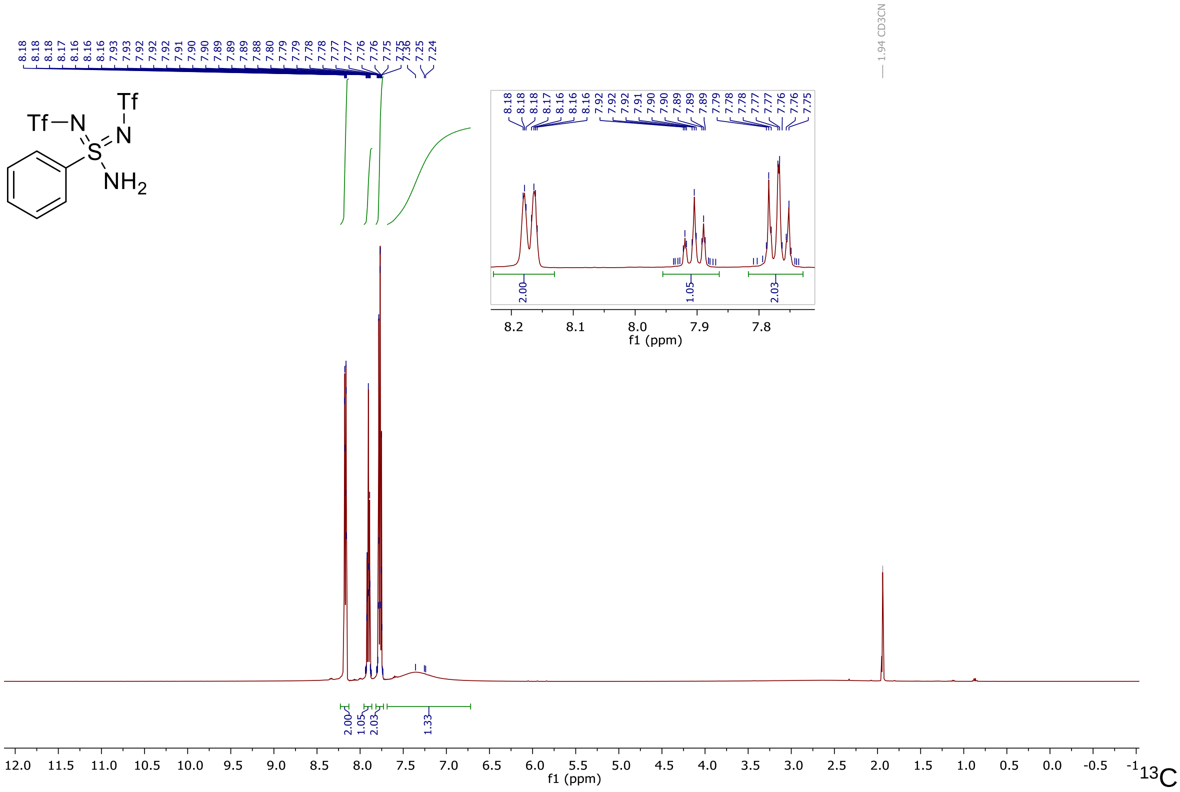

^{13}C NMR (126 MHz, CD_3CN)

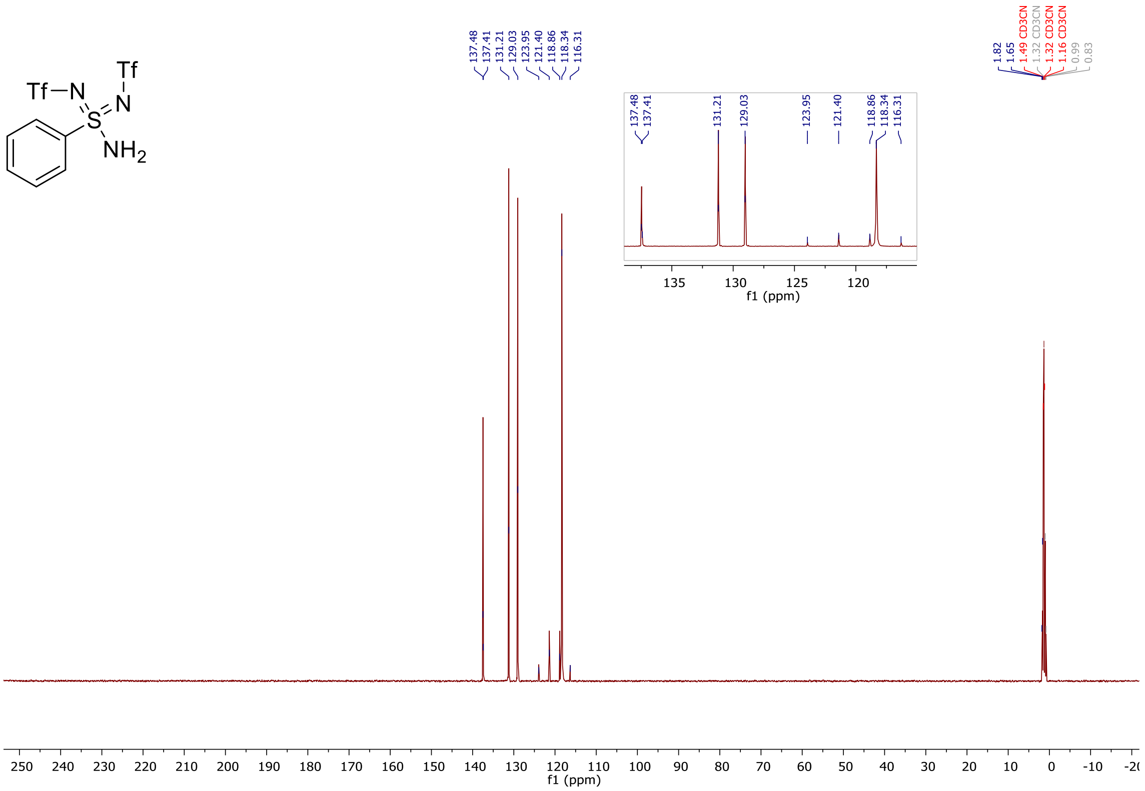

^{19}F NMR (565 MHz, CD_3CN)

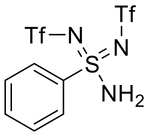

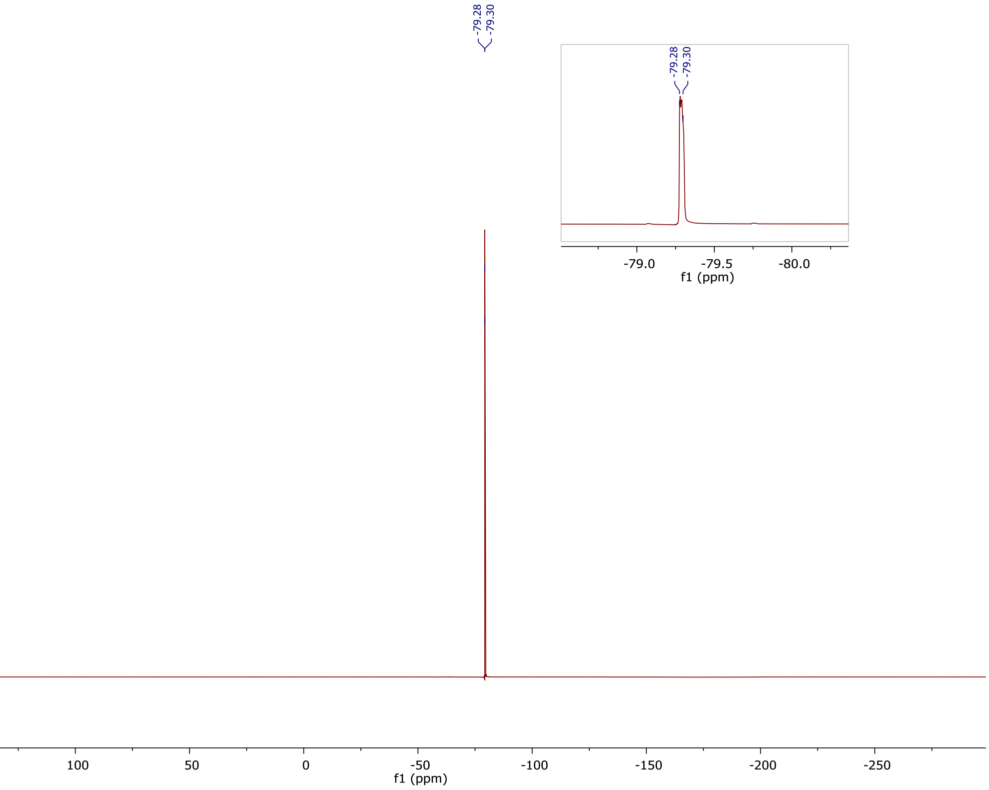

^{1}H NMR (501 MHz, CD_2Cl_2)

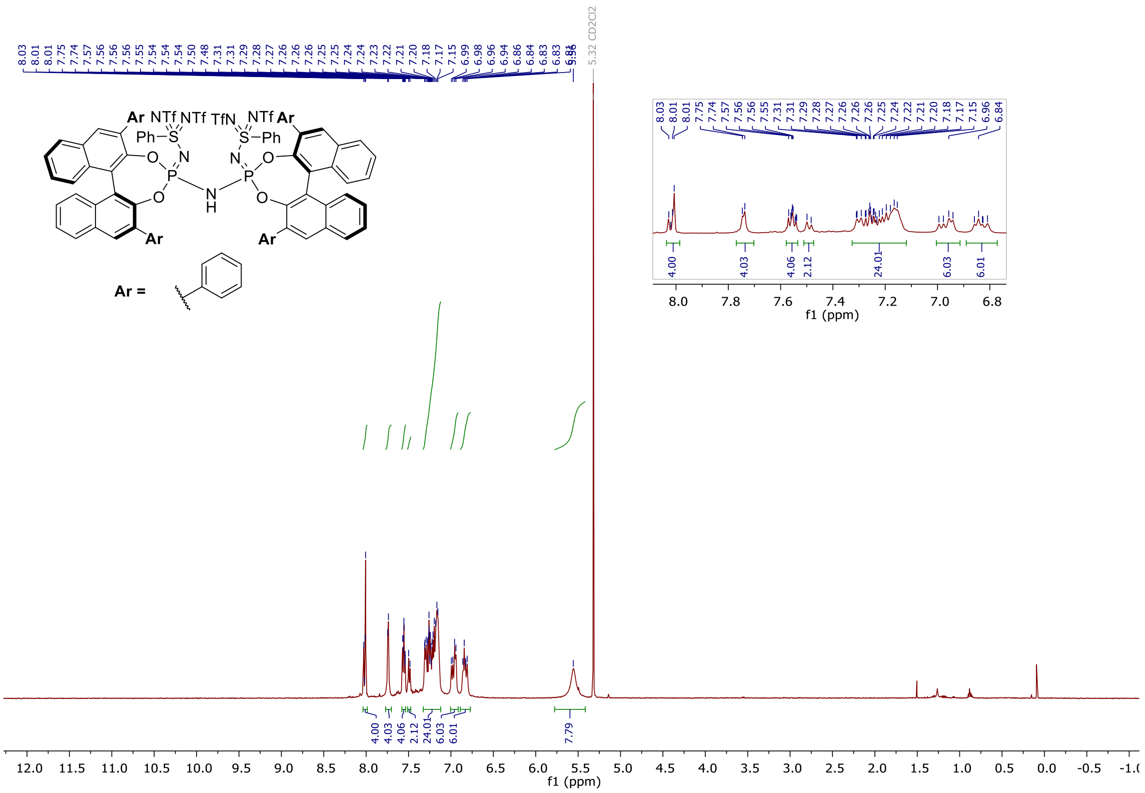

^{13}C NMR (151 MHz, CD_2Cl_2)

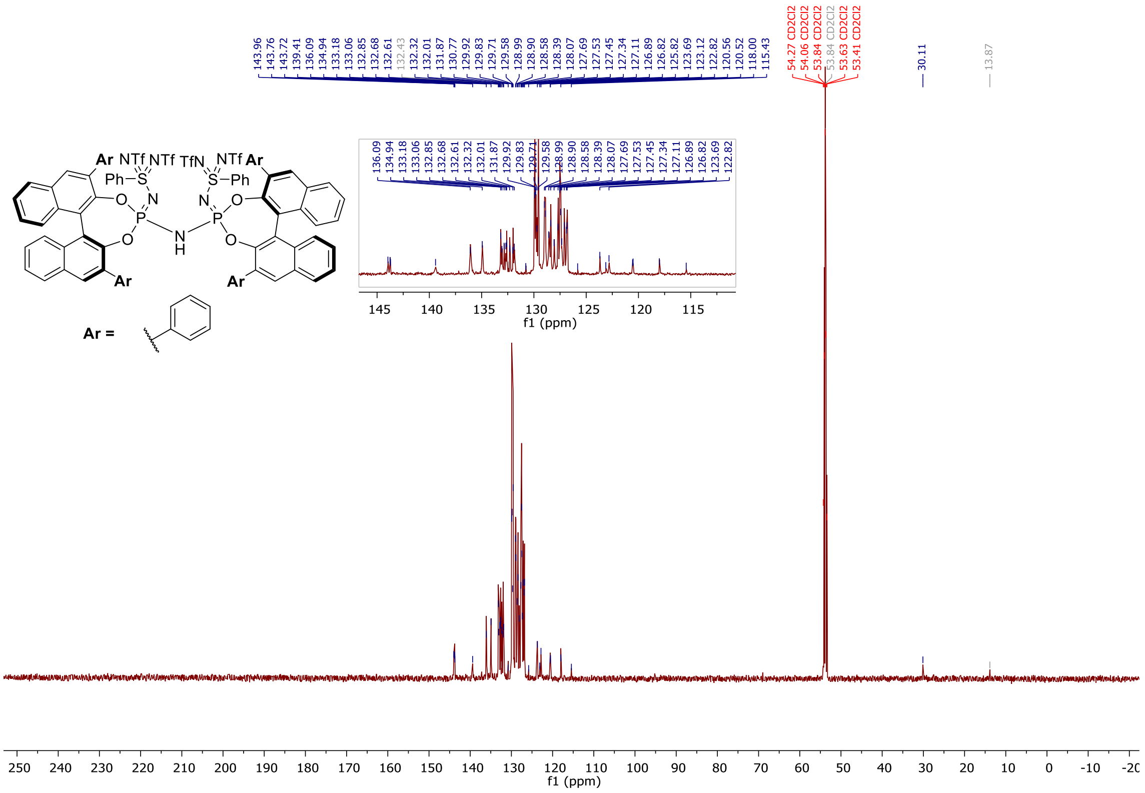

^{19}F NMR (471 MHz, CD_2Cl_2)

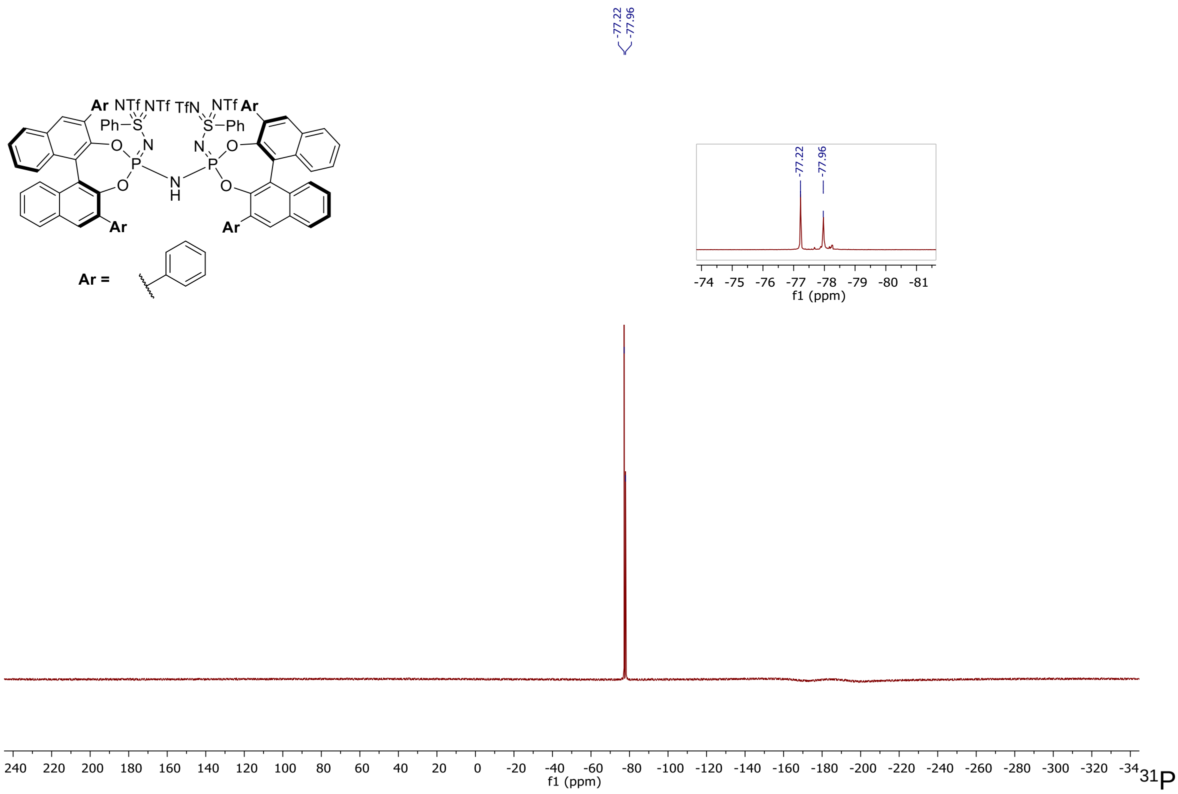

^{31}P NMR (203 MHz, CD_2Cl_2)

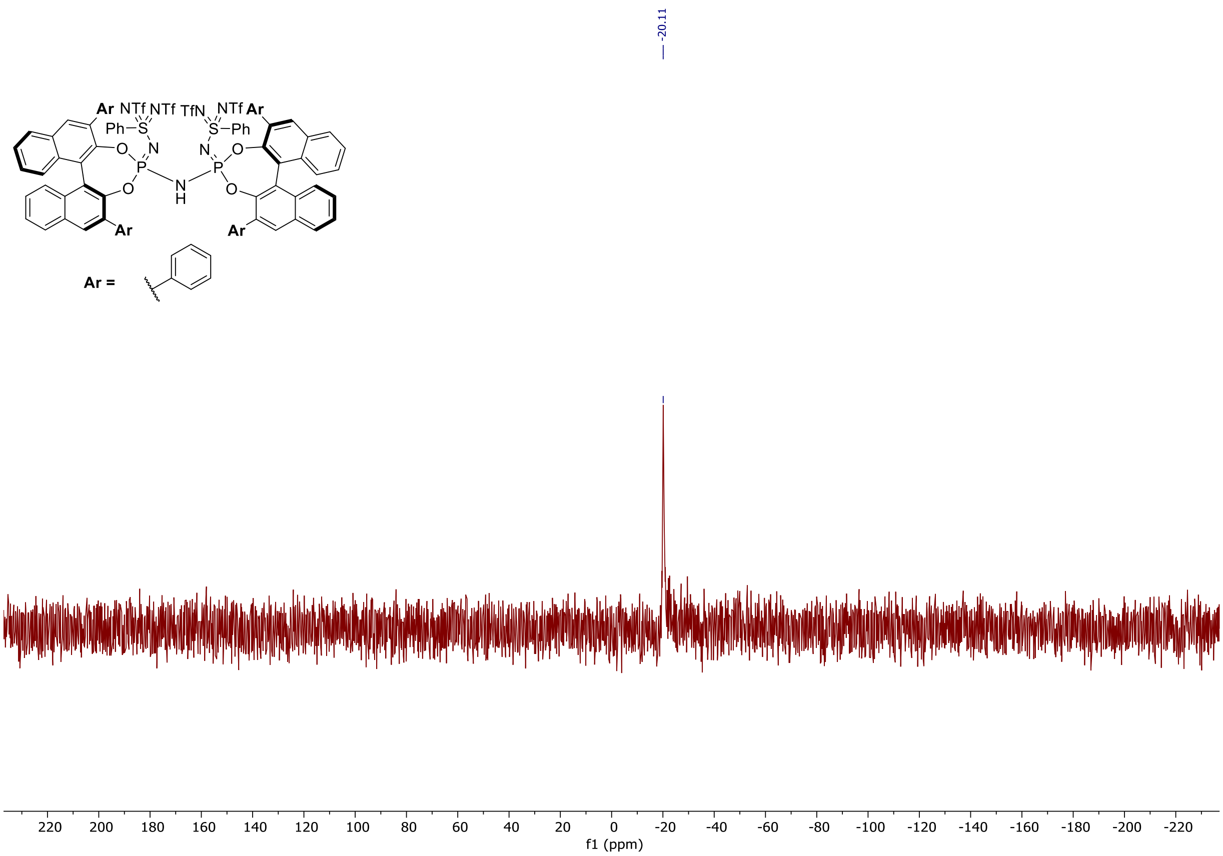

^{1}H NMR (600 MHz, CD_2Cl_2)

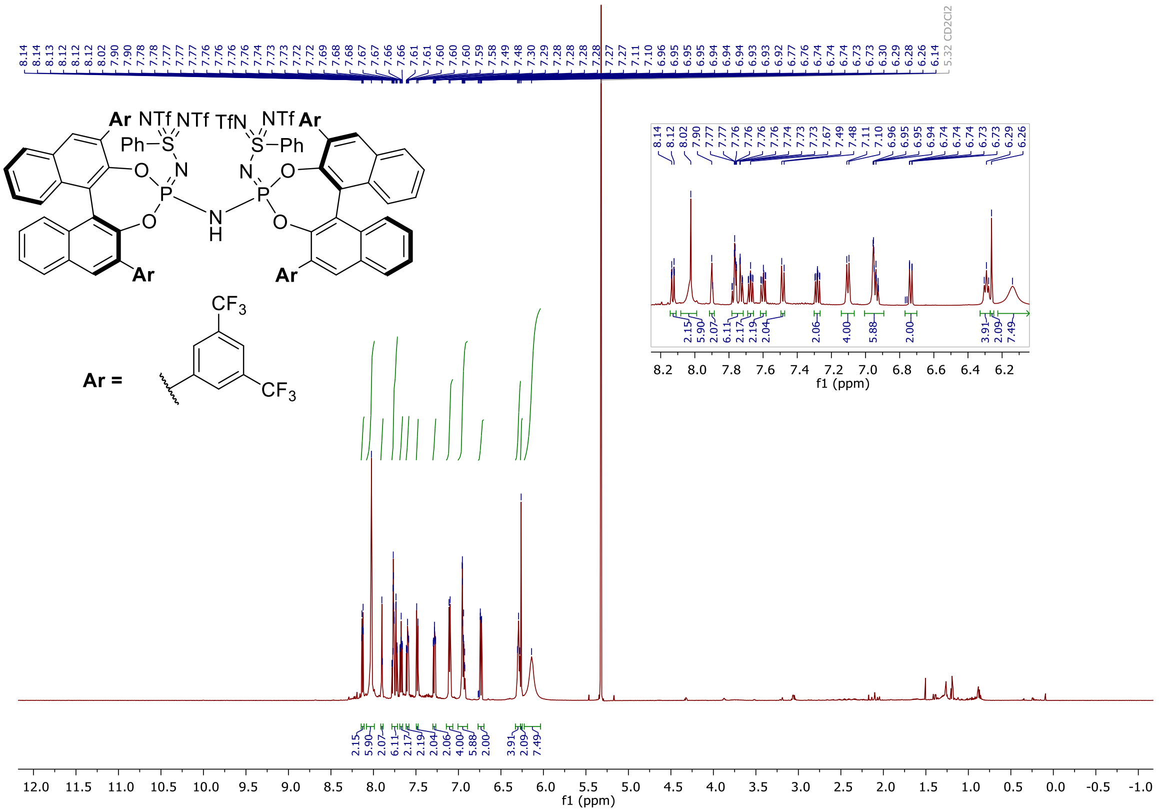

^{13}C NMR (151 MHz, CD_2Cl_2):

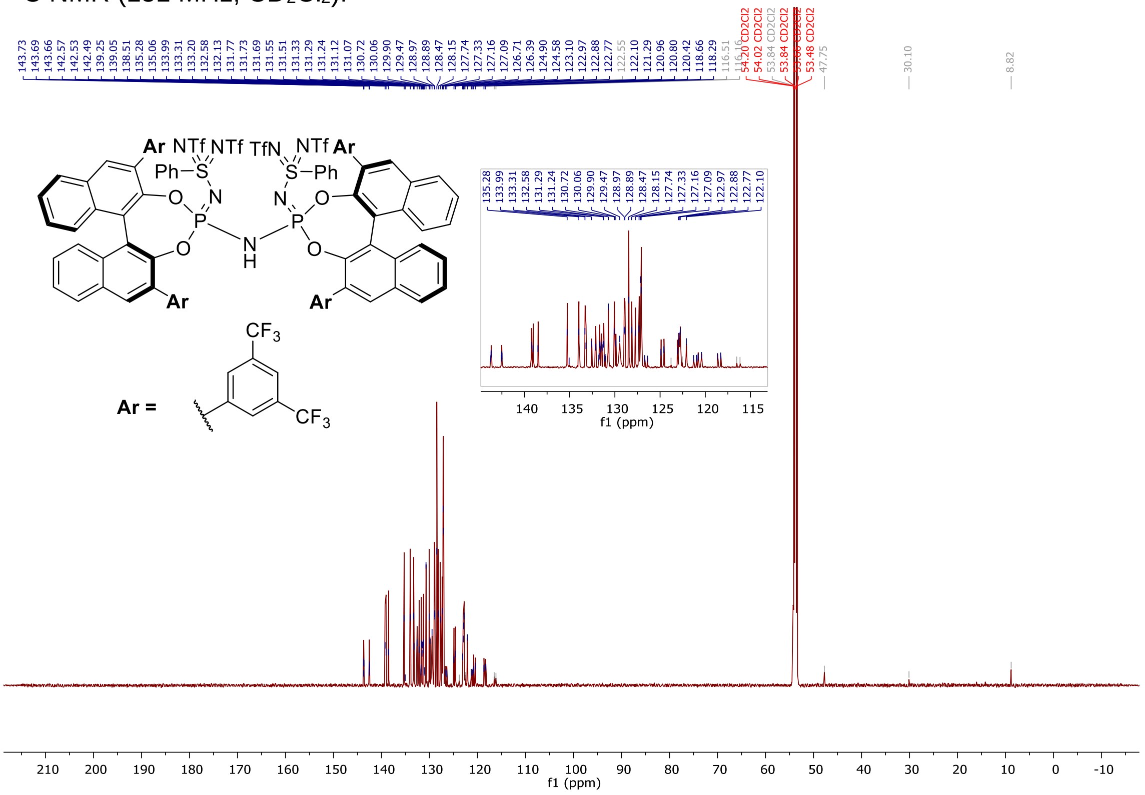

^{19}F NMR (471 MHz, CD_2Cl_2)

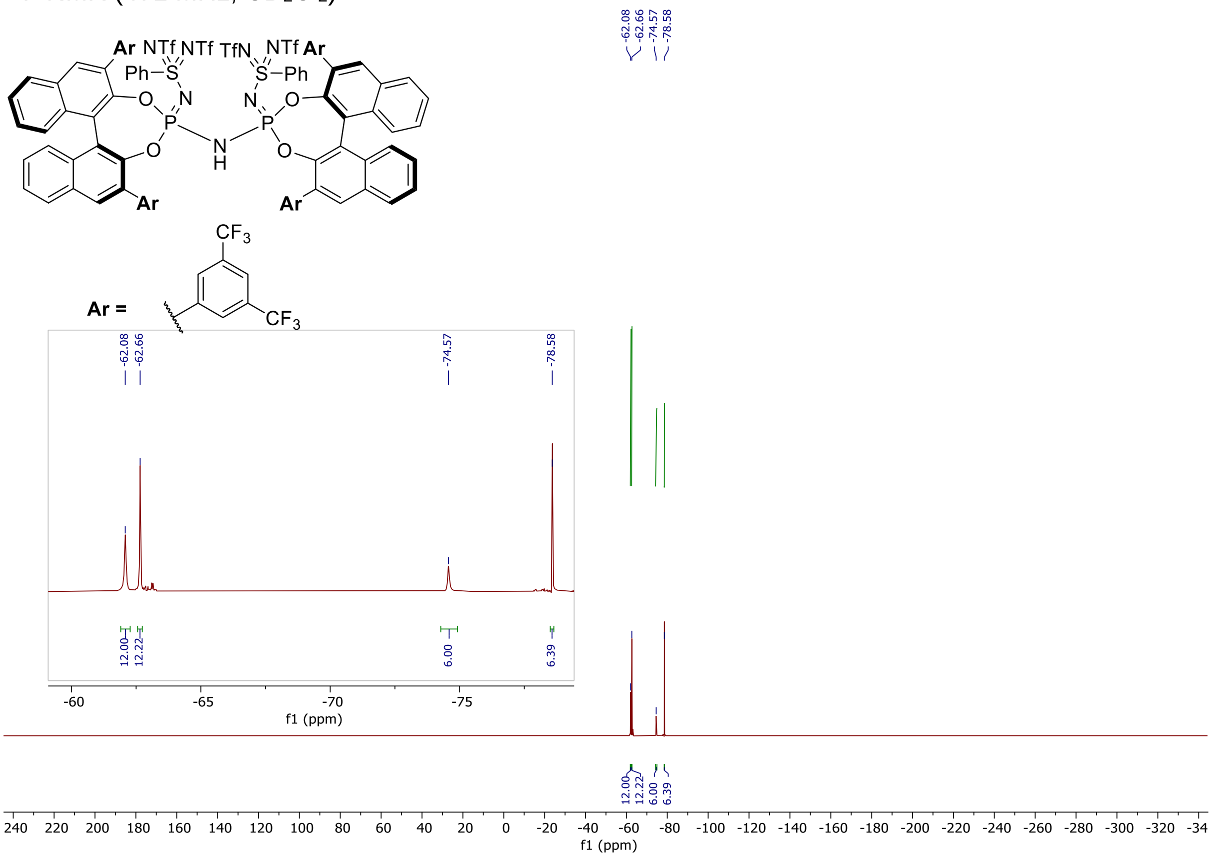

^{31}P NMR (203 MHz, CD_2Cl_2)

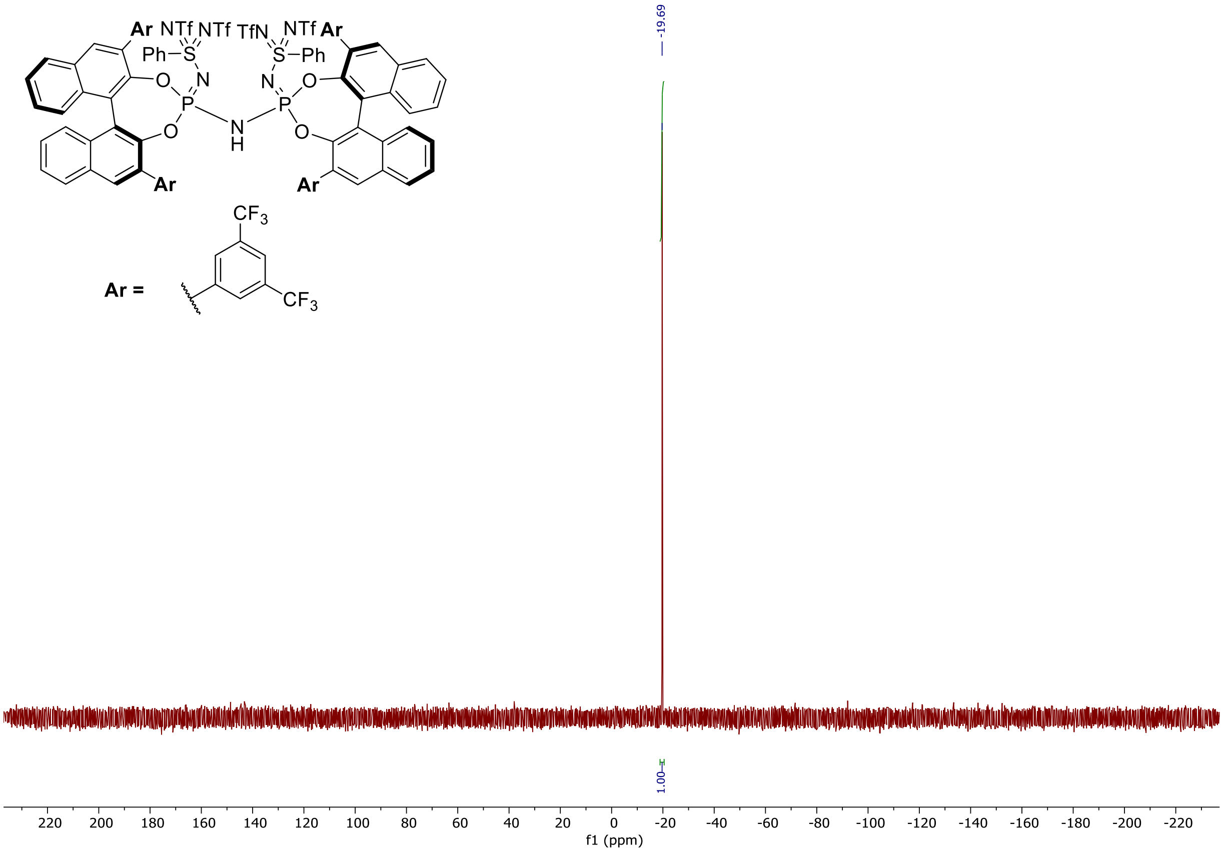

10.1 ^{29}Si-NMR studies

NMR tubes were charged with the corresponding catalyst, IDPi, IDPii, TfOH, Tf_2NH (0.01 mmol each), evacuated and purged with argon. Meanwhile a stock solution of allyltrimethylsilane (137 mg, 1.2 mmol) in anhydrous CD_2Cl_2 (3 ml) was prepared. 500 µl of this stock solution was added to each (four different with the corresponding catalyst) NMR tube, sealed and analyzed by NMR. ^{29}Si-NMR shifts were measured by ^{1}H-^{29}Si-HMBC

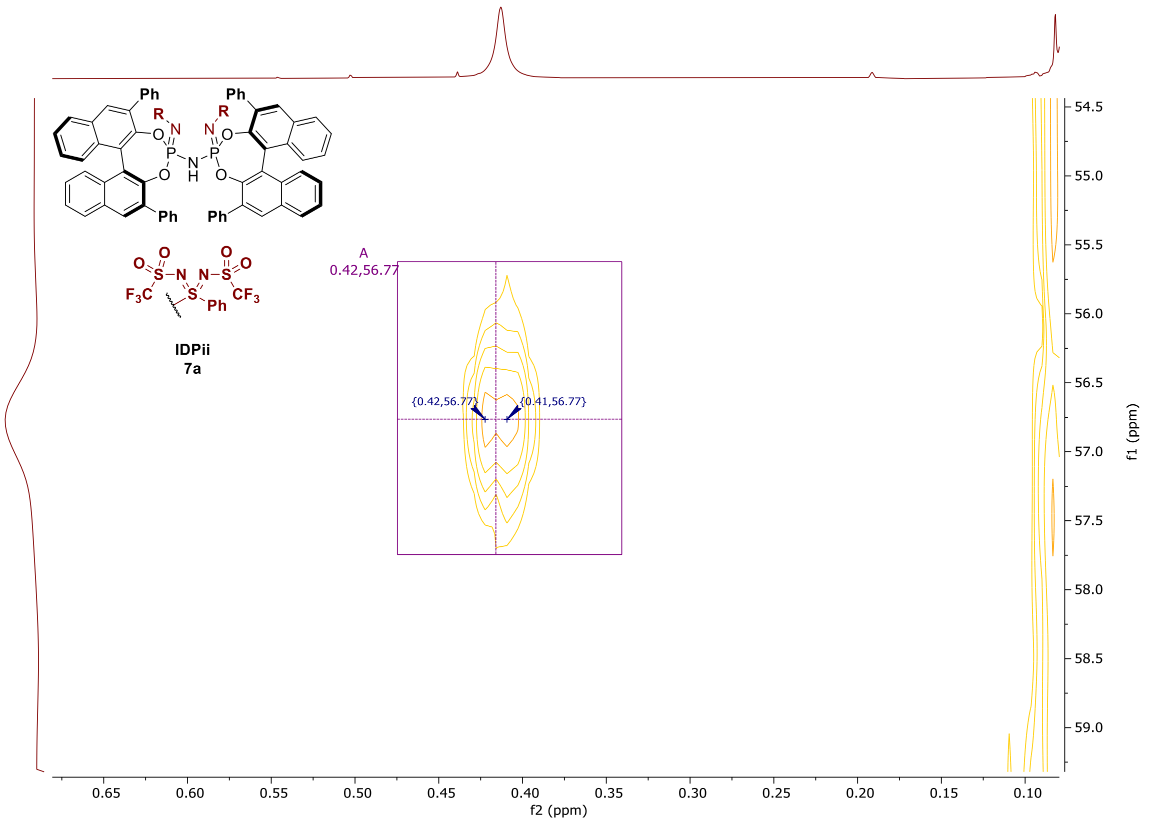

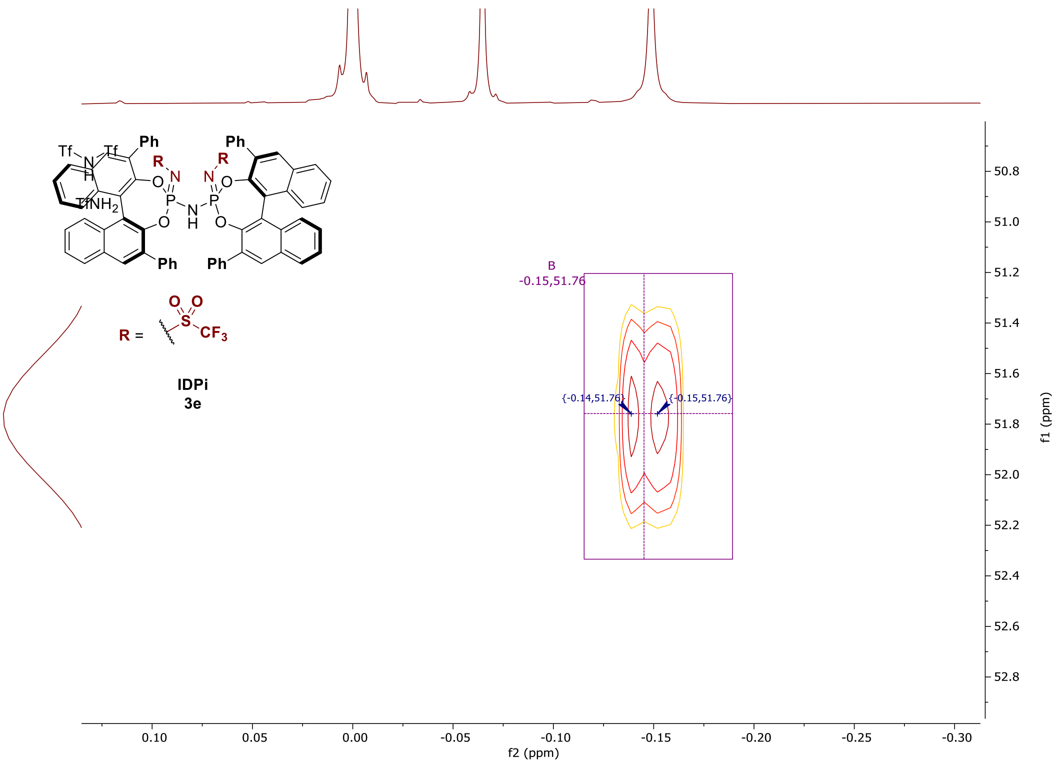

IDPi
3e
R =
B
-0.15,51.76
{-0.14,51.76}
{-0.15,51.76}
f2 (ppm)
f1 (ppm)

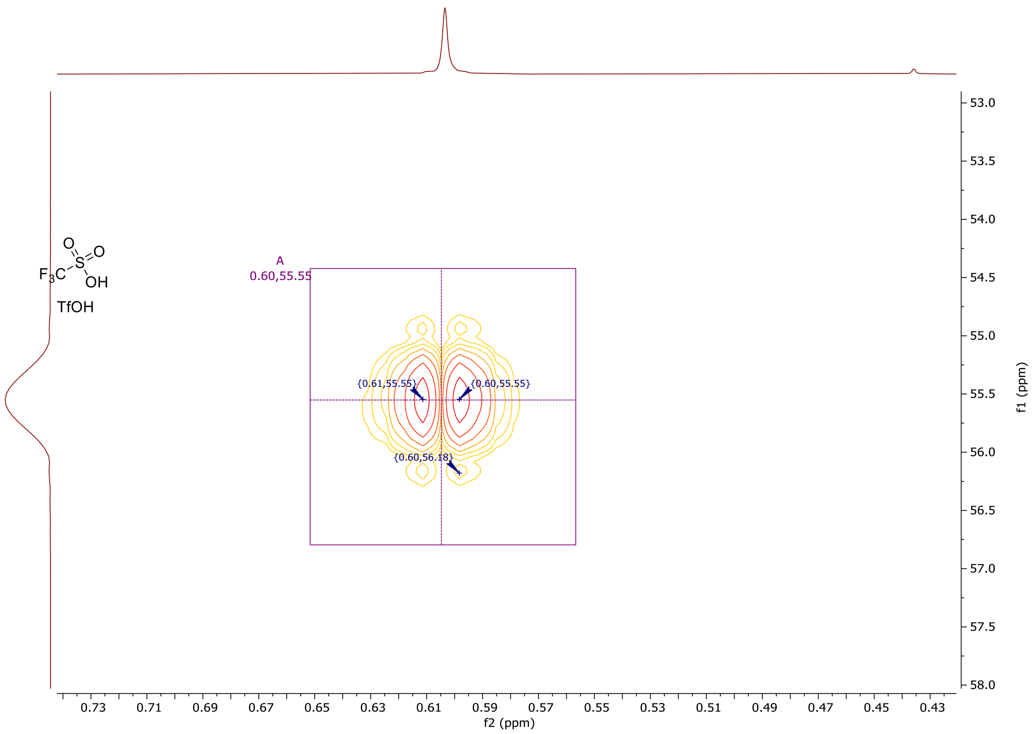

TfOH
A
0.60,55.55
{0.61,55.55}
{0.60,55.55}
{0.60,56.18}
f2 (ppm)
f1 (ppm)

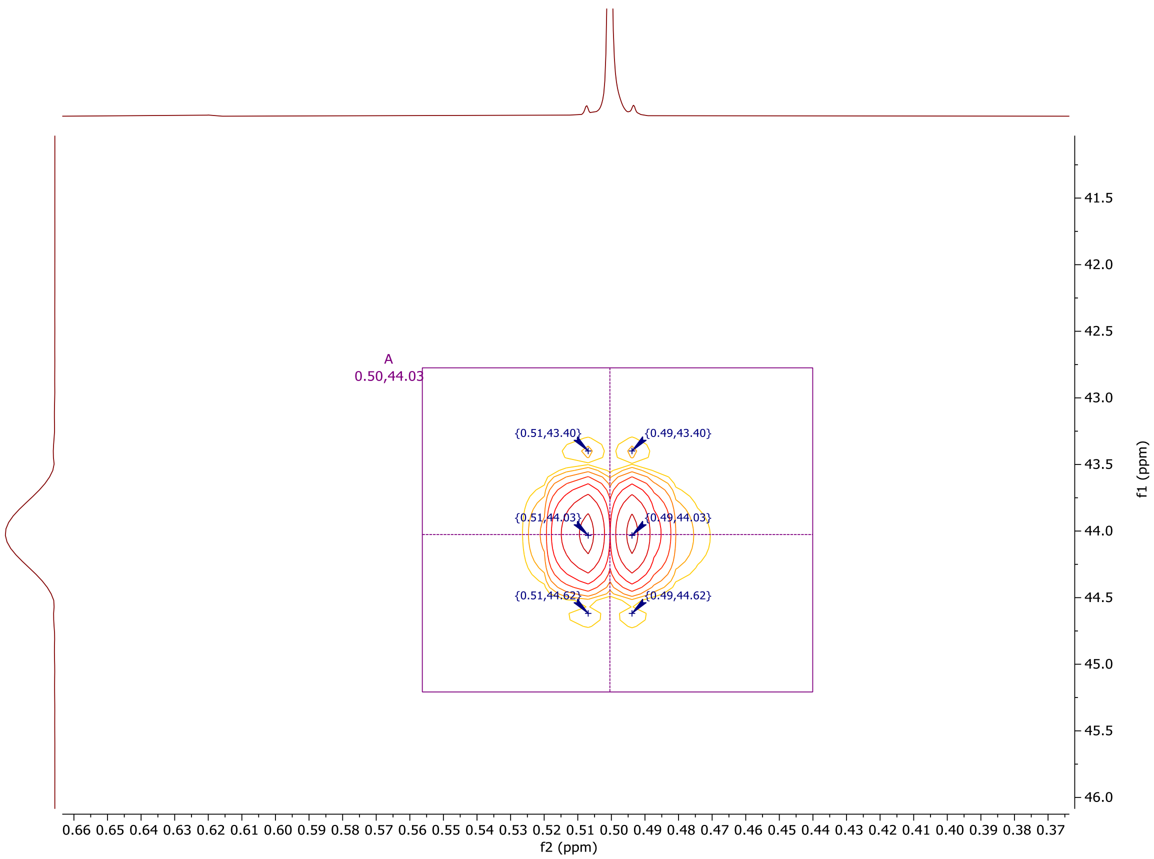

A
0.50,44.03
{0.51,43.40}
{0.49,43.40}
{0.51,44.03}
{0.49,44.03}
{0.51,44.62}
{0.49,44.62}
41.5
42.0
42.5
43.0
43.5
44.0
44.5
45.0
45.5
46.0
f1 (ppm)
0.66 0.65 0.64 0.63 0.62 0.61 0.60 0.59 0.58 0.57 0.56 0.55 0.54 0.53 0.52 0.51 0.50 0.49 0.48 0.47 0.46 0.45 0.44 0.43 0.42 0.41 0.40 0.39 0.38 0.37
f2 (ppm)

10.2 Gutmann-Beckett Studies

Two NMR tubes were charged with IDPii-7a (17.8 mg, 10 µmol) or IDPi-3e (12.7 mg, 10 µmol), dissolved in 200 µl anhydrous CD_2Cl_2 followed by subsequent addition of allyltrimethylsilane (8 µl, 50 µmol). A stock solution containing triethylphosphinoxide $Et_3P=O$ in anhydrous CD_2Cl_2 was prepared (3.8 mg, in 900 µl CD_2Cl_2). Of this stock solution, 300 µl were transferred to each NMR-tube containing the catalyst, respectively (1.27 mg, $Et_3P=O$, 9.5 µmol were transferred to each NMR-sample). Meanwhile, $Et_3P=O$ was measured by NMR. The NMR samples containing the Lewis acid and Et_3PO were NMR-spectroscopically analyzed after 30 minutes.

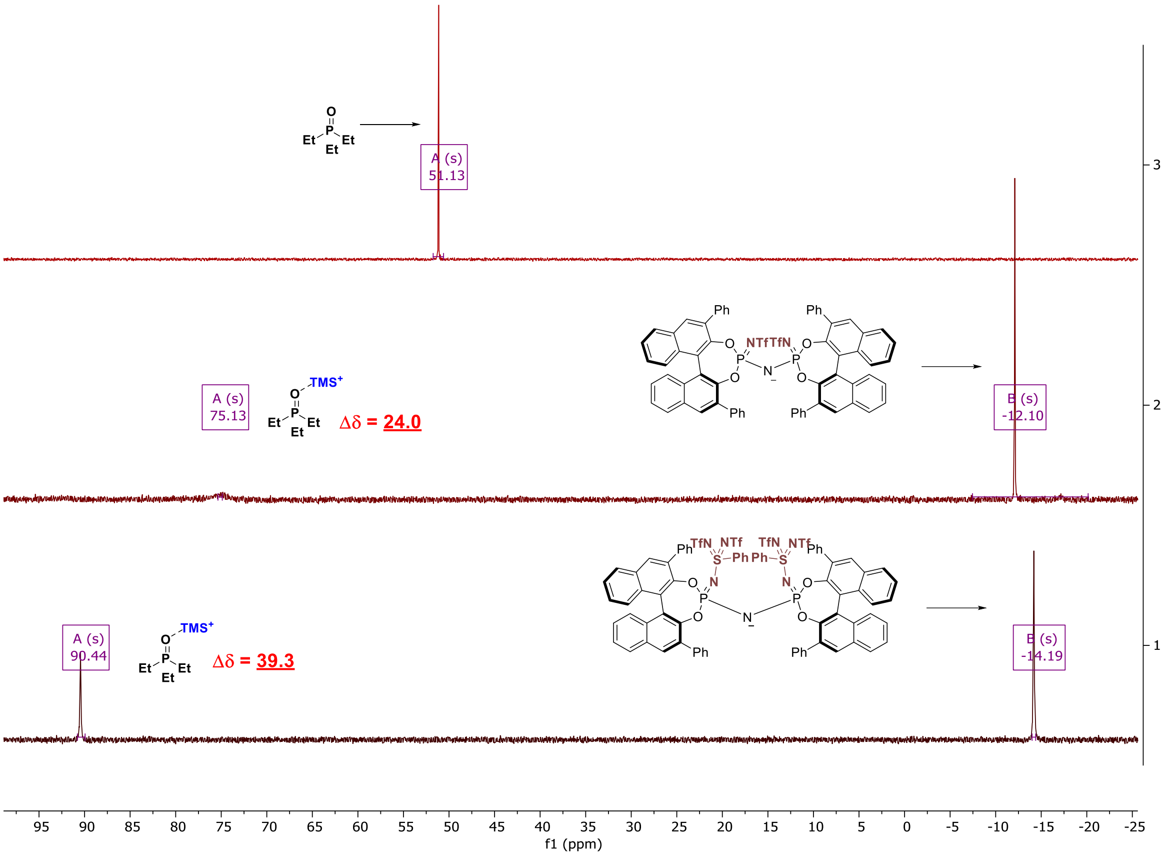

11 GC-traces

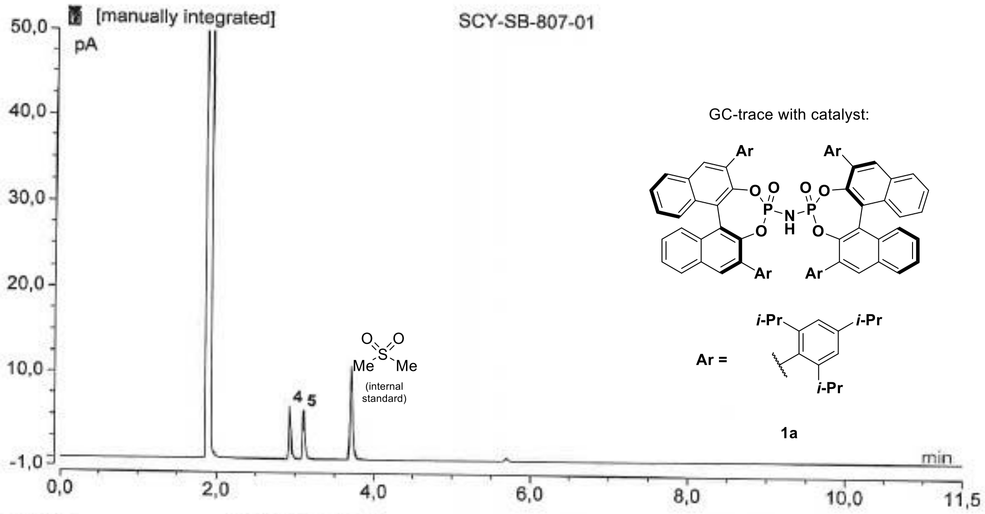

Sample:	**SCY-SB-807-01**	Instrument:	**GC_213**
Sequenz:	**7679 SCY-SB VD**	Measured:	**30.09.20 11:39**
Sequenz date:	**30.09.20**	Processing M.:	ee SCY-SB
		Report-File:	ee SCY-SB

chirale Messung, Verhältnis der Enantiomere
Zuordnung siehe Racemat WII-WD-432-01 19/5879

No.	Ret.Time min	Rel.Area %	Peak Name
4	2,93	50,32	.
5	3,11	49,68	.

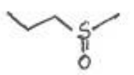

Instrument parameters:

Column:	30,0 m	G-TA 0,25/?df ; G/448
Temperature:	220/ 160, 6 min iso	8/min 180, 3 min iso / 350
Gas:	0,60 bar	H2
Sample size:	0,2 µL	

V. Diehl

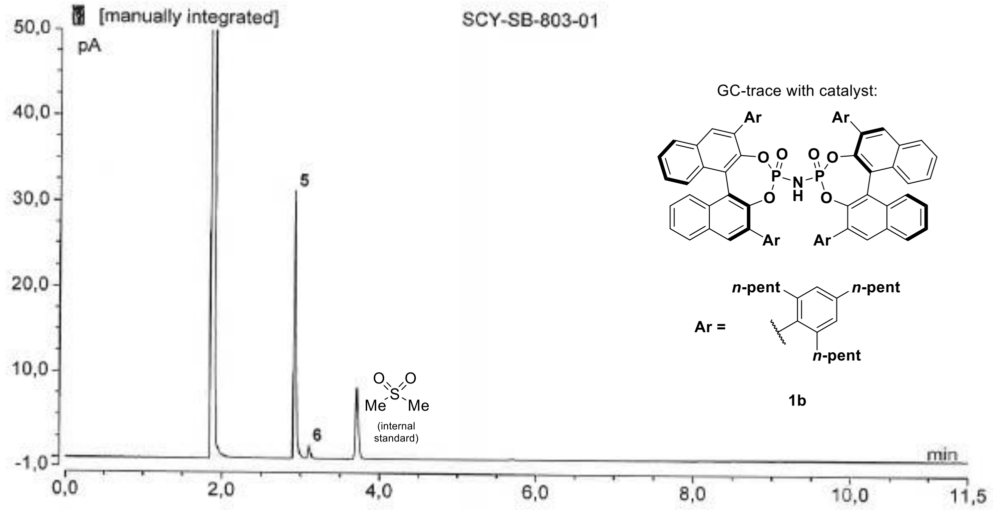

Sample:	SCY-SB-803-01	Instrument:	GC_213
Sequenz:	7679 SCY-SB VD	Measured:	30.09.20 09:56
Sequenz date:	30.09.20	Processing M.:	ee SCY-SB
		Report-File:	ee SCY-SB

chirale Messung, Verhältnis der Enantiomere
Zuordnung siehe Racemat WII-WD-432-01 19/5879

No.	Ret.Time min	Rel.Area %	Peak Name
5	2,92	95,08	.
6	3,11	4,92	.

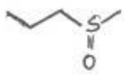

Instrument parameters:

Column:	30,0 m	G-TA 0,25/?df ; G/448
Temperature:	220/ 160, 6 min iso	8/min 180, 3 min iso / 350
Gas:	0,60 bar	H2
Sample size:	0,2 µL	

V. Diehl

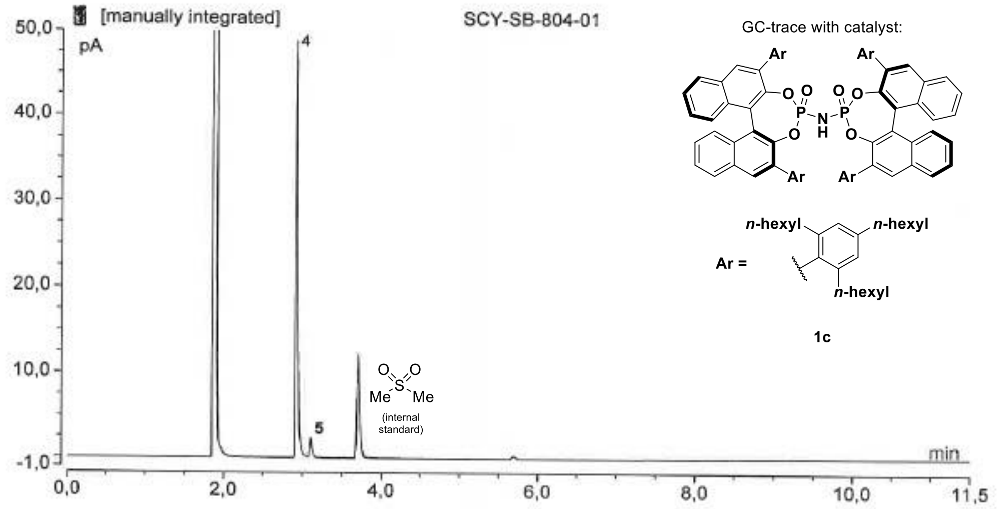

1c

Sample:	**SCY-SB-804-01**	Instrument:	**GC_213**
Sequenz:	**7679 SCY-SB VD**	Measured:	**30.09.20 10:16**
Sequenz date:	**30.09.20**	Processing M.:	ee SCY-SB
		Report-File:	ee SCY-SB

chirale Messung, Verhältnis der Enantiomere
Zuordnung siehe Racemat WII-WD-432-01 19/5879

No.	Ret.Time min	Rel.Area %	Peak Name
4	2,92	95,18	.
5	3,11	4,82	.

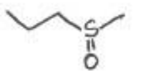

Instrument parameters:

Column:	30,0 m	G-TA 0,25/?df ; G/448
Temperature:	220/ 160, 6 min iso	8/min 180, 3 min iso / 350
Gas:	0,60 bar	H2
Sample size:	0,2 µL	

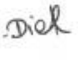

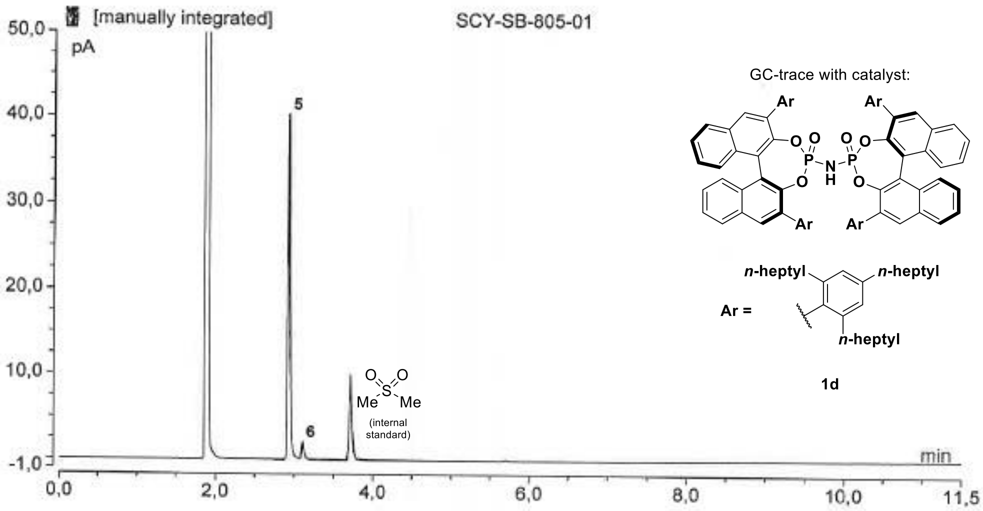

Sample:	SCY-SB-805-01	Instrument: GC_213
Sequenz:	7679 SCY-SB VD	Measured: 30.09.20 11:01
Sequenz date:	30.09.20	Processing M.: ee SCY-SB
		Report-File: ee SCY-SB

chirale Messung, Verhältnis der Enantiomere
Zuordnung siehe Racemat WII-WD-432-01 19/5879

No.	Ret.Time min	Rel.Area %	Peak Name
5	2,92	94,81	.
6	3,11	5,19	.

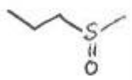

Instrument parameters:

Column:	30,0 m	G-TA 0,25/?df ; G/448
Temperature:	220/ 160, 6 min iso	8/min 180, 3 min iso / 350
Gas:	0,60 bar	H2
Sample size:	0,2 µL	

V. Diel

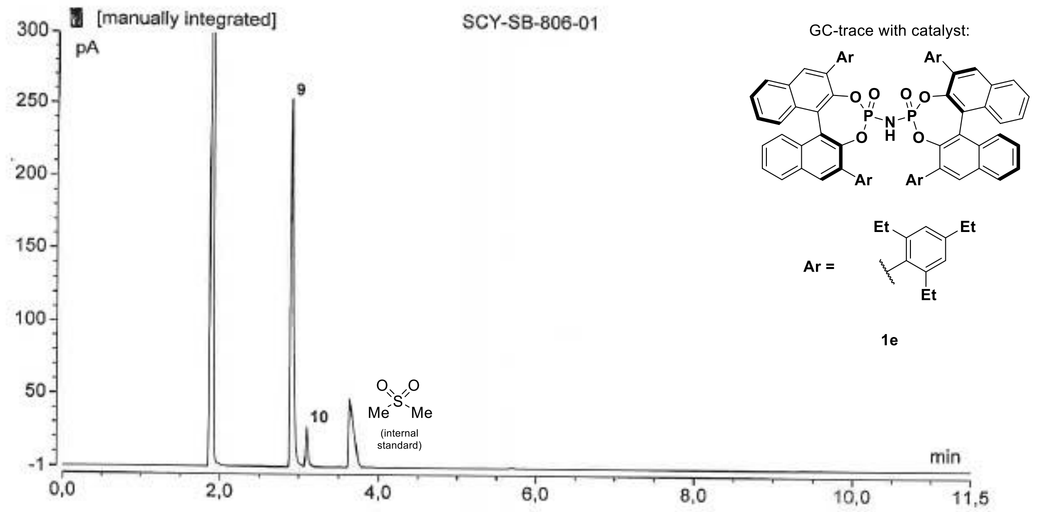

Sample:	**SCY-SB-806-01**	Instrument:	**GC_213**
Sequenz:	**7679 SCY-SB VD**	Measured:	**30.09.20 11:19**
Sequenz date:	**30.09.20**	Processing M.:	ee SCY-SB
		Report-File:	ee SCY-SB-806-01

chirale Messung, Verhältnis der Enantiomere
Zuordnung siehe Racemat WII-WD-432-01 19/5879

No.	Ret.Time min	Rel.Area %	Peak Name
9	2,89	91,48	.
10	3,09	8,52	.

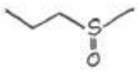

Instrument parameters:

Column:	30,0 m	G-TA 0,25/?df ; G/448
Temperature:	220/ 160, 6 min iso 8/min 180, 3 min iso / 350	
Gas:	0,60 bar	H2
Sample size:	0,2 µL	

V. Diehl

12.1 Single crystal structure analysis of [2d$^-$ H_3O^+], 1·H_2O 2· CH_2Cl_2

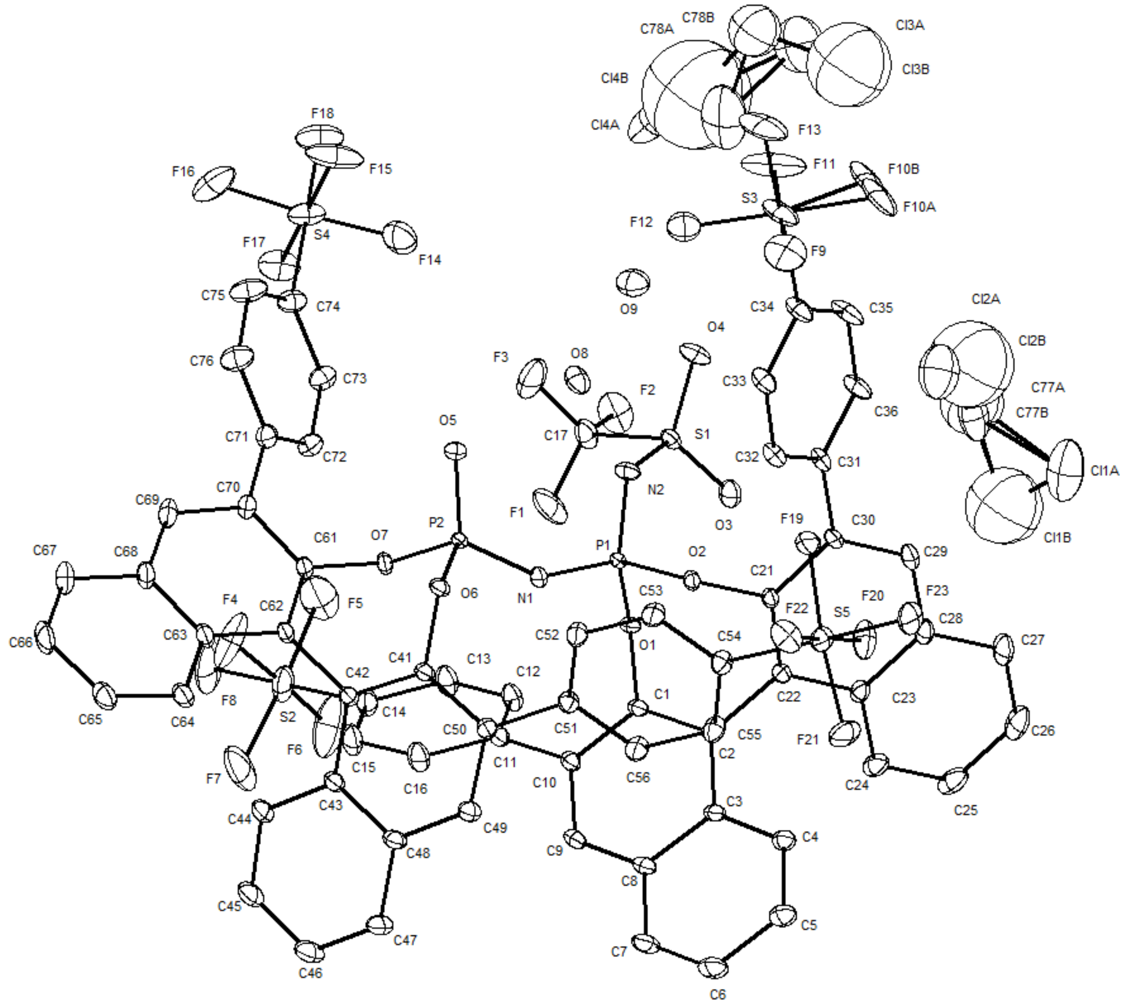

Figure S1. The molecular structure of complex **[2d$^-$ H_3O^+], 1·H_2O 2· CH_2Cl_2**. H atoms have been removed for clarity.

X-ray Crystal Structure Analysis of complex [2d$^-$ H_3O^+], 1·H_2O 2· CH_2Cl_2: $C_{67}H_{45}Cl_4F_{23}N_2O_9P_2S_5$, M_r = 1823.09 g mol^{-1}, colourless block, crystal size 0.18 x 0.17 x 0.16 mm^3, orthorhombic, $P2_12_12_1$ [19], a = 15.828(3) Å, b = 18.4770(16) Å, c = 25.099(3) Å, V = 7340.3(18) Å^3, T = 100(2) K, Z = 4, D_{calc} = 1.650 g·cm^3, λ = 0.6199 Å, μ(0.6199 Å) = 0.315 mm^{-1}, no absorption correction, P11 beamline @PETRA III with Pilatus 6M detector and (l)N_2 cooled double crystal monochromator, 1.194 < θ < 27.011°, 151485 measured reflections, 23763 independent reflections, 23338 reflections with $I > 2\sigma(I)$, R_{int} = 0.0470. The structure was solved by *SHELXT* and refined by full-matrix least-squares (*SHELXL*) against F^2 to R_1 = 0.0322 [$I > 2\sigma(I)$], wR_2 = 0.0875, 1059 parameters, absolute structure parameter Flack (x) = 0.030(11)

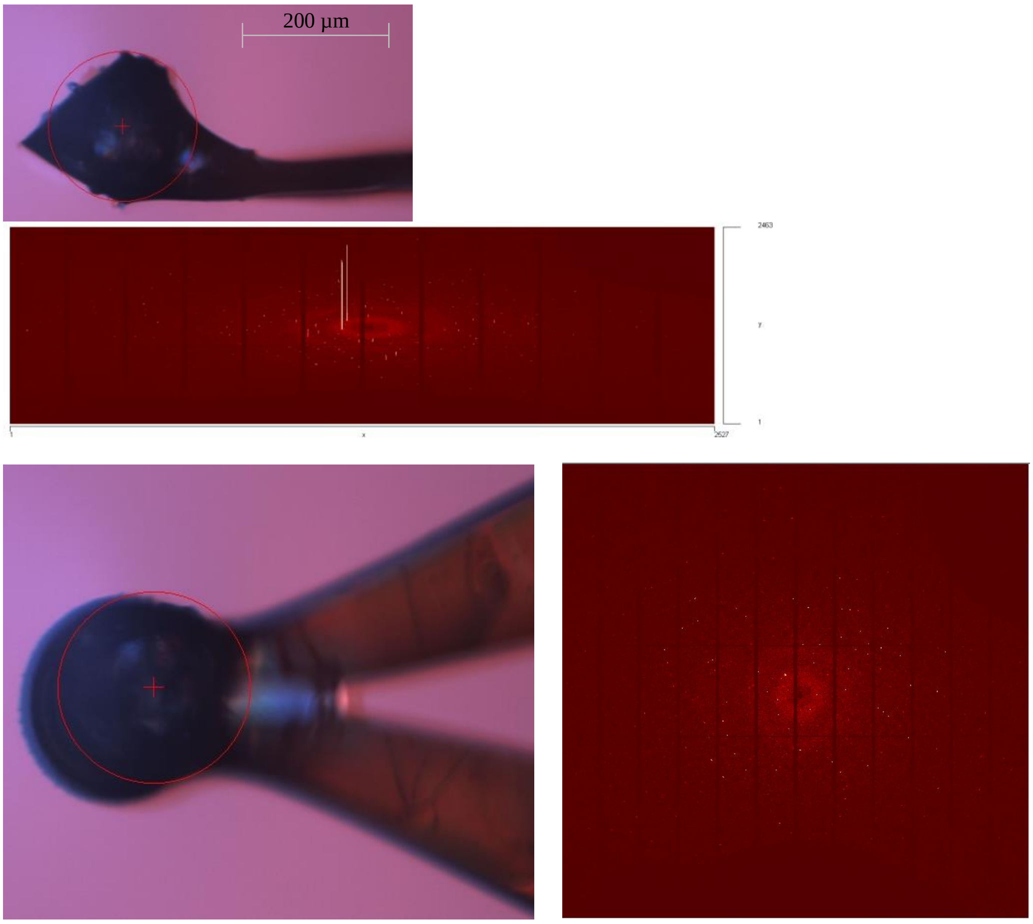

Figure S2. Crystal mounted on a 150µm MiTiGen-Loop with beam size diameter (red ring) and selected reflection frame as heat map and in 3D view with pixel coordinates.

INTENSITY STATISTICS FOR DATASET

Resolution	#Data	#Theory	%Complete	Redundancy	Mean I	Mean I/s	Rmerge	Rsigma
Inf - 2.79	360	365	98.6	7.28	73.11	43.35	0.0400	0.0215
2.79 - 1.86	842	846	99.5	6.78	28.58	41.22	0.0409	0.0233
1.86 - 1.47	1236	1236	100.0	7.27	18.11	40.48	0.0416	0.0227
1.47 - 1.29	1163	1163	100.0	6.69	14.34	38.02	0.0421	0.0243
1.29 - 1.17	1219	1219	100.0	6.76	12.64	37.07	0.0427	0.0244
1.17 - 1.08	1311	1311	100.0	7.02	10.69	36.76	0.0447	0.0244
1.08 - 1.02	1134	1136	99.8	7.04	7.05	33.66	0.0476	0.0256
1.02 - 0.97	1176	1176	100.0	6.59	5.18	30.50	0.0492	0.0277
0.97 - 0.93	1116	1117	99.9	6.39	4.18	27.95	0.0528	0.0298
0.93 - 0.89	1368	1372	99.7	6.70	4.05	27.79	0.0526	0.0296
0.89 - 0.86	1170	1172	99.8	6.74	3.34	26.17	0.0570	0.0312
0.86 - 0.83	1367	1369	99.9	6.61	3.10	24.83	0.0593	0.0326
0.83 - 0.81	1010	1016	99.4	6.28	2.87	23.88	0.0624	0.0348
0.81 - 0.79	1122	1129	99.4	5.96	2.97	22.81	0.0621	0.0357
0.79 - 0.77	1236	1260	98.1	6.06	2.60	21.73	0.0641	0.0368
0.77 - 0.75	1363	1387	98.3	6.14	2.78	21.81	0.0649	0.0364
0.75 - 0.73	1468	1533	95.8	5.99	2.36	20.57	0.0727	0.0392
0.73 - 0.72	770	821	93.8	5.20	2.25	18.81	0.0785	0.0438
0.72 - 0.70	1718	1840	93.4	4.82	2.24	17.59	0.0758	0.0460
0.70 - 0.69	931	978	95.2	4.88	1.98	16.48	0.0801	0.0490
0.69 - 0.68	732	791	92.5	4.68	1.85	15.66	0.0838	0.0521
0.78 - 0.68	7625	8006	95.2	5.40	2.32	19.05	0.0724	0.0423
Inf - 0.68	23812	24237	98.2	6.26	7.42	27.45	0.0469	0.0267

Complete .cif-data of the compound are avaliable under the CCDC number **CCDC-2043273**.

Five low indexed reflections [0 1 2], [0 2 0], [1 0 2], [2 0 1], [2 0 0] were shadowed by the beamstop and removed from the dataset before the final refinement cycles. The structure contains two-disordered solute molecules (CH_2Cl_2) with occupancy of 72:28 and 82:18 over two positions. One terminal SF_5 group in meta position appears to be disordered over two positions with occupancy of 68:32, thermal eliposids were equalized using ISOR 0.005 0.01 F10B F10A and EADP F10B F10A restraints. The H atoms on the solvent water molecule and hydroxonium ion were located on a difference Fourier map and their coordinates were restrained using a SADI O8 H5A O8 H8A O8 H8B O9 H9A O9 H9B instruction. There was no evidence in a difference Fourier map that either N1 or N2 carries a hydrogen atom.

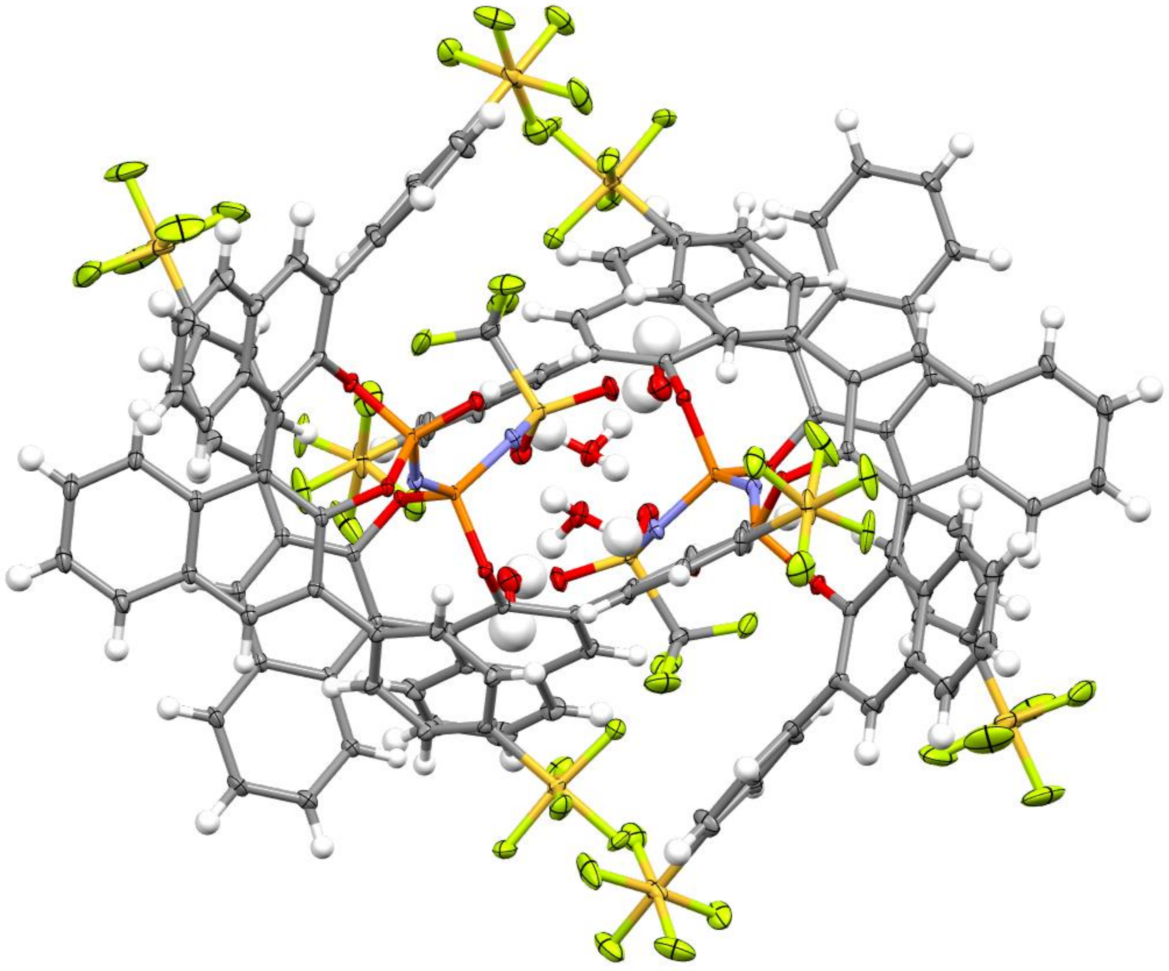

Figure S3. The anions of 2d viewed along *a* axis of the unit cell showing the encapsulated water molecules and hydroxonium cations. Disorder solvent molecules have been removed for clarity.

Table 1. Crystal data and structure refinement.

Identification code	12605

Empirical formula	$C_{67} H_{45} Cl_4 F_{23} N_2 O_9 P_2 S_5$
Color	colourless
Formula weight	1823.09 g·mol^{-1}
Temperature	100(2) K
Wavelength	0.619900 Å
Crystal system	Orthorhombic
Space group	$P2_12_12_1$, (No. 19)
Unit cell dimensions	a = 15.828(3) Å α= 90°.
	b = 18.4770(16) Å β= 90°.
	c = 25.099(3) Å γ = 90°.
Volume	7340.3(18) Å^3
Z	4
Density (calculated)	1.650 Mg·m^{-3}
Absorption coefficient	0.315 mm^{-1}
F(000)	3672 e
Crystal size	0.18 x 0.17 x 0.16 mm^3
θ range for data collection	1.194 to 27.011°.
Index ranges	$-23 \le h \le 23$, $-27 \le k \le 27$, $-36 \le l \le 36$
Reflections collected	151485
Independent reflections	23763 [R_{int} = 0.0470]
Reflections with I>2σ(I)	23338
Completeness to θ = 21.835°	99.8 %
Absorption correction	None
Max. and min. transmission	1.0000 and 0.9667
Refinement method	Full-matrix least-squares on F^2
Data / restraints / parameters	23763 / 22 / 1059
Goodness-of-fit on F^2	1.033
Final R indices [I>2σ(I)]	R_1 = 0.0322 wR^2 = 0.0875
R indices (all data)	R_1 = 0.0328 wR^2 = 0.0880
Absolute structure parameter	0.030(11)
Extinction coefficient	n/a
Largest diff. peak and hole	0.819 and -0.654 e·Å^{-3}

Table 2. Bond lengths [Å] and angles [°].

_

C(1)-C(2)	1.377(2)	C(1)-C(10)
1.422(2)	C(1)-O(1)	1.404(2)
C(2)-C(3)	1.430(2)	C(2)-C(22)
1.490(2)	C(3)-C(4)	1.419(2)
C(3)-C(8)	1.424(2)	C(4)-C(5)
1.372(3)	C(5)-C(6)	1.415(3)
C(6)-C(7)	1.370(3)	C(7)-C(8)
1.421(3)	C(8)-C(9)	1.414(3)
C(9)-C(10)	1.375(2)	C(10)-C(11)
1.482(3)	C(11)-C(12)	1.391(3)
C(11)-C(16)	1.396(2)	C(12)-C(13)
1.389(3)	C(13)-C(14)	1.391(3)
C(14)-C(15)	1.393(3)	C(14)-S(2)
1.796(2)	C(15)-C(16)	1.389(3)
C(17)-F(1)	1.324(3)	C(17)-F(2)
1.332(3)	C(17)-F(3)	1.320(3)
C(17)-S(1)	1.836(2)	C(21)-C(22)
1.375(2)	C(21)-C(30)	1.423(2)
C(21)-O(2)	1.403(2)	C(22)-C(23)
1.431(2)	C(23)-C(24)	1.414(3)
C(23)-C(28)	1.423(3)	C(24)-C(25)
1.377(3)	C(25)-C(26)	1.410(4)
C(26)-C(27)	1.371(3)	C(27)-C(28)
1.426(3)	C(28)-C(29)	1.413(3)
C(29)-C(30)	1.381(2)	C(30)-C(31)
1.484(2)	C(31)-C(32)	1.400(2)
C(31)-C(36)	1.402(2)	C(32)-C(33)
1.387(3)	C(33)-C(34)	1.387(3)
C(34)-C(35)	1.396(3)	C(34)-S(3)
1.799(2)	C(35)-C(36)	1.387(3)
C(41)-C(42)	1.381(2)	C(41)-C(50)
1.418(2)	C(41)-O(6)	1.403(2)
C(42)-C(43)	1.429(2)	C(42)-C(62)
1.482(3)	C(43)-C(44)	1.418(2)
C(43)-C(48)	1.422(3)	C(44)-C(45)
1.373(3)	C(45)-C(46)	1.408(3)

C(46)-C(47)	1.374(3)	C(47)-C(48)
1.421(3)	C(48)-C(49)	1.415(3)
C(49)-C(50)	1.381(2)	C(50)-C(51)
1.487(2)	C(51)-C(52)	1.401(2)
C(51)-C(56)	1.395(2)	C(52)-C(53)
1.392(3)	C(53)-C(54)	1.390(3)
C(54)-C(55)	1.387(3)	C(54)-S(5)
1.7987(18)	C(55)-C(56)	1.386(3)
C(61)-C(62)	1.378(3)	C(61)-C(70)
1.421(3)	C(61)-O(7)	1.402(2)
C(62)-C(63)	1.438(2)	C(63)-C(64)
1.419(3)	C(63)-C(68)	1.420(3)
C(64)-C(65)	1.376(3)	C(65)-C(66)
1.414(3)	C(66)-C(67)	1.372(3)
C(67)-C(68)	1.421(3)	C(68)-C(69)
1.408(3)	C(69)-C(70)	1.385(3)
C(70)-C(71)	1.482(3)	C(71)-C(72)
1.402(3)	C(71)-C(76)	1.394(3)
C(72)-C(73)	1.388(3)	C(73)-C(74)
1.390(3)	C(74)-C(75)	1.390(3)
C(74)-S(4)	1.802(2)	C(75)-C(76)
1.385(3)	F(4)-S(2)	1.575(2)
F(5)-S(2)	1.573(2)	F(6)-S(2)
1.567(2)	F(7)-S(2)	1.577(2)
F(8)-S(2)	1.5856(17)	F(9)-S(3)
1.5841(19)	F(10B)-S(3)	1.514(11)
F(11)-S(3)	1.574(2)	F(12)-S(3)
1.5837(18)	F(13)-S(3)	1.5933(16)
F(14)-S(4)	1.5814(19)	F(15)-S(4)
1.5790(17)	F(16)-S(4)	1.5848(19)
F(17)-S(4)	1.5810(15)	F(18)-S(4)
1.5874(16)	F(19)-S(5)	1.5884(13)
F(20)-S(5)	1.5828(15)	F(21)-S(5)
1.5862(14)	F(22)-S(5)	1.5946(15)
F(23)-S(5)	1.5927(13)	N(1)-P(1)
1.5423(16)	N(1)-P(2)	1.5535(16)
N(2)-P(1)	1.5871(16)	N(2)-S(1)
1.5343(16)	O(1)-P(1)	1.5920(13)
O(2)-P(1)	1.5915(13)	O(3)-S(1)

1.4432(16)	O(4)-S(1)	1.4437(15)
O(5)-P(2)	1.4965(14)	O(6)-P(2)
1.5957(14)	O(7)-P(2)	1.5902(14)
S(3)-F(10A)	1.629(6)	C(77A)-Cl(1A)
1.748(6)	C(77A)-Cl(2A)	1.754(6)
C(77B)-Cl(1B)	1.81(7)	C(77B)-Cl(2B)
1.62(7)	C(78A)-Cl(3A)	1.708(8)
C(78A)-Cl(4A)	1.742(8)	C(78B)-Cl(3B)
1.63(3)	C(78B)-Cl(4B)	1.90(4)
C(2)-C(1)-C(10)	123.67(15)	C(2)-C(1)-O(1)
118.25(15)	O(1)-C(1)-C(10)	118.08(15)
C(1)-C(2)-C(3)	118.12(15)	C(1)-C(2)-C(22)
119.30(15)	C(3)-C(2)-C(22)	122.50(15)
C(4)-C(3)-C(2)	122.14(16)	C(4)-C(3)-C(8)
118.75(16)	C(8)-C(3)-C(2)	119.00(16)
C(5)-C(4)-C(3)	120.64(17)	C(4)-C(5)-C(6)
120.58(18)	C(7)-C(6)-C(5)	120.13(18)
C(6)-C(7)-C(8)	120.63(18)	C(7)-C(8)-C(3)
119.25(17)	C(9)-C(8)-C(3)	119.79(16)
C(9)-C(8)-C(7)	120.95(17)	C(10)-C(9)-C(8)
121.64(16)	C(1)-C(10)-C(11)	121.62(15)
C(9)-C(10)-C(1)	117.28(16)	C(9)-C(10)-C(11)
120.98(16)	C(12)-C(11)-C(10)	119.81(16)
C(12)-C(11)-C(16)	119.06(17)	C(16)-C(11)-C(10)
120.94(16)	C(13)-C(12)-C(11)	121.04(17)
C(12)-C(13)-C(14)	119.02(18)	C(13)-C(14)-C(15)
120.98(18)	C(13)-C(14)-S(2)	118.93(15)
C(15)-C(14)-S(2)	120.09(15)	C(16)-C(15)-C(14)
119.14(18)	C(15)-C(16)-C(11)	120.74(18)
F(1)-C(17)-F(2)	108.1(2)	F(1)-C(17)-S(1)
111.11(15)	F(2)-C(17)-S(1)	109.88(14)
F(3)-C(17)-F(1)	108.59(19)	F(3)-C(17)-F(2)
108.45(19)	F(3)-C(17)-S(1)	110.61(16)
C(22)-C(21)-C(30)	123.65(16)	C(22)-C(21)-O(2)
117.67(15)	O(2)-C(21)-C(30)	118.68(15)
C(21)-C(22)-C(2)	119.39(15)	C(21)-C(22)-C(23)
118.85(16)	C(23)-C(22)-C(2)	121.71(15)
C(24)-C(23)-C(22)	122.78(17)	C(24)-C(23)-C(28)

118.81(16)	C(28)-C(23)-C(22)	118.41(16)
C(25)-C(24)-C(23)	120.9(2)	C(24)-C(25)-C(26)
120.4(2)	C(27)-C(26)-C(25)	120.14(19)
C(26)-C(27)-C(28)	120.7(2)	C(23)-C(28)-C(27)
118.99(18)	C(29)-C(28)-C(23)	120.18(16)
C(29)-C(28)-C(27)	120.82(18)	C(30)-C(29)-C(28)
121.76(16)	C(21)-C(30)-C(31)	122.61(15)
C(29)-C(30)-C(21)	117.06(16)	C(29)-C(30)-C(31)
120.32(16)	C(32)-C(31)-C(30)	121.47(16)
C(32)-C(31)-C(36)	118.40(17)	C(36)-C(31)-C(30)
120.11(16)	C(33)-C(32)-C(31)	121.18(17)
C(32)-C(33)-C(34)	119.34(17)	C(33)-C(34)-C(35)
120.80(18)	C(33)-C(34)-S(3)	118.91(15)
C(35)-C(34)-S(3)	120.27(15)	C(36)-C(35)-C(34)
119.30(18)	C(35)-C(36)-C(31)	120.96(18)
C(42)-C(41)-C(50)	124.03(16)	C(42)-C(41)-O(6)
117.21(15)	O(6)-C(41)-C(50)	118.76(15)
C(41)-C(42)-C(43)	118.44(16)	C(41)-C(42)-C(62)
119.81(15)	C(43)-C(42)-C(62)	121.74(15)
C(44)-C(43)-C(42)	122.46(17)	C(44)-C(43)-C(48)
118.97(16)	C(48)-C(43)-C(42)	118.54(16)
C(45)-C(44)-C(43)	120.58(19)	C(44)-C(45)-C(46)
120.55(19)	C(47)-C(46)-C(45)	120.23(19)
C(46)-C(47)-C(48)	120.59(19)	C(47)-C(48)-C(43)
118.99(17)	C(49)-C(48)-C(43)	120.00(16)
C(49)-C(48)-C(47)	121.00(18)	C(50)-C(49)-C(48)
121.95(17)	C(41)-C(50)-C(51)	123.54(16)
C(49)-C(50)-C(41)	116.74(16)	C(49)-C(50)-C(51)
119.71(16)	C(52)-C(51)-C(50)	122.94(16)
C(56)-C(51)-C(50)	118.50(16)	C(56)-C(51)-C(52)
118.52(17)	C(53)-C(52)-C(51)	120.66(17)
C(54)-C(53)-C(52)	119.28(17)	C(53)-C(54)-S(5)
120.19(14)	C(55)-C(54)-C(53)	121.09(17)
C(55)-C(54)-S(5)	118.72(14)	C(56)-C(55)-C(54)
119.02(17)	C(55)-C(56)-C(51)	121.42(17)
C(62)-C(61)-C(70)	123.41(17)	C(62)-C(61)-O(7)
118.33(16)	O(7)-C(61)-C(70)	118.24(16)
C(61)-C(62)-C(42)	120.41(16)	C(61)-C(62)-C(63)
118.77(17)	C(63)-C(62)-C(42)	120.76(17)

C(64)-C(63)-C(62)	122.60(18)	C(64)-C(63)-C(68)
118.83(17)	C(68)-C(63)-C(62)	118.56(18)
C(65)-C(64)-C(63)	120.3(2)	C(64)-C(65)-C(66)
120.8(2)	C(67)-C(66)-C(65)	120.04(19)
C(66)-C(67)-C(68)	120.4(2)	C(63)-C(68)-C(67)
119.6(2)	C(69)-C(68)-C(63)	119.85(17)
C(69)-C(68)-C(67)	120.58(19)	C(70)-C(69)-C(68)
122.26(18)	C(61)-C(70)-C(71)	123.86(16)
C(69)-C(70)-C(61)	116.95(18)	C(69)-C(70)-C(71)
119.19(17)	C(72)-C(71)-C(70)	122.89(17)
C(76)-C(71)-C(70)	118.74(18)	C(76)-C(71)-C(72)
118.26(19)	C(73)-C(72)-C(71)	120.83(18)
C(72)-C(73)-C(74)	119.37(19)	C(73)-C(74)-C(75)
121.01(19)	C(73)-C(74)-S(4)	119.67(16)
C(75)-C(74)-S(4)	119.31(16)	C(76)-C(75)-C(74)
118.8(2)	C(75)-C(76)-C(71)	121.7(2)
P(1)-N(1)-P(2)	146.65(11)	S(1)-N(2)-P(1)
131.58(11)	C(1)-O(1)-P(1)	115.46(11)
C(21)-O(2)-P(1)	118.89(11)	C(41)-O(6)-P(2)
116.26(11)	C(61)-O(7)-P(2)	118.85(12)
N(1)-P(1)-N(2)	115.52(9)	N(1)-P(1)-O(1)
111.45(8)	N(1)-P(1)-O(2)	106.20(8)
N(2)-P(1)-O(1)	108.54(8)	N(2)-P(1)-O(2)
110.53(8)	O(2)-P(1)-O(1)	103.98(7)
N(1)-P(2)-O(6)	108.97(8)	N(1)-P(2)-O(7)
106.43(8)	O(5)-P(2)-N(1)	120.80(8)
O(5)-P(2)-O(6)	105.52(8)	O(5)-P(2)-O(7)
109.97(8)	O(7)-P(2)-O(6)	103.93(7)
N(2)-S(1)-C(17)	102.85(9)	O(3)-S(1)-C(17)
103.64(10)	O(3)-S(1)-N(2)	116.74(9)
O(3)-S(1)-O(4)	116.89(9)	O(4)-S(1)-C(17)
103.80(10)	O(4)-S(1)-N(2)	110.66(9)
F(4)-S(2)-C(14)	91.76(11)	F(4)-S(2)-F(7)
88.98(15)	F(4)-S(2)-F(8)	87.93(12)
F(5)-S(2)-C(14)	92.26(10)	F(5)-S(2)-F(4)
89.77(17)	F(5)-S(2)-F(7)	175.33(10)
F(5)-S(2)-F(8)	87.97(11)	F(6)-S(2)-C(14)
92.55(10)	F(6)-S(2)-F(4)	175.67(11)
F(6)-S(2)-F(5)	90.47(17)	F(6)-S(2)-F(7)

90.43(16)	F(6)-S(2)-F(8)	87.75(11)
F(7)-S(2)-C(14)	92.29(9)	F(7)-S(2)-F(8)
87.49(11)	F(8)-S(2)-C(14)	179.62(11)
F(9)-S(3)-C(34)	92.96(10)	F(9)-S(3)-F(13)
87.28(10)	F(9)-S(3)-F(10A)	85.2(3)
F(10B)-S(3)-C(34)	94.8(5)	F(10B)-S(3)-F(9)
103.1(6)	F(10B)-S(3)-F(11)	76.9(6)
F(10B)-S(3)-F(12)	165.3(6)	F(10B)-S(3)-F(13)
85.4(5)	F(11)-S(3)-C(34)	92.54(10)
F(11)-S(3)-F(9)	174.48(10)	F(11)-S(3)-F(12)
90.22(13)	F(11)-S(3)-F(13)	87.21(10)
F(11)-S(3)-F(10A)	95.1(3)	F(12)-S(3)-C(34)
92.70(9)	F(12)-S(3)-F(9)	89.11(10)
F(12)-S(3)-F(13)	87.10(10)	F(12)-S(3)-F(10A)
173.3(3)	F(13)-S(3)-C(34)	179.69(13)
F(13)-S(3)-F(10A)	89.07(19)	F(10A)-S(3)-C(34)
91.15(19)	F(14)-S(4)-C(74)	92.27(9)
F(14)-S(4)-F(16)	175.22(10)	F(14)-S(4)-F(18)
87.43(10)	F(15)-S(4)-C(74)	92.63(9)
F(15)-S(4)-F(14)	89.84(12)	F(15)-S(4)-F(16)
89.66(12)	F(15)-S(4)-F(17)	175.29(9)
F(15)-S(4)-F(18)	87.71(9)	F(16)-S(4)-C(74)
92.49(10)	F(16)-S(4)-F(18)	87.80(10)
F(17)-S(4)-C(74)	92.07(9)	F(17)-S(4)-F(14)
90.27(10)	F(17)-S(4)-F(16)	89.84(11)
F(17)-S(4)-F(18)	87.59(9)	F(18)-S(4)-C(74)
179.55(11)	F(19)-S(5)-C(54)	92.87(8)
F(19)-S(5)-F(22)	89.61(8)	F(19)-S(5)-F(23)
87.01(7)	F(20)-S(5)-C(54)	92.69(8)
F(20)-S(5)-F(19)	89.93(8)	F(20)-S(5)-F(21)
90.28(9)	F(20)-S(5)-F(22)	175.20(8)
F(20)-S(5)-F(23)	87.70(8)	F(21)-S(5)-C(54)
92.82(8)	F(21)-S(5)-F(19)	174.28(7)
F(21)-S(5)-F(22)	89.70(9)	F(21)-S(5)-F(23)
87.29(8)	F(22)-S(5)-C(54)	92.11(8)
F(23)-S(5)-C(54)	179.59(9)	F(23)-S(5)-F(22)
87.50(8)	Cl(1A)-C(77A)-Cl(2A)	111.9(3)
Cl(2B)-C(77B)-Cl(1B)	124(4)	Cl(3A)-C(78A)-Cl(4A)
113.8(4)	Cl(3B)-C(78B)-Cl(4B)	109.0(17)

12.2 Single crystal structure analysis of 6

Table 1. Crystal data and structure refinement of 6

Identification code	12241	
Empirical formula	$C_8 H_7 F_6 N_3 O_4 S_3$	
Color	colourless	
Formula weight	419.35 g·mol^{-1}	
Temperature	100(2) K	
Wavelength	0.71073 Å	
Crystal system	triclinic	
Space group	*P*-1, (No. 2)	
Unit cell dimensions	a = 10.4242(7) Å	α= 88.846(2)°.
	b = 11.0369(7) Å	β= 73.910(2)°.
	c = 13.8643(9) Å	γ = 86.750(2)°.
Volume	1530.13(17) Å^3	
Z	4	
Density (calculated)	1.820 Mg·m^{-3}	
Absorption coefficient	0.573 mm^{-1}	
F(000)	840 e	
Crystal size	0.114 x 0.111 x 0.110 mm^3	
θ range for data collection	1.529 to 33.869°.	
Index ranges	$-16 \le h \le 16$, $-17 \le k \le 17$, $-20 \le l \le 21$	
Reflections collected	57397	
Independent reflections	12191 [R_{int} = 0.0262]	
Reflections with I>2σ(I)	10697	
Completeness to θ = 25.242°	99.5 %	
Absorption correction	Gaussian	
Max. and min. transmission	0.95756 and 0.94732	
Refinement method	Full-matrix least-squares on F^2	
Data / restraints / parameters	12191 / 0 / 494	
Goodness-of-fit on F^2	1.123	
Final R indices [I>2σ(I)]	R_1 = 0.0408	wR^2 = 0.1032
R indices (all data)	R_1 = 0.0471	wR^2 = 0.1059
Extinction coefficient	n/a	
Largest diff. peak and hole	0.700 and -0.552 e·Å^{-3}	

Table 2. Bond lengths [Å] and angles [°].

_

C(1)-C(6)	1.388(2)	C(1)-C(2)
1.395(2)	C(1)-S(1)	1.7561(15)
C(2)-C(3)	1.386(2)	C(2)-H(2)
0.9500	C(3)-C(4)	1.391(3)
C(3)-H(3)	0.9500	C(4)-C(5)
1.384(3)	C(4)-H(4)	0.9500
C(5)-C(6)	1.386(2)	C(5)-H(5)
0.9500	C(6)-H(6)	0.9500
C(7)-F(4)	1.317(3)	C(7)-F(5)
1.324(2)	C(7)-F(6)	1.327(3)
C(7)-S(3)	1.836(2)	C(8)-F(2)
1.312(3)	C(8)-F(3)	1.319(3)
C(8)-F(1)	1.324(3)	C(8)-S(2)
1.830(2)	C(11)-C(16)	1.386(2)
C(11)-C(12)	1.386(2)	C(11)-S(4)
1.7579(16)	C(12)-C(13)	1.386(3)
C(12)-H(12)	0.9500	C(13)-C(14)
1.385(3)	C(13)-H(13)	0.9500
C(14)-C(15)	1.373(3)	C(14)-H(14)
0.9500	C(15)-C(16)	1.388(2)
C(15)-H(15)	0.9500	C(16)-H(16)
0.9500	C(17)-F(9)	1.308(3)
C(17)-F(7)	1.323(3)	C(17)-F(8)
1.325(3)	C(17)-S(5)	1.843(2)
N(1)-S(1)	1.5939(14)	N(1)-H(1A)
0.85(3)	N(1)-H(1B)	0.84(3)
N(2)-S(1)	1.5521(13)	N(2)-S(2)
1.6003(14)	N(3)-S(1)	1.5608(13)
N(3)-S(3)	1.5892(14)	N(4)-S(4)
1.5897(14)	N(4)-H(4A)	0.85(3)
N(4)-H(4B)	0.77(3)	N(5)-S(4)
1.5452(14)	N(5)-S(5)	1.5995(14)
N(6)-S(4)	1.5676(14)	N(6)-S(6)
1.5832(14)	O(1)-S(2)	1.4243(13)
O(2)-S(2)	1.4220(13)	O(3)-S(3)
1.4233(13)	O(4)-S(3)	1.4253(13)

O(5)-S(5)	1.4217(14)	O(6)-S(5)
1.4319(13)	O(8)-S(6)	1.4210(13)
S(6)-O(7A)	1.388(5)	S(6)-O(7B)
1.486(5)	S(6)-C(18B)	1.759(5)
S(6)-C(18A)	1.899(6)	C(18A)-F(12A)
1.306(7)	C(18A)-F(10A)	1.316(6)
C(18A)-F(11A)	1.338(7)	C(18B)-F(12B)
1.306(8)	C(18B)-F(10B)	1.323(7)
C(18B)-F(11B)	1.330(7)	

C(6)-C(1)-C(2)	122.78(15)	C(6)-C(1)-S(1)
120.85(12)	C(2)-C(1)-S(1)	116.36(12)
C(3)-C(2)-C(1)	117.86(16)	C(3)-C(2)-H(2)
121.1	C(1)-C(2)-H(2)	121.1
C(2)-C(3)-C(4)	120.30(16)	C(2)-C(3)-H(3)
119.9	C(4)-C(3)-H(3)	119.9
C(5)-C(4)-C(3)	120.59(16)	C(5)-C(4)-H(4)
119.7	C(3)-C(4)-H(4)	119.7
C(4)-C(5)-C(6)	120.48(17)	C(4)-C(5)-H(5)
119.8	C(6)-C(5)-H(5)	119.8
C(5)-C(6)-C(1)	117.99(16)	C(5)-C(6)-H(6)
121.0	C(1)-C(6)-H(6)	121.0
F(4)-C(7)-F(5)	109.08(19)	F(4)-C(7)-F(6)
108.96(19)	F(5)-C(7)-F(6)	109.1(2)
F(4)-C(7)-S(3)	110.98(18)	F(5)-C(7)-S(3)
108.38(14)	F(6)-C(7)-S(3)	110.31(15)
F(2)-C(8)-F(3)	108.98(19)	F(2)-C(8)-F(1)
108.6(2)	F(3)-C(8)-F(1)	109.5(2)
F(2)-C(8)-S(2)	110.17(18)	F(3)-C(8)-S(2)
108.48(16)	F(1)-C(8)-S(2)	111.12(15)
C(16)-C(11)-C(12)	122.11(15)	C(16)-C(11)-S(4)
121.22(12)	C(12)-C(11)-S(4)	116.67(12)
C(13)-C(12)-C(11)	118.81(17)	C(13)-C(12)-H(12)
120.6	C(11)-C(12)-H(12)	120.6
C(14)-C(13)-C(12)	119.84(17)	C(14)-C(13)-H(13)
120.1	C(12)-C(13)-H(13)	120.1
C(15)-C(14)-C(13)	120.36(17)	C(15)-C(14)-H(14)
119.8	C(13)-C(14)-H(14)	119.8
C(14)-C(15)-C(16)	121.12(17)	C(14)-C(15)-H(15)

119.4	C(16)-C(15)-H(15)	119.4
C(11)-C(16)-C(15)	117.73(16)	C(11)-C(16)-H(16)
121.1	C(15)-C(16)-H(16)	121.1
F(9)-C(17)-F(7)	109.6(2)	F(9)-C(17)-F(8)
109.6(2)	F(7)-C(17)-F(8)	108.71(19)
F(9)-C(17)-S(5)	110.38(15)	F(7)-C(17)-S(5)
110.15(16)	F(8)-C(17)-S(5)	108.44(17)
S(1)-N(1)-H(1A)	112.2(18)	S(1)-N(1)-H(1B)
113.6(19)	H(1A)-N(1)-H(1B)	116(3)
S(1)-N(2)-S(2)	123.10(8)	S(1)-N(3)-S(3)
123.06(9)	S(4)-N(4)-H(4A)	112.6(18)
S(4)-N(4)-H(4B)	114(2)	H(4A)-N(4)-H(4B)
119(3)	S(4)-N(5)-S(5)	123.65(9)
S(4)-N(6)-S(6)	123.91(9)	N(2)-S(1)-N(3)
121.77(7)	N(2)-S(1)-N(1)	111.03(8)
N(3)-S(1)-N(1)	99.93(7)	N(2)-S(1)-C(1)
102.13(7)	N(3)-S(1)-C(1)	110.73(8)
N(1)-S(1)-C(1)	111.47(7)	O(2)-S(2)-O(1)
120.20(9)	O(2)-S(2)-N(2)	114.46(8)
O(1)-S(2)-N(2)	107.74(8)	O(2)-S(2)-C(8)
106.27(11)	O(1)-S(2)-C(8)	104.24(10)
N(2)-S(2)-C(8)	101.79(9)	O(3)-S(3)-O(4)
119.67(9)	O(3)-S(3)-N(3)	107.70(8)
O(4)-S(3)-N(3)	114.26(7)	O(3)-S(3)-C(7)
104.44(10)	O(4)-S(3)-C(7)	104.93(11)
N(3)-S(3)-C(7)	104.19(9)	N(5)-S(4)-N(6)
120.04(8)	N(5)-S(4)-N(4)	113.06(8)
N(6)-S(4)-N(4)	99.46(8)	N(5)-S(4)-C(11)
101.87(7)	N(6)-S(4)-C(11)	113.37(8)
N(4)-S(4)-C(11)	109.12(8)	O(5)-S(5)-O(6)
119.81(9)	O(5)-S(5)-N(5)	115.11(8)
O(6)-S(5)-N(5)	108.04(7)	O(5)-S(5)-C(17)
107.30(11)	O(6)-S(5)-C(17)	104.46(10)
N(5)-S(5)-C(17)	99.60(9)	O(7A)-S(6)-O(8)
128.1(2)	O(8)-S(6)-O(7B)	110.63(19)
O(7A)-S(6)-N(6)	113.7(2)	O(8)-S(6)-N(6)
108.91(8)	O(7B)-S(6)-N(6)	113.94(19)
O(8)-S(6)-C(18B)	107.9(2)	O(7B)-S(6)-C(18B)
106.0(3)	N(6)-S(6)-C(18B)	109.3(2)

O(7A)-S(6)-C(18A)	104.2(3)	O(8)-S(6)-C(18A)
98.73(17)	N(6)-S(6)-C(18A)	96.7(2)
F(12A)-C(18A)-F(10A)	110.0(6)	F(12A)-C(18A)-F(11A)
108.8(5)	F(10A)-C(18A)-F(11A)	109.3(5)
F(12A)-C(18A)-S(6)	111.0(4)	F(10A)-C(18A)-S(6)
111.5(4)	F(11A)-C(18A)-S(6)	106.2(4)
F(12B)-C(18B)-F(10B)	108.8(6)	F(12B)-C(18B)-F(11B)
109.2(5)	F(10B)-C(18B)-F(11B)	108.6(6)
F(12B)-C(18B)-S(6)	109.8(5)	F(10B)-C(18B)-S(6)
110.5(4)	F(11B)-C(18B)-S(6)	110.0(4)

_

12.3 Single crystal structure analysis of IDPii 7a

Table 1. Crystal data and structure refinement of IDPii 7a

Identification code	12144	
Empirical formula	$C_{89} H_{72} Cl_2 F_{12} N_8 O_{12} P_2 S_6$	
Color	colourless	
Formula weight	1998.74 g·mol^{-1}	
Temperature	100(2) K	
Wavelength	0.71073 Å	
Crystal system	orthorhombic	
Space group	$P2_12_12_1$, (No. 19)	
Unit cell dimensions	a = 13.169(2) Å	α= 90°.
	b = 15.101(3) Å	β= 90°.
	c = 44.396(7) Å	γ = 90°.
Volume	8829(3) Å^3	
Z	4	
Density (calculated)	1.504 Mg·m^{-3}	
Absorption coefficient	0.344 mm^{-1}	
F(000)	4104 e	
Crystal size	0.109 x 0.083 x 0.032 mm^3	
θ range for data collection	1.613 to 33.578°.	
Index ranges	$-20 \le h \le 20$, $-23 \le k \le 23$, $-69 \le l \le 68$	
Reflections collected	264447	
Independent reflections	34630 [R_{int} = 0.0663]	
Reflections with I>2σ(I)	30152	
Completeness to θ = 25.242°	99.8 %	
Absorption correction	Gaussian	
Max. and min. transmission	0.98987 and 0.96681	
Refinement method	Full-matrix least-squares on F^2	
Data / restraints / parameters	34630 / 6 / 1214	
Goodness-of-fit on F^2	1.134	
Final R indices [I>2σ(I)]	R_1 = 0.0549	wR^2 = 0.1251
R indices (all data)	R_1 = 0.0674	wR^2 = 0.1301
Absolute structure parameter	0.021(10)	
Extinction coefficient	n/a	
Largest diff. peak and hole	0.750 and -0.607 e·Å^{-3}	

Table 2. Bond lengths [Å] and angles [°].

_

C(1)-C(2)	1.374(4)	C(1)-C(16)
1.429(4)	C(1)-C(17)	1.490(4)
C(2)-O(1)	1.412(3)	C(2)-C(3)
1.415(4)	C(3)-C(10)	1.388(4)
C(3)-C(4)	1.492(4)	C(4)-C(9)
1.387(4)	C(4)-C(5)	1.397(4)
C(5)-C(6)	1.391(5)	C(5)-H(5)
0.9500	C(6)-C(7)	1.394(5)
C(6)-H(6)	0.9500	C(7)-C(8)
1.387(5)	C(7)-H(7)	0.9500
C(8)-C(9)	1.389(5)	C(8)-H(8)
0.9500	C(9)-H(9)	0.9500
C(10)-C(11)	1.418(5)	C(10)-H(10)
0.9500	C(11)-C(16)	1.415(4)
C(11)-C(12)	1.431(4)	C(12)-C(13)
1.364(5)	C(12)-H(12)	0.9500
C(13)-C(14)	1.411(5)	C(13)-H(13)
0.9500	C(14)-C(15)	1.374(4)
C(14)-H(14)	0.9500	C(15)-C(16)
1.425(4)	C(15)-H(15)	0.9500
C(17)-C(18)	1.372(4)	C(17)-C(32)
1.433(4)	C(18)-O(2)	1.398(3)
C(18)-C(19)	1.418(4)	C(19)-C(26)
1.387(4)	C(19)-C(20)	1.492(4)
C(20)-C(21)	1.397(4)	C(20)-C(25)
1.410(4)	C(21)-C(22)	1.398(4)
C(21)-H(21)	0.9500	C(22)-C(23)
1.384(5)	C(22)-H(22)	0.9500
C(23)-C(24)	1.394(6)	C(23)-H(23)
0.9500	C(24)-C(25)	1.383(4)
C(24)-H(24)	0.9500	C(25)-H(25)
0.9500	C(26)-C(27)	1.411(4)
C(26)-H(26)	0.9500	C(27)-C(32)
1.416(4)	C(27)-C(28)	1.424(4)
C(28)-C(29)	1.367(5)	C(28)-H(28)
0.9500	C(29)-C(30)	1.406(5)

C(29)-H(29)	0.9500	C(30)-C(31)
1.371(4)	C(30)-H(30)	0.9500
C(31)-C(32)	1.424(4)	C(31)-H(31)
0.9500	C(33)-C(34)	1.376(4)
C(33)-C(48)	1.438(4)	C(33)-C(49)
1.485(4)	C(34)-C(35)	1.414(4)
C(34)-O(7)	1.415(3)	C(35)-C(42)
1.379(4)	C(35)-C(36)	1.484(4)
C(36)-C(41)	1.398(4)	C(36)-C(37)
1.398(4)	C(37)-C(38)	1.388(5)
C(37)-H(37)	0.9500	C(38)-C(39)
1.383(6)	C(38)-H(38)	0.9500
C(39)-C(40)	1.390(5)	C(39)-H(39)
0.9500	C(40)-C(41)	1.386(5)
C(40)-H(40)	0.9500	C(41)-H(41)
0.9500	C(42)-C(43)	1.414(4)
C(42)-H(42)	0.9500	C(43)-C(44)
1.419(4)	C(43)-C(48)	1.423(4)
C(44)-C(45)	1.373(4)	C(44)-H(44)
0.9500	C(45)-C(46)	1.414(5)
C(45)-H(45)	0.9500	C(46)-C(47)
1.372(4)	C(46)-H(46)	0.9500
C(47)-C(48)	1.422(4)	C(47)-H(47)
0.9500	C(49)-C(50)	1.378(4)
C(49)-C(64)	1.442(4)	C(50)-O(8)
1.402(3)	C(50)-C(51)	1.419(4)
C(51)-C(58)	1.380(4)	C(51)-C(52)
1.479(4)	C(52)-C(53)	1.396(4)
C(52)-C(57)	1.407(4)	C(53)-C(54)
1.391(4)	C(53)-H(53)	0.9500
C(54)-C(55)	1.381(5)	C(54)-H(54)
0.9500	C(55)-C(56)	1.397(5)
C(55)-H(55)	0.9500	C(56)-C(57)
1.386(4)	C(56)-H(56)	0.9500
C(57)-H(57)	0.9500	C(58)-C(59)
1.410(4)	C(58)-H(58)	0.9500
C(59)-C(64)	1.416(4)	C(59)-C(60)
1.427(4)	C(60)-C(61)	1.365(4)
C(60)-H(60)	0.9500	C(61)-C(62)

1.413(5)	C(61)-H(61)	0.9500
C(62)-C(63)	1.370(4)	C(62)-H(62)
0.9500	C(63)-C(64)	1.418(4)
C(63)-H(63)	0.9500	C(65)-C(66)
1.389(4)	C(65)-C(70)	1.391(4)
C(65)-S(1)	1.769(3)	C(66)-C(67)
1.380(4)	C(66)-H(66)	0.9500
C(67)-C(68)	1.397(5)	C(67)-H(67)
0.9500	C(68)-C(69)	1.388(5)
C(68)-H(68)	0.9500	C(69)-C(70)
1.382(4)	C(69)-H(69)	0.9500
C(70)-H(70)	0.9500	C(71)-C(72)
1.379(4)	C(71)-C(76)	1.392(4)
C(71)-S(4)	1.767(3)	C(72)-C(73)
1.391(4)	C(72)-H(72)	0.9500
C(73)-C(74)	1.384(5)	C(73)-H(73)
0.9500	C(74)-C(75)	1.388(5)
C(74)-H(74)	0.9500	C(75)-C(76)
1.389(4)	C(75)-H(75)	0.9500
C(76)-H(76)	0.9500	C(77)-F(2)
1.314(4)	C(77)-F(3)	1.323(5)
C(77)-F(1)	1.343(4)	C(77)-S(2)
1.841(4)	C(78)-F(5)	1.308(7)
C(78)-F(6)	1.327(6)	C(78)-F(4)
1.327(5)	C(78)-S(3)	1.844(4)
C(79)-F(8)	1.322(4)	C(79)-F(9)
1.328(4)	C(79)-F(7)	1.340(4)
C(79)-S(5)	1.836(3)	C(81)-N(8)
1.515(5)	C(81)-C(82)	1.522(5)
C(81)-H(81A)	0.9900	C(81)-H(81B)
0.9900	C(82)-H(82A)	0.9800
C(82)-H(82B)	0.9800	C(82)-H(82C)
0.9800	C(83)-N(8)	1.509(5)
C(83)-C(85)	1.535(7)	C(83)-C(84)
1.546(6)	C(83)-H(83)	1.0000
C(84)-H(84A)	0.9800	C(84)-H(84B)
0.9800	C(84)-H(84C)	0.9800
C(85)-H(85A)	0.9800	C(85)-H(85B)
0.9800	C(85)-H(85C)	0.9800

C(86)-C(87)	1.521(6)	C(86)-C(88)
1.524(6)	C(86)-N(8)	1.541(5)
C(86)-H(86)	1.0000	C(87)-H(87A)
0.9800	C(87)-H(87B)	0.9800
C(87)-H(87C)	0.9800	C(88)-H(88A)
0.9800	C(88)-H(88B)	0.9800
C(88)-H(88C)	0.9800	C(89)-Cl(2)
1.753(5)	C(89)-Cl(1)	1.761(5)
C(89)-H(89A)	0.9900	C(89)-H(89B)
0.9900	N(1)-P(2)	1.539(3)
N(1)-P(1)	1.545(2)	N(2)-S(1)
1.531(2)	N(2)-P(1)	1.583(2)
N(3)-S(1)	1.573(3)	N(3)-S(2)
1.582(2)	N(4)-S(1)	1.567(3)
N(4)-S(3)	1.587(3)	N(5)-S(4)
1.530(3)	N(5)-P(2)	1.588(3)
N(6)-S(5)	1.567(3)	N(6)-S(4)
1.583(3)	N(7)-S(4)	1.551(3)
N(7)-S(6A)	1.582(3)	N(7)-S(6B)
1.666(4)	N(8)-H(8A)	1.0000
O(1)-P(1)	1.591(2)	O(2)-P(1)
1.593(2)	O(3)-S(2)	1.431(3)
O(4)-S(2)	1.432(3)	O(5)-S(3)
1.422(3)	O(6)-S(3)	1.424(3)
O(7)-P(2)	1.588(2)	O(8)-P(2)
1.584(2)	O(9)-S(5)	1.442(3)
O(10)-S(5)	1.426(2)	C(80A)-F(10A)
1.309(7)	C(80A)-F(11A)	1.321(10)
C(80A)-F(12A)	1.333(7)	C(80A)-S(6A)
1.831(6)	O(11A)-S(6A)	1.438(7)
O(12A)-S(6A)	1.426(6)	C(80B)-F(10B)
1.276(17)	C(80B)-F(11B)	1.334(16)
C(80B)-F(12B)	1.339(16)	C(80B)-S(6B)
1.819(14)	O(11B)-S(6B)	1.40(2)
O(12B)-S(6B)	1.411(12)	

C(2)-C(1)-C(16)	118.5(3)	C(2)-C(1)-C(17)
119.9(2)	C(16)-C(1)-C(17)	121.6(3)
C(1)-C(2)-O(1)	117.6(2)	C(1)-C(2)-C(3)

124.7(3)	O(1)-C(2)-C(3)	117.8(3)
C(10)-C(3)-C(2)	116.0(3)	C(10)-C(3)-C(4)
121.0(3)	C(2)-C(3)-C(4)	123.0(3)
C(9)-C(4)-C(5)	119.1(3)	C(9)-C(4)-C(3)
119.4(3)	C(5)-C(4)-C(3)	121.4(3)
C(6)-C(5)-C(4)	120.4(3)	C(6)-C(5)-H(5)
119.8	C(4)-C(5)-H(5)	119.8
C(5)-C(6)-C(7)	120.0(3)	C(5)-C(6)-H(6)
120.0	C(7)-C(6)-H(6)	120.0
C(8)-C(7)-C(6)	119.5(3)	C(8)-C(7)-H(7)
120.3	C(6)-C(7)-H(7)	120.3
C(7)-C(8)-C(9)	120.4(3)	C(7)-C(8)-H(8)
119.8	C(9)-C(8)-H(8)	119.8
C(4)-C(9)-C(8)	120.6(3)	C(4)-C(9)-H(9)
119.7	C(8)-C(9)-H(9)	119.7
C(3)-C(10)-C(11)	121.8(3)	C(3)-C(10)-H(10)
119.1	C(11)-C(10)-H(10)	119.1
C(16)-C(11)-C(10)	120.4(3)	C(16)-C(11)-C(12)
119.2(3)	C(10)-C(11)-C(12)	120.4(3)
C(13)-C(12)-C(11)	120.2(3)	C(13)-C(12)-H(12)
119.9	C(11)-C(12)-H(12)	119.9
C(12)-C(13)-C(14)	120.7(3)	C(12)-C(13)-H(13)
119.7	C(14)-C(13)-H(13)	119.7
C(15)-C(14)-C(13)	120.7(3)	C(15)-C(14)-H(14)
119.6	C(13)-C(14)-H(14)	119.6
C(14)-C(15)-C(16)	120.0(3)	C(14)-C(15)-H(15)
120.0	C(16)-C(15)-H(15)	120.0
C(11)-C(16)-C(15)	119.2(3)	C(11)-C(16)-C(1)
118.3(3)	C(15)-C(16)-C(1)	122.4(3)
C(18)-C(17)-C(32)	118.8(3)	C(18)-C(17)-C(1)
120.8(3)	C(32)-C(17)-C(1)	120.4(2)
C(17)-C(18)-O(2)	118.4(2)	C(17)-C(18)-C(19)
123.5(3)	O(2)-C(18)-C(19)	118.1(2)
C(26)-C(19)-C(18)	117.0(3)	C(26)-C(19)-C(20)
119.1(3)	C(18)-C(19)-C(20)	123.9(3)
C(21)-C(20)-C(25)	119.0(3)	C(21)-C(20)-C(19)
123.0(3)	C(25)-C(20)-C(19)	117.9(3)
C(20)-C(21)-C(22)	120.0(3)	C(20)-C(21)-H(21)
120.0	C(22)-C(21)-H(21)	120.0

C(23)-C(22)-C(21)	120.4(3)	C(23)-C(22)-H(22)
119.8	C(21)-C(22)-H(22)	119.8
C(22)-C(23)-C(24)	119.9(3)	C(22)-C(23)-H(23)
120.1	C(24)-C(23)-H(23)	120.1
C(25)-C(24)-C(23)	120.2(3)	C(25)-C(24)-H(24)
119.9	C(23)-C(24)-H(24)	119.9
C(24)-C(25)-C(20)	120.4(3)	C(24)-C(25)-H(25)
119.8	C(20)-C(25)-H(25)	119.8
C(19)-C(26)-C(27)	122.0(3)	C(19)-C(26)-H(26)
119.0	C(27)-C(26)-H(26)	119.0
C(26)-C(27)-C(32)	119.7(3)	C(26)-C(27)-C(28)
120.7(3)	C(32)-C(27)-C(28)	119.6(3)
C(29)-C(28)-C(27)	120.4(3)	C(29)-C(28)-H(28)
119.8	C(27)-C(28)-H(28)	119.8
C(28)-C(29)-C(30)	120.4(3)	C(28)-C(29)-H(29)
119.8	C(30)-C(29)-H(29)	119.8
C(31)-C(30)-C(29)	120.4(3)	C(31)-C(30)-H(30)
119.8	C(29)-C(30)-H(30)	119.8
C(30)-C(31)-C(32)	120.9(3)	C(30)-C(31)-H(31)
119.6	C(32)-C(31)-H(31)	119.6
C(27)-C(32)-C(31)	118.2(3)	C(27)-C(32)-C(17)
118.9(3)	C(31)-C(32)-C(17)	122.9(3)
C(34)-C(33)-C(48)	118.0(3)	C(34)-C(33)-C(49)
120.8(2)	C(48)-C(33)-C(49)	121.3(3)
C(33)-C(34)-C(35)	124.4(2)	C(33)-C(34)-O(7)
117.5(2)	C(35)-C(34)-O(7)	118.1(2)
C(42)-C(35)-C(34)	116.8(3)	C(42)-C(35)-C(36)
119.8(3)	C(34)-C(35)-C(36)	123.4(2)
C(41)-C(36)-C(37)	118.6(3)	C(41)-C(36)-C(35)
119.3(3)	C(37)-C(36)-C(35)	122.0(3)
C(38)-C(37)-C(36)	120.1(3)	C(38)-C(37)-H(37)
119.9	C(36)-C(37)-H(37)	119.9
C(39)-C(38)-C(37)	120.7(3)	C(39)-C(38)-H(38)
119.7	C(37)-C(38)-H(38)	119.7
C(38)-C(39)-C(40)	119.8(3)	C(38)-C(39)-H(39)
120.1	C(40)-C(39)-H(39)	120.1
C(41)-C(40)-C(39)	119.7(3)	C(41)-C(40)-H(40)
120.1	C(39)-C(40)-H(40)	120.1
C(40)-C(41)-C(36)	121.0(3)	C(40)-C(41)-H(41)

119.5	C(36)-C(41)-H(41)	119.5
C(35)-C(42)-C(43)	122.1(3)	C(35)-C(42)-H(42)
119.0	C(43)-C(42)-H(42)	119.0
C(42)-C(43)-C(44)	121.0(3)	C(42)-C(43)-C(48)
119.8(3)	C(44)-C(43)-C(48)	119.2(3)
C(45)-C(44)-C(43)	120.5(3)	C(45)-C(44)-H(44)
119.7	C(43)-C(44)-H(44)	119.7
C(44)-C(45)-C(46)	120.4(3)	C(44)-C(45)-H(45)
119.8	C(46)-C(45)-H(45)	119.8
C(47)-C(46)-C(45)	120.3(3)	C(47)-C(46)-H(46)
119.9	C(45)-C(46)-H(46)	119.9
C(46)-C(47)-C(48)	120.8(3)	C(46)-C(47)-H(47)
119.6	C(48)-C(47)-H(47)	119.6
C(47)-C(48)-C(43)	118.7(3)	C(47)-C(48)-C(33)
122.6(3)	C(43)-C(48)-C(33)	118.6(3)
C(50)-C(49)-C(64)	118.3(3)	C(50)-C(49)-C(33)
120.5(3)	C(64)-C(49)-C(33)	121.1(2)
C(49)-C(50)-O(8)	118.2(2)	C(49)-C(50)-C(51)
123.8(3)	O(8)-C(50)-C(51)	117.9(2)
C(58)-C(51)-C(50)	117.0(2)	C(58)-C(51)-C(52)
119.2(2)	C(50)-C(51)-C(52)	123.7(3)
C(53)-C(52)-C(57)	118.6(3)	C(53)-C(52)-C(51)
122.6(3)	C(57)-C(52)-C(51)	118.5(3)
C(54)-C(53)-C(52)	120.5(3)	C(54)-C(53)-H(53)
119.8	C(52)-C(53)-H(53)	119.8
C(55)-C(54)-C(53)	120.6(3)	C(55)-C(54)-H(54)
119.7	C(53)-C(54)-H(54)	119.7
C(54)-C(55)-C(56)	119.6(3)	C(54)-C(55)-H(55)
120.2	C(56)-C(55)-H(55)	120.2
C(57)-C(56)-C(55)	120.2(3)	C(57)-C(56)-H(56)
119.9	C(55)-C(56)-H(56)	119.9
C(56)-C(57)-C(52)	120.4(3)	C(56)-C(57)-H(57)
119.8	C(52)-C(57)-H(57)	119.8
C(51)-C(58)-C(59)	121.9(3)	C(51)-C(58)-H(58)
119.0	C(59)-C(58)-H(58)	119.0
C(58)-C(59)-C(64)	120.3(3)	C(58)-C(59)-C(60)
120.2(3)	C(64)-C(59)-C(60)	119.5(3)
C(61)-C(60)-C(59)	120.4(3)	C(61)-C(60)-H(60)
119.8	C(59)-C(60)-H(60)	119.8

C(60)-C(61)-C(62)	120.0(3)	C(60)-C(61)-H(61)
120.0	C(62)-C(61)-H(61)	120.0
C(63)-C(62)-C(61)	120.6(3)	C(63)-C(62)-H(62)
119.7	C(61)-C(62)-H(62)	119.7
C(62)-C(63)-C(64)	120.8(3)	C(62)-C(63)-H(63)
119.6	C(64)-C(63)-H(63)	119.6
C(59)-C(64)-C(63)	118.4(3)	C(59)-C(64)-C(49)
118.4(2)	C(63)-C(64)-C(49)	123.1(3)
C(66)-C(65)-C(70)	121.8(3)	C(66)-C(65)-S(1)
120.2(2)	C(70)-C(65)-S(1)	118.0(2)
C(67)-C(66)-C(65)	118.4(3)	C(67)-C(66)-H(66)
120.8	C(65)-C(66)-H(66)	120.8
C(66)-C(67)-C(68)	120.6(3)	C(66)-C(67)-H(67)
119.7	C(68)-C(67)-H(67)	119.7
C(69)-C(68)-C(67)	120.1(3)	C(69)-C(68)-H(68)
119.9	C(67)-C(68)-H(68)	119.9
C(70)-C(69)-C(68)	119.9(3)	C(70)-C(69)-H(69)
120.0	C(68)-C(69)-H(69)	120.0
C(69)-C(70)-C(65)	119.2(3)	C(69)-C(70)-H(70)
120.4	C(65)-C(70)-H(70)	120.4
C	(72)-C(71)-C(76)	122.3(3)
C(72)-C(71)-S(4)	119.9(2)	C(76)-C(71)-S(4)
117.9(2)	C(71)-C(72)-C(73)	118.5(3)
C(71)-C(72)-H(72)	120.8	C(73)-C(72)-H(72)
120.8	C(74)-C(73)-C(72)	120.3(3)
C(74)-C(73)-H(73)	119.9	C(72)-C(73)-H(73)
119.9	C(73)-C(74)-C(75)	120.5(3)
C(73)-C(74)-H(74)	119.8	C(75)-C(74)-H(74)
119.8	C(74)-C(75)-C(76)	120.1(3)
C(74)-C(75)-H(75)	120.0	C(76)-C(75)-H(75)
120.0	C(75)-C(76)-C(71)	118.4(3)
C(75)-C(76)-H(76)	120.8	C(71)-C(76)-H(76)
120.8	F(2)-C(77)-F(3)	108.4(3)
F(2)-C(77)-F(1)	107.7(3)	F(3)-C(77)-F(1)
108.0(3)	F(2)-C(77)-S(2)	113.2(3)
F(3)-C(77)-S(2)	111.1(2)	F(1)-C(77)-S(2)
108.4(3)	F(5)-C(78)-F(6)	109.6(4)
F(5)-C(78)-F(4)	107.8(4)	F(6)-C(78)-F(4)
107.8(5)	F(5)-C(78)-S(3)	111.4(4)

F(6)-C(78)-S(3)	111.1(3)	F(4)-C(78)-S(3)
109.0(3)	F(8)-C(79)-F(9)	109.0(3)
F(8)-C(79)-F(7)	108.4(3)	F(9)-C(79)-F(7)
107.8(3)	F(8)-C(79)-S(5)	112.7(2)
F(9)-C(79)-S(5)	111.0(2)	F(7)-C(79)-S(5)
107.7(2)	N(8)-C(81)-C(82)	111.7(3)
N(8)-C(81)-H(81A)	109.3	C(82)-C(81)-H(81A)
109.3	N(8)-C(81)-H(81B)	109.3
C(82)-C(81)-H(81B)	109.3	H(81A)-C(81)-H(81B)
107.9	C(81)-C(82)-H(82A)	109.5
C(81)-C(82)-H(82B)	109.5	H(82A)-C(82)-H(82B)
109.5	C(81)-C(82)-H(82C)	109.5
H(82A)-C(82)-H(82C)	109.5	H(82B)-C(82)-H(82C)
109.5	N(8)-C(83)-C(85)	107.7(3)
N(8)-C(83)-C(84)	111.5(3)	C(85)-C(83)-C(84)
111.8(4)		N(8)-C(83)-H(83)
108.6	C(85)-C(83)-H(83)	
108.6	C(84)-C(83)-H(83)	108.6
C(83)-C(84)-H(84A)	109.5	C(83)-C(84)-H(84B)
109.5	H(84A)-C(84)-H(84B)	109.5
C(83)-C(84)-H(84C)	109.5	H(84A)-C(84)-H(84C)
109.5	H(84B)-C(84)-H(84C)	109.5
C(83)-C(85)-H(85A)	109.5	C(83)-C(85)-H(85B)
109.5	H(85A)-C(85)-H(85B)	109.5
C(83)-C(85)-H(85C)	109.5	H(85A)-C(85)-H(85C)
109.5	H(85B)-C(85)-H(85C)	109.5
C(87)-C(86)-C(88)	112.8(4)	C(87)-C(86)-N(8)
112.1(3)	C(88)-C(86)-N(8)	109.4(3)
C(87)-C(86)-H(86)	107.4	C(88)-C(86)-H(86)
107.4	N(8)-C(86)-H(86)	107.4
C(86)-C(87)-H(87A)	109.5	C(86)-C(87)-H(87B)
109.5	H(87A)-C(87)-H(87B)	109.5
C(86)-C(87)-H(87C)	109.5	H(87A)-C(87)-H(87C)
109.5	H(87B)-C(87)-H(87C)	109.5
C(86)-C(88)-H(88A)	109.5	C(86)-C(88)-H(88B)
109.5	H(88A)-C(88)-H(88B)	109.5
C(86)-C(88)-H(88C)	109.5	H(88A)-C(88)-H(88C)
109.5	H(88B)-C(88)-H(88C)	109.5
Cl(2)-C(89)-Cl(1)	111.2(2)	Cl(2)-C(89)-H(89A)

109.4	Cl(1)-C(89)-H(89A)	109.4
Cl(2)-C(89)-H(89B)	109.4	Cl(1)-C(89)-H(89B)
109.4	H(89A)-C(89)-H(89B)	108.0
P(2)-N(1)-P(1)	149.51(18)	S(1)-N(2)-P(1)
132.89(17)	S(1)-N(3)-S(2)	127.92(17)
S(1)-N(4)-S(3)	123.71(17)	S(4)-N(5)-P(2)
132.80(18)	S(5)-N(6)-S(4)	126.90(17)
S(4)-N(7)-S(6A)	121.63(19)	S(4)-N(7)-S(6B)
128.6(2)	C(83)-N(8)-C(81)	111.7(3)
C(83)-N(8)-C(86)	114.0(3)	C(81)-N(8)-C(86)
110.7(3)	C(83)-N(8)-H(8A)	106.6
C(81)-N(8)-H(8A)	106.6	C(86)-N(8)-H(8A)
106.6	C(2)-O(1)-P(1)	114.73(17)
C(18)-O(2)-P(1)	122.81(18)	C(34)-O(7)-P(2)
115.81(17)		C(50)-O(8)-P(2)
121.95(19)	N(1)-P(1)-N(2)	114.71(14)
N(1)-P(1)-O(1)	111.12(13)	
N(2)-P(1)-O(1)	107.95(12)	N(1)-P(1)-O(2)
105.77(13)	N(2)-P(1)-O(2)	112.65(13)
O(1)-P(1)-O(2)	104.18(11)	N(1)-P(2)-O(8)
107.25(13)	N(1)-P(2)-O(7)	111.18(13)
O(8)-P(2)-O(7)	103.82(11)	N(1)-P(2)-N(5)
114.27(15)	O(8)-P(2)-N(5)	111.54(15)
O(7)-P(2)-N(5)	108.29(13)	N(2)-S(1)-N(4)
114.55(15)	N(2)-S(1)-N(3)	110.61(14)
N(4)-S(1)-N(3)	108.58(14)	N(2)-S(1)-C(65)
104.63(14)	N(4)-S(1)-C(65)	110.72(14)
N(3)-S(1)-C(65)	107.51(14)	O(3)-S(2)-O(4)
119.05(16)	O(3)-S(2)-N(3)	107.89(15)
O(4)-S(2)-N(3)	116.09(15)	O(3)-S(2)-C(77)
103.16(17)	O(4)-S(2)-C(77)	105.13(17)
N(3)-S(2)-C(77)	103.46(15)	O(5)-S(3)-O(6)
119.61(17)	O(5)-S(3)-N(4)	109.72(17)
O(6)-S(3)-N(4)	114.79(15)	O(5)-S(3)-C(78)
104.6(2)	O(6)-S(3)-C(78)	103.2(2)
N(4)-S(3)-C(78)	102.68(18)	N(5)-S(4)-N(7)
115.99(18)	N(5)-S(4)-N(6)	110.47(15)
N(7)-S(4)-N(6)	107.84(15)	N(5)-S(4)-C(71)
104.59(14)	N(7)-S(4)-C(71)	110.09(16)

N(6)-S(4)-C(71)	107.56(14)	O(10)-S(5)-O(9)
117.35(15)	O(10)-S(5)-N(6)	117.28(14)
O(9)-S(5)-N(6)	107.35(15)	O(10)-S(5)-C(79)
105.05(16)	O(9)-S(5)-C(79)	102.32(15)
N(6)-S(5)-C(79)	105.75(15)	F(10A)-C(80A)-F(11A)
110.5(6)	F(10A)-C(80A)-F(12A)	107.6(6)
F(11A)-C(80A)-F(12A)	109.0(6)	F(10A)-C(80A)-S(6A)
111.4(5)	F(11A)-C(80A)-S(6A)	110.6(4)
F(12A)-C(80A)-S(6A)	107.6(5)	O(12A)-S(6A)-O(11A)
117.9(4)	O(12A)-S(6A)-N(7)	116.3(3)
O(11A)-S(6A)-N(7)	112.7(3)	O(12A)-S(6A)-C(80A)
106.7(4)	O(11A)-S(6A)-C(80A)	106.8(4)
N(7)-S(6A)-C(80A)	92.4(2)	F(10B)-C(80B)-F(11B)
110.3(12)	F(10B)-C(80B)-F(12B)	108.0(11)
F(11B)-C(80B)-F(12B)	106.9(11)	F(10B)-C(80B)-S(6B)
109.9(10)	F(11B)-C(80B)-S(6B)	110.1(9)
F(12B)-C(80B)-S(6B)	111.6(10)	O(11B)-S(6B)-O(12B)
117.7(9)	O(11B)-S(6B)-N(7)	110.9(8)
O(12B)-S(6B)-N(7)	119.8(5)	O(11B)-S(6B)-C(80B)
105.0(10)	O(12B)-S(6B)-C(80B)	105.9(6)
N(7)-S(6B)-C(80B)	93.3(4)	
